# Evaluating agreement between evidence from randomised controlled trials and cohort studies in nutrition: a meta-research replication study

**DOI:** 10.1007/s10654-023-01058-5

**Published:** 2024-01-04

**Authors:** Julia Stadelmaier, Jessica Beyerbach, Isabelle Roux, Louisa Harms, Julian Eble, Adriani Nikolakopoulou, Lukas Schwingshackl

**Affiliations:** 1 Institute for Evidence in Medicine, Medical Center - University of Freiburg, Faculty of Medicine, University of Freiburg, Freiburg, Germany; 2 Institute of Medical Biometry and Statistics, Medical Center - University of Freiburg, Faculty of Medicine, University of Freiburg, Freiburg, Germany

**Keywords:** Meta-epidemiological study, Concordance, Randomized controlled trials, Cohort studies

## Abstract

This meta-research study aims to evaluate the agreement of effect estimates between bodies of evidence (BoE) from RCTs and cohort studies included in the same nutrition evidence synthesis, to identify factors associated with disagreement, and to replicate the findings of a previous study. We searched Medline, Epistemonikos and the Cochrane Database of Systematic Reviews for nutrition systematic reviews that included both RCTs and cohort studies for the same patient-relevant outcome or intermediate-disease marker. We rated similarity of PI/ECO (population, intervention/exposure, comparison, outcome) between BoE from RCTs and cohort studies. Agreement of effect estimates across BoE was analysed by pooling ratio of risk ratios (RRR) for binary outcomes and difference of standardised mean differences (DSMD) for continuous outcomes. We performed subgroup and sensitivity analyses to explore determinants associated with disagreements. We included 82 BoE-pairs from 51 systematic reviews. For binary outcomes, the RRR was 1.04 (95% confidence interval (CI) 0.99 to 1.10, I^2^ = 59%, τ^2^ = 0.02, prediction interval (PI) 0.77 to 1.41). For continuous outcomes, the pooled DSMD was  − 0.09 (95% CI  − 0.26 to 0.09, PI  − 0.55 to 0.38). Subgroup analyses yielded that differences in type of intake/exposure were drivers towards disagreement. We replicated the findings of a previous study, where on average RCTs and cohort studies had similar effect estimates. Disagreement and wide prediction intervals were mainly driven by PI/ECO-dissimilarities. More research is needed to explore other potentially influencing factors (e.g. risk of bias) on the disagreement between effect estimates of both BoE.

*Trial registration*: CRD42021278908

**Supplementary Information:**

The online version contains supplementary material available at 10.1007/s10654-023-01058-5.

## Introduction

Bodies of evidence (BoE) from randomised controlled trials (RCTs) and cohort studies provide valuable insights into relations between dietary intervention or exposures (e.g. foods, micronutrients or dietary patterns) and health outcomes (e.g. event rates or intermediate disease markers) [1–4], and inform dietary guidelines and health reports [5–7].

Cohort studies are the most common evidence sources in nutrition research and outnumber the evidence from RCTs [8]. This is subject of an ongoing debate in nutritional epidemiology since observational studies are considered to provide less trustworthy findings [9, 10]. They are prone to risk of bias due to confounding and measurement error [10–12]. RCTs, in contrast, are the gold standard to assess benefits and harms of interventions, and for drawing causal inference [13]. If well conducted, randomisation provides – by chance – two or more study arms that are balanced for all prognostic factors and effect modifiers [14, 15]. However, RCTs are challenging in nutritional research [10, 16] and their conducting is not feasible for all research questions for ethical reasons [16]. RCTs are also considered to lack external validity as study participants may not be representative of the population to which study results are applied [14]. Cohort studies may complement evidence from RCTs, and enlarge the available BoE when evidence from RCTs is scare or indirect [17].

Previous meta-epidemiological studies have investigated the agreement of effect estimates from RCTs and observational studies in medical research and observed a high degree of concordance [18–20]. The recent study by our group [21] was the first that focused exclusively on diet-disease relations in the field of nutrition research. Although in the past, several dietary RCTs have failed to confirm associations between dietary exposures and risk of chronic diseases found in large cohort studies [22–26], we observed that on average RCTs and cohort studies had similar effect estimates [21]. As in other research fields, replication of studies in the field of nutrition and health is crucial, to validate earlier findings or explore transferability to closer or broader research questions [27, 28]. In our previous study [21], we matched BoE from Cochrane reviews of RCTs with BoE from systematic reviews of cohort studies. Our matching approach, however, has the limitation that comparability between BoE-pairs might be impaired due to differing methodological approaches, such as search strategies, eligibility criteria, study selection, and bias assessment.

Thus, this meta-research study aimed to replicate our previous findings [21] and created a new sample where only BoE-pairs from RCTs and cohort studies included in the same evidence synthesis were considered.

The findings of our study will contribute to a better understanding for the possible integration of both study designs in future nutrition evidence syntheses, re-evaluate and validate important determinants explaining disagreement between BoE from RCTs and cohort studies.

## Materials and methods

We conducted a meta-research study, adhering the PRISMA 2020 statement for reporting systematic reviews [29] and guidelines for meta-epidemiological research [30]. A protocol was prospectively registered in PROSPERO (CRD42021278908). This study is a replication and changes made to the original study [21] are shown in Appendix S1 (Online Resource).

Eligibility criteria are described in Table 1. Briefly, we included nutrition systematic reviews that included both RCTs and cohort studies for a similar dietary exposure and patient-relevant outcome or intermediate disease marker, and that performed meta-analyses for at least one BoE. We defined BoE as all studies of a specific study design (RCTs or cohort studies) in a systematic review that provide evidence on a particular PI/ECO (population, intervention/exposure, comparison, outcome) question.

**Table 1:** Description of eligibility criteria

**Population**	**General population**
**Intervention/Exposure** (dietary intake, supplementation or biomarkers of dietary intake)	**Dietary pattern:** e.g. Mediterranean diet, vegetarian diet, carbohydrate-restricted diet. OR **Food groups:** food groups (macro-level), and foods (micro-level) are considered: e.g. grains, vegetables, fruit, milk and dairy products, meat, fish, eggs, nuts, chocolate, oil. OR **Macronutrients:** *carbohydrates*, e.g. starch, fructose, glucose, sucrose; *fat*, e.g. omega–3 fatty acids (EPA, DHA, α-linolenic acid); omega–6 fatty acids (linoleic acid), monounsaturated fat; *proteins*, e.g. amino acids. OR **Micronutrients:** *vitamins*, e.g. β-carotene, vitamins A, E, C (ascorbic acid), and D (cholecalciferol, ergocalciferol); B vitamins (thiamine, riboflavin, niacin, pyridoxine, cobalamin, folic acid); *minerals*, e.g. calcium, magnesium, selenium, sodium, potassium, iron, zinc, copper, iodine. OR **Other:** fibre (psyllium, inulin, cellulose); probiotics; prebiotics; synbiotics
**Control / Comparison**	**Low/ no intake, supplementation or status level** of the above mentioned interventions/exposure. OR **Placebo.** OR **Usual care**
**Outcome**	**Patient-relevant outcomes:** e.g. mortality, cancer, type 2 diabetes, dementia, age-related macular degeneration, *coronary heart disease*, e.g. myocardial infarction, ischemic heart disease, and acute coronary syndrome; *stroke*, e.g. ischemic or haemorrhagic **Intermediate disease markers**: e.g. systolic and diastolic blood pressure, fasting glucose, LDL-cholesterol; body weight
**Study design**	**Systematic reviews with meta-analysis**, including both designs: **Randomised controlled trials**: parallel, crossover, factorial, cluster design **Cohort studies:** nested case–control, case-cohort studies, long-term prospective cohort studies. Retrospective and cross-sectional studies are excluded

*DHA* Docosahexaenoic acid; *EPA* Eicosapentaenoic acid; *LDL* Low-density lipoprotein

### Literature search

We searched MEDLINE (via OVID), the Cochrane Database of Systematic Reviews and Epistemonikos for systematic reviews published in the period between 01.01.2011 to 06.09.2021. This cut-off was chosen to cover a 10-year period in line with a recent meta-epidemiological study in nutrition research [21]. The search strategy is presented in Appendix S2 (Online Resource). Two reviewers independently (IR, JE, JS or LS) screened titles and abstracts as well as potentially relevant full texts. Any discrepancy was resolved by discussion or by consulting a third reviewer (JS or LS).

For each eligible systematic review we included a maximum of three patient-relevant outcomes (e.g. cardiovascular disease) and a maximum of three intermediate disease markers (e.g. systolic blood pressure). We excluded highly correlated outcomes from our sample (e.g. cardiovascular disease and coronary heart disease) (Online Resource Table S1). If more than three outcomes were available for a given systematic review, we included the primary outcomes and thereafter we used a top down approach (highest number of studies included in BoE from RCTs; highest number of study participants; highest number of cases).

When two or more identified systematic reviews investigated the same PI/ECO, we included the BoE-pair with more studies (or more study participants) (Online Resource Table S2).

### Data extraction

For each included BoE, we extracted information on the study characteristics of the primary studies forming this BoE. These data included the description of study population (e.g. age, disease status), intervention or exposure (e.g. dietary pattern), comparator (e.g. low intake), and outcome (e.g. all-cause mortality), as well the duration and follow-up of the intervention or exposure, and the study design (e.g. parallel or factorial for RCTs). Moreover, we extracted for each BoE the number of included studies, number of participants, number of events, type of comparison (e.g. high vs. low intake), effect estimates, type of effect measure (risk ratio [RR], odds ratio [OR], hazard ratio, mean difference or standardised mean difference), 95% confidence interval (CI), and measure of heterogeneity (τ^2^ or I^2^). Data extraction was performed by one reviewer (JB, IR, LH, JE, or JS) and checked by at least one second reviewer (JB, JS). Discrepancies were discussed with a third reviewer (LS).

### Recalculation and conversion of effect estimates

Where necessary, we recalculated meta-analyses and/or converted effect estimates: If in a systematic review a meta-analysis was not available for one study type (e.g. cohort studies) but relevant data were available, we pooled the respective primary studies. If the summary effect estimate was based on a pool of studies of different designs (e.g. trials including RCTs and quasi-randomised controlled trials, or observational studies including case–control studies, or retrospective cohort studies), we recalculated the summary effect estimates by excluding non-randomised controlled trials and non-cohort studies, while retaining the studies fulfilling our eligibility criteria. In cases where effect estimates were reported without subgroup analysis by study design type, we separated the studies and performed meta-analyses for BoE from RCTs and cohort studies, respectively. Also, if pooled effect estimates were only available for variable subtypes of one BoE (for cohort studies, e.g. nested case–control studies, clinical cohorts), we pooled them in a meta-analysis to obtain a summary effect estimate for the respective BoE.

To improve comparability between interventions in RCTs and exposures in cohort studies, we recalculated (whenever feasible) effect estimates when a BoE reported summary effect estimates based on different types of dietary measure (e.g. dietary intake, dietary supplements, nutrient status). For example, if a meta-analysis of RCTs investigated the effect of selenium supplements, and a meta-analysis of cohort studies combined plasma selenium status with selenium supplements, we excluded the studies with plasma selenium status and recalculated the summary effect estimates only based on the studies with selenium supplements.

When the dose between BoE from RCTs and cohort studies differed, we attempted to convert effect estimates between RCTs and cohort studies to standardised doses. The dose used in BoE of cohort studies served as reference. For example, if the dose of folic acid in BoE of RCTs was 0.8mg/day (RR 0.42, 95% CI 0.19 to 0.98) and in BoE of cohort studies 0.6mg/day, we recalculated the RR and 95% CI in BoE of RCTs for 0.6mg/day (RR 0.58, 95% CI 0.33 to 1.02).

We converted summary effect estimates if BoE from RCTs and BoE from cohort studies investigated opposite comparisons (e.g. low vs. high sodium intake in RCTs and high vs. low intake in cohort studies). Moreover, in line with our previous study [19] we standardised the direction of effect of the outcomes so that summary effect estimates < 1 are always expressing a beneficial effect.

If the summary effect measure for binary or continuous outcomes was not the same for BoE from RCTs and BoE from cohort studies, we used the appropriate conversion formulas in order to have the two estimates expressed in the same measure. For binary outcomes, we used risk ratios (RR). Odds ratio (OR) was transformed into RR using an assumed control risk (ACR); $\mathrm{RR}=\frac{\mathrm{OR}}{1-\mathrm{ACR x }(1-\mathrm{OR})}$) [31, 32]. For hazard ratios, we went back to the primary studies of the respective BoE and extracted the relevant data (number of participants and events in intervention and control group) to calculate a RR. For continuous outcomes, we computed mean differences (MD) for outcomes measured on the same scale (e.g. body weight in kg) and standardised mean differences (SMD) to pool intermediate disease markers with different outcomes scales.

### Evaluating similarity between BoE from RCTs and cohort studies

Similarity between each BoE-pair was rated using the PI/ECO similarity criteria as described previously [19] (Online Resource Appendix S3). Similarity of each PI/ECO domain was rated as "more or less identical", "similar but not identical", or "broadly similar". The overall similarity of each BoE-pair was determined by the domain with the lowest degree of similarity. For instance, if the domain "population" was rated as "broadly similar", the overall similarity of this BoE-pair was also rated as "broadly similar".

Two reviewers (JB, JS) independently assessed the PI/ECO similarity between each BoE-pair. Discrepancies were resolved through discussion with a third reviewer (LS).

### Statistical analysis

We assessed concordance between results from eligible BoE from RCTs or cohort studies, using a structured approach [33]. We defined effect estimates of the BoE from RCTs and cohort studies as concordant, if one of the following conditions are met: (1) Both effect estimates suggest the same direction (e.g. both effect estimates suggesting lower risk of disease) and are statistically significant (p-value ≤ 0.05). (2) Both effect estimates are not statistically significant, and within the range of 0.8 to 1.25 [34] of a 95% CI (for binary outcomes) or the minimal important difference (for continuous outcomes). Thresholds for minimal important differences are listed in Online Resource Table S3.

To quantify differences of effect estimates we computed a ratio of risk ratios (RRR) [35] for each BoE-pair with binary outcome and a difference of mean difference (DMD) or standardised mean differences (DSMD) for continuous outcomes. BoE from cohort studies served as the reference group. To assess whether in total effect estimates of BoE from RCTs are larger or smaller in relation to those of BoE from cohort studies, we pooled the summary effect estimates (RRR, DMD or DSMD) using a random-effects model [36]. Statistical heterogeneity of effect estimates was assessed with the τ^2^ or I^2^ statistics [36, 37]. To estimate τ^2^ we used the Paule and Mandel method [38, 39]. We computed 95% prediction intervals (PI) to provide the range of possible parameters for the differences between results of BoE from RCTs and BoE from cohort studies likely to occur in future studies comparing the two sources. Meta-analyses were performed with the R package meta (version 4.2.1) [40].

### Subgroup and sensitivity analyses

We conducted subgroup analyses to explore determinants that are potentially related to disagreement of effect estimates. Therefore, we formed subgroups with respect to the different types of intervention/exposure (e.g. dietary pattern, food groups, macronutrients), type of intake (e.g. dietary intake, supplementation, status), and type of outcome (e.g. all-cause mortality, cardiovascular disease, pregnancy outcomes). Moreover, we performed subgroup analysis based on the degree of PI/ECO similarity (overall, and for each domain separately) and the methodological quality of the review (using AMSTAR 2 [41]).

We assessed the robustness of our findings with three sensitivity analyses. First, by including only one BoE-pair from each systematic review – the one with the highest number of RCTs (or if the number of RCTs was equal we primarily included the BoE with the highest number of participants, followed by the highest number of events, and the highest number of cohort studies). Second and third, we performed sensitivity analyses by direction of cohort study summary effect estimate with RR < 1 and RR ≥ 1, respectively.

In a post-hoc analysis, we performed subgroup analyses for type of micronutrients (vitamin D vs. other micronutrients) and type of cancer. Moreover, we accounted for overlaps between the current sample and the previous sample [21] and performed sensitivity analyses by excluding BoE-pairs with highly similar PI/ECO questions and overlapping primary studies.

## Results

**Fig. 1:**
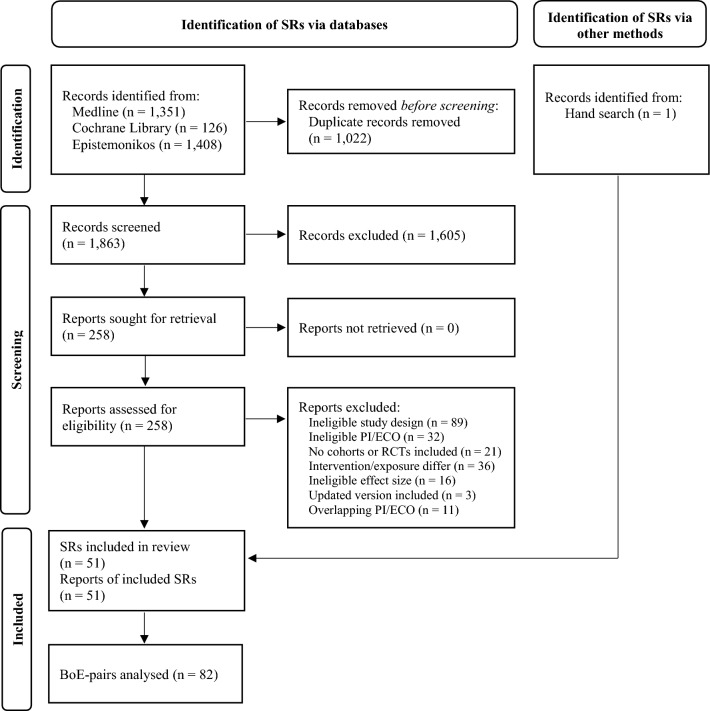
PRISMA flow diagram of the study search and selection process [29] *BoE* Body of evidence; *PI/ECO* Population, intervention/exposure, comparator, outcome; *RCT* Randomised controlled trial; *SR* Systematic review

The literature search identified 2885 records. After removing duplicates with the Systematic Review Accelerator Deduplicator (https://sr-accelerator.com/#/deduplicator) 1863 records remained for screening. Among these, 258 reports were assessed for eligibility in full text screening. We listed any excluded record with its reason for exclusion in Appendix S4 (Online Resource). Finally, we included 51 systematic reviews in this study (Fig. 1) [6, 42–91].

After exclusion of highly correlating outcomes (Online Resource Table S1), a final sample of 82 BoE-pairs from RCTs and cohort studies was analysed (Online Resource Table S4).

### Descriptive characteristics

The number of studies in BoE from RCTs ranged from 1 to 27 (median 3, interquartile range [IQR] 1 to 6) and in BoE from cohort studies from 1 to 68 (median 4, IQR 2 to 8). The range of participants was 201 to 152,848 (median: 4862, IQR: 1565 to 24,947.5) in BoE from RCTs, and 302 to 1,926,520 (median 119,269, IQR 13,637 to 239,862) in BoE from cohort studies.

Out of 82, we performed re-analyses of 49 BoE-pairs from 29 systematic reviews [6, 42–44, 46, 47, 50, 52, 54, 60–62, 65, 71, 72, 76–83, 85, 86, 88–91]. For three BoE-pairs, effect estimates were not convertible (body weight in Miller et al. [67], Mini Mental State Examination Score in Setien-Suero et al. [75] and fasting glucose in Zhang et al. [88]) and thus were not analysed. Detailed descriptions of all transformation made are reported in the supplement (Online Resource Table S5).

The following intervention categories were identified: micronutrients (n = 51, 62.2%), dietary pattern (n = 13, 15.8%), food groups (n = 8, 9.8%), macronutrients (n = 6, 7.3%) and others (n = 4, 4.9%). The outcomes of the BoE-pairs were categorised as follows: cancer (n = 22, 26.8%), cardiovascular disease (n = 17, 20.7%), pregnancy (n = 13, 15.9%), intermediate disease markers (n = 12, 14.6%), endocrine/metabolic (n = 8, 9.8%), eye disease (n = 5, 6.1%), and others (n = 5, 6.1%). With regard to the type of intake/exposure, 24 (29.3%) BoE-pairs compared intake vs. intake, 23 (28.1%) supplementation vs. supplementation, 12 (14.6%) supplementation vs. intake, 12 (14.6%) supplementation vs. status, and 11 (13.4%) others.

Study characteristics for each BoE including detailed descriptions of PI/ECO are depicted in the Online Resource (Tables S6 and S7).

Of the 51 included systematic reviews, 44 (86.3%) were of critically low, five (9.8%) of low, and two (3.9%) of moderate methodological quality according to the AMSTAR 2 tool (Online Resource Table S8).

### PI/ECO similarity degree

Of the 82 included BoE-pairs, ten (12.2%) pairs were rated overall as "more or less identical", 57 (69.5%) as "similar but not identical" and 15 (18.3%) as "broadly similar" (Online Resource Table S9). The rating "broadly similar" was mainly attributable to differences in interventions and comparators (n = 12). In these BoE-pairs [44, 46, 50, 52, 75, 80, 85, 87, 88, 90], supplementation of micronutrients (e.g. dose: 2000–4000IU/day of vitamin D vs. 0-400IU/day) in BoE from RCTs was compared to biomarkers of micronutrient status (e.g. 25-hydroxy vitamin D level in blood ≥ 28nmol/l vs. < 28nmol/l) in BoE from cohort studies. Overall, we rated three BoE-pairs as "broadly similar" due to differences in study population [55, 71], e.g. populations at high risk (e.g. in RCTs) were compared to general healthy population (e.g. cohort studies). In Filippini et al. [55], for instance, the BoE from RCTs focused on participants with precancerous lesions of the prostate, whereas the BoE from cohort studies focused on a general healthy population without history of prostate cancer.

### Statistical heterogeneity of included individual comparisons

Across individual meta-analyses of RCTs, the median τ^2^ was 0.015 (I^2^ = 32.8%) for binary outcomes (measured as RRs) and τ^2^ = 0.01 (I^2^ = 23%) for continuous outcomes (measured as SMDs), and τ^2^ = 0.01 (I^2^ = 47.5%) and τ^2^ = 0.02 (I^2^ = 41%) for cohort studies, respectively.

When stratified by overall PI/ECO similarity degree, the median τ^2^ across meta-analyses with binary outcomes showed higher statistical heterogeneity for BoE-pairs with a "broadly similar" rating: τ^2^ = 0.08 (I^2^ = 40.4%) for meta-analyses of RCTs and τ^2^ = 0.05 (I^2^ = 60%) for meta-analyses of cohort studies. For BoE-pairs with a "similar but not identical" rating, the heterogeneity was τ^2^ = 0.015 (I^2^ = 19.5%) and τ^2^ = 0.01 (I^2^ = 38%) for meta-analyses of RCTs and of cohort studies, respectively. For BoE-pairs with a "more or less identical" rating, the heterogeneity was τ^2^ = 0.01 (I^2^ = 0%) and τ^2^ = 0.02 (I^2^ = 67%) for meta-analyses of RCTs and of cohort studies, respectively.

### Meta-epidemiological analysis

Using the structured approach to assess concordance, 15 (19.0%) out of 79 analysed diet-disease outcome pairs were concordant. Proportion of concordance was similar for binary (12/66) and continuous (3/13) outcomes, respectively (Online Resource Table S10).

**Fig. 2:**
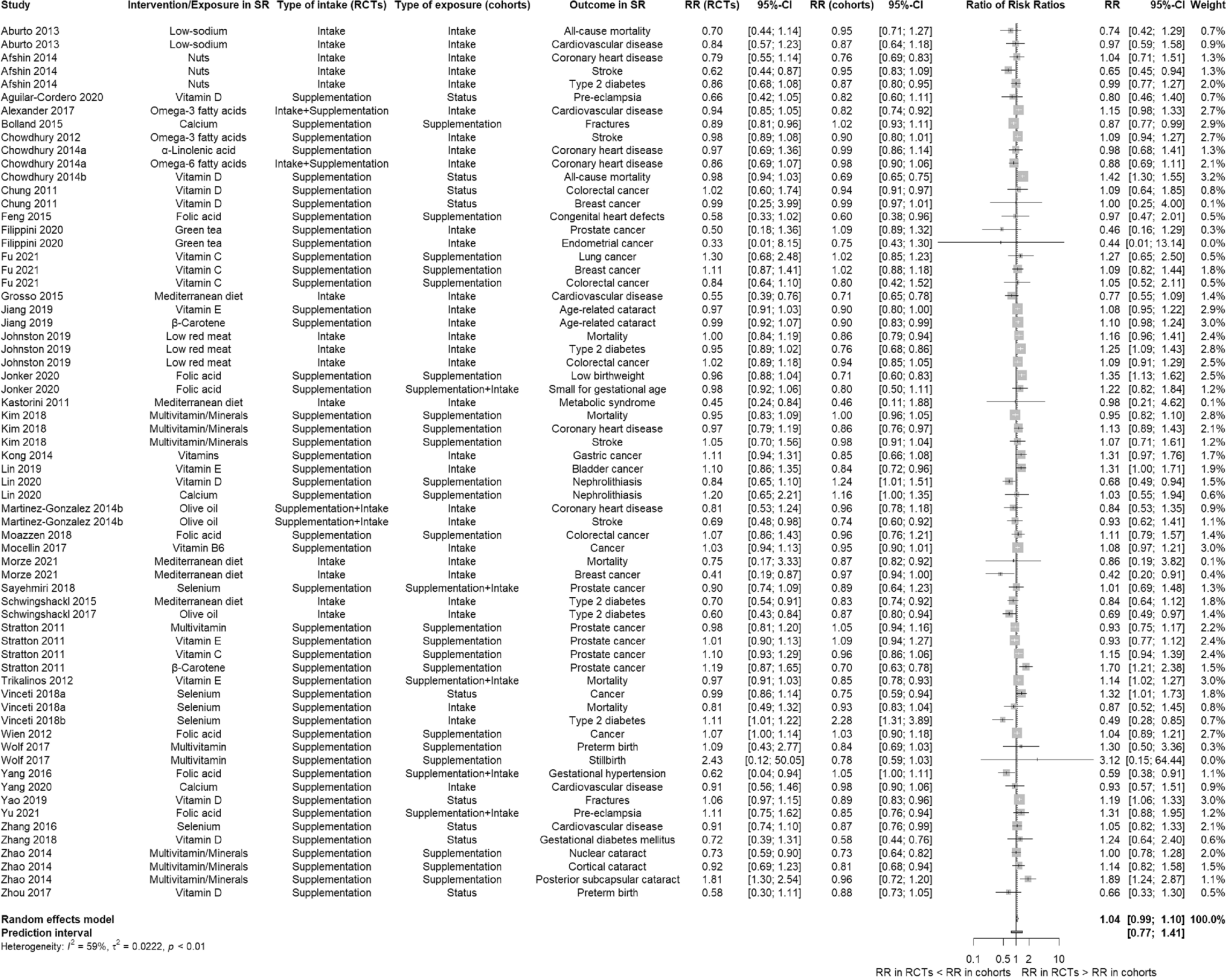
Forest plot of the overall comparison between bodies of evidence from randomised controlled trials versus those from cohort studies for binary outcomes using pooled ratio of risk ratios *CI* Confidence interval; *RCT* Randomised controlled trial; *RR* Risk ratio; *RRR* Ratio of risk ratios; *SR* Systematic review

We performed an analysis for 66 BoE-pairs with binary outcomes and 13 for continuous outcomes (among these 13 pairs with MD and 6 with SMD). On average, the BoE from RCTs had similar estimates compared to the BoE from cohort studies: For binary outcomes, the pooled effect estimate across BoE-pairs was RRR 1.04 (95% CI 0.99 to 1.10, PI 0.77 to 1.41; Fig. 2). The statistical heterogeneity was moderate (I^2^ = 59%, τ^2^ = 0.02). With regard to the included effect estimates (RRR) in each BoE-pair, 39.4% were within 0.9 and 1.1, 27.3% < 0.9 and 33.3% > 1.1.

**Fig. 3:**
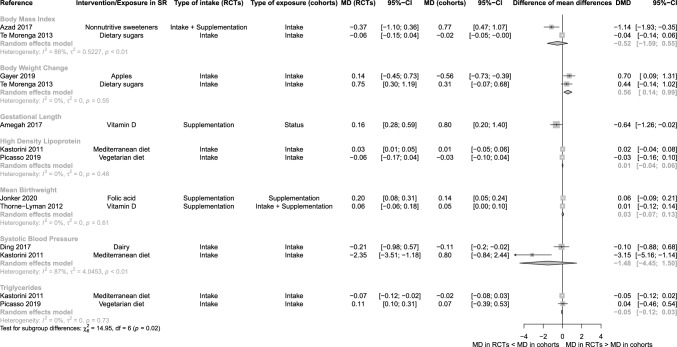
Forest plot of the comparison between bodies of evidence from randomised controlled trials versus those from cohort studies for continuous outcomes using difference of mean difference. *CI* Confidence interval; *DMD* Difference of mean differences; *MD* Mean difference; *RCT* Randomised controlled trial; *SR* Systematic review

For continuous outcome pairs, the pooled DSMD was − 0.09 (95% CI − 0.26 to 0.09, PI − 0.55 to 0.38; Online Resource Figure S1). We observed no differences in the MDs between BoE from RCTs and cohort studies for various intermediate disease markers, except for slight disagreement in body weight change (MD 0.56 (95% CI 0.14 to 0.99; Fig. 3).

### Subgroup analysis

Results of subgroup analysis are depicted in Table 2. When stratified by dietary intervention/exposure, we observed no disagreement across BoE from RCTs and cohort studies for the subgroups dietary pattern, food group, macronutrients, and green tea. Effect estimates for micronutrient comparisons, however, were slightly different (RRR 1.08, 95% CI 1.02 to 1.15, I^2^ = 62, τ^2^ = 0.02, PI 0.81 to 1.45; Online Resource Figure S2).

**Table 2:** Overview of main results for binary outcomes

	BoE-pairs included	Ratio of risk ratios (95% CI)	Heterogeneity (I^2^ (%); τ^2^)	95% prediction interval
Main analysis	66	1.04 (0.99 to 1.10)	59; 0.02	0.77 to 1.41
*Stratified by type of dietary intervention/exposure*				
Micronutrients	46	1.08 (1.02 to 1.15)	62; 0.02	0.81 to 1.45
Dietary pattern	8	0.99 (0.81 to 1.20)	59; 0.04	0.57 to 1.70
Foods group	6	0.86 (0.73 to 1.00)	18; 0.01	0.63 to 1.17
Macronutrients	4	1.06 (0.94 to 1.18)	21; 0.003	0.75 to 1.48
Green tea	2	0.46 (0.17 to 1.23)	0; 0	N/A
*Stratified by type of intake/exposure*				
Supplementation vs. Supplementation	22	1.07 (0.98 to 1.16)	53; 0.02	0.79 to 1.44
Intake vs. Intake	14	0.93 (0.81 to 1.06)	60; 0.03	0.62 to 1.39
Supplementation vs. Intake	12	1.05 (0.92 to 1.19)	31; 0.03	0.71 to 1.54
Supplementation vs. Status	9	1.20 (1.06 to 1.36)	53; 0.01	0.90 to 1.60
Supplementation vs. Intake+Supplementation	5	1.04 (0.80 to 1.34)	58; 0.06	0.44 to 2.47
Intake+Supplementation vs. Intake	4	1.01 (0.87 to 1.17)	35; 0.01	0.63 to 1.60
*Stratified by type of outcome*				
Cancer	22	1.09 (1.01 to 1.18)	23; 0.01	0.88 to 1.36
Cardiovascular disease	17	1.03 (0.97 to 1.10)	22; 0.003	0.90 to 1.19
Pregnancy outcomes	10	1.05 (0.85 to 1.29)	49; 0.04	0.63 to 1.75
Endocrine / metabolic disease	8	0.86 (0.70 to 1.06)	75; 0.05	0.47 to 1.57
Eye disease	5	1.15 (0.96 to 1.37)	43; 0.03	0.63 to 2.09
Fractures	2	1.02 (0.75 to 1.38)	93; 0.04	N/A
All-cause mortality	2	1.09 (0.58 to 2.04)	81; 0.17	N/A
*Stratified by overall PI/ECO similarity degree*				
More or less identical	8	1.01 (0.87 to 1.18)	60; 0.02	0.67 to 1.52
Similar but not identical	48	1.03 (0.97 to 1.10)	48; 0.02	0.76 to 1.40
Broadly similar	10	1.15 (0.99 to 1.34)	57; 0.02	0.78 to 1.69
*Stratified by AMSTAR 2 rating*				
Moderate	4	0.98 (0.65 to 1.49)	45; 0.07	0.23 to 4.19
Low	8	1.06 (0.91 to 1.24)	54; 0.03	0.66 to 1.72
Critically low	54	1.03 (0.97 to 1.09)	60; 0.02	0.77 to 1.37

*AMSTAR2* A measurement tool to assess systematic reviews, version 2; *BoE* Body of evidence; *CI* Confidence interval; *N/A* Not applicable; *PI/ECO* Population, intervention or exposure, comparator, outcome

Subgroup analyses by type of dietary exposure showed substantial disagreement in the comparison between supplementation vs. status (RRR 1.20, 95% CI 1.06 to 1.36, I^2^ = 53%, τ^2^ = 0.01, PI 0.90 to 1.60; whereas no differences for all other types were observed (Online Resource Figure S3).

Analysis by outcome type showed mainly no differences (Online Resource Figure S4). Best agreement of effect estimates was observed for the subgroups cardiovascular disease (RRR 1.03, 95% CI 0.97 to 1.10, I^2^ = 22%, τ^2^ = 0.003, PI 0.90 to 1.19) and pregnancy outcomes (RRR 1.05, 95% CI 0.85 to 1.29, I^2^ = 49%, τ^2^ = 0.04, PI 0.63 to 1.75).

The stratified analysis by overall PI/ECO similarity revealed that for “broadly similar” BoE-pairs, we observed some degree of disagreement and high statistical heterogeneity (RRR 1.15, 95% CI 0.99 to 1.34, I^2^ = 57%, τ^2^ = 0.02, PI 0.78 to 1.69; Online Resource Figure S5). In subgroup analyses with stratification for each PI/ECO domain (Online Resource Table S9, Figures S6 to S9), we observed that dissimilarities between intervention and exposure, i.e. supplementation vs. status, explained most of the differences (RRR 1.20, 95% CI 1.06 to 1.36, I^2^ = 53%, τ^2^ = 0.01, PI 0.90 to 1.60).

Subgroup analysis with stratification by AMSTAR 2 rating revealed on average no disagreement between effect estimates across BoE from RCTs and cohort studies (Online Resource Figure S10).

### Sensitivity analysis

The sensitivity analysis where only one outcome (i.e. with the largest number of RCTs) was chosen from each systematic review confirmed the findings from the main analysis (RRR 1.01, 95% CI 0.94 to 1.09, I^2^ = 69%, τ^2^ = 0.03, PI 0.69 to 1.48, n = 42) (Online Resource Figure S11).

Sensitivity analyses by direction of effect yielded a RRR of 1.10 (95% CI 1.04 to 1.15, I^2^ = 48%, τ^2^ = 0.01, PI 0.86 to 1.39, n = 54), and 0.88 (95% CI 0.77 to 1.01, I^2^ = 45%, τ^2^ = 0.03, PI 0.58 to 1.33, n = 12) for BoE-pairs where the RR of the BoE from cohort studies was < 1 and ≥ 1 respectively (Online Resource Figure S12 and S13).

In post-hoc analyses, we did not observe differences between effect estimates of BoE from RCTs and BoE from cohort studies for vitamin D (RRR 1.04, 95% CI 0.85 to 1.29, I^2^ = 75%, τ^2^ = 0.04, PI 0.58 to 1.86), however effect estimates were slightly dissimilar in the group of non vitamin D micronutrients (RRR 1.08, 95% CI 1.01 to 1.15, I^2^ = 45%, τ^2^ = 0.02, PI 0.83 to 1.40; Online Resource Figure S14). The stratified analyses by cancer type also revealed on average no disagreement between effect estimates of BoE-pairs of colorectal cancer, breast cancer and prostate cancer respectively (Online Resource Figure S15).

Compared to the sample used in the previous study, we identified an overlap in PI/ECO questions and primary studies in 18 BoE-pairs (out of 66; 27.3%) with binary outcomes (Online Resource Table S12). Excluding these overlapping BoE-pairs did not impact the findings of the main analysis (RRR 1.03, 95% CI 0.96 to 1.11, I^2^ = 53%, τ^2^ = 0.03, PI 0.73 to 1.46; Online Resource Figure S16).

We did not perform subgroup and sensitivity analyses for continuous outcomes since the number of eligible BoE-pairs was small.

## Discussion

### Summary of findings

We performed a large meta-research replication study evaluating the agreement of effect estimates between BoE from RCTs and cohort studies included in the same nutrition evidence synthesis. Overall, we identified 82 BoE-pairs from 51 systematic reviews. Dietary interventions/exposures focused mainly on micronutrients (n = 51, 62.2%). With regard to the PI/ECO similarity degree, ten BoE-pairs (12.2%) were rated as "more or less identical", 57 (69.5%) as "similar but not identical" and 15 (18.3%) as "broadly similar". The majority of the included systematic reviews (n = 44, 86.3%) were of critically low methodological quality according to the AMSTAR 2 tool. Of the 66 binary and 13 continuous outcome BoE-pairs included in the analysis, 19% were concordant.

We successfully replicated the findings of our previous study [21], where on average RCTs and cohort studies had similar effect estimates: For binary outcomes, the pooled RRR was 1.04 (95% CI 0.99 to 1.10, PI 0.77 to 1.41), and for continuous outcome pairs, the pooled DSMD was − 0.09 (95% CI − 0.26 to 0.09, PI − 0.55 to 0.38). However, the wide prediction intervals suggest that differences could be considerably larger or smaller in either direction. Subgroup analyses revealed that disagreement was driven by PI/ECO dissimilarity, in particular the comparisons of dietary supplements in RCTs and nutrient status in cohort studies, explained most of the differences. Statistical heterogeneity was highest and prediction intervals were wider in BoE-pairs with the most dissimilar PI/ECO.

### Comparison with other studies

Our meta-research study is in line with previous studies in the medical field: Bröckelmann et al. [19] evaluated the agreement between BoE from RCTs and cohort studies for various medical research questions by considering also only BoE included in the same evidence synthesis. Based on 129 BoE-pairs, they revealed a summary effect of 1.04 (95% CI 0.97 to 1.11), which is highly concordant with our main finding (RRR 1.04, 95% CI 0.99 to 1.10). The Cochrane review by Anglemyer et al. [18] revealed also similar effect estimates (RRR 1.04, 95% CI 0.89 to 1.21), by considering RCTs and cohort studies in a subgroup analysis of nine methodological reviews.

With regard to our previous study in nutrition research [21], some nuanced differences between both studies findings were observed. First, in the replication study, the agreement between RCTs and cohort studies was slightly higher (RRR 1.04, 95% CI 0.99 to 1.10 vs. RRR 1.09, 95% CI 1.04 to 1.14), which provides support for our main hypothesis, that RCTs and cohort studies on average show similar results. In line with previous studies [19, 21], we also showed in subgroup analyses, that dissimilarities were driven by PI/ECO characteristics, and occurred especially in "broadly similar" BoE-pairs. Second, in our sample, heterogeneity and prediction intervals were slightly smaller (I^2^ = 59%, τ^2^ = 0.02 and 95% PI 0.78 to 1.41 vs. I^2^ = 68%, τ^2^ = 0.02, and 95% PI 0.81 to 1.46 [21]). This might be, since we considered only BoE-pairs of the same systematic review, whereas in our previous study we matched BoE from Cochrane reviews of RCTs with corresponding BoE from systematic review of cohort studies. Third, our eligibility criteria for BoE-pairs were slightly different: we accounted for possible overlap between systematic reviews and excluded correlating outcomes already in the main analysis.

### Dissimilarities between BoE-pairs

RCTs and cohort studies may often differ regarding study population and intervention/exposure, as shown in our sample. The most frequent observed dissimilarity was the difference in type of intake/exposure, for example when comparing vitamin D supplementation in RCTs to plasma vitamin D status in cohort studies [90]. In these comparisons, disagreement may also result from differences in study population: In RCTs, participants might already have an adequate vitamin D supply at baseline (e.g. due to inclusion criteria), while in cohort studies wider ranges of vitamin D status can be observed [44, 46, 50, 90]. Dissimilarities may also arise from differences in administered doses in interventions or exposure. As an example, for the risk of lung cancer vitamin C supplementation of 500mg/day vs. placebo in BoE of RCTs was compared to any (> 120.2mg/day) vs. no supplementation in BoE from cohort studies [56]. The type of intervention administration and exposure assessment may also influence effect estimates. In BoE-pairs on dietary pattern, participants randomised to a dietary pattern were compared to participants of cohorts studies who adhered to this dietary pattern according to a food-frequency questionnaire at baseline or designated time point(s) [58].

With regard to the population, we observed that in ‘similar but not identical’ and ‘broadly similar’ BoE-pairs populations at risk or with a specific disease condition in BoE from RCTs were frequently compared to general healthy populations in BoE from cohort studies. In the analysis of green tea on the risk of prostate cancer, for instance, population at with precancerous lesions in RCTs were compared to a general healthy population without prostate cancer in cohort studies [55]. This may cause differences in effect estimates since prognostic factors are not equally distributed between the two study design types.

Finally, our sample also provides examples, where research questions were closely similar: In Lin et al. 2020, for instance, both BoE investigated the impact of calcium supplementation on risk of nephrolithiasis in general population [65]. Moreover, the impact of vitamin E supplementation in mid-aged general male population on risk of prostate cancer was evaluated in both BoE in Stratton et al. 2011 [76].

On an individual comparison level the most two discordant comparisons in either direction were: Mediterranean diet and breast cancer (RR 0.42, 95% CI 0.20 to 0.91) [70] and multivitamin/mineral supplementation and posterior subcapsular cataract (RR 1.89, 95% CI 1.24 to 2.87) [89].

In the first comparison [70], disagreement may be due to differences in population. BoE from RCTs based on women at high risk of cardiovascular disease, with a mean age of 68 (range 60–80 years) and a mean body mass index > 30. In contrast, BoE from cohort studies included younger general healthy populations (mean ages ranging between 35 and 61), which had a lower body mass index (mean ≤ 25 in 8/12 included cohorts). These population differences may lead to the different findings, as, for example, body fatness is classified a probable risk factor for breast cancer according to the World Cancer Research Fund [92]. Moreover, we observed smaller sample sizes (4,152 vs. 982,733), less cases (35 vs. 35,338) and shorter follow-up time (4.8 vs. 3–18 years) in BoE of RCTs, leading to more imprecise effect estimates (and wide CI) compared to cohort studies.

In the second comparison [89], we also detected major dissimilarities in the included population. In BoE from RCTs participants with and without early cataract were included, whereas BoE from cohort studies focused on a general healthy population with intact lens. Additionally, supplemented doses of multivitamins may differ between BoEs: in BoE from RCTs, participants received 1–2 capsules of combined multivitamins and minerals per day, whereas participants in the highest exposure groups in cohort studies indicate in their questionnaire that they have used multivitamins (and minerals) on a regularly base (e.g. for > 10 years).

### Potential implications

Cohort studies are a valuable evidence source in nutrition research to inform about diet-disease relations, by providing sequential and complementary information or replace findings from RCTs when these are not available [17, 93]. There are ongoing efforts to develop guidance for upcoming systematic reviews on when and how to integrate BoE from different study design types into their evidence syntheses and meta-analyses [94, 95].

Overall, agreement between effect estimates was highest when BoE from RCTs and cohort studies compared the same type of intake/exposure, however effect estimates were significantly different in broadly similar comparisons (supplementation vs. status). So, when future systematic review authors aim to include both RCTs and observational studies in meta-analyses, a careful evaluation of PI/ECO characteristics of each BoE-pair (and the included primary studies) is highly needed. Authors should also be encouraged to highlight differences observed across different BoE included and discuss their impact on the direction and magnitude of effect estimates.

Disagreement may also occur from bias and statistical heterogeneity on the individual study level. In our sample, we noticed that statistical heterogeneity was moderate or substantial for various individual meta-analyses of the same study design. These may be due to PI/ECO dissimilarities within a BoE. Chowdhury et al. [51], for example, included in their BoE from RCTs both participants with and without pre-existing chronic diseases. Therefore, performing a priori planned sensitivity and subgroup analyses based on PI/ECO criteria are crucial steps to explore sources of statistical heterogeneity.

The appropriateness of the available BoE from RCTs is considered as an important criteria when debating for or against the search and integration of non-randomised studies in evidence syntheses [96]. To generate trustworthy recommendations, it is recommended to rely on the evidence available that provides the highest certainty [95]. According to the GRADE approach, this is initially determined by study design; with BoE from RCTs staring at a high certainty, and BoE from observational studies at a low certainty rating [97]. A part from the study design per se, it is sensible to have a look at the risk of bias, imprecision, inconsistency, indirectness, and publication bias [95, 98]. A rigorous risk of bias assessment, for instance, informs about the credibility of the study results of the included primary studies. Bias may not only arise from design specifics, such as confounding in cohort studies or limitations like short duration or small sample size in RCTs, but also more generally from the duration of the study, the motivation and conscientiousness of its participants, the assessment of intervention/exposure, or the amount of missing data [9, 99, 100]. In our study, we observed wide prediction intervals, which could indicate that these potential factors cause bias in individual comparisons. Bias may affect effect estimates in each primary study, and consequently pooled effect estimates in BoE and (dis-)agreement of results across BoE.

Moreover, an evaluation of inconsistency may give valuable hints to potential sources of heterogeneity. Our analysis indicated that PI/ECO similarity was an important determinant for inconsistency, with high heterogeneity and wide prediction intervals in meta-analyses of dissimilar BoE-pairs. A prior pooling scenario showed, that the statistical inconsistency is mainly driven by the integrated observation studies, as these are more variable in their methodological procedures than the RCTs [101]. As a perspective, future meta-research should explore the risk of bias and certainty of evidence as potential source of disagreement and inconsistency.

High-quality evidence syntheses are important sources to provide a comprehensive and accurate summary of studies available for a research question at hand [41]. In our sample, however, we show that nutrition reviews were mainly of critically low rating according to the AMSTAR 2 tool. Future systematic review authors should thus be encouraged to pay attention to the reporting of important methodological aspects, especially with regard to the registration of a protocol and the risk of bias assessment.

### Strengths and limitations

We were able to perform a successful replication of our previous study, using a similar methodological approach and producing similar findings. Our meta-research study benefits from a large sample of 82 BoE-pairs from 51 systematic reviews, representing various dietary interventions/exposures. Besides, we registered a protocol of our study a priori on PROSPERO. We proceeded an extensive data extraction, including detailed description of the systematic review and the corresponding primary studies, and an assessment of the methodological quality with AMSTAR 2. This allowed us to perform a rigorous examination of differences in PI/ECO across the included BoE-pairs. Thus, we were also able to perform multiple a priori planned subgroup analyses to examine determinants potentially contributing to disagreement between effect estimates of RCTs and cohort studies. Moreover, we recalculated various effect estimates to ensure comparability between the BoE from both study design.

We acknowledge also several limitations: First, our sample covers only a period of 10 years due to our search strategy. Choosing another timeframe may yield more eligible BoE-pairs and different results. Second, the restriction to BoE-pairs included in the same systematic review may limit the representativeness of our sample. However, it also improves the comparability between BoE-pairs since methodological approaches for the identification, selection and data extraction and analysis of relevant primary studies may be similar in the same systematic reviews. Third, in 36 out of 82 BoE-pairs, only one RCT (n = 27) or one cohort study (n = 14) was included, which may have affected the statistical power to detect significant discordance. However, this may be mitigated by the fact that sample sizes in many of these studies were large (> 3500 participants) including a long-term follow-up (e.g. the PREDIMED study [102–104]). Fourth, even though we excluded overlapping studies and correlating outcomes a-priori, some degree of overlap cannot be ruled out. Primary studies may have contributed to more than one included BoE, which might have increased precision of our findings. However, the findings of our sensitivity analysis of including only one BoE-pair per systematic review confirmed those of the main analysis. Fifth, PI/ECO similarity was rated based on our previous study [21]. The criteria, however, were limited to the pre-selected characteristics in the guidance sheet. There might be additional determinants such as geographic location and ethnics, which may affect dietary pattern and intake, and thus lead to dissimilarities between BoE. Moreover, even tough criteria were predefined the rating may still be party subjective and limited in interrater reliability. To improve comparability, however, similarity rating was piloted with a sample of five studies, and performed independently by two reviewers. Sixth, the comparability between BoE-pairs was limited due to differences in doses in study intervention or exposures. In cohort studies, open exposure categories and missing information on median doses limited the comparability with RCTs. However, whenever possible we standardised doses between both study design types. Seventh, we observed moderate or substantial statistical heterogeneity in various individual meta-analyses of the same study design. Conducting meta-epidemiological study on meta-analysis may further increase heterogeneity. Finally, we did not evaluate the impact of risk of bias in the primary studies. In general, many included systematic reviews did not report on the assessment of risk of bias for both BoE or did not use state of the art methods in line with AMSTAR 2 item 9. Inadequate reporting was especially the case for the assessment of cohort studies (n = 47 BoE from cohort studies vs. n = 24 BoE from RCTs). However, risk of bias of primary studies might be an important driver of disagreement between RCTs and cohort studies, and needs to be addressed in future research. Risk of bias may affect especially results in individual cohort studies and contribute to statistical heterogeneity and wide confidence and prediction intervals [16].

## Conclusion

We were able to replicate the findings of our previous study, and showed that on average the pooled effect estimates between BoE from RCTs and cohort studies did not differ. However, the wide prediction intervals suggest that differences between BoE from RCTs and cohort studies could be considerably larger or smaller in either direction.

We observed that disagreement and wide prediction intervals were mainly driven by PI/ECO dissimilarities, i.e. by differences in intervention and comparator, and the direction of the effect estimate in cohort studies (RR < 1).

Future meta-research studies should take into consideration the assessment of risk of bias and the certainty in each BoE, and evaluate their influence on differences between findings from RCTs and cohort studies. A further promising step is to match primary studies by PI/ECO similarity and to assess their risk of bias using established tools for RCTs and cohort studies [99, 100]. This approach will also provide the possibility to account for differences in doses of intake or exposure.

### Supplementary Information

Below is the link to the electronic supplementary material.

Supplementary file1 Appendix S1 Changes made to the original study. Appendix S2 Search strategy for systematic reviews. Appendix S3 Criteria for Rating Population (P), Intervention/Exposure (I/E), Comparator (C), and Outcome (O) similarities. Appendix S4 Reasons for exclusion of systematic reviews. Table S1 Exclusion reasons for highly correlated outcomes. Table S2 Exclusion reasons for BoE-pairs due to overlap. Table S3 Minimal important differences (MID) for the included continuous outcomes. Table S4 Description of BoE-pairs. Table S5 Overview of transformations made to the original data extraction. Table S6 Characteristics of included BoE from randomised controlled trials. Table S7 Characteristics of included BoE from cohort studies. Table S8 Methodological quality assessment of the included systematic reviews. Table S9 Ratings of PI/ECO similarity degree for included BoE-pairs. Table S10 Analysis of concordance of the included BoE-pairs. Table S11 Subgroup analysis by PI/ECO similarity degree for each domain. Table S12 Overlap of primary studies in BoE-pairs with highly similar PI/ECO questions – comparison between the present sample and the sample in Schwingshackl 2021. Fig. S1 Forest plot, analysis of BoE-pairs with continuous outcomes and standardised mean difference. Fig. S2 Forest plot, subgroup analysis of BoE-pairs with binary outcomes by type of intervention/exposure. Fig. S3 Forest plot, subgroup analysis of BoE-pairs with binary outcomes by type of intake/exposure. Fig. S4 Forest plot, subgroup analysis of BoE-pairs with binary outcomes by type of outcome. Fig. S5 Forest plot, subgroup analysis of BoE-pairs with binary outcomes by overall PI/ECO similarity degree. Fig. S6 Forest plot, subgroup analysis of BoE-pairs with binary outcomes by population similarity degree. Fig. S7 Forest plot, subgroup analysis of BoE-pairs with binary outcomes by intervention/exposure similarity degree. Fig. S8 Forest plot, subgroup analysis of BoE-pairs with binary outcomes by comparator similarity degree. Fig. S9 Forest plot, subgroup analysis of BoE-pairs with binary outcomes by outcome similarity degree. Fig. S10 Forest plot, subgroup analysis of BoE-pairs with binary outcomes by AMSTAR 2 rating. Fig. S11 Forest plot, sensitivity analysis including one BoE-pair per systematic review. Fig. S12 Forest plot, sensitivity analysis by direction of cohort study summary effect estimate (cohort studies with risk ratio [RR] <1). Fig. S13 Forest plot, sensitivity analysis by direction of cohort study summary effect estimate (cohort studies with risk ratio [RR] ≥1). Fig. S14 Forest plot, sensitivity analysis for interventions with micronutrients. Fig. S15 Forest plot, sensitivity analysis for cancer outcomes. Fig. S16 Forest plot, sensitivity analysis excluding BoE-pairs with highly similar PI/ECO questions and overlapping primary studies. (PDF 4881 KB)

## Abbreviations

ACR: Assumed control risk;
AMSTAR 2: A measurement tool to assess systematic reviews, version 2
BoE: Body of evidence
CI: Confidence interval
DMD: Difference of mean differences
DSMD: Difference of standardised mean differences
IQR: Interquartile range
MD: Mean difference
OR: Odds ratio
PI: Prediction interval
PI/ECO: Population, intervention/exposure, comparator, outcome
PREDIMED: Prevención con Dieta Mediterránea
RCT: Randomised controlled trial
RR: Risk ratio
RRR: Ratio of risk ratios
SMD: Standardised mean difference
