# Supplementary material for: Evaluating agreement between evidence from randomised controlled trials and cohort studies in nutrition: a meta-research replication study
Source: Eur J Epidemiol. 2024 Jan 4;39(4):363–78. doi: 10.1007/s10654-023-01058-5 (PMC11101378; doi:10.1007/s10654-023-01058-5)
Supplement: Supplementary file 1 — Supplementary file1 Appendix S1 Changes made to the original study. Appendix S2 Search strategy for systematic reviews. Appendix S3 Criteria for Rating Population (P), Intervention/Exposure (I/E), Comparator (C), and Outcome (O) similarities. Appendix S4 Reasons for exclusion of systematic reviews. Table S1 Exclusion reasons for highly correlated outcomes. Table S2 Exclusion reasons for BoE-pairs due to overlap. Table S3 Minimal important differences (MID) for the included continuous outcomes. Table S4 Description of BoE-pairs. Table S5 Overview of transformations made to the original data extraction. Table S6 Characteristics of included BoE from randomised controlled trials. Table S7 Characteristics of included BoE from cohort studies. Table S8 Methodological quality assessment of the included systematic reviews. Table S9 Ratings of PI/ECO similarity degree for included BoE-pairs. Table S10 Analysis of concordance of the included BoE-pairs. Table S11 Subgroup analysis by PI/ECO similarity degree for each domain. Table S12 Overlap of primary studies in BoE-pairs with highly similar PI/ECO questions – comparison between the present sample and the sample in Schwingshackl 2021. Fig. S1 Forest plot, analysis of BoE-pairs with continuous outcomes and standardised mean difference. Fig. S2 Forest plot, subgroup analysis of BoE-pairs with binary outcomes by type of intervention/exposure. Fig. S3 Forest plot, subgroup analysis of BoE-pairs with binary outcomes by type of intake/exposure. Fig. S4 Forest plot, subgroup analysis of BoE-pairs with binary outcomes by type of outcome. Fig. S5 Forest plot, subgroup analysis of BoE-pairs with binary outcomes by overall PI/ECO similarity degree. Fig. S6 Forest plot, subgroup analysis of BoE-pairs with binary outcomes by population similarity degree. Fig. S7 Forest plot, subgroup analysis of BoE-pairs with binary outcomes by intervention/exposure similarity degree. Fig. S8 Forest plot, subgroup analysis of BoE-pairs with binary outcom [file 10654_2023_1058_MOESM1_ESM.pdf]

Supplementary material

Evaluating agreement between evidence from randomised controlled trials and cohort studies in nutrition: a meta-research replication study

Julia Stadelmaier[1], Jessica Beyerbach[1], Isabelle Roux[1], Louisa Harms[1], Julian Eble[1], Adriani Nikolakopoulou[2], Lukas Schwingshackl[1]

[1] Institute for Evidence in Medicine, Medical Center - University of Freiburg, Faculty of Medicine, University of Freiburg, Freiburg, Germany.

[2] Institute of Medical Biometry and Statistics, Medical Center - University of Freiburg, Faculty of Medicine, University of Freiburg, Freiburg, Germany.

Corresponding author: Julia Stadelmaier, MSc

Breisacher Straße 86, 79110 Freiburg, Germany

M: julia.stadelmaier@uniklinik-freiburg.de

P: +49 (0)761 270 85330

Table of contents

Supplementary Appendix S1 Changes made to the original study 4

Supplementary Appendix S2 Search strategy for systematic reviews 5

Supplementary Appendix S3 Criteria for Rating Population (P), Intervention/Exposure (I/E), Comparator (C), and Outcome (O) similarities 7

Supplementary Appendix S4 Reasons for exclusion of systematic reviews 9

Supplementary Table S1 Exclusion reasons for highly correlated outcomes 10

Supplementary Table S2 Exclusion reasons for BoE-pairs due to overlap 13

Supplementary Table S3 Minimal important differences (MID) for the included continuous outcomes 14

Supplementary Table S4 Description of BoE-pairs 15

Supplementary Table S5 Overview of transformations made to the original data extraction 23

Supplementary Table S6 Characteristics of included BoE from randomised controlled trials 36

Supplementary Table S7 Characteristics of included BoE from cohort studies 48

Supplementary Table S8 Methodological quality assessment of the included systematic reviews (AMSTAR 2) 57

Supplementary Table S9 Ratings of PI/ECO similarity degree for included BoE-pairs 60

Supplementary Table S10 Analysis of concordance of the included BoE-pairs 63

Supplementary Table S11 Subgroup analysis by PI/ECO similarity degree for each domain 67

Supplementary Table S12 Overlap of primary studies in BoE-pairs with highly similar PI/ECO questions – comparison between the present sample and the sample in Schwingshackl 2021 68

Supplementary Fig. S1 Forest plot, analysis of BoE-pairs with continuous outcomes and standardised mean difference 71

Supplementary Fig. S2 Forest plot, subgroup analysis of BoE-pairs with binary outcomes by type of intervention/exposure 72

Supplementary Fig. S3 Forest plot, subgroup analysis of BoE-pairs with binary outcomes by type of intake/exposure 73

Supplementary Fig. S4 Forest plot, subgroup analysis of BoE-pairs with binary outcomes by type of outcome 74

Supplementary Fig. S5 Forest plot, subgroup analysis of BoE-pairs with binary outcomes by overall PI/ECO similarity degree 75

Supplementary Fig. S6 Forest plot, subgroup analysis of BoE-pairs with binary outcomes by population similarity degree 76

Supplementary Fig. S7 Forest plot, subgroup analysis of BoE-pairs with binary outcomes by intervention/exposure similarity degree 77

Supplementary Fig. S8 Forest plot, subgroup analysis of BoE-pairs with binary outcomes by comparator similarity degree 78

Supplementary Fig. S9 Forest plot, subgroup analysis of BoE-pairs with binary outcomes by outcome similarity degree 79

Supplementary Fig. S10 Forest plot, subgroup analysis of BoE-pairs with binary outcomes by AMSTAR 2 rating 80

Supplementary Fig. S11 Forest plot, sensitivity analysis including one BoE-pair per systematic review 81

Supplementary Fig. S12 Forest plot, sensitivity analysis by direction of cohort study summary effect estimate (cohort studies with risk ratio [RR] <1) 82

Supplementary Fig. S13 Forest plot, sensitivity analysis by direction of cohort study summary effect estimate (cohort studies with risk ratio [RR] ≥1) 83

Supplementary Fig. S14 Forest plot, sensitivity analysis for interventions with micronutrients 84

Supplementary Fig. S15 Forest plot, sensitivity analysis for cancer outcomes...................................... 85

Supplementary Fig. S16 Forest plot, sensitivity analysis excluding BoE-pairs with highly similar PI/ECO questions and overlapping primary studies.. 86

Supplementary Appendix S1 Changes made to the original study

	Schwingshackl 2021 [1]	present replication study
**Sample**	Systematic reviews of RCTs (Cochrane reviews) and systematic reviews of cohort studies of the same patient-relevant outcome or intermediate disease marker*<br>→ matching approach<br><br>Exclusion of highly correlated outcomes in post hoc sensitivity analyses	Systematic reviews including both RCTs and cohort studies of the same patient-relevant outcome or intermediate disease marker*<br><br>Exclusion of highly correlated outcomes<br><br>Exclusion of systematic review with overlapping PI/ECO with another included systematic review
**Search**	database: Cochrane Database of Systematic Reviews and Medline (via OVID)<br><br>filter: published between 1 January 2010 and 31 December 2019	database: Cochrane Database of Systematic Reviews, Medline (via OVID), and Epistemonikos<br><br>filter: published between 1 January 2011 and 6 September 2021
**Screening**	Title and abstracts were screened by one reviewer, and full texts by two reviewers independently.	Title and abstracts, and full texts were screened by two reviewers independently.
**Selection of outcomes**	Based on the ranking in the summary of findings table in the identified Cochrane reviews (from top to bottom)	Based on the list of primary outcomes, and thereafter selection with an top down approach (highest number of studies included in BoE from RCTs; highest number of study participants; highest number of cases)
**Additional analyses**	N/A	AMSTAR 2 assessment<br><br>Analysis of concordance

* There is an overlap of nine systematic reviews between both samples: The reviews of Aburto 2013 [2], Chowdhury 2014 [3], Feng 2015 [4], Jiang 2019 [5], Kastorini 2011 [6], Vinceti 2018 [7], Wolf 2017 [8] and Zhang 2016 [9] were also included in the review by Schwingshackl 2021 [1].
AMSTAR 2=a measurement tool to assess systematic reviews, version 2; BoE=body of evidence; PI/ECO=population, intervention/exposure, comparator, outcome; RCT=randomised controlled trial

Supplementary Appendix S2 Search strategy for systematic reviews

**Medline (via Ovid), date: 06.09.2021**	
**ID**	**Query**
#1	Systematic Review/ or (systematic-review or systematic literature review or systematic search).ti,ab,kf or systematic review.pt.
#2	Meta-analysis/ or (meta-analys* or meta analys* or metaanalys* or metasynthe* or meta-synthe* or meta-review or methodological review).ti,ab,kf or meta-analysis.pt.
#3	(randomi* or randomly or RCT or RCTs or (intervention* adj2 (stud* or trial*)) or (controlled adj1 (stud* or trial*))).ti,ab,kf.
#4	Cohort Studies/ or (cohort* or observational or longitudinal or prospective or case-cohort or nested case-control or (epidemiologic* adj2 stud*)).ti,ab,kf.
#5	(#1 or #2) and #3 and #4
#6	Diet/ or (mediterranean or vegetarian or low-carb* or low-fat or high-protein or gluten* or FODMAP*).ti,ab,kf.
#7	Edible Grain/ or Fruit/ or Vegetables/ or Milk/ or Dairy Products/ or Meat/ or Eggs/ or Nuts/ or Chocolate/ or Oil/ or beverages/ or (grain* or fruit* or vegetabl* or legume* or milk or dairy or soy or meat or fish or eggs or nuts or chocolate or oil* or beverage*).ti,ab,kf.
#8	Dietary Carbohydrates/ or Dietary Fats/ or Dietary Proteins/ or (carbohydrat* or fructose or glucose or starch or sucrose or sweetener* or aspartame or saccharin* or protein* or amino acid* or fatty acid* or omega or unsaturated or monounsaturated or polyunsaturated or eicosapentaenoic or docosahexaenoic or linoleic acid*).ti,ab,kf.
#9	Micronutrients/ or Trace Elements/ or Vitamins/ or<br>(Vitamin* or multivitamin* or retinol or caroten* or thiamine or riboflavin or niacin or pantothenic or pyridoxine or biotin or folic or folate or folacin or cobalamin or ascorbic or ascorbate or calciferol* or cholecalciferol* or ergocalciferol* or tocopherole or tocotrienol or phylloquinone or menaquinone or calcium or chromium or copper or iodine or iron or fluorine or fluoride or magnesium or manganese or phosphor* or potassium or selenium or sodium or zinc).ti,ab,kf.
#10	Dietary Fiber/ or Dietary Supplements/ or (fibre* or fiber* or psyllium or inulin or cellulose or prebiotic* or probiotic* or symbiotic*).ti,ab,kf.
#11	(diet* or nutrition or eat* or feed* or food* or consum* or intake* or supplement*).ti,ab,kf.
#12	(#6 or #7 or #8 or #9 or #10) and #11
#13	#5 and #12
	limit 13 to last 10 years
	**results: 1,351**

**Cochrane Library (via Website, advanced search), date: 06.09.2021**	
**ID**	**Query**
#1	(randomi* or randomly or RCT or RCTs):ti,ab,kw
#2	(controlled NEAR/1 (stud* or trial)):ti,ab,kw
#3	(clinical NEAR/2 (stud* or trial)):ti,ab,kw
#4	#1 or #2 or #3
#5	(cohort* or observational or longitudinal or prospective or case-cohort or “nested case-control” or epidemiologic*):ti,ab,kw
#6	MeSH descriptor: [Cohort Studies] explode all trees
#7	#5 or #6
#8	#4 and #7
#9	(diet* or mediterranean or vegetarian or low-carb* or low-fat or high-protein or gluten* or FODMAP*):ti,ab,kw
#10	MeSH descriptor: [Diet] explode all trees
#11	(grain* or fruit* or vegetabl* or legume* or milk or dairy or soy or meat or fish or eggs or nuts or chocolate or oil* or beverage*):ti,ab,kw
#12	MeSH descriptor: [Food] explode all trees
#13	MeSH descriptor: [Beverages] explode all trees

#14	(carbohydrat* or fructose or glucose or starch or sucrose or sweetener* or aspartame or saccharin* or omega or unsaturated or monounsaturated or polyunsaturated or eicosapentaenoic or docosahexaenoic or “linoleic acid*” or “fatty acid*” or protein* or “amino acid*”):ti,ab,kw
#15	MeSH descriptor: [Dietary Carbohydrates] explode all trees
#16	MeSH descriptor: [Dietary Fats] explode all trees
#17	MeSH descriptor: [Fatty acids] explode all trees
#18	MeSH descriptor: [Dietary Proteins] explode all trees
#19	(Vitamin* or multivitamin* or retinol or caroten* or thiamine or riboflavin or niacin or pantothenic or pyridoxine or biotin or folic or folate or folacin or cobalamin or ascorbic or ascorbate or calciferol* or cholecalciferol* or ergocalciferol* or tocopherole or tocotrienol or phylloquinone or menaquinone or calcium or chromium or copper or iodine or iron or fluorine or fluoride or magnesium or manganese or phosphor* or potassium or selenium or sodium or zinc):ti,ab,kw
#20	MeSH descriptor: [Micronutrients] explode all trees
#21	(fibre* or fiber* or psyllium or inulin or cellulose or prebiotic* or probiotic* or symbiotic*):ti,ab,kw
#22	#9 or #10 or #11 or #12 or #13 or #14 or #15 or #16 or #17 or #18 or #19 or #20 or #21
#23	(diet* or nutrition or eat* or feed* or food* or consum* or intake* or supplement*):ti,ab,kw
#24	#22 and #23
#25	#8 and #24
	#25 in Cochrane Reviews
	with Cochrane Library publication date from Jan 2011 to Sep 2021
	**results: 126**

**Epistemonikos (via Website, advanced search, title/abstract), 06.09.2021**	
**ID**	**Query**
#1	“systematic review" OR systematic-review OR "systematic literature review" OR "systematic search”
#2	meta-analys* OR "meta analys*" OR metaanalys* OR metasynthe* OR meta-synthe* OR meta-review OR "methodological review"
#3	#1 or #2
#4	randomi* OR randomly OR RCT OR RCTs OR “intervention* studies” OR “intervention* trials” OR “controlled studies” OR “controlled trials”
#5	cohort* OR observational OR longitudinal OR prospective OR case-cohort OR "nested case-control" OR epidemiologic*
#6	#3 and #4 and #7
#7	diet* OR mediterranean OR vegetarian OR low-carb* OR low-fat or high-protein OR gluten* OR FODMAP*
#8	grain* OR fruit* OR vegetabl* OR legume* OR milk OR dairy OR soy OR meat OR fish OR eggs OR nuts OR chocolate OR oil* OR beverage*
#9	carbohydrat* OR fructose OR glucose OR starch OR sucrose OR sweetener* OR aspartame OR saccharin* OR omega OR unsaturated OR monounsaturated OR polyunsaturated OR eicosapentaenoic OR docosahexaenoic OR “linoleic acid*” OR “fatty acid*” OR protein* OR “amino acid*”
#10	Vitamin* OR multivitamin* OR retinol OR caroten* OR thiamine OR riboflavin OR niacin OR pantothenic OR pyridoxine OR biotin OR folic OR folate or folacin OR cobalamin OR ascorbic OR ascorbate OR calciferol* OR cholecalciferol* OR ergocalciferol* or tocopherole OR tocotrienol OR phylloquinone OR menaquinone OR calcium OR chromium OR copper OR iodine OR iron OR fluorine OR fluoride OR magnesium OR manganese OR phosphor* OR potassium OR selenium OR sodium OR zinc
#11	fibre* OR fiber* OR psyllium OR inulin OR cellulose OR prebiotic* OR probiotic* OR symbiotic*
#12	#7 OR #8 OR #9 OR #10 OR #11
#13	diet* OR nutrition OR eat* OR feed* or food* OR consum* OR intake* OR supplement*
#14	#12 AND #13
#15	#6 AND #14
	publication year: “Last 10 years”
	**results: 1,408**

Supplementary Appendix S3 Criteria for Rating Population (P), Intervention/Exposure (I/E), Comparator (C), and Outcome (O) similarities (modified from Schwingshackl 2021 [1])

Rating	Population	Intervention/ Exposure	Comparator	Outcome
**1 = more or less identical**	**Both BoE include primary or secondary prevention**<br><br>Example:<br>- both BoE with healthy population, general population<br>- both BoE with diseased population (e.g. people with cardiovascular disease)<br><br>**Both BoE include same type of population regarding basic characteristics (age, gender)**<br><br>Example:<br>- both BoE with adults<br>- both BoE with postmenopausal women	**Both BoE use intake, supplementation, or status**<br><br>Example:<br>- folate supplementation in BoE from RCTs and BoE from cohort studies<br>- apple intake in BoE from RCTs and BoE from cohort studies	**Both BoE use nil/ low intake, supplementation/ placebo, or status.**<br><br>Example:<br>- no/ low intake from BoE from RCTs and BoE from cohort studies	**Both BoE examine the same outcome.**<br><br>Example:<br>- all-cause mortality in BoE from RCTs and BoE from cohort studies
**2 = similar but not identical**	**Both BoE include primary and secondary prevention or mixed population vs. primary or secondary prevention or high-risk population vs. primary or secondary prevention**<br><br>Example:<br>- mixed population (healthy people and partly people with cardiovascular disease) in BoE from RCTs vs. general healthy population in BoE from cohort studies<br>- population at high-risk in BoE from RCTs vs. general healthy population in BoE from cohort studies	**Both BoE use similar intervention/ exposure:**<br>**- similar products with different routes of administration**<br>**- different products of the same class**<br>**- similar products with different co-interventions**<br>**- similar time frame of intervention**<br><br>Example:<br>- intake vs. supplementation; intake vs. urinary excretion; intake + supplementation vs. intake<br>- supplementation vs. supplementation of similar products (multi-micronutrient supplementation vs. multivitamin supplementation)<br>- intake of product + medicament (e.g. aspirin) vs. intake of product	**BoE use similar but not identical comparator.**<br><br>Example:<br>see Intervention/ Exposure	**Both BoE examine the same cluster of outcomes.**<br><br>Example:<br>- cardiovascular disease in BoE from RCTs and coronary heart disease in BoE from cohort studies<br>- cardiovascular mortality in BoE from RCTs vs. cardiovascular incidence + mortality in BoE from cohort studies

**3 =**<br><br>**broadly similar**	**One BoE include primary prevention and the other BoE (mostly) secondary prevention**<br><br>Example:<br>- people with cancer in BoE from RCTs vs. general healthy population in BoE from cohort studies<br>- mixed population (most [≥66%] of the people with cardiovascular disease) in BoE from RCTs vs. general population in BoE from cohort studies	**BoE use broadly similar intervention/ exposure:**<br>**- intake or supplementation vs. status**<br>**- broadly similar products**<br>**- different time frame/ early treatment vs. any treatment**<br><br>Example:<br>- selenium supplementation vs. high selenium status<br>- Vitamin A supplementation vs. ß-carotene intake	**BoE use broadly similar comparator.**<br><br>Example:<br>see Intervention/ Exposure	**BoE examine similar outcomes.**<br><br>Example:<br>- colorectal adenoma in BoE from RCTs vs. colorectal cancer in BoE from cohort studies

BoE=bodies of evidence; RCT=randomised controlled trial

Supplementary Appendix S4 Reasons for exclusion of systematic reviews

References	Reason for exclusion
[10-98]	Ineligible study design
[99-130]	Ineligible PI/ECO
[131-151]	No RCTs or cohort studies included or RCTs analysed as cohort studies
[152-187]	Intervention/exposure or outcome differ
[188-203]	Ineligible effect size
[204-206]	Updated version included
[207-217]	Overlapping PI/ECO with another included systematic review

PI/ECO=population, intervention/exposure, comparator, outcome; RCT=randomised controlled trial

Supplementary Table S1 Exclusion reasons for highly correlated outcomes

Reference: Author, Year	Intervention	Outcome	Reason for exclusion
Aburto 2013 [2]	Sodium	Stroke	Highly likely correlated with outcome “Cardiovascular disease”
Aburto 2013 [2]	Sodium	Coronary heart disease	Highly likely correlated with outcome “Cardiovascular disease”
Aguilar-Cordero 2020 [218]	Vitamin D (maternal)	Prematurity	Highly likely correlated with outcome “Pre-eclampsia”
Alexander 2017 [219]	Omega-3 fatty acids	Any fatal coronary heart disease event	Highly likely correlated with outcome “Coronary heart disease”
Alexander 2017 [219]	Omega-3 fatty acids	Any nonfatal coronary heart disease event	Highly likely correlated with outcome “Coronary heart disease”
Alexander 2017 [219]	Omega-3 fatty acids	Coronary death	Highly likely correlated with outcome “Coronary heart disease”
Azad 2017 [220]	Nonnutritive sweeteners	Weight (change)	Highly likely correlated with outcome “Body mass index”
Azad 2017 [220]	Nonnutritive sweeteners	Waist circumference	Highly likely correlated with outcome “Body mass index”
Bolland 2015 [221]	Calcium	Hip fractures	Highly likely correlated with outcome “All fractures”
Bolland 2015 [221]	Calcium	Vertebral fractures	Highly likely correlated with outcome “All fractures”
Bolland 2015 [221]	Calcium	Forearm fractures	Highly likely correlated with outcome “All fractures”
Grosso 2015 [222]	Mediterranean diet	Myocardial infarction	Highly likely correlated with outcome “Cardiovascular disease”
Grosso 2015 [222]	Mediterranean diet	Stroke	Highly likely correlated with outcome “Cardiovascular disease”
Grosso 2015 [222]	Mediterranean diet	Cardiovascular disease mortality	Highly likely correlated with outcome “Cardiovascular disease”
Kim 2018 [223]	Multivitamins/ Minerals	Coronary heart disease mortality	Highly likely correlated with outcome “Cardiovascular disease mortality”

Kim 2018 [223]	Multivitamins/ Minerals	Stroke mortality	Highly likely correlated with outcome “Cardiovascular disease mortality”
Stratton 2011 [224]	Vitamin E	Metastatic prostate cancer/ death from prostate cancer	Highly likely correlated with outcome “Prostate cancer”
Stratton 2011 [224]	β-carotene	Metastatic prostate cancer/ death from prostate cancer	Highly likely correlated with outcome “Prostate cancer”
Thorne-Lyman 2012 [225]	Vitamin D	Mean gestational age	Highly likely correlated with outcome “Mean birth weight”
Vinceti 2018a [7]	Selenium	Colorectal cancer	Highly likely correlated with outcome “Any cancer”
Vinceti 2018a [7]	Selenium	Lung cancer	Highly likely correlated with outcome “Any cancer”
Vinceti 2018a [7]	Selenium	Breast cancer	Highly likely correlated with outcome “Any cancer”
Vinceti 2018a [7]	Selenium	Bladder cancer	Highly likely correlated with outcome “Any cancer”
Vinceti 2018a [7]	Selenium	Prostate cancer	Highly likely correlated with outcome “Any cancer”
Wien 2012 [226]	Folic acid	Pancreatic cancer	Highly likely correlated with outcome “Any cancer”
Wien 2012 [226]	Folic acid	Breast cancer	Highly likely correlated with outcome “Any cancer”
Wien 2012 [226]	Folic acid	Lung cancer	Highly likely correlated with outcome “All cancer”
Wolf 2017 [8]	Multivitamins	Low birthweight	Highly likely correlated with outcome “Preterm birth”
Wolf 2017 [8]	Multivitamins	Small for gestational age	Highly likely correlated with outcome “Preterm birth”
Wolf 2017 [8]	Multivitamins	Neural tube defect	Highly likely correlated with outcome “Stillbirth”
Yang 2020 [227]	Calcium	Coronary heart disease	Highly likely correlated with outcome “Cardiovascular disease”
Yang 2020 [227]	Calcium	Stroke	Highly likely correlated with outcome “Cardiovascular disease”

Yao 2019 [228]	Vitamin D	Hip fracture	Highly likely correlated with outcome “Any fracture”
Yu 2021 [229]	Folic acid	Gestational hypertension	Highly likely correlated with outcome “Pre-eclampsia”
Zhang 2016 [9]	Selenium	Coronary heart disease	Highly likely correlated with outcome “Cardiovascular disease”
Zhang 2016 [9]	Selenium	Myocardial infarction	Highly likely correlated with outcome “Cardiovascular disease”
Zhang 2018 [230]	Vitamin D	Fasting insulin	Highly likely correlated with outcome “Fasting plasma glucose”
Zhang 2018 [230]	Vitamin D	HOMA-IR	Highly likely correlated with outcome “Fasting plasma glucose”
Zhang 2018 [230]	Vitamin D	HbA1c	Highly likely correlated with outcome “Fasting plasma glucose”

HbA1c=Haemoglobin A1c; HOMA-IR=Homeostasis model assessment-estimated insulin resistance

Supplementary Table S2 Exclusion reasons for BoE-pairs due to overlap

Reference: Author, Year	Intervention/Exposure	Outcome	Reason
Amegah 2017 [231]	Vitamin D	Preterm birth	Most of the included studies (5/7) are also identified by Zhou 2017 [232].
Becerra-Tomas 2020 [207]	Mediterranean diet	Cardiovascular disease mortality	Some of the included studies (4/18) are identified by Grosso 2015 [222], which also used a larger sample of studies for BoE from RCTs (n=4) and BoE from cohort studies (n=20).
Bloomfield 2016 [208]	Mediterranean diet	Breast cancer	Some of the included studies (4/14) are identified by Morze 2021 [233], which also used the corrected publication of the PREDIMED for BoE from RCTs.
Chowdhury 2014a [234]	Omega-3 fatty acids	Coronary disease	Many of the included studies (14/25) are also identified by Alexander 2017 [219], which also used a larger sample of studies for BoE from RCTs (n=18) and BoE from cohort studies (n=17).
Chung 2016 [209]	Calcium	Cardiovascular disease mortality	Some of the included studies (2/6) are also identified by Yang 2020 [227], which also used a larger sample of studies for BoE from RCTs (n=4) and BoE from cohort studies (n=13).
Hua 2016 [210]	Folic acid	Gestational hypertension and pre-eclampsia	All of the included studies (6/6) are identified by Yu 2021 [229] or Yang 2016 [235].
Hyppönen 2013 [211]	Vitamin D	Pre-eclampsia	All of the included studies (6/6) are identified by Aguilar-Cordero 2020 [218].
Kohler 2018 [212]	Selenium	Type 2 diabetes	All of the included studies (4/4) are identified by Vinceti 2018b [236].
Koloverou 2014 [213]	Mediterranean diet	Type 2 diabetes	Most of the included studies (9/10) are identified by Schwingshackl 2015 [237], which also used a larger sample of the PREDIMED study for BoE from RCTs [238].
Kuria 2020 [214]	Selenium	Cardiovascular disease	All of the included studies (7/7) are also identified by Zhang 2016 [9].
Liu 2018 [215]	Folic acid	Pre-eclampsia	All of the included studies (10/10) are identified by Yu 2021 [229].
Martinez-Gonzalez 2014a [216]	Mediterranean diet	Cardiovascular disease	All of the included studies (14/14) are also identified by Grosso 2015 [222].
Shim 2016 [217]	Folic acid	Gestational hypertension	All of the included studies (2/2) are also identified by Yang 2016 [235].
Trikalinos 2012 [239]	Omega-3 fatty acids	Cardiovascular mortality	Most of the included studies (11/16) are identified by Alexander 2017 [219], which also used a larger sample of studies for BoE from RCTs (n=18) and BoE from cohort studies (n=17).

BoE=bodies of evidence; PREDIMED=Prevención con Dieta Mediterránea; RCT=randomised controlled trial

Supplementary Table S3 Minimal important differences (MID) for the included continuous outcomes

Outcome	Normal value	MID	Lower 95% CI	Upper 95% CI
Body weight	75 kg	2.5%	-1.9	1.9
Body mass index	24.4	2.5%	-1.2	1.2
Birth weight	3,400 g	2.5%	-85	85
Gestational length	40 weeks	2.5%	-1	1
HDL-cholesterol	1.036 mmol/l	5%	-0.05	0.05
Mini Mental State Examination Score	>26	5%	-1.3	1.3
Systolic blood pressure	120 mmHg	2.5%	-3	3
Triglyceride	1.7 mmol/l	2.5%	-0.04	0.04

CI=confidence interval; HDL=high density lipoprotein; MID=minimal important difference

Supplementary Table S4 Description of BoE-pairs

	BoE from randomised controlled trials					BoE from cohort studies					
**Reference**	**Intervention and type of intake**	**Comparison**	**Outcome**	**Studies** (n)	**Summary measure, and effect estimate** (95% CI)	**Exposure and type of intake**	**Comparison**	**Outcome**	**Studies** (n)	**Summary measure, and effect estimate** (95% CI)	**Included in the meta-analysis** (Yes/ No)
Aburto 2013 [2]	Sodium/ Intake	Low vs. High	All-cause mortality	4	RR 0.70 (0.44 to 1.14)	Sodium/ Intake	High vs. Low	All-cause mortality	2	RR 1.06 (0.94 to 1.20)	Yes
Aburto 2013 [2]	Sodium/ Intake	Low vs. High	Cardiovascular disease	2	RR 0.84 (0.57 to 1.23)	Sodium/ Intake	High vs. Low	Cardiovascular disease	3	RR 1.12 (0.93 to 1.34)	Yes
Afshin 2014 [240]	Nuts/ Intake	Dose-response; per 4 weekly servings (28.4g) of nuts	Coronary heart disease	1	RR 0.79 (0.55 to 1.14)	Nuts/ Intake	Dose-response; per 4 weekly servings (28.4g) of nuts	Coronary heart disease	6	RR 0.76 (0.69 to 0.83)	Yes
Afshin 2014 [240]	Nuts/ Intake	Dose-response; per 4 weekly servings (28.4g) of nuts	Stroke	1	RR 0.62 (0.44 to 0.87)	Nuts/ Intake	Dose-response; per 4 weekly servings (28.4g) of nuts	Stroke	2	RR 0.95 (0.83 to 1.09)	Yes
Afshin 2014 [240]	Nuts/ Intake	Dose-response; per 4 weekly servings (28.4g) of nuts	Type 2 diabetes	1	RR 0.86 (0.68 to 1.08)	Nuts/ Intake	Dose-response; per 4 weekly servings (28.4g) of nuts	Type 2 diabetes	4	RR 0.87 (0.80 to 0.95)	Yes
Aguilar-Cordero 2020 [218]	Vitamin D/ Supplementation	High vs. Low	Pre-eclampsia	7	OR 0.61 (0.36 to 1.04)	Vitamin D/ Status	Low vs. High	Pre-eclampsia	7	OR 1.25 (0.89 to 1.75)	Yes
Alexander 2017 [219]	Omega-3 fatty acids/ Intake + Supplementation	High vs. Low	Coronary heart disease	18	RR 0.94 (0.85 to 1.05)	Omega-3 fatty acids/ Intake	High vs. Low	Coronary heart disease	17	RR 0.82 (0.74 to 0.92)	Yes

Amegah 2017 [231]	Vitamin D/ Supplementation	High vs. Low	Gestational length	2	MD 0.16 (0.28 to 0.59)	Vitamin D/ Status	Low vs. High	Gestational length	1	MD 0.80 (0.20 to 1.40)	Yes
Azad 2017 [220]	Nonnutritive sweeteners/ Intake + Supplementation	High vs. Low	Body mass index	3	MD -0.37 (-1.10 to 0.36)	Nonnutritive sweeteners/ Intake	High vs. Low	Body mass index	1	MD 0.77 (0.47 to 1.07)	Yes
Bolland 2015 [221]	Calcium/ Supplementation	High vs. Low	All fractures	20	RR 0.89 (0.81 to 0.96)	Calcium/ Supplementation	High vs. Low	All fractures	5	RR 1.02 (0.93 to 1.11)	Yes
Chowdhury 2012 [241]	Omega-3 fatty acids/ Supplementation	High vs. Low	Cerebrovascula r disease	2	RR 0.98 (0.89 to 1.08)	Omega-3 fatty acids/ Intake	High vs. Low	Cerebrovascula r disease	10	RR 0.90 (0.80 to 1.01)	Yes
Chowdhury 2014a [234]	α-linolenic acid/ Supplementation	High vs. Low	Coronary disease	4	RR 0.97 (0.69 to 1.36)	α-linolenic acid/ Intake	High vs. Low	Coronary disease	7	RR 0.99 (0.86 to 1.14)	Yes
Chowdhury 2014a [234]	Omega-6 fatty acids/ Intake + Supplementation	High vs. Low	Coronary disease	8	RR 0.86 (0.69 to 1.07)	Omega-6 fatty acids/ Intake	High vs. Low	Coronary disease	8	RR 0.98 (0.90 to 1.06)	Yes
Chowdhury 2014b [3]	Vitamin D/ Supplementation	High vs. Low	All-cause mortality	22	RR 0.98 (0.94 to 1.03)	Vitamin D/ Status	Low vs. High	All-cause mortality	68	RR 1.44 (1.34 to 1.55)	Yes
Chung 2011 [242]	Vitamin D/ Supplementation	High vs. Low	Colorectal cancer	1	RR 1.02 (0.60 to 1.74)	Vitamin D/ Status	Dose-response; per 10nmol/l increase	Colorectal cancer	9	OR 0.94 (0.91 to 0.97)	Yes
Chung 2011 [242]	Vitamin D/ Supplementation	High vs. Low	Breast cancer	1	RR 0.99 (0.25 to 4.00)	Vitamin D/ Status	Dose-response; per 10nmol/l increase	Breast cancer	4	OR 0.99 (0.97 to 1.01)	Yes
Ding 2017 [243]	Dairy/ Intake	High vs. Low	Systolic blood pressure	8	MD -0.21 (-0.98 to 0.57)	Dairy/ Intake	Dose-response; per serving increase	Systolic blood pressure	27	MD -0.11 (-0.20 to -0.02)	Yes
Feng 2015 [4]	Folic acid/ Supplementation	High vs. Low	Congenital heart defects	1	OR 0.42 (0.19 to 0.98)	Folic acid/ Supplementation	High vs. Low	Congenital heart defects	1	OR 0.60 (0.38 to 0.96)	Yes

Filippini 2020 [244]	Green Tea/ Supplementation	High vs. Low	Prostate cancer	3	RR 0.50 (0.18 to 1.36)	Green Tea/ Intake	High vs. Low	Prostate cancer	5	RR 1.09 (0.89 to 1.32)	Yes
Filippini 2020 [244]	Green Tea/ Supplementation	High vs. Low	Endometrial cancer	1	RR 0.33 (0.01 to 8.15)	Green Tea/ Intake	High vs. Low	Endometrial cancer	1	RR 0.75 (0.43 to 1.30)	Yes
Fu 2021 [245]	Vitamin C/ Supplementation	High vs. Low	Lung cancer	2	RR 1.30 (0.68 to 2.48)	Vitamin C/ Supplementation	High vs. Low	Lung cancer	3	RR 1.02 (0.85 to 1.23)	Yes
Fu 2021 [245]	Vitamin C/ Supplementation	High vs. Low	Breast cancer	1	RR 1.11 (0.87 to 1.41)	Vitamin C/ Supplementation	High vs. Low	Breast cancer	6	RR 1.02 (0.88 to 1.18)	Yes
Fu 2021 [245]	Vitamin C/ Supplementation	High vs. Low	Colorectal cancer	2	RR 0.84 (0.64 to 1.10)	Vitamin C/ Supplementation	High vs. Low	Colorectal cancer	2	RR 0.80 (0.42 to 1.52)	Yes
Gayer 2019 [246]	Apples/ Intake	High vs. Low	Body weight	5	MD 1.14 (-0.45 to 0.73)	Apples/ Intake	Dose-response; per serving/day	Body weight	1	MD 0.56 (0.39 to 0.73)	Yes
Grosso 2015 [222]	Mediterranean diet/ Intake	High vs. Low adherence	Cardiovascular disease	4	RR 0.55 (0.39 to 0.76)	Mediterranean diet/ Intake	High vs. Low adherence	Cardiovascular disease	20	RR 0.71 (0.65 to 0.78)	Yes
Jiang 2019 [5]	Vitamin E/ Supplementation	High vs. Low	Age-related cataract	6	RR 0.97 (0.91 to 1.03)	Vitamin E/ Intake	High vs. Low	Age-related cataract	6	RR 0.90 (0.80 to 1.00)	Yes
Jiang 2019 [5]	β-carotene/ Supplementation	High vs. Low	Age-related cataract	4	RR 0.99 (0.92 to 1.07)	β-carotene/ Intake	High vs. Low	Age-related cataract	7	RR 0.90 (0.83 to 0.99)	Yes
Johnston 2019 [247]	Red meat/ Intake	Low vs. High	Cardiovascular disease	1	HR 0.98 (0.91 to 1.06)	Red meat/ Intake	Low vs. High	Cardiovascular disease	25	RR 0.86 (0.79 to 0.94)	Yes
Johnston 2019 [247]	Red meat/ Intake	Low vs. High	Type 2 diabetes	1	HR 0.96 (0.90 to 1.03)	Red meat/ Intake	Low vs. High	Type 2 diabetes	14	RR 0.76 (0.68 to 0.86)	Yes
Johnston 2019 [247]	Red meat/ Intake	Low vs. High	Colorectal cancer	1	HR 1.04 (0.90 to 1.20)	Red meat/ Intake	Low vs. High	Colorectal cancer	16	RR 0.94 (0.85 to 1.05)	Yes

Jonker 2020 [248]	Folic acid/ Supplementation	High vs. Low	Mean birthweight	3	SMD 0.56 (0.15 to 0.97)	Folic acid/ Supplementation	High vs. Low	Mean birthweight	2	SMD 0.33 (0.19 to 0.47)	Yes
Jonker 2020 [248]	Folic acid/ Supplementation	High vs. Low	Low birthweight	1	OR 0.93 (0.81 to 1.08)	Folic acid/ Supplementation	High vs. Low	Low birthweight	5	OR 0.58 (0.46 to 0.73)	Yes
Jonker 2020 [248]	Folic acid/ Supplementation	High vs. Low	Small for gestational age	1	OR 0.98 (0.92 to 1.06)	Folic acid/ Supplementation + Intake	High vs. Low	Small for gestational age	4	OR 0.62 (0.29 to 1.31)	Yes
Kastorini 2011 [6]	Mediterranean diet/ Intake	High vs. Low adherence	Metabolic syndrome	2	logHR -0.80 (-1.42 to -0.18)	Mediterranean diet/ Intake	High vs. Low adherence	Metabolic syndrome	2	logHR -0.76 (-2.16 to 0.63)	Yes
Kastorini 2011 [6]	Mediterranean diet/ Intake	High vs. Low adherence	HDL-cholesterol	27	MD 1.25 (0.52 to 1.99)	Mediterranean diet/ Intake	High vs. Low adherence	HDL-cholesterol	1	MD 0.30 (-1.77 to 2.37)	Yes
Kastorini 2011 [6]	Mediterranean diet/ Intake	High vs. Low adherence	Triglycerides	27	MD -6.45 (-10.91 to -1.99)	Mediterranean diet/ Intake	High vs. Low adherence	Triglycerides	1	MD -2.00 (-6.74 to 2.74)	Yes
Kastorini 2011 [6]	Mediterranean diet/ Intake	High vs. Low adherence	Systolic blood pressure	14	MD -2.35 (-3.51 to -1.18)	Mediterranean diet/ Intake	High vs. Low adherence	Systolic blood pressure	1	MD 0.80 (-0.84 to 2.44)	Yes
Kim 2018 [223]	Multivitamins/ Minerals/ Supplementation	High vs. Low	Cardiovascular disease	1	RR 0.95 (0.83 to 1.09)	Multivitamins/ Minerals/ Supplementation	High vs. Low	Cardiovascular disease	9	RR 1.00 (0.96 to 1.05)	Yes
Kim 2018 [223]	Multivitamins/ Minerals/ Supplementation	High vs. Low	Coronary heart disease	2	RR 0.97 (0.80 to 1.19)	Multivitamins/ Minerals/ Supplementation	High vs. Low	Coronary heart disease	6	RR 0.90 (0.85 to 0.96)	Yes
Kim 2018 [223]	Multivitamins/ Minerals/ Supplementation	High vs. Low	Stroke	1	RR 1.05 (0.70 to 1.56)	Multivitamins/ Minerals/ Supplementation	High vs. Low	Stroke	3	RR 0.98 (0.91 to 1.04)	Yes
Kong 2014 [249]	Vitamins/ Supplementation	High vs. Low	Gastric cancer	11	RR 1.11 (0.94 to 1.31)	Vitamins/ Intake	High vs. Low	Gastric cancer	7	RR 0.85 (0.66 to 1.08)	Yes
Lin 2019 [250]	Vitamin E/ Supplementation	High vs. Low	Bladder cancer	3	RR 1.10 (0.86 to 1.35)	Vitamin E/ Intake	High vs. Low	Bladder cancer	8	RR 0.84 (0.72 to 0.96)	Yes

Lin 2020 [251]	Vitamin D/ Supplementation	High vs. Low	Nephrolithiasis	7	RR 0.84 (0.65 to 1.10)	Vitamin D/ Supplementation	High vs. Low	Nephrolithiasis	3	RR 1.24 (1.01 to 1.51)	Yes
Lin 2020 [251]	Calcium/ Supplementation	High vs. Low	Nephrolithiasis	4	RR 1.20 (0.65 to 2.21)	Calcium/ Supplementation	High vs. Low	Nephrolithiasis	2	RR 1.16 (1.00 to 1.35)	Yes
Martinez-Gonzalez 2014b [252]	Olive oil/ Supplementation + Intake	Dose-response; per 25g/day increase	Coronary heart disease	1	RR 0.81 (0.53 to 1.24)	Olive oil/ Intake	Dose-response; per 25g/day increase	Coronary heart disease	3	RR 0.96 (0.78 to 1.18)	Yes
Martinez-Gonzalez 2014b [252]	Olive oil/ Supplementation + Intake	Dose-response; per 25g/day increase	Stroke	1	RR 0.69 (0.48 to 0.98)	Olive oil/ Intake	Dose-response; per 25g/day increase	Stroke	2	RR 0.74 (0.60 to 0.92)	Yes
Miller 2014 [253]	Low-calorie sweeteners/ Intake	High vs. Low	Body weight	13	WGMD -0.80 (-1.17 to -0.43)	Low-calorie sweeteners/ Intake	Mean correlation	Body weight	4	WGMC 0.02 (-0.01 to 0.06)	No
Moazzen 2018 [254]	Folic acid/ Supplementation	High vs. Low	Colorectal cancer	4	RR 1.07 (0.86 to 1.43)	Folic acid/ Supplementation	High vs. Low	Colorectal cancer	3	RR 0.96 (0.76 to 1.21)	Yes
Mocellin 2017 [255]	Vitamin B6/ Supplementation	High vs. Low	Cancer	9	RR 1.03 (0.94 to 1.13)	Vitamin B6/ Intake	High vs. Low	Cancer	29	RR 0.95 (0.90 to 1.01)	Yes
Morze 2021 [233]	Mediterranean diet/ Intake	High vs. Low adherence	Cancer mortality	1	RR 0.75 (0.17 to 3.33)	Mediterranean diet/ Intake	High vs. Low adherence	Cancer mortality	18	RR 0.87 (0.82 to 0.92)	Yes
Morze 2021 [233]	Mediterranean diet/ Intake	High vs. Low adherence	Breast cancer	1	RR 0.41 (0.19 to 0.87)	Mediterranean diet/ Intake	High vs. Low adherence	Breast cancer	12	RR 0.97 (0.94 to 1.00)	Yes
Picasso 2019 [256]	Vegetarian diet/ Intake	Vegetarian vs. Omnivore diet	Triglycerides	5	MD 0.11 (-0.10 to 0.31)	Vegetarian diet/ Intake	Vegetarian vs. Omnivore diet	Triglycerides	1	MD 0.07 (-0.39 to 0.53)	Yes
Picasso 2019 [256]	Vegetarian diet/ Intake	Vegetarian vs. Omnivore diet	HDL-cholesterol	4	MD -0.06 (-0.17 to 0.04)	Vegetarian diet/ Intake	Vegetarian vs. Omnivore diet	HDL-cholesterol	1	MD -0.03 (-0.10 to 0.04)	Yes

Sayehmiri 2018 [257]	Selenium/ Supplementation	High vs. Low	Prostate cancer	9	RR 0.90 (0.74 to 1.09)	Selenium/ Supplementation + Intake	High vs. Low	Prostate cancer	2	RR 0.89 (0.64 to 1.23)	Yes
Schwingshackl 2015 [237]	Mediterranean diet/ Intake	High vs. Low adherence	Type 2 diabetes	1	RR 0.70 (0.54 to 0.91)	Mediterranean diet/ Intake	High vs. Low adherence	Type 2 diabetes	8	RR 0.83 (0.74 to 0.92)	Yes
Schwingshackl 2017 [258]	Olive oil/ Intake	High vs. Low	Type 2 diabetes	1	RR 0.60 (0.43 to 0.84)	Olive oil/ Intake	High vs. Low	Type 2 diabetes	4	RR 0.87 (0.80 to 0.94)	Yes
Setien-Suero 2016 [259]	Homocysteine/ Supplementation	Low vs. High	Mini Mental State Examination Score	2	SMD 0.05 (-0.07 to 0.17)	Homocysteine/ Status	Correlation	Mini Mental State Examination Score	3	ß -0.11 (-0.18 to -0.05)	No
Stratton 2011 [224]	Multivitamins/ Supplementation	High vs. Low	Prostate cancer	1	OR 0.90 (0.61 to 1.33)	Multivitamins/ Supplementation	High vs. Low	Prostate cancer	1	OR 1.05 (0.94 to 1.16)	Yes
Stratton 2011 [224]	Vitamin E/ Supplementation	High vs. Low	Prostate cancer	3	NR	Vitamin E/ Supplementation	High vs. Low	Prostate cancer	5	NR	Yes
Stratton 2011 [224]	Vitamin C/ Supplementation	High vs. Low	Prostate cancer	1	HR 1.01 (0.92 to 1.10)	Vitamin C/ Supplementation	High vs. Low	Prostate cancer	1	NR	Yes
Stratton 2011 [224]	β-carotene/ Supplementation	High vs. Low	Prostate cancer	1	OR 1.20 (0.86 to 1.66)	β-carotene/ Supplementation	High vs. Low	Prostate cancer	1	OR 0.69 (0.62 to 0.77)	Yes
Te Morenga 2013 [260]	Dietary sugars/ Intake	High vs. Low	Weight gain	10	MD 0.75 (0.30 to 1.19)	Dietary sugars/ Intake	High vs. Low	Weight gain	4	MD 0.31 (-0.07 to 0.68)	Yes
Te Morenga 2013 [260]	Dietary sugars/ Intake	Low vs. High	Body mass index (change)	3	SMD 0.09 (-0.14 to 0.32)	Dietary sugars/ Intake	Dose-response; per serving/day or increase of 100g/day SSB intake	Body mass index (change)	4	MD 0.02 (0.00 to 0.05)	Yes
Thorne-Lyman 2012 [225]	Vitamin D/ Supplementation	High vs. Low	Mean birthweight	5	MD 58.07 (-63.87 to 180.00)	Vitamin D/ Supplementation + Intake	High vs. Low	Mean birthweight	2	MD 52.66 (3.54 to 101.77)	Yes

Trikalinos 2012 [239]	Vitamin E/ Supplementation	High vs. Low	Cardiovascular disease	14	RR 0.97 (0.91 to 1.03)	Vitamin E/ Supplementation + Intake	High vs. Low	Cardiovascular disease	8	HR 0.85 (0.78 to 0.93)	Yes
Vinceti 2018a [7]	Selenium/ Supplementation	High vs. Low	Any cancer	5	RR 0.99 (0.86 to 1.14)	Selenium/ Status	High vs. Low	Any cancer	7	OR 0.72 (0.55 to 0.93)	Yes
Vinceti 2018a [7]	Selenium/ Supplementation	High vs. Low	Cancer mortality	2	RR 0.81 (0.49 to 1.32)	Selenium/ Intake	High vs. Low	Cancer mortality	1	OR 0.93 (0.83 to 1.04)	Yes
Vinceti 2018b [236]	Selenium/ Supplementation	High vs. Low	Type 2 diabetes	5	RR 1.11 (1.01 to 1.22)	Selenium/ Intake	High vs. Low	Type 2 diabetes	1	OR 2.39 (1.32 to 4.32)	Yes
Wien 2012 [226]	Folic acid/ Supplementation	High vs. Low	Cancer	10	RR 1.07 (1.00 to 1.14)	Folic acid/ Supplementation	High vs. Low	Cancer	5	RR 1.03 (0.90 to 1.18)	Yes
Wolf 2017 [8]	Multivitamins/ Supplementation	High vs. Low	Preterm birth	1	RR 1.09 (0.43 to 2.77)	Multivitamins/ Supplementation	High vs. Low	Preterm birth	4	RR 0.84 (0.69 to 1.03)	Yes
Wolf 2017 [8]	Multivitamins/ Supplementation	High vs. Low	Stillbirth	1	RR 2.43 (0.12 to 50.05)	Multivitamins/ Supplementation	High vs. Low	Stillbirth	2	RR 0.78 (0.59 to 1.03)	Yes
Yang 2016 [235]	Folic acid/ Supplementation	High vs. Low	Gestational hypertension	1	RR 0.62 (0.40 to 0.94)	Folic acid/ Supplementation + Intake	High vs. Low	Gestational hypertension	3	RR 1.02 (0.95 to 1.09)	Yes
Yang 2020 [227]	Calcium/ Supplementation	High vs. Low	Cardiovascular disease	4	RR 0.91 (0.56 to 1.46)	Calcium/ Intake	High vs. Low	Cardiovascular disease	13	RR 0.98 (0.90 to 1.06)	Yes
Yao 2019 [228]	Vitamin D/ Supplementation	High vs. Low	Any fracture	11	Rate Ratio 1.06 (0.98 to 1.14)	Vitamin D/ Status	Dose-response; per 25nmol/l increase in 25(OH)D level in blood	Any fracture	10	Rate Ratio 0.93 (0.89 to 0.96)	Yes
Yu 2021 [229]	Folic acid/ Supplementation	High vs. Low	Pre-eclampsia	3	RR 1.11 (0.75 to 1.62)	Folic acid/ Supplementation + Intake	High vs. Low	Pre-eclampsia	13	RR 0.85 (0.76 to 0.94)	Yes

Zhang 2016 [9]	Selenium/ Supplementation	High vs. Low	Cardiovascular disease	9	RR 0.91 (0.74 to 1.10)	Selenium/ Status	High vs. Low	Cardiovascular disease	14	RR 0.87 (0.76 to 0.99)	Yes
Zhang 2018 [230]	Vitamin D/ Supplementation	High vs. Low	Gestational diabetes	4	RR 0.72 (0.39 to 1.31)	Vitamin D/ Status	Low vs. High	Gestational diabetes	24	OR 1.82 (1.36 to 2.44)	Yes
Zhang 2018 [230]	Vitamin D/ Supplementation	High vs. Low	Fasting plasma glucose	16	SMD -0.43 (-0.67 to -0.18)	Vitamin D/ Status	Correlation	Fasting plasma glucose	13	CC -0.09 (-0.20 to 0.01)	No
Zhao 2014 [261]	Multivitamins/ Minerals/ Supplementation	High vs. Low	Nuclear cataract	2	RR 0.73 (0.59 to 0.90)	Multivitamins/ Minerals/ Supplementation	High vs. Low	Nuclear cataract	5	RR 0.73 (0.64 to 0.82)	Yes
Zhao 2014 [261]	Multivitamins/ Minerals/ Supplementation	High vs. Low	Cortical cataract	2	RR 0.92 (0.69 to 1.23)	Multivitamins/ Minerals/ Supplementation	High vs. Low	Cortical cataract	4	RR 0.81 (0.68 to 0.94)	Yes
Zhao 2014 [261]	Multivitamins/ Minerals/ Supplementation	High vs. Low	Posterior subcapsular cataract	2	RR 1.81 (1.30 to 2.54)	Multivitamins/ Minerals/ Supplementation	High vs. Low	Posterior subcapsular cataract	3	RR 0.96 (0.72 to 1.20)	Yes
Zhou 2017 [232]	Vitamin D/ Supplementation	High vs. Low	Preterm birth	5	OR 0.57 (0.36 to 0.91)	Vitamin D/ Status	Low vs. High	Preterm birth	12	OR 1.14 (0.95 to 1.38)	Yes

BoE=bodies of evidence; CC=correlation coefficient; CI=confidence interval; HR=hazard ratio; MD=mean difference; NR=not reported; OR=odds ratio; RR=risk ratio; SMD=standardised mean difference; SSB=sugar sweetened beverages; WGMC=weighted group mean correlation; WGMD=weighted group mean difference

Supplementary Table S5 Overview of transformations made to the original data extraction

Systematic review	Intervention/ Exposure	Outcome	Type of BoE (RCTs/ CSs)	n (studies)	Original		What we used		Rationale
					**HR**	**95% CI**	**RR**	**95% CI**	
Johnston 2019 [247]	Low red meat	Cardiovascular disease	RCT	1	0.98	0.91 to 1.06	1.00	0.84 to 1.19	Number of patients and events in intervention and control group for coronary heart disease death and fatal stroke, mean 8.1 years follow-up; Table 4 in Howard 2006 [262]
Johnston 2019 [247]	Low red meat	Colorectal cancer	RCT	1	1.04	0.90 to 1.20	1.02	0.89 to 1.18	Number of patients and events in intervention and control group for colorectal cancer, mean 5 years follow-up; Supplementary Table 2 in Thomson 2014 [263]
Johnston 2019 [247]	Low red meat	Type 2 diabetes	RCT	1	0.96	0.90 to 1.03	0.95	0.89 to 1.02	Number of patients and events in intervention and control group for type 2 diabetes, mean 8.1 years follow-up; Figure 2 in Tinker 2008 [264]
Stratton 2011 [224]	Vitamin C	Prostate cancer	RCT	1	1.01	0.92 to 1.10	1.10	0.93 to 1.29	Number of patients and events in intervention and control group for prostate cancer, mean 8 years follow-up; Figure 3 in Gaziano 2009 [265]
Trikalinos 2012 [239]	Vitamin E	Cardiovascular disease	CS	8	0.85	0.78 to 0.93	0.85	0.78 to 0.93	Data not available to calculate a RR for the HR; assumption HR=RR
					**logHR**	**95% CI**	**RR**	**95% CI**	
Kastorini 2011 [6]	Mediterranean diet	Metabolic syndrome	RCT	2	-0.80	-1.42 to -0.18	0.45	0.24 to 0.84	Meta-analysis of RCTs (clinical trials) shown in Figure 2; HRs pooled with inverse-variance method; data not available to calculate a RR for the HR; assumption HR=RR

			CS	2	-0.76	-2.16 to 0.63	0.46	0.11 to 1.88	Meta-analysis of CSs (prospective studies) shown in Figure 2; HRs pooled using inverse-variance models with random effects; data not available to calculate a RR for the HR; assumption HR=RR
					**OR**	**95% CI**	**RR**	**95% CI**	
Aguilar-Cordero 2020 [218]	Vitamin D	Pre-eclampsia	RCT	7	0.61	0.36 to 1.04	0.66	0.42 to 1.05	Meta-analysis of RCTs (n=7) shown in Figure 27; RRs calculated using number of patients and events in intervention and control groups; pooled using inverse-variance models with random effects
			CS	7	1.47	1.29 to 1.67	0.82	0.60 to 1.11	Meta-analysis of observational studies (n=11) shown in Figure 4; prospective studies were separated (Achkar 2015 [266], Arisoy 2015 [267], Bener 2013 [268], Burris 2014 [269], Gidlöf 2015 [270], Schneuer 2014 [271], van Weert 2016 [272]); ORs inverted (from low vs. high to high vs. low); ORs converted to RR with ACR= 0.10; risk observed in the control group of the corresponding BoE of RCTs (Figure 24); pooled using inverse-variance models with random effects
Chung 2011 [242]	Vitamin D	Colorectal cancer	CS	9	0.94	0.91 to 0.97	0.94	0.91 to 0.97	ACR= 0.0201; risk observed in the control group of the corresponding BoE of RCTs (Trivedi 2003 [273], colorectal cancer)
Chung 2011 [242]	Vitamin D	Breast cancer	CS	4	0.99	0.97 to 1.01	0.99	0.97 to 1.01	ACR= 0.0124; risk observed in the control group of the corresponding BoE of RCTs (Trivedi 2003 [273], breast cancer)

Feng 2015 [4]	Folic acid	Congenital heart defects	RCT	1	0.42	0.19 to 0.98	0.58	0.33 to 1.02	RR calculated using number of patients and events in intervention and control group for cardiovascular defects; Table 4 in Czeizel 1998 [274]; converted to a standardized food portion of the corresponding CS (from 0.8mg/day to 0.6mg/day)
			CS	1	0.60	0.38 to 0.96	0.60	0.38 to 0.96	ACR= 0.0084; risk observed in the control group of the corresponding BoE of RCTs (Czeizel 1998 [274], cardiovascular defects)
Jonker 2020 [248]	Folic acid	Low birthweight	RCT	1	0.93	0.81 to 1.08	0.96	0.88 to 1.04	Meta-analysis shown in Figure 4; RR calculated using number of patients and events in intervention and control group of Christian 2003 [275]
			CS	5	0.55	0.44 to 0.70	0.71	0.60 to 0.83	Meta-analysis for non-randomised studies (n=7) in Figure 4; prospective cohorts were separated (Balarajan 2013 [276], Chaudhary 2012 [277], Joseph 2011 [278], Nisar 2014 [279], Wang 2016 [280]); OR converted to RR with ACR= 0.4341; risk observed in the control group of the corresponding BoE of RCTs (Christian 2003 [275])
Jonker 2020 [248]	Folic acid	Small for gestational age	RCT	1	0.96	0.81 to 1.14	0.98	0.92 to 1.06	Meta-analysis shown in Figure 6; RR calculated using number of patients and events in intervention and control group of Christian 2003 [275]
			CS	4	0.66	0.41 to 1.07	0.80	0.50 to 1.11	Meta-analysis for non-randomised studies (n=4) in Figure 4; we combined post-conceptional and pre-conceptional folic acid use in Zheng 2016 [281]; OR converted to RR with ACR= 0.5870; risk observed in the control group of the corresponding BoE of RCTs Christian 2003 [275]

Stratton 2011 [224]	Multivitamins	Prostate cancer	RCT	1	0.90	0.61 to 1.33	0.90	0.62 to 1.33	Meta-analysis shown in Figure 2a; RR calculated using number of patients and events in intervention and control group of Meyer 2005 [282]
			CS	1	1.05	0.94 to 1.16	1.05	0.94 to 1.16	ACR= 0.0215; risk observed in the control group of the corresponding BoE of RCTs (Meyer 2005 [282], Occurrence of prostate cancer)
Stratton 2011 [224]	β-carotene	Prostate cancer	RCT	1	1.20	0.86 to 1.66	1.19	0.87 to 1.65	Meta-analysis shown in Figure 2E; RR calculated using number of patients and events in intervention and control group of Heinonen 1998 [283]
			CS	1	0.69	0.62 to 0.77	0.69	0.63 to 0.77	ACR= 0.0092; risk observed in the control group of the corresponding BoE of RCTs (Heinonen 1998 [283]); Figure 2E
Stratton 2011 [224]	Vitamin E	Prostate cancer	RCT	3	1.02	0.89 to 1.16	0.98	0.81 to 1.20	Meta-analysis for all study types (Figure 2B) was separated; effect estimates for RCTs and CS were converted from OR to RR; pooled using inverse-variance models with random effects
			CS	5			1.09	0.94 to 1.27	
Vinceti 2018a [7]	Selenium	Any cancer	CS	7	0.72	0.55 to 0.93	0.75	0.59 to 0.94	ACR= 0.1509; risk observed in the control group of the corresponding BoE of RCTs; Analysis 1.1., Outcome 1 Any cancer risk
Vinceti 2018a [7]	Selenium	Cancer mortality	CS	1	0.93	0.82 to 1.04	0.93	0.83 to 1.04	ACR= 0.0597; risk observed in the control group of the corresponding BoE of RCTs; Analysis 1.2., Outcome 2 Cancer mortality
Vinceti 2018b [236]	Selenium	Type 2 diabetes	CS	1	2.39	1.32 to 4.32	2.28	1.31 to 3.89	ACR= 0.0335; risk observed in the control group of the corresponding BoE of RCTs; Table 2

Zhang 2018 [230]	Vitamin D	Gestational diabetes	CS	24	1.85	1.47 to 2.32	0.58	0.44 to 0.76	Meta-analysis for observational studies (n=38) in Figure 1; we included only prospective cohort (Alvarez-Silvares 2016 [284], Burris 2011 [285], Burris 2012 [286], Davies-Tuck 2013 [287], Davies-Tuck 2015 [288], Fernandez-Alonso 2010 [289], Hu 2015 [290], Inancli 2016 [291], Kramer 2014 [292], Loy 2015 [293], Park 2014 [294], Song 2015 [295], Tomedi 2013 [296], Zhou 2014 [297], Zhu 2015 [298]) and nested-case-control studies (Arnold 2015 [299], Baker 2012 [300], Dodds 2013 [301], Dodds 2016 [302], Giguere 2014 [303], Li 2016 [304] Makgoba 2011 [305], Schneuer 2014 [271], Zhang 2008 [306]); ORs were inverted (from low vs. high to high vs. low); ORs were pooled using inverse-variance models with random effects; ACR= 0,1060; risk observed in the control group of the corresponding BoE of RCTs (Hossain 2014 [307], Mojibian 2015 [308], Sablok 2015 [309], Yap 2014 [310]; Figure S19
Zhou 2017 [232]	Vitamin D	Preterm birth	RCT	5	0.57	0.36 to 0.91	0.58	0.30 to 1.11	Meta-analysis for RCTs (n=6) in Figure 3; we excluded Asemi 2013 [311] due to retraction; RRs calculated using number of patients and events in intervention and control groups; pooled using inverse-variance models with random effects

			CS	12	1.25	1.13 to 1.38	0.88	0.73 to 1.05	Meta-analysis of observational studies (n=14) in Supplementary Figure 2; we separated the studies of the prospective, case-cohort and nested case-cohort subgroups; ORs were inverted (from low vs. high to high vs. low); ORs were pooled using inverse-variance models with random effects; ACR= 0.0460; risk observed in the control group of the corresponding BoE of RCTs, Figure 3
					**Rate Ratio**	**95% CI**	**RR**	**95% CI**	
Yao 2019 [228]	Vitamin D	Any fracture	RCT	11	1.06	0.98 to 1.14	1.06	0.97 to 1.15	Meta-analysis for RCTs (n=11) in Figure 2, Any fracture; RRs calculated using number of patients and events in intervention and control groups; pooled using inverse-variance models with random effects
			CS	10	0.93	0.89 to 0.96	0.89	0.83 to 0.96	Meta-analysis for CSs (n=10) in Figure 1, Any fracture; RRs calculated using number of patients and events in intervention and control groups; pooled using inverse-variance models with random effects
					**RR**	**95% CI**	**RR**	**95% CI**	
Aburto 2013 [2]	Low sodium	All-cause mortality	CS	2	1.06	0.94 to 1.20	0.95	0.71 to 1.27	Meta-analysis of CSs (n=7) shown in Figure 3.20 [312]; we included only CSs reporting on effect of dietary intake (Cohen 2008 [313], He 1999 [314]); RRs were inverted (from high vs. low to low vs. high); pooled using inverse-variance models with random effects

Aburto 2013 [2]	Low sodium	Cardiovascular disease	CS	3	1.12	0.93 to 1.34	0.87	0.64 to 1.18	Meta-analysis of CSs (n=9) shown in Figure 3.4 [312]; we included only CSs reporting on effect of dietary intake (Cohen 2008 [313], He 1999 [314], Umesawa 2008 [315]); RRs were inverted (from high vs. low to low vs. high); pooled using inverse-variance models with random effects
Afshin 2014 [240]	Nuts	Coronary heart disease	RCT	1	Fatal: 0.76<br>Nonfatal: 0.78	0.69 to 0.84<br>0.67 to 0.92	0.79	0.55 to 1.14	Meta-analysis for RCTs and CSs (Figure 2A and 2B; Fatal and nonfatal CHD) was separated; RRs for CSs with nonfatal and fatal outcomes were pooled using inverse-variance models with random effects
			CS	6			0.76	0.69 to 0.83	
Afshin 2014 [240]	Nuts	Stroke	RCT	1	0.89	0.74 to 1.05	0.62	0.44 to 0.87	Meta-analysis for RCTs and CSs (Figure 2C; Total stroke) was separated; RRs for CS were pooled using inverse-variance models with random effects
			CS	2			0.95	0.83 to 1.09	
Afshin 2014 [240]	Nuts	Type 2 diabetes	RCT	1	0.87	0.81 to 0.94	0.86	0.68 to 1.08	Meta-analysis for RCTs and CSs (Figure 2D; diabetes) was separated; RRs for CS were pooled using inverse-variance models with random effects
			CS	4			0.87	0.80 to 0.95	
Chowdhury 2014b	Vitamin D	All-cause mortality	CS	68	1.44	1.34 to 1.55	0.69	0.65 to 0.75	Value of RR and 95% CI displayed in Figure 1, All-cause mortality was inverted (from low vs. high to high vs. low).
Kim 2018 [223]	Multivitamins/ Minerals	Cardiovascular disease	RCT	1	1.00	0.97 to 1.04	0.95	0.83 to 1.09	Meta-analysis for RCTs and CSs (Figure 2; CVD mortality) was separated; RRs for CS were pooled using inverse-variance models with random effects
			CS	9			1.00	0.96 to 1.05	
Kim 2018 [223]	Multivitamins/ Minerals	Stroke	RCT	1	0.98	0.91 to 1.05	1.05	0.70 to 1.56	Meta-analysis for RCTs and CSs (Figure 2; Stroke Incidence) was separated; RRs for CS were pooled using inverse-variance models with random effects
			CS	3			0.98	0.91 to 1.04	

Lin 2020 [251]	Vitamin D	Nephrolithiasis	CS	3	1.22	1.01 to 1.49	1.24	1.01 to 1.51	Meta-analysis for observational studies (Figure 5, Total Vitamin D, n=4); prospective studies were separated (NHS I [316], NHS II [317], HPFS [317]; RRs pooled using inverse-variance models with random effects
Sayehmiri 2018 [257]	Selenium	Prostate cancer	CS	2	0.77	0.52 to 1.14	0.89	0.64 to 1.23	Meta-analysis of CSs (n=9) shown in Table 2, Type of study; we included only CSs reporting on effect of dietary intake (Hartman 1998 [318], Peters 2008 [319]; RRs pooled using inverse-variance models with random effects
Stratton 2011 [224]	Vitamin C	Prostate cancer	CS	1	NR	NR	0.96	0.86 to 1.06	Number of patients and events in intervention and control group for prostate cancer reported in Table 2
Wien 2012 [226]	Folic acid	Cancer	CS	5	1.03	0.92 to 1.16	1.03	0.90 to 1.18	Meta-analysis of CSs (n=6); we included only prospective CSs (Maruti 2009 [320], Oaks 2010 [321], Skinner 2004 [322], Slatore 2008 [323], Stolzenberg-Solomon 2006 [324]); RRs pooled using inverse-variance models with random effects
Wolf 2017 [8]	Multivitamins	Preterm birth	RCT	1	NR	NR	1.09	0.43 to 2.77	Number of patients and events in intervention and control group for preterm birth; Table 4 in Brough 2010 [325]
Wolf 2017 [8]	Multivitamins	Stillbirth	RCT	1	NR	NR	2.43	0.12 to 50.05	Number of patients and events in intervention and control group for preterm birth; Table 2 in Kirke 1992 [326]
Yang 2016 [235]	Folic acid	Gestational hypertension	RCT	1	1.02	1.00 to 1.04	0.62	0.40 to 0.94	Meta-analysis for RCTs and CSs (Figure 2) was separated; we included only prospective CSs reporting on postconceptional folic acid use (Li 2013 [327], Oken 2007 [328], Timmermans (Postcon) 2011 [329]; RRs of CSs were pooled using inverse-variance models with random effects
			CS	3			1.05	1.00 to 1.11	

Yu 2021 [229]	Folic acid	Pre-eclampsia	CSs	13	0.83	0.74 to 0.93	0.85	0.76 to 0.94	Meta-analysis for all study types (Figure 3); we included only prospective CSs (Bodnar 2006 [330], Bukowski 2009 [331], Catov 2009 [332], Catov 2011 [333], Li 2020 [334], Liu 2015 [335], Martinussen 2015 [336], Oken 2007 [328], Timmermanns 2011 [329], Vanderlelie 2014 [337], Wang 2015 [338], Wen 2008 [339], Wen 2016 [340]; RRs pooled using inverse-variance models with random effects
Zhao 2014 [261]	Multivitamins/ Minerals	Nuclear cataract	RCT	2	NR	NR	0.73	0.59 to 0.90	RRs of reported RCTs (Maraini 2008 [341], Sperduto 1993 [342]) were pooled using inverse-variance models with random effects
Zhao 2014 [261]	Multivitamins/ Minerals	Cortical cataract	RCT	2	NR	NR	0.92	0.69 to 1.23	RRs of reported RCTs (Maraini 2008 [341], Sperduto 1993 [342]) were pooled using inverse-variance models with random effects
Zhao 2014 [261]	Multivitamins/ Minerals	Posterior subcapsular cataract	RCT	2	NR	NR	1.81	1.30 to 2.54	RRs of reported RCTs (Maraini 2008 [341], Sperduto 1993 [342]) were pooled using inverse-variance models with random effects
					**MD**	**95% CI**	**MD**	**95% CI**	
Amegah 2017 [231]	Vitamin D	Gestational length	RCT	2	-0.24	-0.69 to 0.22	0.16	-0.28 to 0.59	Meta-analysis for RCTs and CS (Figure 3C) was separated; MDs were inverted (from low vs. high to high vs. low)
			CS	1			0.08	0.20 to 1.40	
Kastorini 2011 [6]	Mediterranean diet	HDL-cholesterol	RCT	27	1.17	0.38 to 1.96	0.03	0.01 to 0.05	Meta-analyses for clinical trials shown in Table 4; we excluded non-randomised studies (Papadaki 2008 [343], Gomez 2005 [344]); MD was converted from mg/dl to mmol/l (factor: 0.026)
			CS	1	0.30	-1.77 to 2.37	0.01	-0.05 to 0.06	MD was converted from mg/dl to mmol/l (factor: 0.026)

Kastorini 2011 [6]	Mediterranean diet	Triglycerides	RCT	27	-6.14	-10.35 to 1.93	-0.07	-0.12 to -0.02	Meta-analyses for clinical trials shown in Table 4; we excluded non-randomised studies (Papadaki 2008 [343], Gomez 2005 [344]); MD was converted from mg/dl to mmol/l (factor: 0.0114)
			CS	1	-2.00	-6.74 to 2.74	-0.02	-0.08 to 0.03	MD was converted from mg/dl to mmol/l (factor: 0.0114)
Te Morenga 2013 [260]	Low dietary sugars	Weight gain	RCT	10	0.75	0.30 to 1.19	-0.75	-1.19 to 0.30	Meta-analysis of RCTs shown in Figure 4; MD was inverted (from high vs. low to low vs. high)
			CS	4	0.31	-0.07 to 0.68	-0.31	-0.68 to 0.07	Meta-analysis of CS (n=4) shown in Web figure 3 (Weight difference between highest and lowest intakes at baseline); MD inverted (from high vs. low to low vs. high)
Te Morenga 2013 [260]	Low dietary sugars	Body mass index	CS	4	0.02	-0.00 to 0.05	-0.02	-0.05 to -0.00	Meta-analysis of CS (n=4) shown in Web figure 6; MD inverted (from high vs. low to low vs. high)
					**MD**	**95% CI**	**SMD**	**95% CI**	
Azad 2017 [220]	Nonnutritive sweeteners	Body mass index	RCT	3	-0.37	-1.10 to 0.36	-0.14	-0.43 to 0.16	Meta-analysis for RCTs shown in Figure 2A using MD; we converted MD to SMD using mean, SD and number of patients in intervention and control groups, pooled using inverse-variance models with random effects
			CS	1	0.77	0.47 to 1.07	-0.27	0.17 to 0.38	CS of Fowler 2008 [345], described in results for body mass index; MD was standardised using mean, SD and number of patients in exposure and comparator groups
Jonker 2020 [248]	Folic acid	Mean birthweight	RCT	3	0.56	0.15 to 0.97	0.20	0.08 to 0.31	Meta-analysis of RCTs shown in Figure 2; MD was standardised using mean, SD and number of patients in intervention and control groups, pooled using inverse-variance models with random effects

Picasso 2019 [256]	Vegetarian diet	Triglycerides	RCT	5	0.11	-0.10 to 0.31	0.08	-0.07 to 0.23	Meta-analysis of RCTs shown in Figure 6, Analysis 5.1.1; MD was standardised using mean, SD and number of patients in intervention and control groups, pooled using inverse-variance models with random effects
Picasso 2019 [256]	Vegetarian diet	HDL-cholesterol	RCT	4	-0.06	-0.17 to 0.04	-0.13	-0.29 to 0.04	Meta-analysis of RCTs shown in Figure 8, Analysis 7.1.1; MD was standardised using mean, SD and number of patients in intervention and control groups, pooled using inverse-variance models with random effects
					**SMD**	**95% CI**	**MD**	**95% CI**	
Te Morenga 2013 [260]	Low dietary sugars	Body mass index	RCT	3	0.09	-0.14 to 0.32	-0.06	-0.15 to 0.04	Meta-analysis of RCTs (n=5) shown in Figure 6 with SMDs; we included only RCTs reporting the outcome body mass index (Ebbeling 2006 [346], James 2004 [347], Paineau 2008 [348]; MDs were calculated using mean, SD and number of patients in intervention and control groups; pooled using inverse-variance models with random effects
					**SMD**	**95% CI**	**SMD & MD**	**95% CI**	
Jonker 2020 [248]	Folic acid	Mean birthweight	CS	2	0.31	0.18 to 0.43	SMD 0.36<br>MD 0.14	0.08 to 0.65<br>0.05 to 0.24	Meta-analysis of non-randomised studies shown in Figure 2; we included only the two prospective studies on folic acid supplementation (Chaudhary 2012 [277], Wang 2016 [280]; MD and SMDs calculated and pooled using inverse-variance models with random effects

					MD	95% CI	MD & SMD	95% CI	
Picasso 2019 [256]	Vegetarian diet	Triglycerides	CS	2	0.06	-0.22 to 0.35	MD 0.07<br><br>SMD 0.03	-0.39 to 0.53<br><br>-0.19 to 0.26	Meta-analysis of CSs shown in Figure 6, Analysis 5.1.2; we included only the prospective cohort study (Brestrich 1996 [349]; MD was standardised using mean, SD and number of patients in intervention and control group, pooled using inverse-variance models with random effects
Picasso 2019 [256]	Vegetarian diet	HDL-cholesterol	CS	1	0.16	-0.24 to 0.55	MD -0.03<br><br>SMD -0.09	-0.10 to 0.04<br><br>-0.32 to 0.13	Meta-analysis of CSs shown in Figure 8, Analysis 7.1.2; we included only the prospective cohort study (Brestrich 1996 [349]; MD was standardised using mean, SD and number of patients in intervention and control group, pooled using inverse-variance models with random effects
Thorne-Lyman 2012 [225]	Vitamin D	Mean birthweight	RCT	5	58.07	-63.87 to 180.00	MD 0.06<br><br>SMD 0.13	-0.06 to 0.18<br><br>-0.12 to 0.39	Meta-analysis of observational studies shown in Figure S1 (Bolus or daily dosing and mean birthweight); MD was converted from gram to kilogram; MD was standardised using mean, SD and number of patients in intervention and control groups, pooled using inverse-variance models with random effects
			CS	2	52.66	3.54 to 101.77	MD 0.05<br><br>SMD 0.11	0.003 to 0.10<br><br>0.01 to 0.21	Meta-analysis of observational studies shown in Figure S1 (Observational studies of intake); MD was converted from grams to kilograms; MD was standardised using mean, SD and number of patients in intervention and control groups, pooled using inverse-variance models with random effects
					**Correlation**				
Miller 2014 [253]	Low calorie sweeteners	Body weight	CS	4	0.02	-0.01 to 0.06	-	-	Impossibility to convert weighted group mean correlation (CSs) to weighted group MD (RCTs)

Setien-Suero 2016 [259]	Homocysteine	Mini Mental State Examination Score	CS	3	-0.11	-0.18 to -0.05	-	-	Impossibility to convert β (CSs) to SMD (RCTs)
Zhang 2018 [230]	Vitamin D	Fasting plasma glucose	CS	13	-0.09	-0.20 to 0.01	-	-	Impossibility to convert correlation coefficient (CSs) to SMD (RCTs)

ACR=assumed control risk; BoE=bodies of evidence; CHD=coronary heart disease; CI=confidence interval; CS=cohort study; CVD=cardiovascular disease; HDL=high density lipoprotein; HPFS=Health Professionals Follow-Up Study; HR=hazard ratio; MD=mean difference; NHS=Nurses' Health Study; OR=odds ratio; RCT=randomised controlled trial; RR=risk ratio; SD=standard deviation; SMD=standardised mean difference

Supplementary Table S6 Characteristics of included BoE from randomised controlled trials

Reference	Intervention	Outcome	Studies, n	Sample size, n	Cases, n	Description of population	Age (years)	Description of intervention	Description of comparator	Description of outcome	Study design	Follow-up
Aburto 2013 [2]	Low sodium	All-cause mortality	4	3,595	69	Male and female population/ mostly healthy with normal blood pressure (3x); partly with hypertension (1x)/ partly moderate overweight (1x)	20-54 (range)	Dietary and behavioural counselling to reduce sodium intake/ partly with weight loss intervention (1x)	No or unspecific counselling/ usual care (1x); no intervention (1x) or counselling for generally healthy diet (1x); weight loss alone (1x)	All-cause mortality	Parallel, factorial	0.5-3 years
Aburto 2013 [2]	Low sodium	Cardiovascular disease	2	720	93	Male and female population/ with hypertension, but otherwise healthy/ partly obese	66.5 (mean) or >50	Dietary and behavioural counselling to reduce sodium intake	No or unspecific counselling/ general advice on subjects unrelated to CVD, blood pressure, nutrition (1x); no intervention (1x)	Cardiovascular disease	Parallel, factorial	2-2.5 years
Afshin 2014 [240]	Nuts	Coronary heart disease	1	7,447	69	Male and female population/ without CVD, but with either T2D or ≥3 major cardiovascular risk factors	55-80 (range); 66.7-67.3 (mean)	Nut consumption/ intake of tree nuts (e.g. walnut, almond, hazelnuts, pecan, cashew, and pistachio) and peanuts/ dose-response: per 4 weekly servings (28.4g)	Dose-response	Total incident ischemic heart disease (fatal and nonfatal)	Parallel	4.8 years
Afshin 2014 [240]	Nuts	Stroke	1	7,447	90	Male and female population/ without CVD, but with either T2D or ≥3 major cardiovascular risk factors	55-80 (range); 66.7-67.3 (mean)	Nut consumption/ intake of tree nuts (e.g. walnut, almond, hazelnuts, pecan, cashew, and pistachio) and peanuts/ dose-response: per 4 weekly servings (28.4g)	Dose-response	Total stroke (ischemic and haemorrhagic)	Parallel	4.8 years
Afshin 2014 [240]	Nuts	Type 2 diabetes	1	3,541	273	Male and female population/ without CVD and T2D, but with ≥3 major cardiovascular risk factors	55-80 (range); 66.2-67.2 (mean)	Nut consumption/ intake of tree nuts (e.g. walnut, almond, hazelnuts, pecan, cashew, and pistachio) and peanuts/ dose-response: per 4 weekly servings (28.4g)	Dose-response	Type 2 diabetes	Parallel	4.1 years
Aguilar-Cordero 2020 [218]	Vitamin D	Pre-eclampsia	7	1,660	160	Female population/ pregnant/ without specific previous pathologies	24.8-32.4 (mean)	Vitamin D supplementation/ 600-4,400IU/day or 50,000IU every 2 weeks (2x) or single dose of 60,000IU or 2-4 doses of 120,000IU	No or low vitamin D supplementation/ placebo (2x); routine care (2x); low vitamin D (400IU/day; 2x); no intervention (1x)	Gestational hypertension with proteinuria and/or other maternal organ dysfunction and/or utero-placental dysfunction/ ≥20 weeks of gestation	Parallel	NR
Alexander 2017 [219]	Omega-3 fatty acids	Coronary heart disease	18	93,633	NR	Male and female population/ with or without CHD/ partly with low left ventricular systolic function or T2D or peripheral arterial disease (3x)/ non-hospitalised	>18	EPA+DHA intake or supplementation/ food, ethyl esters, fish oil, fatty fish or EPA+DHA enriched margarine/ 0.38-5.04g/day	Mostly oils not containing EPA+DHA (olive, corn or sunflower oil, 11x)/ partly gelatine (1x); aluminium hydroxide (1x); dietary advice (2x); no supplementation (2x)	Fatal and nonfatal myocardial infarction, coronary death, sudden cardiac death, angina	Parallel	0.5-7 years

Amegah 2017 [231]	Vitamin D	Gestational length	2	525	N/A	Female population/ pregnant/ with singleton pregnancy	25.19-27.4 (mean)	Vitamin D supplementation/ 2,000 or 4,000IU/day/ partly with 400mg/day ferrous sulfate and 600mg/day calcium	No or low vitamin D supplementation/ 400IU/day (1x); routine care with 400mg/day ferrous sulfate and 600mg/day calcium (1x)	Gestational length (weeks)	Parallel	≤16 or ≤20 weeks of gestation till delivery
Azad 2017 [220]	Nonnutritive sweeteners	Body mass index	3	242	N/A	Male and female population/ with mild hypertension (2x); overweight (1x), but otherwise healthy (3x)	32-52 (mean)	Stevioside capsules: 3.8-15mg/kg per day (1x); 1,500mg/day (1x); 250ml/day artificially sweetened beverage (1x)	Placebo (2x); water (1x)	Change in body mass index (kg/m^2) at latest follow-up	Parallel	0.5-2 years (mean)
Bolland 2015 [221]	Calcium	All fractures	20	58,573	6,725	Male and female population/ mostly living in the community (17x); partly in residential care (3x)	56-86 (mean)	Calcium supplementation/ 500-1,600mg/day/ alone (7x); combined with vitamin D (400-1,000IU/day or 300,000IU i.m. or UV exposure, 13x)/ partly with alendronate (1x)	Placebo (10x); not further described (8x)/ partly with vitamin D (1x); alendronate (1x)	Fracture at any site	Parallel, factorial, cluster	1-7 years
Chowdhury 2012 [241]	Omega-3 fatty acids	Cerebrovascular disease	2	31,181	978	Male and female population/ without CVD, but at risk of CVD (with hyperlipidaemia or dysglycaemia)	61-63.6 (mean)	Omega-3 fatty acids supplementation/ 1.8g/day EPA (1x); 1g/day EPA+DHA/ partly with Pravastatin 10mg/day or Simvastatin 6mg/day (1x)	Placebo containing olive oil (1x); Pravastatin 10mg/day or Simvastatin 5mg/day (1x)	Fatal or non-fatal ischaemic stroke or haemorrhagic stroke, cerebro-vascular accident, or transient ischaemic attack	Parallel, factorial	4.6-6.2 years (mean)
Chowdhury 2014a [234]	α-linolenic acid	Coronary disease	4	18,866	419	Male and female population/ mostly with CHD (3x); partly general healthy (1x)	40-80 (range) or <70	α-linolenic acid supplementation or dietary oils; dose: 2-5.5g/day margarine supplemented with α-linolenic acid (2x); flaxseed oil (1); linseed oil (1x)	General dietary advice (1x); sunflower oil (2x); placebo margarine (1x)	Fatal or nonfatal myocardial infarction, CHD, coronary insufficiency, coronary death, angina, angio-graphic coronary stenosis	Parallel	1-3.4 years
Chowdhury 2014a [234]	Omega-6 fatty acids	Coronary disease	8	14,476	974	Male and female population/ mostly male/ with CHD (5x); at high risk of CHD (3x)	30-88 (range) or <66 or <70	Diet rich of linolenic acid; 8-72% linolenic acid intake of total daily fat or 40.5-85g/day of polyunsaturated fatty acids; mixed poly-unsaturated intervention with linoleic acid as the primary fatty acid (6x); linolenic acid–specific supplementation (2x)	Usual diet/ care (2x); unspecific dietary advice (3x); low linolenic acid diet (1x); low linoleic acid diet with high proportion of other fats (2x)	Fatal or nonfatal myocardial infarction, CHD, coronary insufficiency, coronary death, angina,angio-graphic coronary stenosis	Parallel, factorial, crossover, cluster	1-8 years
Chowdhury 2014b [3]	Vitamin D	All-cause mortality	22	30,716	5,114	Male and female population/ with or without pre-existing chronic disease (CVD, metabolic disease, cancer)/ community dwelling or institutionalised	56-85 (IQR)	Vitamin D supplementation/ mostly oral; partly with injections/ vitamin D3: 10-6,000IU/day; vitamin D2: 208-4,500IU/day	Placebo (18x); no treatment (4x)	Deaths from any cause	Parallel, factorial, cluster	0.38-6.8 years (mean)

Chung 2011 [242]	Vitamin D	Colorectal cancer	1	2,686	55	Male and female general population/ elderly/ community dwelling	65-85 (range); 75 (mean)	Vitamin D supplementation/ 100,000IU every 4 months	Placebo	Colorectal cancer incidence (fatal outcomes included)	Parallel	5 years (duration + follow-up)
Chung 2011 [242]	Vitamin D	Breast cancer	1	649	8	Female general population/ elderly/ community dwelling	65-85 (range); 75 (mean)	Vitamin D supplementation/ 100,000IU every 4 months	Placebo	Breast cancer incidence (fatal outcomes included)	Parallel	5 years (duration and follow-up)
Ding 2017 [243]	Dairy	Systolic blood pressure	8	735	N/A	Male and female population/ partly healthy (3x); partly with overweight or obesity (2x); prehypertension (1x); metabolic disease (2x)	20-85 (range)	High dairy intake/ 3-5 servings/day (6x); 400-650g/day (2x)/ partly reduced fat products (4x)	Low or no dairy intake/ usual diet (2x); 1-2 servings/day (2x); diet without milk or dairy (1x); control products equivalent in macro-nutrients and sodium (1x); fruit juice and biscuits (1x); NR (1x)	Systolic blood pressure (mmHg)	Parallel, crossover	1-12 months (duration)
Feng 2015 [4]	Folic acid	Congenital heart defects	1	4,862	30	Female population/ in childbearing age/ planning to get pregnant	NR	Folic acid supplementation/ periconceptional/ capsule including 12 vitamins, four minerals and three trace elements), 3 month before pregnancy/ 0.8mg/day	Placebo-like trace element control group (three trace elements only)	Congenital heart defects/ overall or any one of the subtypes in infants (e.g. conotruncal defects, septal defects)	Parallel	3 month before conception till delivery
Filippini 2020 [244]	Green Tea	Prostate cancer	3	201	32	Male population/ at high risk of prostate cancer / with high-grade prostate intraepithelial neoplasia (2x) and/or atypical small acinar proliferation (1x)	30-80 (range)	Green tea supplementation/ capsules with green tea extract or green tea catechins: 300mg/day (2x); 400mg/day of epigallocatechin-3-gallate (1x)	Placebo	Prostate cancer	Parallel	1 year (duration)
Filippini 2020 [244]	Green Tea	Endometrial cancer	1	1,075	1	Female population/ postmenopausal/ without history of cancer, but with high mammography density	50-70 (range)	Green tea supplementation/ capsules with green tea extract, that is 1,315mg (±116mg) of total catechins/day, including 843mg/day (±44mg/day) of epigallocatechin-3-gallate	Placebo	Endometrial cancer	Parallel	1 year (duration)
Fu 2021 [245]	Vitamin C	Lung cancer	2	22,237	177	Male and female population/ with or at high risk of CVD/ partly with history of CVD, T2D or cancer	60.4-64.3 (mean)	Vitamin C supplementation/ 500mg/day/ with or without vitamin E and/or β-carotene	Placebo/ with or without vitamin E and/or β-carotene	Confirmed diagnosis of lung cancer	Factorial	8-9.4 years (mean)

Fu 2021 [245]	Vitamin C	Breast cancer	1	7,627	257	Female population/ with or at high risk for CVD/ partly with history of cancer or T2D	60.4 (mean)	Vitamin C supplementation/ 500mg/day/ with or without vitamin E and/or β-carotene	Placebo/ with or without vitamin E and/or β-carotene	Confirmed diagnosis of breast cancer	Factorial	9.4 years (mean)
Fu 2021 [245]	Vitamin C	Colorectal cancer	2	22,147	206	Male and female population/ with or at high risk of CVD/ partly with history of CVD, T2D or cancer	60.4-64.3 (mean)	Vitamin C supplementation/ 500mg/day/ with or without vitamin E and/or β-carotene	Placebo/ with or without vitamin E and/or β-carotene	Confirmed diagnosis of colorectal cancer	Factorial	8-9.4 years (mean)
Gayer 2019 [246]	Apples	Body weight	5	393	N/A	Male and female population/ healthy or at risk of CVD or metabolic disease/ partly with hypertension and/or hyper-/ dyslipidaemia	36.2-56.7 (mean)	Apple intake/ 170-900g/day apple or 500-2,250ml/day apple juice or 75g/day dried apple or 22mg/day apple pomace	No or low apple intake: controlled beverage (2,250ml/day); dried plums (100g/day); oatmeal cookie (180g); only restricted diet allowed; green kiwi fruit (360g/day)	Body weight change (kg)	Parallel, crossover	1-12 months
Grosso 2015 [222]	Mediterranean diet	Cardiovascular disease	4	12,293	590	Male and female population/ partly with previous myocardial infarction (2x); partly at risk of CVD (2x)	48-67.3 (mean)	Advice to follow Mediterranean dietary pattern: Indo-Mediterranean diet (1x); Mediterranean diet (3x)	Control interventions: prudent diet (2x); general health advice (1x); low-fat diet (1x)	CVD/ including CVD mortality, myocardial infarction and stroke incidence	Parallel, factorial	2-5 years
Jiang 2019 [5]	Vitamin E	Age-related cataract	6	92,243	5,219	Male and female population/ mostly male (4x)/ without (ascertained) cataract	50-80 (range); ≥45	Vitamin E supplementation/ 50-334mg/day or 267-400mg every other day/ alone (4x)/ partly with β-carotene (20mg/day) (2x)	Placebo	Age-related cataract	Parallel, factorial	4-9.7 years (mean/ median)
Jiang 2019 [5]	β-carotene	Age-related cataract	4	88,266	3,415	Male and female population/ mostly male (3x)/ without (ascertained) cataract	40-84 (range); ≥45	β-carotene supplementation/ alone (2x); with 50mg/day vitamin E (20mg/day) (2x); 50mg every other day	Placebo	Age-related cataract	Factorial	2.1-12 years (median/ mean)
Johnston 2019 [247]	Low red meat	Cardiovascular disease	1	48,835	NR	Female population/ postmenopausal/ mostly overweight or obese (>70%); partly hypertensive	50-79 (range)	Behavioural support to reduce total dietary fat to 20% and to increase intake of vegetables and fruit and grains	Habitual diet/ general dietary advice by educational materials	Cardiovascular mortality	Parallel	<13.8 years
Johnston 2019 [247]	Low red meat	Type 2 diabetes	1	45,887	NR	Female population/ postmenopausal/ without T2D/ mostly overweight or obese (>70%); partly hypertensive	50-79 (range); 62.2 (mean)	Behavioural support to reduce total dietary fat to 20% and to increase intake of vegetables and fruit and grains	Habitual diet/ general dietary advice by educational materials	Type 2 diabetes	Parallel	8.1 years

Johnston 2019 [247]	Low red meat	Colorectal cancer	1	48,835	NR	Female population/ postmenopausal/ mostly overweight or obese (>70%); partly hypertensive	50-79 (range)	Behavioural support to reduce total dietary fat to 20% and to increase intake of vegetables and fruit and grains	Habitual diet/ general dietary advice by educational materials	Colorectal cancer incidence	Parallel	<12.3 years
Jonker 2020 [248]	Folic acid	Mean birthweight	3	1,503	N/A	Female population/ pregnant/ in low- and middle-income countries	NR	Folic acid supplementation/ 0.4-5mg/ with vitamin A (100µg) (1x); with formulated food multimix (1x); with 120mg iron (1x)	No folic acid supplementation: diet + placebo (1x); vitamin A (1x); placebo (1x)	Mean birthweight (kg)	Parallel, cluster	<12 months before pregnancy or 20 weeks of gestation till delivery; NR (1x)
Jonker 2020 [248]	Folic acid	Low birthweight	1	3,084	1,313	Female population/ pregnant/ low- and middle-income countries	NR	Folic acid supplementation/ 400µg with vitamin A (100µg)	No folic acid supplementation: vitamin A alone (100µg)	Incidence of low birthweight; <2500g; measured within 72 hours of birth	Cluster	<12 months before pregnancy
Jonker 2020 [248]	Folic acid	Small for gestational age	1	2,254	1,313	Female population/ pregnant/ low- and middle-income countries	NR	Folic acid supplementation/ 400µg with vitamin A (100µg)	No folic acid supplementation: vitamin A alone, 100µg	Infants whose weight is less than the 10th percentile for gestational age	Cluster	<12 months before pregnancy
Kastorini 2011 [6]	Mediterranean diet	Metabolic syndrome	2	1,404	NR	Male and female population/ at high risk of CVD (1x); with metabolic syndrome (1x)	44 or 67 (mean)	High adherence to Mediterranean diet: at least 250–300g fruits, 125–150g vegetables, 25–50g walnuts, 400g whole grains, increase consumption of olive oil/ carbohydrates: 50%–60%, protein: 15%–20%, fat: 30%, SF: 10%, cholesterol: 300g/day (1x); instructions to increase the 14-item Mediterranean score and free provision of olive oil and/or nuts (1x)	Low adherence to Mediterranean diet; control diet, i.e. low-fat diet or usual care	Metabolic syndrome	Parallel	1-2 years
Kastorini 2011 [6]	Mediterranean diet	HDL-cholesterol	27	3,734	N/A	Male and female population/ healthy and/or at risk of CVD or with CVD, diabetes or metabolic syndrome	18-80 (range); 23-61 (mean)	High adherence to Mediterranean diet: <30-40.1% from fat, mostly diets rich in olive oil and/or nuts and/or fish	Low adherence to Mediterranean diet	HDL-cholesterol (mg/dl)	Parallel, crossover	1 month-5 years
Kastorini 2011 [6]	Mediterranean diet	Triglycerides	27	3,734	N/A	Male and female population/ healthy and/or at risk of CVD or with CVD, diabetes or metabolic syndrome	18-80 (range); 23-61 (mean)	High adherence to Mediterranean diet: <30-40.1% from fat, mostly diets rich in olive oil and/or nuts and/or fish	Low adherence to Mediterranean diet	Triglycerides (mg/dl)	Parallel, crossover	1 months-5 years

Kastorini 2011 [6]	Mediterranean diet	Systolic blood pressure	14	3,068	N/A	Male and female population/ healthy and/or at risk of CVD or with CVD, diabetes or metabolic syndrome	18-80 (range); 44-58 (mean)	High adherence to Mediterranean diet: <30-40.1% from fat, mostly diets rich in olive oil and/or nuts and/or fish	Low adherence to Mediterranean diet	Systolic blood pressure (mmHg)	Parallel, crossover	1 months-5 years
Kim 2018 [223]	Multivitamins/ Minerals	Cardiovascular disease	1	14,641	829	Male population/ generally healthy/ partly with T2D or history of CVD	64.3 (mean)	Multivitamin/ Mineral supplementation: 1 tablet daily containing 13 vitamins and 17 minerals	Placebo	CHD mortality and stroke mortality	Factorial	11.2 years
Kim 2018 [223]	Multivitamins/ Minerals	Coronary heart disease	2	27,658	NR	Male population/ generally healthy/ partly with T2D or history of CVD	48.2-64.3 (mean)	Multivitamin/ Mineral supplementation: 1 tablet/day with 13 vitamins and 17 minerals (1x); combination of 120mg ascorbic acid, 30mg vitamin E, 6mg β-carotene, 100μg selenium, 20mg zinc (1x)	Placebo	Incident CHD events/ cardiac revascularization and fatal and nonfatal myocardial infarction	Parallel, factorial	7.5-11.2 years (mean)
Kim 2018 [223]	Multivitamins/ Minerals	Stroke	1	14,641	786	Male and female population/ generally healthy/ partly with T2D or history of CVD	64.3 (mean)	Multivitamin/ Mineral supplementation/ 1 tablet/day with 13 vitamins and 17 minerals	Placebo	Incident ischemic and haemorrhagic stroke (fatal and nonfatal)	Factorial	11.2 years
Kong 2014 [249]	Vitamins	Gastric cancer	11	152,848	661	Male and female population/ healthy or at high risk for gastric cancer	28-84 (range)	Vitamin supplementation/ vitamin A, B1, B2, B6, B12, C, E, E in different compositions	Placebo	Gastric cancer occurrence	Parallel, factorial	3-14.7 years
Lin 2019 [250]	Vitamin E	Bladder cancer	3	78,590	491	Male population/ mostly general population; partly with (history of) cancer/ partly smokers	56.9-67 (median/ mean)	Vitamin E supplementation/ 50mg/day (1x); 400IU/day α-tocopherol (1x); 400IU/day all-rac-α-tocopherylacetate (1x)	Low vitamin E intake/ control groups, placebo	Bladder cancer incidence	Parallel, factorial	6.1-10.3 years
Lin 2020 [251]	Vitamin D	Nephrolithiasis	7	14,652	220	Male and female population/ without prior nephrolithiasis/ partly with history of low-trauma osteoporotic fracture, knee osteoarthritis, adenomas or prediabetes/ partly obese/ overweight; partly postmenopausal	21-84 (range); 57-78 (mean)	Vitamin D supplementation/ 400-2,000IU/day or 20,000-40,000IU/week or 100,000IU/ month	Placebo/ partly with 500mg/day Calcium (1x)	Nephrolithiasis incidence	NR	1-5 years

Lin 2020 [251]	Calcium	Nephrolithiasis	4	2,934	41	Male and female population/ without prior nephrolithiasis/ partly with adenomas; partly postmenopausal	56-66.7 (mean)	Calcium supplementation/ 600-1,600mg/day	Placebo	Nephrolithiasis incidence	NR	2-4 years
Martinez-Gonzalez 2014b [252]	Olive oil	Coronary heart disease	1	7,447	75	Male and female population/ without CVD, but with T2D or ≥3 cardiovascular risk factors or family history of premature CHD	55-80 (range); 67 (mean)	Mediterranean diet supplemented with extra-virgin olive oil; per +25g/day of consumption (total: 29.3g/day)	Advice to reduce dietary fat (total: 2.6g d)	Myocardial infarction	Parallel	4.8 years
Martinez-Gonzalez 2014b [252]	Olive oil	Stroke	1	7,447	107	Male and female population/ without CVD, but with T2D or ≥3 cardiovascular risk factors or family history of premature CHD	55-80 (range); 67 (mean)	Mediterranean diet supplemented with extra-virgin olive oil; per +25g/day	Advice to reduce dietary fat	Stroke (ischaemic or haemorrhagic)	Parallel	4.8 years
Miller 2014 [253]	Low calorie sweeteners	Body weight	13	1,715	N/A	Male and female population/ generally healthy	4-65 (range): children (3x); adults (10x)	Low calorie sweeteners: low-calorie sweetened beverages (10.8-38oz/day) (7x); aspartame (285-2,700mg/day) (3x); isomalt (30g/day) (1x); aspartame (480-670mg/day) + acesulfame potassium + cyclamate + saccharin (1x); NR (1x)	Sugar sweetened beverages (5x); sugar sweetened food and beverages (1x); assigned to follow a habitual (usual) diet that contained sugar sweetened beverages and sugar sweetened foods (4x); energy-reduced diet (similar to the intervention) that did not include low calorie sweeteners: with table sugar for sweetener (1x); with lactose capsules (1x); without any aspartame- or saccharin-sweetened products (1x)	Body weight change	Parallel, crossover	3-78 weeks
Moazzen 2018 [254]	Folic acid	Colorectal cancer	4	31,192	346	Male and female population/ mostly with history of CVD (3x) or at high risk of CVD; partly with T2D	62.6-68.9 (mean)	Folic acid supplementation/ 2-2.5mg/day/ with vitamin B6 (50mg/day) and/or B12 (1mg/day)	Placebo	Colorectal cancer incidence	Parallel, factorial	3-7.3 years
Mocellin 2017 [255]	Vitamin B6	Cancer	9	34,911	2,539	Male and female population/ mostly with CVD (8x); partly with chronic renal failure (1x)	46.3-68.9 (mean)	Vitamin B6 supplementation/ 3-100mg/day/ partly in combination with vitamin B12 and folate; or high dose of micronutrients	Placebo (8x); low doses of micronutrients (1x)	Incidence of cancer (any site)	Parallel, factorial	2-7.3 (mean)

Morze 2021 [233]	Mediterranean diet	Cancer mortality	1	605	7	Male and female population/ mostly men/ with previous myocardial infarction	54 (mean)	Mediterranean diet/ instruction to follow a Mediterranean-type of diet	Control diet/ close to the step 1 American Heart Association prudent diet	Cancer mortality	Parallel	4 years
Morze 2021 [233]	Mediterranean diet	Breast cancer	1	4,152	35	Female population/ postmenopausal/ without history of cancer or CVD, but at risk of CVD	60-80 (range)	Mediterranean diet/ instruction to follow a Mediterranean-type of diet supplemented with nuts or extra-virgin olive oil	Control diet/ advice on a low-fat diet	Breast cancer	Parallel	4.8 years
Picasso 2019 [256]	Vegetarian diet	Triglycerides	5	689	N/A	Male and female population/ mostly female/ mostly overweight or obese/ partly with T2D (1x)	25.4-57.9 (mean)	Vegetarian diet/ dietary counselling to adhere to vegetarian diet/ emphasise consumption of plant foods like legumes, vegetables, grains and fruits, exclude meats/ partly excluding also animal derived foods (2x)	Omnivorous diet/ conventional diet, consuming meat, poultry or fish	Triglyceride levels (mmol/l)	Parallel, factorial	12-74 weeks
Picasso 2019 [256]	Vegetarian diet	HDL-cholesterol	4	596	N/A	Male and female population/ mostly female/ mostly overweight or obese/ partly with T2D (1x)	45.2-57.9 (mean)	Vegetarian diet/ dietary counselling to adhere to vegetarian diet/ emphasise consumption of plant foods like legumes, vegetables, grains and fruits, exclude meats/ partly excluding also animal derived foods (2x)	Omnivorous diet/ conventional diet, consuming meat, poultry or fish	HDL-cholesterol (mmol/l)	Parallel, factorial	12-74 weeks
Sayehmiri 2018 [257]	Selenium	Prostate cancer	9	45,638	23,994	Male population/ without history of prostate cancer/ partly at high risk of prostate cancer or with history of skin cancer or basal cell or squamous cell carcinoma	63-65.4 (mean); >55; >40	Selenium supplementation/ 200-400µg/day/ as selenised yeast or selenomethionine	Placebo	Prostate cancer	Parallel, factorial	NR
Schwingshackl 2015 [237]	Mediterranean diet	Type 2 diabetes	1	3,541	273	Male and female population/ without CVD, but with CVD risk factors	55-80 (range)	Highest adherence to a Mediterranean diet (in primary study: Intervention promoting the Mediterranean diet supplemented with olive oil or mixed nuts)	Lowest adherence to a Mediterranean diet (in primary study: advice on a low-fat diet)	Type 2 diabetes	Parallel	4.1 years
Schwingshackl 2017 [258]	Olive oil	Type 2 diabetes	1	2,301	181	Male and female population/ without T2D or CVD, but with CVD risk factors	55-80 (range); 67.2 (mean)	Highest olive oil intake (in primary study: Intervention promoting the Mediterranean diet supplemented with extra-virgin olive oil)	Lowest olive oil intake category (in primary study: advice on a low-fat diet)	Type 2 diabetes	Parallel	4.1 years

Setien-Suero 2016 [259]	Homocysteine	Mini mental state examination score	2	1,109	N/A	Male and female general population/ elderly	73.4-79.1 (mean)	Vitamin supplementation to reduce Homocysteine levels/ 1 tablet/day/ with folate (1mg) + B12 (0.5mg) + B6 (10mg) (1x); folate (0.4mg) + B12 (0.5mg) + D3 (0.015mg) (1x)	Placebo	Cognition assessed via mini mental state examination score	Parallel	2 years
Stratton 2011 [224]	Multivitamins	Prostate cancer	1	5,034	103	Male population/ generally healthy/ without severe health problems	51.3 (mean)	Multivitamin supplementation/ 1 tablet/day/ composition: 120mg vitamin C, 30mg α-tocopherol, 6mg β-carotene, 100μg selenium, 20mg zinc	Placebo	Prostate cancer incidence	Parallel	8.8 years (mean)
Stratton 2011 [224]	Vitamin E	Prostate cancer	3	39,318	1,969	Male population/ partly smokers/ partly with PSA <4.0ng/dl and normal rectal examination (1x)	50-69 (range); ≥50	Vitamin E supplementation/ 400IU/day (1x); 400IU every other day (1x); 50mg/day (α-tocopherol) (1x)	Placebo	Prostate cancer incidence	Factorial	5-8 years (mean)
Stratton 2011 [224]	Vitamin C	Prostate cancer	1	7,000	1,008	Male population/ partly with history of cancer or myocardial infarction or stroke	≥50	Vitamin C supplementation/ 500mg/day/ with Placebo vitamin E	Placebo	Prostate cancer incidence	Factorial	8 years (mean)
Stratton 2011 [224]	β-carotene	Prostate cancer	1	14,569	147	Male population/ generally healthy/ smokers (≥5 cigarettes/day)	50-69 (range)	β-carotene supplementation/ 20mg/day	Placebo	Prostate cancer incidence	Factorial	5-8 years/ 6.1 (median)
Te Morenga 2013 [260]	Low dietary sugars	Weight gain	10	382	N/A	Male and female population/ mostly healthy (7x); partly at risk of CVD (2x); with metabolic disease (1x)/ partly overweight	18-55 (range); NR (2x)	High-sugar diet with sugar-rich food, drinks or both/ sugar in foods: 80->100g/day or 28% of total energy/ sugar sweetened beverages: 1l or 1,135g/day/ partly: with additional fibre depletion (1x); low fat (1x)	Diet with high amount of complex carbohydrates, artificial sweeteners or low to moderate sugars (10-40g/day); partly with fibre depletion (1x); low fat (1x)	Change in body weight (kg)	Parallel, crossover	1-6 months

Te Morenga 2013 [260]	Low dietary sugars	Body mass index	3	1,627	N/A	Male and female general population/ primary schoolchildren (2x); adolescents (1x)	7-18 (range)	Dietary intervention to reduce sugar intake/ counselling to reduce sugar-sweetened beverages, home deliveries of non-caloric beverages (1x); nutrition education to reduce fat and sugar intake and increase intake of complex carbohydrates (1x); behavioural classroom intervention to reduce intake of sugar sweetened beverages (1x)	General dietary advice/ nutrition education without specific advice regarding reduction of sugars or no intervention	Change in Body mass index (kg/m^2)	Parallel, cluster	6-8 months (duration)
Thorne-Lyman 2012 [225]	Vitamin D	Mean birthweight	5	932	N/A	Female general population/ pregnant/ without pre-existing diseases	18-45 (range); 25-27 (mean)	Vitamin D supplementation/ 400-4,000IU/day or single dose of 200,000IU or 2 doses of 600,000IU/ postconceptional	Nil (3x); placebo (1x); low dose (400IU/day) (1x)	Mean birthweight (g)	Parallel	<16 -28 weeks of gestation till delivery
Trikalinos 2012 [239]	Vitamin E	Cardiovascular disease	14	111,481	NR	Male and female population/ mostly with (history of) CVD (8x); partly at risk of CVD (3x); partly with T2D (1x); partly general population (2x)	55-69 (mean)	Vitamin E supplementation/ 50-1,200IU/day/ mostly 300-800IU/day (12x)	Placebo (8x); placebo and/or vitamin A (1x); placebo and/or vitamin C (1x); nil (3x); soybean oil (1x)	Cardiac or cardiovascular death (as defined in the primary studies)	Parallel, factorial	NR
Vinceti 2018a [7]	Selenium	Any cancer	5	21,860	2,332	Male and female population/ with diverse health status/ partly healthy, partly at high risk for prostate cancer, with resected non-small-cell lung cancer, BRCA1+ mutation carriers or history of skin cancer	62.3-66 (mean/ median); NR (1x)	Selenium supplementation/ 200-400µg/day as selenised yeast (2x); 200µg/day as L-selenomethionine (1x); 250µg/day as sodium selenite (1x)	Placebo	Incidence of any cancer	Parallel	3-13 years
Vinceti 2018a [7]	Selenium	Cancer mortality	2	18,698	359	Male and female population/ mostly male/ partly healthy, partly with history of skin cancer	62.3-63.4 (mean/ median)	Selenium supplementation/ 200µg/day as selenised yeast (1x); 200µg/day as L-selenomethionine (1x)	Placebo	Death from cancer	Parallel	8-13 years
Vinceti 2018b [236]	Selenium	Type 2 diabetes	5	22,053	1,603	Male and female population/ mostly men/ at risk of prostate cancer, with history of skin cancer, with resected non-small-cell lung cancer or removed colorectal adenomas	63-66 (mean)	Selenium supplementation/ 200µg/day as selenised yeast (4x); 200µg/day as L-selenomethionine (1x)	Placebo	Type 2 diabetes	Parallel	3-13 years (follow-up)

Wien 2012 [226]	Folic acid	Cancer	10	38,233	3,515	Male and female population/ mostly with CVD or at high risk of CVD; partly with history of colorectal adenoma or chronic renal disease	57-69 (mean)	Folic acid supplementation/ ≥0.4mg/day/ alone (1x); with aspirin (2x); vitamin B12 + B6 (6x); vitamin B12 (1x)	Placebo (9x); low dose (20µg/day; 1x)/ alone (5x); with aspirin (2x); vitamins B12 + B6 (1x); vitamin B6 (2x)	Total cancer incidence	Parallel, factorial	1.7-7.3 years (mean)
Wolf 2017 [8]	Multivitamins	Preterm birth	1	353	17	Female population/ pregnant/ ≤13 weeks of gestation/ high-income country, low-income multi-ethnic population	28 (mean)	Multivitamin and mineral supplementation/ postconceptional	Placebo	Preterm birth/ delivery <37 weeks of gestational age	Parallel	First trimester till delivery
Wolf 2017 [8]	Multivitamins	Stillbirth	1	261	2	Female population/ with previous neural tube defect affected pregnancy/ planning to have another child/ in high-income country	31.3 (mean)	Multivitamin supplementation/ 3 tablets/day/ pre- and peri-conceptional (at least two months before conception and until the date of the third missed period)	Folic acid supplementation/ 360µg/day	Stillbirth	Parallel	>2 month pre-conception till delivery
Yang 2016 [235]	Folic acid	Gestational hypertension	1	1,078	89	Female population/ pregnant/ HIV-infected/ low-income country	24.7 (mean)	Folic acid supplementation/ postconceptional/ with other multivitamins	Placebo	Gestational hypertension	Factorial	Till 3 months postpartum
Yang 2020 [227]	Calcium	Cardiovascular disease	4	3,996	NR	Male and female population/ generally healthy	58-75.1 (mean); ≥55	Calcium supplementation/ 1,000-1,200mg/day/ as calcium carbonate (1x); elemental calcium (2x); lactogluconatecarbonate (100g/day, 1x)	Placebo	Cardiovascular disease incidence and mortality	Parallel	4-5 years
Yao 2019 [228]	Vitamin D	Any fracture	11	34,243	2,843	Male and female population/ partly institutionalised (3x) or with previous fractures (5x)	61.8-85 (mean)	Vitamin D supplementation/ 400-833IU/day (3x); 20,000-100,000IU/week (2x); multiple bolus of 100,000-500,000IU (6x)/ oral (10x); i.m. injection (1x)	Placebo (10x); nil (1x)	Any fracture/ fractures that occurred at any site	Parallel, factorial	0.8-5 years
Yu 2021 [229]	Folic acid	Pre-eclampsia	3	5,108	441	Female population/ pregnant/ partly at high risk of pre-eclampsia (2x)	26-31 (mean)	Folic acid supplementation/ 4-5mg/day/ postconceptional	Placebo/ partly with low dose (200µg/day; 1x)	Pre-eclampsia	Parallel	1-4 years
Zhang 2016 [9]	Selenium	Cardiovascular disease	9	36,511	NR	Male and female population/ mostly generally healthy (6x); partly with CVD (3x)	46.6-76.2 (mean)	Selenium supplementation/ 100-200µg/day/ alone (3x); combined with other antioxidants (6x)	Placebo	Cardiovascular disease incidence and mortality	Parallel, factorial	0.25-12 years

Zhang 2018 [230]	Vitamin D	Gestational diabetes	4	968	87	Female population/ pregnant/ partly healthy (2x); partly with Vitamin D insufficiency (2x)	25.2-29.5 (mean); NR (1x)	Vitamin D supplementation/ 4,000-5,000IU/day (2x); 50,000IU/2 weeks (1x); 60,000-480,000IU total depending on 25(OH)D level (1x)/ mostly alone (3x); partly with 200mg/day ferrous sulfate and 600mg/day calcium (1x)	Low dose (400IU/day; 2x); nil (1x); routine care with 200mg/day ferrous sulfate and 600mg/day calcium (1x)	Gestational diabetes mellitus (hyperglycaemia first recognised at any stage of pregnancy)	Parallel	12/ <20weeks of gestation to 34-36 weeks of gestation or delivery
Zhang 2018 [230]	Vitamin D	Fasting plasma glucose	16	NR	N/A	Female population/ pregnant/ mostly with gestational diabetes (13x); partly at risk of pre-eclampsia (3x) or with Vitamin D insufficiency (2x)	NR	Vitamin D supplementation/ 10-5,000IU/day or 50,000IU/2 weeks or 50,000IU/month or 150,000-700,000IU total/ alone (14x); via primrose oil (1x); with calcium (1x)/ oral substitution (12x); intramuscular injection (4x)	Placebo (5x); nil (3x); low dose (400IU/day; 2x); low dose vitamin D + insulin (2x); insulin (2x); dietary intervention (1x); yogurt (1x)	Fasting plasma glucose level (difference)	NR	4-16 weeks (8x); 12 weeks of gestation to 34-36 weeks of gestation or delivery (3x); once (4x);NR(1x)
Zhao 2014 [261]	Multivitamins/ Minerals	Nuclear cataract	2	3,161	362	Male and female general population/ partly at high risk of chronic nutrient deficiencies or with early cataract	45-75 (range)	Multivitamin and mineral supplementation	Placebo	Nuclear cataract incidence	Parallel, factorial	5-9 years
Zhao 2014 [261]	Multivitamins/ Minerals	Cortical cataract	2	3,161	960	Male and female general population/ partly at high risk of chronic nutrient deficiencies or with early cataract	45-75 (range)	Multivitamin and mineral supplementation	Placebo	Cortical cataract incidence	Parallel, factorial	5-9 years
Zhao 2014 [261]	Multivitamins/ Minerals	Posterior subcapsular cataract	2	3,161	156	Male and female general population/ partly at high risk of chronic nutrient deficiencies or with early cataract	45-75 (range)	Multivitamin and mineral supplementation	Placebo	Posterior subcapsular cataract incidence	Parallel, factorial	5-9 years
Zhou 2017 [232]	Vitamin D	Preterm birth	5	1,633	74	Female population/ pregnant/ generally healthy/ without HIV infection/ partly with gestational diabetes (1x)	25.2-31.1 (mean); NR (1x)	Vitamin D supplementation/ 1,000-4,000IU/day or multiple bolus dosage (60,000-480,000IU in 1-4 doses)	Placebo (4x); routine antenatal care (200mg/day ferrous sulfate + 600mg/day calcium) (1x)	Delivery of a liveborn neonate before 37 weeks of gestation	Parallel	14-28 weeks of gestation till delivery

BoE=bodies of evidence; CHD=coronary heart disease; CVD=cardiovascular disease; DHA=docosahexaenoic acid; EPA=eicosapentaenoic acid; HDL=high density lipoprotein; HIV=human immunodeficiency virus; IQR=interquartile range; N/A=not applicable; NR=not reported; PSA=prostate-specific antigen; SF=saturated fatty acids; T2D=type 2 diabetes mellitus; UV=ultraviolet

Supplementary Table S7 Characteristics of included BoE from cohort studies

Reference	Exposure	Outcome	Studies, n	Sample size, n	Cases, n	Description of population	Age (years)	Description of exposure	Description of comparator	Description of outcome	Study design	Study length/ follow-up
Aburto 2013 [2]	Low sodium	All-cause mortality	2	18,184	NR	Male and female general population	24-74 (range); >30	High sodium intake	Low sodium intake	All-cause mortality	Prospective cohort	8.7-22 years
Aburto 2013 [2]	Low sodium	Cardiovascular disease	3	76,914	NR	Male and female general population	25-79 (range); >30	High sodium intake	Low sodium intake	Cardiovascular disease incidence and mortality	Prospective cohort	8.7-22 years
Afshin 2014 [240]	Nuts	Coronary heart disease	6	284,683	8,712	Male and female general population/ mostly healthy; partly adults at high risk of CVD (1x)	34-84 (range); 25+	Nut consumption/ dose-response: per 4 weekly servings (28.4g) of nuts	Dose-response	Total incident ischemic heart disease (fatal and nonfatal)	Cohort	4.8-30 years
Afshin 2014 [240]	Nuts	Stroke	2	148,238	5,454	Male and female population/ generally healthy/ healthcare professionals	30-86 (range)	Nut consumption/ dose-response: per 4 weekly servings (28.4g) of nuts	Dose-response	Total stroke (ischemic and haemorrhagic)	Cohort	21.1-26 years
Afshin 2014 [240]	Nuts	Type 2 diabetes	4	226,675	13,035	Male and female population/ generally healthy	35-87 (range)	Nut consumption/ dose-response: per 4 weekly servings (28.4g) of nuts	Dose-response	Type 2 diabetes	Cohort	4.6-23 years
Aguilar-Cordero 2020 [218]	Vitamin D	Pre-eclampsia	7	11,902	4,658	Pregnant women/ general healthy without specific previous pathologies	NR	Low vitamin D status/ deficiency/ 25(OH)D level in maternal blood <50nmol/l	Normal vitamin D status/ 25(OH)D level in maternal blood >50nmol/l	Gestational hypertension with proteinuria and/or other maternal organ dysfunction and/or utero-placental dysfunction/ ≥20 weeks of gestation	Cohort, nested case-control	10-36.9 weeks of gestation till delivery
Alexander 2017 [219]	Omega-3 fatty acids	Coronary heart disease	17	687,166	NR	Male and female general population (6x male; 3x female; 8x both)/ mostly generally healthy (16x); partly with coronary artery disease (1x)	>18	High intake of omega-3 fatty acids/ EPA and DHA/ ≥0.15-≥0.62g/day EPA and 0.22-5.18g/day DHA	Low intake of omega-3 fatty acids/ <0.04 - ≤0.39g/day EPA and DHA ≤1.48g/day	Fatal or nonfatal myocardial infarction, CHD incidence, coronary death, sudden cardiac death, angina	Cohort	4.8-40 years
Amegah 2017 [231]	Vitamin D	Gestational length	1	401	N/A	Pregnant women/ with singleton pregnancy before 16 weeks of gestation	29.3 (mean)	Low vitamin D status/ insufficiency/ 25(OH)D level in maternal blood <28nmol/l at 28-32 weeks of gestation	Normal vitamin D status/ 25(OH)D level in maternal blood ≥28nmol/l	Gestational length (weeks)	Prospective cohort	1.5 years/ <16 weeks of gestation till delivery

Azad 2017 [220]	Nonnutritive sweeteners	Body mass index	1	2,222	N/A	Male and female general population (58% female)/ mean body mass index at baseline: 27	44 (mean)	High intake of nonnutritive sweeteners/ ≥22 servings/week of artificially sweetened beverages (including sodas and other beverages, e.g. coffee, tea)	Low intake of nonnutritive sweeteners intake/ nonusers	Change in body mass index (kg/m^2)	Prospective cohort	8 years
Bolland 2015 [221]	Calcium	All fractures	5	116,199	11,194	Male and female general population/ mostly female	50-79 (range); 56-77 (mean)	Calcium supplementation/ partly with vitamin D (1x)	No calcium supplementation	Fractures at any site	Prospective cohort	3-8.4 years
Chowdhury 2012 [241]	Omega-3 fatty acids	Cerebrovascular disease	10	301,023	4,197	Male and female general population/ healthy	16-84 (range)	High omega-3 fatty acid intake/ 0.11-2.6g/day	Low omega-3 fatty acid intake	Fatal or non-fatal ischaemic stroke, haemorrhagic stroke, cerebro-vascular accident, transient ischaemic attack	Prospective cohort	4-28 years
Chowdhury 2014a [234]	α-linolenic acid	Coronary disease	7	157,258	7,431	Male and female population/ mostly healthy (6x); partly population with high CVD risk (1x)	30-84 (range)	High α-linolenic acid intake	Low α-linolenic acid intake	Fatal or nonfatal myocardial infarction, CHD, coronary insufficiency, coronary death, angina, angio-graphic coronary stenosis	Prospective cohort	5-23 years
Chowdhury 2014a [234]	Omega-6 fatty acids	Coronary disease	8	206,376	8,155	Male and female population/ mostly generally healthy (7x); partly with pre-existing high CVD risk (1x)	20-75 (range)	High omega-6 fatty acid intake	Low omega-6 fatty acid intake	Fatal or nonfatal myocardial infarction, CHD, coronary insufficiency, coronary death, angina, angio-graphic coronary stenosis	Prospective cohort	5-23 years
Chowdhury 2014b [3]	Vitamin D	All-cause mortality	68	840,908	64,636	Male and female population/ with or without pre-existing chronic disease	29.2-80 (range); 63-63.4 (mean/ median)	Low vitamin D status/ 25(OH)D level <10-29ng/ml in serum or plasma	High vitamin D status/ 25(OH)D level ≥30ng/ml in serum or plasma	Deaths from any cause	Cohort	0.3-29 years
Chung 2011 [242]	Vitamin D	Colorectal cancer	9	7,402	3,136	Male and female general population/ partly smokers (2x)	40-79 (range); 57-69.2 (mean)	Vitamin D status/ dose-response per 10nmol/l increase in 25(OH)D concentration	Dose-response	Colorectal cancer incidence and mortality	Nested case-control	7 months- 17 years
Chung 2011 [242]	Vitamin D	Breast cancer	4	5,995	2,986	Female population/ partly postmenopausal	30-85 (range); 57-70 (mean)	Vitamin D status/ dose-response per 10nmol/l increase in 25(OH)D concentration	Dose-response	Breast cancer incidence and/or death	Nested case-control	3.9-15 years
Ding 2017 [243]	Dairy	Systolic blood pressure	27	171,426	N/A	Male and female general population	1-84 (range); NR (3x)	Dairy intake/ dose-response per serving/day increase	Dose-response	Systolic blood pressure (mmHg)	Prospective cohort	2-27 years

Feng 2015 [4]	Folic acid	Congenital heart defects	1	6,112	81	Female population/ women in childbearing age	27.4 (mean)	Folic acid supplementation/ 0.6mg/day/ periconceptional: 1 months before through 3 months after conception/ combined with other multivitamins/ minerals	No folic acid supplementation/ non-user	Congenital heart defects/ overall or any one of the subtypes in infants	Matched cohort	1 month before conception till delivery
Filippini 2020 [244]	Green Tea	Prostate cancer	5	122,110	1,179	Male population/ without history of cancer (2x); without history of prostate cancer (2x); atomic-bomb survivors without prostate cancer (1x)	40-79 (range); NR (2x)	High green tee intake	Low green tea intake/ <1 cup or time/day	Prostate cancer	Prospective cohort	8-21 years
Filippini 2020 [244]	Green Tea	Endometrial cancer	1	53,724	117	Female general population/ without history of cancer	40-69 (range)	Highest green tee exposure/ ≥5 cups/day	Lowest green tea exposure/ ≤4 cups/week	Endometrial cancer	Prospective cohort	5 years
Fu 2021 [245]	Vitamin C	Lung cancer	3	144,263	1,406	Male and female general population/ partly elderly (1x)	50-84 (range)	Vitamin C supplementation/ >66.6->322mg/day or 500mg/day	No vitamin C supplementation/ non-users	Lung cancer incidence	Prospective cohort	4.05-10.6 years
Fu 2021 [245]	Vitamin C	Breast cancer	6	215,581	8,050	Female population/ postmenopausal (3x)	40-84 (range)	Vitamin C supplementation/ >64mg/day - >1,000IU/day or ever supplement use (1x)	No vitamin C supplementation/ non-users	Breast cancer incidence	Prospective cohort, nested case-control	5-10.6 years
Fu 2021 [245]	Vitamin C	Colorectal cancer	2	50,700	246	Male and female general population/ partly residents of a retirement community (1x)	57.1 (mean); NR (1x)	Vitamin C supplementation/ ≥360mg/day (1x)	No vitamin C supplementation/ non-users	Colorectal cancer incidence	Prospective cohort	4.5-7 years
Gayer 2019 [246]	Apples	Body weight	1	133,468	N/A	Male and female population/ healthy or at risk for CVD or cardio-metabolic disease/ without T2D or existing CVD	41.65 (mean)	Increased intake of apple per servings/day	Dose-response, per serving/day	Body weight change (kg)	Prospective cohort	4 years
Grosso 2015 [222]	Mediterranean diet	Cardiovascular disease	20	888,257	22,987	Male and female population/ partly with previous coronary artery disease (1x); with diabetes (1x); with cardiovascular disease (1x)	20-90 (range); 38-68.6 (mean)	High adherence to Mediterranean diet	Low adherence to Mediterranean diet	CVD incidence (including deaths)	Prospective cohort	2-40 years
Jiang 2019 [5]	Vitamin E	Age-related cataract	6	42,147	NR	Male and female population/ mostly female/ without (ascertained) cataract	43-84 (range); ≥43; ≥49	High vitamin E intake/ 8.4-90.8mg/day	Low vitamin E intake/ 3-6.7mg/day	Age-related cataract	Longitudinal and non-longitudinal cohort	5-15 years
Jiang 2019 [5]	β-carotene	Age-related cataract	7	154,449	NR	Male and female population/ without (ascertained) cataract	43-84 (range); ≥45; ≥49	High β-carotene intake/ 1.94-12.11mg/day	Low β-carotene intake/ 0.47-3mg/day	Age-related cataract	Longitudinal and non-longitudinal cohort	5-15 years

Johnston 2019 [247]	Low red meat	Cardiovascular disease	25	858,554	NR	Male and female population/ generally healthy	33-74.1 (mean)	Low adherence to diet rich in red and processed meat or non-vegetarian diet	High adherence to diet rich in red and processed meat or non-vegetarian diet	Cardiovascular mortality	Prospective cohort	4-26 years
Johnston 2019 [247]	Low red meat	Type 2 diabetes	14	378,788	NR	Male and female population/ generally healthy	36-61.7 (mean); NR (3x)	Low adherence to diet rich in red and processed meat or non-vegetarian diet	Higher adherence to diet rich in red and processed meat or non-vegetarian diet	Type 2 diabetes incidence	Cohort	2-34 years
Johnston 2019 [247]	Low red meat	Colorectal cancer	16	840,980	NR	Male and female population/ generally healthy	NR	Low adherence to diet rich in red and processed meat or non-vegetarian diet	Higher adherence to diet rich in red and processed meat or non-vegetarian diet	Colorectal cancer incidence	Prospective cohort	5-26 years
Jonker 2020 [248]	Folic acid	Mean birthweight	2	2,892	N/A	Female population/ pregnant/ in low- and middle-income countries	NR	Folic acid supplementation/ alone (1x); with iron (1x)	No folic acid supplementation	Mean birthweight (kg)	Cohort	NR
Jonker 2020 [248]	Folic acid	Low birthweight	5	26,270	5,114	Female population/ pregnant/ in low- and middle-income countries	NR	Folic acid supplementation/ mostly with iron supplementation (4x)	No folic acid supplementation	Incidence of low birthweight	Cohort	NR
Jonker 2020 [248]	Folic acid	Small for gestational age	4	239,862	23,666	Female population/ pregnant/ in low- and middle-income countries	NR	Folic acid supplementation/ alone (2x); with iron (1x); with dietary folate intake (1x)	No folic acid supplementation/ non-users	Infants whose weight is <10th percentile for gestational age	Cohort	NR
Kastorini 2011 [6]	Mediterranean diet	Metabolic syndrome	2	4,495	NR	Male and female population/ without CVD risk factors (1x); without diabetes mellitus or metabolic syndrome (1x)	20-90 (range); 54 (mean)	High adherence to Mediterranean diet	Low adherence to Mediterranean diet	Metabolic syndrome	Prospective cohort	6.2-7 years
Kastorini 2011 [6]	Mediterranean diet	HDL-cholesterol	1	2,563	N/A	Male and female population/ without CVD risk factors	20-90 (range)	High adherence to Mediterranean diet	Low adherence to Mediterranean diet	HDL Cholesterol (mg/dl)	Prospective cohort	6.2 years
Kastorini 2011 [6]	Mediterranean diet	Triglycerides	1	2,563	N/A	Male and female population/ without CVD risk factors	20-90 (range)	High adherence to Mediterranean diet	Low adherence to Mediterranean diet	Triglycerides (mg/dl)	Prospective cohort	6.2 years
Kastorini 2011 [6]	Mediterranean diet	Systolic blood pressure	1	2,563	N/A	Male and female population/ without CVD risk factors	20-90 (range)	High adherence to Mediterranean diet	Low adherence to Mediterranean diet	Systolic blood pressure (mmHg)	Prospective cohort	6.2 years
Kim 2018 [223]	Multivitamins/ Minerals	Cardiovascular disease	9	587,638	NR	Male and female general population/ without history of CVD and cancer; without medical conditions with a predicted survival of ≤3 years (1x)	50.6-63.9 (mean)	Multivitamin/ Mineral supplementation/ >3 vitamin and mineral ingredients	No multivitamin/Mineral supplementation/ non-users	CHD mortality and stroke mortality	Prospective cohort	5-19 years

Kim 2018 [223]	Multivitamins/ Minerals	Coronary heart disease	6	370,085	NR	Male and female general population/ without history of CVD and cancer; without medical conditions with a predicted survival of ≤3 years (1x)	30-55y (range); 50-63.9 (mean)	Multivitamin/ Mineral supplementation/ >3 vitamin and mineral ingredients	No multivitamin/ Mineral supplementation/ non-users	Incident CHD events/ cardiac revascularization and fatal and nonfatal myocardial infarction	Prospective cohort	7.9-16.2 years
Kim 2018 [223]	Multivitamins/ Minerals	Stroke	3	217,529	NR	Male and female general population/ without history of CVD and cancer (2x); without medical conditions with a predicted survival of ≤3 years (1x)	52.9-63.9 (mean)	Multivitamin/ Mineral supplementation/ >3 vitamin and mineral ingredients	No multivitamin/ Mineral supplementation/ non-users	Incident ischemic and haemorrhagic stroke (fatal and nonfatal)	Prospective cohort	7.9-16.2 years
Kong 2014 [249]	Vitamins	Gastric cancer	7	1,037,967	1,606	Male and female population	40-83 (range); >40	High vitamin intake/ in different compositions: ACE + folate (2x); ACE (1x); A + folate (1x); E (1x); A (1x); NR (1x)	Low vitamin intake	Gastric cancer occurrence	Prospective cohort	6.3-14 years
Lin 2019 [250]	Vitamin E	Bladder cancer	8	497,011	2,774	Male and female general population	30-84 (range)	High vitamin E intake	Low vitamin E intake	Bladder cancer incidence	Prospective cohort	6-20 years
Lin 2020 [251]	Vitamin D	Nephrolithiasis	3	192,126	6,449	Male and female population/ without prior nephrolithiasis	25-75 (range)	High vitamin D supplementation	Low vitamin D supplementation	Nephrolithiasis incidence	Prospective cohort	11.3-12.1 years
Lin 2020 [251]	Calcium	Nephrolithiasis	2	187,976	2,087	Female population/ without prior nephrolithiasis	25-55 (range)	High calcium supplementation	Low calcium supplementation	Nephrolithiasis incidence	Prospective cohort	8-12 years
Martinez-Gonzalez 2014b [252]	Olive oil	Coronary heart disease	3	93,760	1,367	Male and female population/ healthy (2x); without CVD or cancer (1x)	20-86 (range); 49-50 (median)	Olive oil intake/ dose-response per increase of 25g/day	Dose-response	CHD events (fatal and non-fatal)/ acute myocardial infarction, revascularisation, angina and other	Prospective cohort	7.85-10.4 years
Martinez-Gonzalez 2014b [252]	Olive oil	Stroke	2	31,226	543	Male and female population/ without history of stroke (1x); free of CVD and cancer (1x)	73.8 (mean); <55 = 58%, 55-64=23%, ≥65 = 19%	Olive oil intake/ dose-response per increase of 25g/day	Dose-response	Stroke (ischaemic or haemorrhagic)	Prospective cohort	5.25-10.6 years
Miller 2014 [253]	Low calorie sweeteners	Body weight	4	85,353	N/A	Male and female population/ mostly female/ generally healthy	3x adults: 18-64 (range); 1x preschool children: 2-5 (range)	Low calorie sweeteners intake/ saccharin (2x); low-calorie sweetened beverages (2x)	Mean correlation	Body weight change (kg)	Prospective cohort	0.5-4 years
Moazzen 2018 [254]	Folic acid	Colorectal cancer	3	1,926,520	10,514	Male and female general population/ without colorectal cancer	50-64.5 (mean)	High folic acid supplementation/ >800->939µg/day	Low folic acid supplementation/ <242-<300µg/day (2x)	Colorectal cancer or adenoma incidence	Cohort	9.1-16 years

Mocellin 2017 [255]	Vitamin B6	Cancer	29	NR	10,825	Male and female general population	NR	High vitamin B6 intake	Low vitamin B6 intake	Incidence of cancer (any site)	Prospective cohort, nested case-control	NR
Morze 2021 [233]	Mediterranean diet	Cancer mortality	18	1,660,652	71,138	Male and female population	20-90 (range); 38 (mean)	High adherence to Mediterranean diet	Low adherence to Mediterranean diet	Cancer mortality	Prospective cohort	6.25-40 years
Morze 2021 [233]	Mediterranean diet	Breast cancer	12	982,723	35,338	Male and female population/ mostly women	18-104 (range)	High adherence to Mediterranean diet:	Low adherence to Mediterranean diet	Breast cancer	Prospective cohort	3-18 years
Picasso 2019 [256]	Vegetarian diet	Triglycerides	1	302	N/A	Population in inpatient rehabilitation centre/ with cardiovascular disease/ partly overweight	54.5 (mean)	Adherence to lacto-ovo-vegetarian diet	Adherence to omnivorous diet	Triglyceride levels (mmol/l)	Prospective cohort	NR
Picasso 2019 [256]	Vegetarian diet	HDL-cholesterol	1	302	N/A	Population in inpatient rehabilitation centre/ with cardiovascular disease/ partly overweight	54.5 (mean)	Adherence to lacto-ovo-vegetarian diet	Adherence to omnivorous diet	HDL-cholesterol (mmol/l)	Prospective cohort	NR
Sayehmiri 2018 [257]	Selenium	Prostate cancer	2	45,370	672	Male population/ without history of prostate cancer (1x); smokers without history of cancer(1x)	50-76 (range)	High selenium intake and supplementation/ >50 >111.05µg/day	Low selenium intake/ nil (1x); <71.52µg/day (1x)	Prostate cancer incidence	Prospective cohort, trial-based cohort	9-10 years
Schwingshackl 2015 [237]	Mediterranean diet	Type 2 diabetes	8	119,269	NR	Male and female population/ mostly without CVD (6x); partly with myocardial infarction (1x) or prior gestational diabetes mellitus (1x)	20-90 (range)	High adherence to Mediterranean diet	Low adherence to Mediterranean diet	Type 2 diabetes	Prospective cohort	3.2-20 years
Schwingshackl 2017 [258]	Olive oil	Type 2 diabetes	4	183,370	21,688	Male and female general population/ without T2D	36-55 (mean); <50	High olive oil intake	Low olive oil intake	Type 2 diabetes	Prospective cohort	5.7-22 years
Setien-Suero 2016 [259]	Homocysteine	Mini mental state examination score	3	1,625	N/A	Male and female general population/ elderly	67-75.4 (mean)	Homocysteine status	Linear regression	Cognition/ Mini mental state examination score	Prospective cohort	3-7 years
Stratton 2011 [224]	Multivitamins	Prostate cancer	1	295,344	1,331	Male general population/ without cancer	50-71 (range)	Multivitamin supplementation	No multivitamin supplementation/ non-users	Prostate cancer incidence	Prospective cohort	5 years
Stratton 2011 [224]	Vitamin E	Prostate cancer	5	457,136	17,541	Male general population	40-76 (range)	Vitamin E supplementation	No vitamin E supplementation	Prostate cancer incidence	Prospective cohort	4-10 years
Stratton 2011 [224]	Vitamin C	Prostate cancer	1	29,361	1,338	Male population/ without history of prostate, colon, or lung cancer	55-74 (range)	Vitamin C supplementation	No vitamin C supplementation	Prostate cancer incidence	Prospective cohort	4.2 years

Stratton 2011 [224]	β-carotene	Prostate cancer	1	25,016	1,338	Male population/ without history of prostate, colon, or lung cancer	55-74 (range)	β-carotene supplementation	No β-carotene supplementation	Prostate cancer incidence	Prospective cohort	4.2 years
Te Morenga 2013 [260]	Low dietary sugars	Weight gain	4	149,305	N/A	Male and female population/ generally healthy/ younger (university graduates, nurses) and older adults/ without: diabetes (3x); severe diseases (3x)	21-74 (range)	High intake of sugar sweetened beverages/ including: sugar sweetened soft drinks, fruit drinks and fruit juices	Low intake of sugar sweetened beverages	Weight difference (kg)	Prospective cohort	4-6 years
Te Morenga 2013 [260]	Low dietary sugars	Body mass index	4	4,094	N/A	Male and female general population/ children and adolescents	2-14 (range)	Intake of dietary sugars/ dose-response per 1 serving/day or 100g/day intake increase of sugar sweetened beverages (soft drinks, fruit juice)	Dose-response	Change in body mass index (kg/m^2)	Prospective cohort, prospective cohort nested in RCT	1-10 years
Thorne-Lyman 2012 [225]	Vitamin D	Mean birthweight	2	1,497	N/A	Pregnant woman and their children/ without serious non-obstetric problems (1x); without multiple gestations or gestational age >22 weeks at enrolment (1x)	22-32.5 (mean)	Highest categories of vitamin D intake/ including dietary intake and supplementation: >658IU (1x); >535IU/day (1x)	Lowest categories of vitamin D intake/ <446IU (1x); <185IU/day (1x)	Mean birthweight (g)	Prospective cohort	NR (till birth)
Trikalinos 2012 [239]	Vitamin E	Cardiovascular disease	8	137,237	NR	Male and female population/ with or without history of cardiovascular disease	30-93 (range); 50-88 (mean)	High vitamin E intake	Lowest intake category of vitamin E	Cardiac or cardiovascular death (as defined in the primary studies)	Prospective cohort	6-15 years
Vinceti 2018a [7]	Selenium	Any cancer	7	76,239	1,940	Male and female general population/ without cancer	20-74 (range); ≥15; NR (2x)	High selenium exposure/ mostly in serum (6x); partly in plasma and serum (1x)/ 60-136µg/l (1x) or ≥78-240µg/l	Low selenium exposure/ ≤48.9-≤142µg/l	Incidence of any cancer	Nested case-control, cohort-subcohort controlled	5-19 years
Vinceti 2018a [7]	Selenium	Cancer mortality	1	133,957	2,603	Male and female general population/ without history of cancer	40-74 (range)	High selenium intake/ men: ≥31.92µg per 1,000kcal/day; women: ≥33.36µg per 1,000kcal/day	Low selenium intake/ men: <19.36µg/1,000; women: <19.05µg/ 1,000kcal/day	Cancer mortality	Prospective cohort	8.4-13.9 years
Vinceti 2018b [236]	Selenium	Type 2 diabetes	1	7,182	253	Female population/ healthy without history of cancer or T2D	35-70 (range)	High dietary selenium intake/ >65.9µg/day	Low selenium exposure/ ≤47µg/day	Type 2 diabetes	Cohort	16 years
Wien 2012 [226]	Folic acid	Cancer	5	372,799	2,547	Male and female general population	51.7-67 (mean)	Folic acid supplementation/ ≥0.4mg/day	No or low folic acid supplementation/ <0.2mg for women or <0.3mg for men (1x)	Breast (2x); pancreatic (2x) or lung (1x) cancer incidence	Prospective cohort	4-14 years
Wolf 2017 [8]	Multivitamins	Preterm birth	4	42,592	2,280	Female population/ pregnant/ in high-income countries	NR	Multivitamin supplementation/ ≥3 vitamins or minerals/ peri- and postconceptional	No multivitamin use	Preterm birth/ delivery <37 weeks of gestational age	Prospective cohort, matched cohort	3-10 years

Wolf 2017 [8]	Multivitamins	Stillbirth	2	39,845	208	Female population/ pregnant/ in high-income countries	NR	Multivitamin supplementation/ ≥3 vitamins or minerals/ periconceptional	No vitamin use	Stillbirth: fetal death at gestational age ≥28 weeks and/or fetus weigh >1,000g (1x); fetal death at gestational age >20 weeks (1x)	Prospective cohort, matched cohort	3-6 years
Yang 2016 [235]	Folic acid	Gestational hypertension	3	201,265	18,894	Female general population/ pregnant (2x); preparing for marriage or becoming pregnant (1x)	24.9-31.2 (mean)	Folic acid supplementation/ periconceptional/ partly with folate intake	No or low folic acid supplementation (2x) or intake (1x)	Gestational hypertension	Prospective cohort	NR (till delivery)
Yang 2020 [227]	Calcium	Cardiovascular disease	13	823,293	30,117	Male and female general population	17-79 (range)	High calcium intake	Low calcium intake	Cardiovascular disease incidence and mortality	Prospective cohort	9-28 years
Yao 2019 [228]	Vitamin D	Any fracture	10	39,141	6,278	Male and female population/ mostly female/ not living in institutions/ baseline blood 25(OH)D level: 53.5 - 81.0nmol/l	52.6-76.7 (mean) or 68.6 (mean of means)	Vitamin D status/ 25(OH)D level in blood	Dose-response	Any fracture/ fractures at any site	Prospective cohort, nested case-control, case-cohort	5-15 years
Yu 2021 [229]	Folic acid	Pre-eclampsia	13	154,268	NR	Female population/ pregnant/ generally healthy	14-44 (range); 27.8-33.1 (mean); or >18	Folic acid supplementation/ 0.4mg/day/ alone (12x); combined with dietary intake (1x)/ pre- or postconceptional	No folic acid supplementation	Pre-eclampsia	Prospective cohort	2-6 years
Zhang 2016 [9]	Selenium	Cardiovascular disease	14	34,109	3,749	Male and female general population	20-90 (range); ≥35; ≥65; 48	High selenium status/ 101.5µg/l (median)/ serum (11x); plasma (1x); erythrocyte (1x)	Low selenium status/ 53.7µg/l	Cardiovascular disease incidence or mortality	Prospective cohort, nested case-control studies	3-15 years
Zhang 2018 [230]	Vitamin D	Gestational diabetes	24	NR	NR	Female population/ pregnant/ prevalence of Vitamin D sufficiency in populations 3.3 - 73.33% (range); NR (4x)	NR	Low vitamin D status/ 25(OH)D level in blood 25-50nmol/l and/or 50 - 75nmol/l	Normal vitamin D status/ 25(OH)D level in blood >50 or 75nmol/l	Gestational diabetes mellitus (hyperglycaemia first recognised at any stage of pregnancy	Cohort, nested case-control	NR
Zhang 2018 [230]	Vitamin D	Fasting plasma glucose	13	NR	N/A	Female population/ pregnant/ prevalence of Vitamin D sufficiency in populations 5.8 - 58.7% (range); NR (3x)	NR	Vitamin D status/ 25(OH)D level in blood	Correlation	Fasting plasma glucose level	Cohort, nested case-control	NR
Zhao 2014 [261]	Multivitamins/ Minerals	Nuclear cataract	5	13,637	NR	Male and female population/ general (2x); clinic-based population (2x); nondiabetic women (1x)	43-97 (range); ≥40; ≥45	Multivitamin/ Mineral supplementation/ longest duration	No multivitamin/ Mineral supplementation	Nuclear, age-related cataract incidence	Prospective cohort	4.8-15 years

Zhao 2014 [261]	Multivitamins/ Minerals	Cortical cataract	4	12,887	NR	Male and female population/ general (2x); clinic-based population (1x); nondiabetic women (1x)	43-97 (range); ≥45	Multivitamin/ Mineral supplementation/ longest duration	No multivitamin/ Mineral supplementation	Cortical, age-related cataract incidence	Prospective cohort	6.3-15 years
Zhao 2014 [261]	Multivitamins/ Minerals	Posterior subcapsular cataract	3	8,772	NR	Male and female population/ general (1x); clinic-based population (1x); nondiabetic women (1x)	43-86 (range); ≥45	Multivitamin/ Mineral supplementation/ longest duration	No multivitamin/ Mineral supplementation	Posterior subcapsular, age-related cataract incidence	Prospective cohort	5-15 years
Zhou 2017 [232]	Vitamin D	Preterm birth	12	15,184	2,347	Female population/ pregnant women/ without HIV infection	NR	Low vitamin D status/ 25(OH)D level <50nmol/l	High vitamin D status/ 25(OH)D level >50nmol/l	Delivery of a liveborn neonate before 37 weeks of gestation	Prospective cohort, case-cohort, nested case-control	NR (till birth)

BoE=bodies of evidence; CHD=coronary heart disease; CVD=cardiovascular disease; DHA=docosahexaenoic acid; EPA=eicosapentaenoic acid; HDL=high density lipoprotein; HIV=human immunodeficiency virus; N/A=not applicable; NR=not reported; T2D=type 2 diabetes mellitus

Supplementary Table S8 Methodological quality assessment of the included systematic reviews (AMSTAR 2)

**Reference**	**1**	**2**	**3**	**4**	**5**	**6**	**7**	**8**	**9 (RCTs)**	**9 (CSs)**	**10**	**11 (RCTs)**	**11 (CSs)**	**12**	**13**	**14**	**15**	**16**	**Overall confidence**	**Search period**
Aburto 2013 [2]	Y	N	Y	Y	Y	Y	N	Y	Y	Y	N	Y	Y	Y	Y	Y	Y	Y	Critically low	08.2011
Afshin 2014 [240]	Y	N	N	Y	N	Y	N	PY	N	N	N	Y	N	N	N	N	Y	Y	Critically low	25.12.2013
Aguilar-Cordero 2020 [218]	Y	N	N	N	N	Y	N	N	Y	PY	N	Y	Y	N	N	N	Y	Y	Critically low	01.2013 – 02.2019
Alexander 2017 [219]	Y	N	Y	PY	Y	N	Y	PY	Y	PY	N	Y	Y	N	N	N	Y	N	Critically low	01.01.1947 – 02.11.2015
Amegah 2017 [231]	Y	N	Y	PY	N	Y	N	Y	Y	PY	N	Y	Y	Y	N	Y	Y	Y	Critically low	06.2015
Azad 2017 [220]	Y	PY	Y	PY	Y	Y	N	Y	Y	PY	Y	Y	Y	Y	Y	Y	Y	Y	Low	01.2016
Bolland 2015 [221]	Y	N	Y	N	N	N	N	Y	Y	N	Y	Y	No MA	Y	Y	Y	Y	Y	Critically low	09.2014
Chowdhury 2012 [241]	Y	N	Y	PY	Y	Y	N	PY	PY	Y	N	Y	Y	N	N	Y	Y	Y	Critically low	09.2012
Chowdhury 2014a [234]	Y	N	Y	Y	N	Y	N	PY	Y	PY	N	Y	Y	Y	Y	N	Y	Y	Critically low	01.07.2013
Chowdhury 2014b [3]	Y	N	Y	PY	Y	Y	N	Y	Y	PY	N	Y	Y	Y	N	N	Y	Y	Critically low	01.08.2013
Chung 2011 [242]	Y	PY	Y	N	N	N	N	Y	N	N	N	N	N	N	N	N	N	Y	Critically low	07.2011
Ding 2017 [243]	Y	N	N	N	N	N	N	PY	N	N	N	Y	Y	N	N	N	Y	Y	Critically low	NR
Feng 2015 [4]	N	N	N	N	N	Y	N	N	PY	PY	N	No MA	No MA	No MA	N	Y	No MA	Y	Critically low	10.10.2014
Filippini 2020 [244]	Y	Y	Y	PY	Y	Y	Y	Y	Y	PY	Y	Y	Y	Y	Y	Y	Y	Y	Moderate	01.2019
Fu 2021 [245]	N	N	Y	N	Y	Y	N	PY	N	N	N	Y	Y	N	N	N	N	Y	Critically low	18.02.2021
Gayer 2019 [246]	Y	N	N	PY	Y	N	N	Y	Y	PY	Y	N	N	N	N	N	N	Y	Critically low	1946 – 08.2019
Grosso 2015 [222]	Y	N	Y	N	Y	N	N	PY	N	PY	N	Y	Y	N	N	Y	Y	N	Critically low	01.2000 – 06.2014
Jiang 2019 [5]	Y	N	N	PY	Y	Y	N	Y	Y	PY	N	Y	Y	N	N	Y	Y	Y	Critically low	06.2018
Johnston 2019 (Vernooij 2019) [247, 350]	Y	Y	Y	Y	Y	Y	N	N	N/A	Y	Y	No MA	Y	Y	Y	N	N	Y	Critically low	04.2019

Johnston 2019 (Zeraatkar 2019) [247, 351]	Y	Y	Y	Y	Y	Y	N	PY	Y	N/A	Y	Y	No MA	Y	Y	No MA	No MA	Y	Low	04.2019
Jonker 2020 [248]	Y	PY	N	N	Y	N	N	PY	Y	PY	Y	N	N	N	N	N	Y	Y	Critically low	1990 – 2017
Kastorini 2011 [6]	N	N	N	N	N	Y	N	N	N	N	N	N	N	N	N	N	Y	Y	Critically low	30.04.2010
Kim 2018 [223]	Y	N	N	PY	Y	Y	PY	PY	N	N	N	Y	N	N	N	N	Y	Y	Critically low	01.1970 – 08.2016
Kong 2014 [249]	Y	N	Y	N	Y	Y	N	PY	PY	PY	N	Y	Y	N	N	Y	Y	Y	Critically low	02.02.2014
Lin 2019 [250]	N	N	N	PY	N	Y	N	PY	N	N	N	Y	Y	N	N	Y	Y	Y	Critically low	01.01.2016
Lin 2020 [251]	Y	N	Y	N	Y	Y	N	Y	Y	PY	N	Y	Y	N	N	Y	Y	Y	Critically low	05.2019
Martinez-Gonzalez 2014b [252]	Y	N	Y	PY	N	Y	Y	Y	N	PY	N	No MA	Y	N	N	N	N	Y	Critically low	12.2013
Miller 2014 [253]	Y	N	Y	N	N	N	N	Y	N	N	N	N	N	N	N	N	Y	Y	Critically low	16.09.2013
Moazzen 2018 [254]	N	N	Y	N	N	N	N	PY	PY	PY	N	N	N	N	N	N	Y	Y	Critically low	01.2000 – 09.2016
Mocellin 2017 [255]	Y	N	Y	PY	N	Y	N	PY	Y	PY	N	Y	Y	Y	N	Y	Y	N	Critically low	01.2016
Morze 2021 [233]	Y	Y	Y	PY	Y	N	Y	Y	Y	Y	Y	Y	Y	N	N	N	Y	Y	Low	01.2017 – 04.2020
Picasso 2019 [256]	Y	N	N	N	Y	Y	N	PY	Y	PY	N	Y	Y	Y	Y	Y	Y	Y	Critically low	24.05.2017
Sayehmiri 2018 [257]	N	N	N	N	Y	Y	N	N	N	N	N	Y	Y	N	N	N	N	Y	Critically low	2016
Schwingshackl 2015 [237]	Y	N	N	PY	Y	Y	Y	PY	PY	PY	N	Y	Y	Y	Y	Y	Y	Y	Low	02.04.2014
Schwingshackl 2017 [258]	Y	N	N	PY	Y	N	N	PY	Y	N	N	Y	Y	Y	N	Y	Y	Y	Critically low	08.2016
Setien-Suero 2016 [259]	N	N	N	N	N	N	PY	PY	N	N	N	Y	Y	N	N	N	N	N	Critically low	01.2005 – 12.2015
Stratton 2011 [224]	Y	N	N	N	Y	N	N	Y	N	N	N	N	N	N	N	N	N	Y	Critically low	02.2010
Te Morenga 2013 [260]	Y	N	Y	N	N	Y	Y	Y	Y	N	Y	Y	Y	Y	Y	Y	N	Y	Critically low	12.2011
Thorne-Lyman 2012 [225]	Y	N	Y	N	N	N	N	PY	Y	N	N	Y	Y	N	N	N	N	Y	Critically low	06.2011
Trikalinos 2012 [239]	N	N	Y	N	N	N	N	PY	N	N	N	N	N	N	N	Y	N	Y	Critically low	07.10.2010
Vinceti 2018a [7]	Y	Y	Y	Y	Y	N	Y	Y	Y	PY	N	Y	Y	Y	Y	Y	Y	Y	Moderate	02.2017

Vinceti 2018b [236]	N	N	Y	N	N	N	N	PY	N	N	N	Y	No MA	N	N	Y	N	N	Critically low	11.06.2018
Wien 2012 [226]	Y	PY	N	PY	Y	Y	PY	Y	Y	N	N	Y	N	N	Y	Y	Y	Y	Low	06.05.2010
Wolf 2017 [8]	Y	N	N	PY	Y	Y	Y	PY	Y	PY	N	No MA	N	Y	Y	N	Y	Y	Critically low	17.06.2016
Yang 2016 [235]	N	N	N	PY	Y	Y	N	N	PY	PY	N	No MA	N	N	N	Y	Y	Y	Critically low	08.12.2014
Yang 2020 [227]	Y	N	Y	N	Y	Y	N	Y	PY	PY	N	Y	Y	Y	Y	Y	Y	Y	Critically low	03.2019
Yao 2019 [228]	Y	PY	Y	N	Y	Y	PY	PY	Y	Y	N	Y	Y	N	Y	Y	Y	Y	Low	31.12.2019
Yu 2021 [229]	Y	N	N	N	Y	Y	N	PY	Y	PY	N	Y	N	N	N	N	N	Y	Critically low	18.06.2020
Zhang 2016 [9]	Y	N	Y	N	Y	Y	N	PY	N	N	N	Y	Y	N	N	Y	Y	Y	Critically low	15.12.2013
Zhang 2018 [230]	Y	N	Y	PY	N	Y	N	PY	Y	PY	N	Y	Y	Y	N	Y	Y	Y	Critically low	16.05.2017
Zhao 2014 [261]	Y	N	Y	N	N	Y	N	PY	N	PY	N	No MA	Y	Y	N	N	Y	Y	Critically low	01.09.2013
Zhou 2017 [232]	Y	N	Y	N	N	Y	N	PY	Y	PY	N	Y	Y	Y	N	Y	Y	Y	Critically low	06.2016

CS=cohort study; MA=meta-analysis; N=no; N/A=not applicable; NR=not reported; PY=partial yes; RCT=randomised controlled trial; Y=yes

NB: We used AMSTAR 2 (A MeaSurement Tool to Assess systematic Reviews, version 2) [352] to evaluate the methodological quality of the systematic reviews included in our sample. Two reviewers (JB, JS) rated the 16 predefined items independently; discrepancies were discussed with a third reviewer (LS). Based on the presence of critical and noncritical weaknesses, the overall confidence in the systematic review findings was graded as "high", "moderate", "low", or "critically low".

Supplementary Table S9 Ratings of PI/ECO similarity degree for included BoE-pairs

BoE			PI/ECO similarities				
**Reference**	**Intervention/ Exposure**	**Outcome**	**Population**	**Intervention/ Exposure**	**Comparator**	**Outcome**	**Overall**
Aburto 2013 [2]	Low sodium	All-cause mortality	2	1	1	1	2
Aburto 2013 [2]	Low sodium	Cardiovascular disease	2	1	1	2	2
Afshin 2014 [240]	Nuts	Coronary heart disease	2	1	1	1	2
Afshin 2014 [240]	Nuts	Stroke	2	1	1	1	2
Afshin 2014 [240]	Nuts	Type 2 diabetes	2	1	1	1	2
Aguilar-Cordero 2020 [218]	Vitamin D	Pre-eclampsia	1	3	3	1	3
Alexander 2017 [219]	Omega-3 fatty acids	Coronary heart disease	2	2	2	1	2
Amegah 2017 [231]	Vitamin D	Gestational length	1	3	3	1	3
Azad 2017 [220]	Nonnutritive sweeteners	Body mass index	2	2	1	1	2
Bolland 2015 [221]	Calcium	All fractures	1	2	1	1	2
Chowdhury 2012 [241]	Omega-3 fatty acids	Cerebrovascular disease	2	2	2	1	2
Chowdhury 2014a [234]	α-linolenic acid	Coronary disease	2	2	2	1	2
Chowdhury 2014a [234]	Omega-6 fatty acids	Coronary disease	2	2	2	1	2
Chowdhury 2014b [3]	Vitamin D	All-cause mortality	2	3	3	1	3
Chung 2011 [242]	Vitamin D	Colorectal cancer	2	3	3	1	3
Chung 2011 [242]	Vitamin D	Breast cancer	2	3	3	1	3
Ding 2017 [243]	Dairy	Systolic blood pressure	2	1	1	1	2
Feng 2015 [4]	Folic acid	Congenital heart defects	1	1	1	1	1
Filippini 2020 [244]	Green Tea	Prostate cancer	3	2	2	1	3
Filippini 2020 [244]	Green Tea	Endometrial cancer	2	2	2	1	2
Fu 2021 [245]	Vitamin C	Lung cancer	2	1	1	1	2
Fu 2021 [245]	Vitamin C	Breast cancer	2	1	1	1	2
Fu 2021 [245]	Vitamin C	Colorectal cancer	2	1	1	1	2
Gayer 2019 [246]	Apples	Body weight	2	1	1	1	2
Grosso 2015 [222]	Mediterranean diet	Cardiovascular disease	2	1	1	1	2
Jiang 2019 [5]	Vitamin E	Age-related cataract	1	2	2	1	2

Jiang 2019 [5]	β-carotene	Age-related cataract	1	2	2	1	2
Johnston 2019 [247]	Low red meat	Cardiovascular disease	2	2	2	1	2
Johnston 2019 [247]	Low red meat	Type 2 diabetes	2	2	2	1	2
Johnston 2019 [247]	Low red meat	Colorectal cancer	2	2	2	1	2
Jonker 2020 [248]	Folic acid	Mean birthweight	1	1	1	1	1
Jonker 2020 [248]	Folic acid	Low birthweight	1	1	1	1	1
Jonker 2020 [248]	Folic acid	Small for gestational age	1	2	2	1	2
Kastorini 2011 [6]	Mediterranean diet	Metabolic syndrome	2	1	1	1	2
Kastorini 2011 [6]	Mediterranean diet	HDL-cholesterol	2	1	1	1	2
Kastorini 2011 [6]	Mediterranean diet	Triglycerides	2	1	1	1	2
Kastorini 2011 [6]	Mediterranean diet	Systolic blood pressure	2	1	1	1	2
Kim 2018 [223]	Multivitamins/ Minerals	Cardiovascular disease	2	1	1	1	2
Kim 2018 [223]	Multivitamins/ Minerals	Coronary heart disease	1	1	1	1	1
Kim 2018 [223]	Multivitamins/ Minerals	Stroke	2	1	1	1	2
Kong 2014 [249]	Vitamins	Gastric cancer	2	2	2	1	2
Lin 2019 [250]	Vitamin E	Bladder cancer	1	2	1	1	2
Lin 2020 [251]	Vitamin D	Nephrolithiasis	1	1	1	1	1
Lin 2020 [251]	Calcium	Nephrolithiasis	1	1	1	1	1
Martinez-Gonzalez 2014b [252]	Olive oil	Coronary heart disease	2	2	2	2	2
Martinez-Gonzalez 2014b [252]	Olive oil	Stroke	2	2	2	1	2
Miller 2014 [253]	Low calorie sweeteners	Body weight	1	1	1	1	1
Moazzen 2018 [254]	Folic acid	Colorectal cancer	2	1	1	1	2
Mocellin 2017 [255]	Vitamin B6	Cancer	2	2	2	1	2
Morze 2021 [233]	Mediterranean diet	Cancer mortality	2	1	1	1	2
Morze 2021 [233]	Mediterranean diet	Breast cancer	2	1	1	1	2
Picasso 2019 [256]	Vegetarian diet	Triglycerides	3	2	2	1	3
Picasso 2019 [256]	Vegetarian diet	HDL-cholesterol	3	2	2	1	3
Sayehmiri 2018 [257]	Selenium	Prostate cancer	2	2	2	1	2
Schwingshackl 2015 [237]	Mediterranean diet	Type 2 diabetes	2	1	1	1	2
Schwingshackl 2017 [258]	Olive oil	Type 2 diabetes	2	1	1	1	2

Setien-Suero 2016 [259]	Homocysteine	Mini mental state examination score	1	3	3	1	3
Stratton 2011 [224]	Multivitamins	Prostate cancer	1	1	1	1	1
Stratton 2011 [224]	Vitamin E	Prostate cancer	1	1	1	1	1
Stratton 2011 [224]	Vitamin C	Prostate cancer	2	1	1	1	2
Stratton 2011 [224]	β-carotene	Prostate cancer	2	1	1	1	2
Te Morenga 2013 [260]	Low dietary sugars	Weight gain	2	2	2	1	2
Te Morenga 2013 [260]	Low dietary sugars	Body mass index	2	2	2	1	2
Thorne-Lyman 2012 [225]	Vitamin D	Mean birthweight	1	2	2	1	2
Trikalinos 2012 [239]	Vitamin E	Cardiovascular disease	2	2	2	1	2
Vinceti 2018a [7]	Selenium	Any cancer	2	3	3	1	3
Vinceti 2018a [7]	Selenium	Cancer mortality	2	2	2	1	2
Vinceti 2018b [236]	Selenium	Type 2 diabetes	2	2	2	1	2
Wien 2012 [226]	Folic acid	Cancer	2	2	2	2	2
Wolf 2017 [8]	Multivitamins	Preterm birth	1	1	1	1	1
Wolf 2017 [8]	Multivitamins	Stillbirth	2	1	1	1	2
Yang 2016 [235]	Folic acid	Gestational hypertension	2	2	2	1	2
Yang 2020 [227]	Calcium	Cardiovascular disease	1	2	2	1	2
Yao 2019 [228]	Vitamin D	Any fracture	2	3	3	1	3
Yu 2021 [229]	Folic acid	Pre-eclampsia	2	2	2	1	2
Zhang 2016 [9]	Selenium	Cardiovascular disease	2	3	3	1	3
Zhang 2018 [230]	Vitamin D	Gestational diabetes	2	3	3	1	3
Zhang 2018 [230]	Vitamin D	Fasting plasma glucose	2	3	3	1	3
Zhao 2014 [261]	Multivitamins/ Minerals	Nuclear cataract	2	1	1	1	2
Zhao 2014 [261]	Multivitamins/ Minerals	Cortical cataract	2	1	1	1	2
Zhao 2014 [261]	Multivitamins/ Minerals	Posterior subcapsular cataract	2	1	1	1	2
Zhou 2017 [232]	Vitamin D	Preterm birth	2	3	3	1	3

BoE= bodies of evidence; HDL=high density lipoprotein; PI/ECO=Population, Intervention/Exposure, Comparator, Outcome

Supplementary Table S10 Analysis of concordance of the included BoE-pairs

Reference	Intervention/ Exposure	Outcome	Summary measure	Direction of effect (RCT)	Significance (RCT)	Direction of association (cohorts)	Significance (cohorts)	Concordance
Aburto 2013 [2]	Low sodium	All-cause mortality	RR	decreasing	not significant	decreasing	not significant	not concordant
Aburto 2013 [2]	Low sodium	Cardiovascular disease	RR	decreasing	not significant	decreasing	not significant	not concordant
Afshin 2014 [240]	Nuts	Coronary heart disease	RR	decreasing	not significant	decreasing	significant	not concordant
Afshin 2014 [240]	Nuts	Stroke	RR	decreasing	significant	decreasing	not significant	not concordant
Afshin 2014 [240]	Nuts	Type 2 diabetes	RR	decreasing	not significant	decreasing	significant	not concordant
Aguilar-Cordero 2020 [218]	Vitamin D	Pre-eclampsia	RR	decreasing	not significant	decreasing	not significant	not concordant
Alexander 2017 [219]	Omega-3 fatty acids	Coronary heart disease	RR	decreasing	not significant	decreasing	significant	not concordant
Amegah 2017 [231]	Vitamin D	Gestational length	SMD	increasing	not significant	increasing	significant	not concordant
			MD	increasing	significant	increasing	significant	concordant
Azad 2017 [220]	Nonnutritive sweeteners	Body mass index	SMD	decreasing	not significant	increasing	significant	not concordant
			MD	decreasing	not significant	increasing	significant	not concordant
Bolland 2015 [221]	Calcium	All fractures	RR	decreasing	significant	increasing	not significant	not concordant
Chowdhury 2012 [241]	Omega-3 fatty acids	Cerebrovascular disease	RR	decreasing	not significant	decreasing	not significant	concordant
Chowdhury 2014a [234]	α-linolenic acid	Coronary disease	RR	decreasing	not significant	decreasing	not significant	not concordant
Chowdhury 2014a [234]	Omega-6 fatty acids	Coronary disease	RR	decreasing	not significant	decreasing	not significant	not concordant
Chowdhury 2014b [3]	Vitamin D	All-cause mortality	RR	decreasing	not significant	decreasing	significant	not concordant
Chung 2011 [242]	Vitamin D	Colorectal cancer	RR	increasing	not significant	decreasing	significant	not concordant
Chung 2011 [242]	Vitamin D	Breast cancer	RR	decreasing	not significant	decreasing	not significant	not concordant
Ding 2017 [243]	Dairy	Systolic blood pressure	MD	decreasing	not significant	decreasing	significant	not concordant
Feng 2015 [4]	Folic acid	Congenital heart defects	RR	decreasing	not significant	decreasing	significant	not concordant
Filippini 2020 [244]	Green Tea	Prostate cancer	RR	decreasing	not significant	increasing	not significant	not concordant
Filippini 2020 [244]	Green Tea	Endometrial cancer	RR	decreasing	not significant	decreasing	not significant	not concordant
Fu 2021 [245]	Vitamin C	Lung cancer	RR	increasing	not significant	increasing	not significant	not concordant
Fu 2021 [245]	Vitamin C	Breast cancer	RR	increasing	not significant	increasing	not significant	not concordant
Fu 2021 [245]	Vitamin C	Colorectal cancer	RR	decreasing	not significant	decreasing	not significant	not concordant
Gayer 2019 [246]	Apples	Body weight	MD	increasing	not significant	decreasing	significant	not concordant

Grosso 2015 [222]	Mediterranean diet	Cardiovascular disease	RR	decreasing	significant	decreasing	significant	concordant
Jiang 2019 [5]	Vitamin E	Age-related cataract	RR	decreasing	not significant	decreasing	not significant	concordant
Jiang 2019 [5]	β-carotene	Age-related cataract	RR	decreasing	not significant	decreasing	significant	not concordant
Johnston 2019 [247]	Low red meat	Cardiovascular disease	RR	increasing	not significant	decreasing	significant	not concordant
Johnston 2019 [247]	Low red meat	Type 2 diabetes	RR	decreasing	not significant	decreasing	significant	not concordant
Johnston 2019 [247]	Low red meat	Colorectal cancer	RR	increasing	not significant	decreasing	not significant	concordant
Jonker 2020 [248]	Folic acid	Mean birthweight	SMD	increasing	significant	increasing	significant	concordant
			MD	increasing	significant	increasing	significant	concordant
Jonker 2020 [248]	Folic acid	Low birthweight	RR	decreasing	not significant	decreasing	significant	not concordant
Jonker 2020 [248]	Folic acid	Small for gestational age	RR	decreasing	not significant	decreasing	not significant	not concordant
Kastorini 2011 [6]	Mediterranean diet	Metabolic syndrome	RR	decreasing	significant	decreasing	not significant	not concordant
Kastorini 2011 [6]	Mediterranean diet	HDL-cholesterol	MD	increasing	significant	increasing	not significant	not concordant
Kastorini 2011 [6]	Mediterranean diet	Triglycerides	MD	decreasing	significant	decreasing	not significant	not concordant
Kastorini 2011 [6]	Mediterranean diet	Systolic blood pressure	MD	decreasing	significant	increasing	not significant	not concordant
Kim 2018 [223]	Multivitamins/ Minerals	Cardiovascular disease	RR	decreasing	not significant	increasing	not significant	concordant
Kim 2018 [223]	Multivitamins/ Minerals	Coronary heart disease	RR	decreasing	not significant	decreasing	significant	not concordant
Kim 2018 [223]	Multivitamins/ Minerals	Stroke	RR	increasing	not significant	decreasing	not significant	not concordant
Kong 2014 [249]	Vitamins	Gastric cancer	RR	increasing	not significant	decreasing	not significant	not concordant
Lin 2019 [250]	Vitamin E	Bladder cancer	RR	increasing	not significant	decreasing	significant	not concordant
Lin 2020 [251]	Vitamin D	Nephrolithiasis	RR	decreasing	not significant	increasing	significant	not concordant
Lin 2020 [251]	Calcium	Nephrolithiasis	RR	increasing	not significant	increasing	significant	not concordant
Martinez-Gonzalez 2014b [252]	Olive oil	Coronary heart disease	RR	decreasing	not significant	decreasing	not significant	not concordant
Martinez-Gonzalez 2014b [252]	Olive oil	Stroke	RR	decreasing	significant	decreasing	significant	concordant
Moazzen 2018 [254]	Folic acid	Colorectal cancer	RR	increasing	not significant	decreasing	not significant	not concordant
Mocellin 2017 [255]	Vitamin B6	Cancer	RR	increasing	not significant	decreasing	not significant	concordant
Morze 2021 [233]	Mediterranean diet	Cancer mortality	RR	decreasing	not significant	decreasing	significant	not concordant
Morze 2021 [233]	Mediterranean diet	Breast cancer	RR	decreasing	significant	decreasing	not significant	not concordant

Picasso 2019 [256]	Vegetarian diet	Triglycerides	SMD	increasing	not significant	increasing	not significant	not concordant
			MD	increasing	not significant	increasing	not significant	not concordant
Picasso 2019 [256]	Vegetarian diet	HDL-cholesterol	SMD	decreasing	not significant	decreasing	not significant	not concordant
			MD	decreasing	not significant	decreasing	not significant	not concordant
Sayehmiri 2018 [257]	Selenium	Prostate cancer	RR	decreasing	not significant	decreasing	not significant	not concordant
Schwingshackl 2015 [237]	Mediterranean diet	Type 2 diabetes	RR	decreasing	significant	decreasing	significant	concordant
Schwingshackl 2017 [258]	Olive oil	Type 2 diabetes	RR	decreasing	significant	decreasing	significant	concordant
Stratton 2011 [224]	Multivitamins	Prostate cancer	RR	decreasing	not significant	increasing	not significant	concordant
Stratton 2011 [224]	Vitamin E	Prostate cancer	RR	increasing	not significant	increasing	not significant	not concordant
Stratton 2011 [224]	Vitamin C	Prostate cancer	RR	increasing	not significant	decreasing	not significant	not concordant
Stratton 2011 [224]	β-carotene	Prostate cancer	RR	increasing	not significant	decreasing	significant	not concordant
Te Morenga 2013 [260]	Low dietary sugars	Body weight change	MD	increasing	significant	increasing	not significant	not concordant
Te Morenga 2013 [260]	Low dietary sugars	Body mass index	MD	decreasing	not significant	decreasing	not significant	concordant
Thorne-Lyman 2012 [225]	Vitamin D	Mean birthweight	SMD	increasing	not significant	increasing	significant	not concordant
			MD	increasing	not significant	increasing	significant	not concordant
Trikalinos 2012 [239]	Vitamin E	Cardiovascular disease	RR	decreasing	not significant	decreasing	significant	not concordant
Vinceti 2018a [7]	Selenium	Any cancer	RR	decreasing	not significant	decreasing	significant	not concordant
Vinceti 2018a [7]	Selenium	Cancer mortality	RR	decreasing	not significant	decreasing	not significant	not concordant
Vinceti 2018b [236]	Selenium	Type 2 diabetes	RR	increasing	significant	increasing	significant	concordant
Wien 2012 [226]	Folic acid	Cancer	RR	increasing	significant	increasing	not significant	not concordant
Wolf 2017 [8]	Multivitamins	Preterm birth	RR	increasing	not significant	decreasing	not significant	not concordant
Wolf 2017 [8]	Multivitamins	Stillbirth	RR	increasing	not significant	decreasing	not significant	not concordant
Yang 2016 [235]	Folic acid	Gestational hypertension	RR	decreasing	significant	increasing	significant	not concordant
Yang 2020 [227]	Calcium	Cardiovascular disease	RR	decreasing	not significant	decreasing	not significant	not concordant
Yao 2019 [228]	Vitamin D	Any fracture	RR	increasing	not significant	decreasing	significant	not concordant
Yu 2021 [229]	Folic acid	Pre-eclampsia	RR	increasing	not significant	decreasing	significant	not concordant
Zhang 2016 [9]	Selenium	Cardiovascular disease	RR	decreasing	not significant	decreasing	significant	not concordant
Zhang 2018 [230]	Vitamin D	Gestational diabetes	RR	decreasing	not significant	decreasing	significant	not concordant
Zhao 2014 [261]	Multivitamins/ Minerals	Nuclear cataract	RR	decreasing	significant	decreasing	significant	concordant

Zhao 2014 [261]	Multivitamins/ Minerals	Cortical cataract	RR	decreasing	not significant	decreasing	significant	not concordant
Zhao 2014 [261]	Multivitamins/ Minerals	Posterior subcapsular cataract	RR	increasing	significant	decreasing	not significant	not concordant
Zhou 2017 [232]	Vitamin D	Preterm birth	RR	decreasing	not significant	decreasing	not significant	not concordant

MD=mean difference; RCT=randomised controlled trial; RR=relative risk; SMD=standardised mean difference

Supplementary Table S11 Subgroup analysis by PI/ECO similarity degree for each domain

	**BoE-pairs included**	**Ratio of risk ratios (95% CI)**	**Heterogeneity (I^2 (%); τ^2)**	**95% prediction interval**
Main analysis	66	1.04 (0.99 to 1.10)	59; 0.02	0.77 to 1.41
Stratified by population similarity degree				
More or less identical	15	1.03 (0.94 to 1.13)	55; 0.01	0.79 to 1.35
Similar but not identical	50	1.05 (0.98 to 1.12)	59; 0.03	0.76 to 1.46
Broadly similar	1	0.46 (0.16 to 1.29)	N/A	N/A
Stratified by intervention/exposure similarity degree				
More or less identical	30	0.99 (0.90 to 1.08)	42; 0.03	0.70 to 1.39
Similar but not identical	27	1.05 (0.98 to 1.13)	46; 0.01	0.81 to 1.36
Broadly similar	9	1.20 (1.06 to 1.36)	53; 0.01	0.90 to 1.60
Stratified by comparator similarity degree				
More or less identical	32	0.99 (0.91 to 1.08)	46; 0.03	0.70 to 1.39
Similar but not identical	25	1.07 (0.99 to 1.15)	39; 0.02	0.84 to 1.34
Broadly similar	9	1.20 (1.06 to 1.36)	53; 0.01	0.90 to 1.60
Stratified by outcome similarity degree				
More or less identical	63	1.05 (0.99 to 1.11)	61; 0.02	0.76 to 1.44
Similar but not identical	3	1.02 (0.89 to 1.16)	0; 0	0.42 to 2.46
Broadly similar	0	N/A	N/A	N/A

BoE=bodies of evidence; CI=confidence interval; N/A=not applicable; PI/ECO=Population, Intervention/Exposure, Comparator, Outcome

Supplementary Table S12 Overlap of primary studies in BoE-pairs with highly similar PI/ECO questions – comparison between the present sample and the sample in Schwingshackl 2021 [1]

Reference	Intervention/ Exposure	Outcome	Matching BoE-pair in Schwingshackl 2021 [1]	Intervention/ Exposure	Outcome	Overlap of RCTs#	Overlap of CSs#
**Binary outcomes (n=19)**							
Aburto 2013 [2]	Low sodium	All-cause mortality	Adler 2014 [353]/ Aburto 2013 [2]	Low sodium	All-cause mortality	4/4	2/2
Aburto 2013 [2]	Low sodium	Cardiovascular disease	Adler 2014 [353]/ Aburto 2013 [2]	Low sodium	Cardiovascular disease	2/2	3/3
			Adler 2014 [353]/ Aburto 2013 [2]	Low sodium	Cardiovascular mortality	1/2	3/3
Aguilar-Cordero 2020 [218]	Vitamin D	Pre-eclampsia	Palacios 2019 [354]/ Yuan 2019 [355]	Vitamin D	Pre-eclampsia	0/7	5/7
Alexander 2017 [219]	Omega-3 fatty acids	Coronary heart disease	Abdelhamid 2018a [356]/ Chowdhury 2014a [234]	Omega-3 fatty acids	Coronary heart disease/ Cardiovascular disease	13/18	7/17
			Abdelhamid 2018a [356]/ Chowdhury 2014a [234]	Omega-3 fatty acids	Cardiovascular mortality/ Coronary heart disease mortality	10/18	N/A
Chowdhury 2014a [234]	α-linolenic acid	Coronary disease	Abdelhamid 2018a [356]/ Wei 2018 [357]	α-linolenic acid	Coronary heart disease	2/4	5/7
Chowdhury 2014a [234]	Omega-6 fatty acids	Coronary disease	Abdelhamid 2018b [358]/ Chowdhury 2014a [234]	Polyunsaturated fat/ Omega-6 fatty acids	Coronary heart disease	4/8	8/8
			Hooper 2018 [359]/ Chowdhury 2014a [234]	Omega-6 fatty acids	Combined cardiovascular events/ Coronary heart disease	4/8	8/8
Chowdhury 2014b [3]	Vitamin D	All-cause mortality	Bjelakovic 2014a [360]/ Chowdhury 2014b [3]	Vitamin D	All-cause mortality	19/22	68/68
Chung 2011 [242]	Vitamin D	Breast cancer	Bjelakovic 2014b [361]/ Hossain 2019 [362]	Vitamin D/ Vitamin D3	Breast cancer	0/1	0/4
Feng 2015 [4]	Folic acid	Congenital heart defects	De-Regil 2015 [363]/ Feng 2015 [4]	Folic acid	Congenital cardiovascular anomalies / Congenital heart defects	0/1	1/1

Grosso 2015 [222]	Mediterranean diet	Cardiovascular disease	Rees 2019 [364]/ Rosato 2019 [365]	Mediterranean diet	Combined cardiovascular events / Cardiovascular disease	1/4	5/20
			Rees 2019 [364]/ Rosato 2019 [365]	Mediterranean diet	Cardiovascular mortality	1/4	4/20
Jiang 2019 [5]	Vitamin E	Age-related cataract	Mathew 2012 [366]/ Jiang 2019 [5]	Vitamin E	Cataract	3/6	6/6
Jiang 2019 [5]	β-carotene	Age-related cataract	Mathew 2012 [366]/ Jiang 2019 [5]	β-carotene	Cataract	2/4	7/7
Vinceti 2018a [7]	Selenium	Any cancer	Vinceti 2018a [7]	Selenium	Cancer	5/5	7/7
Vinceti 2018a [7]	Selenium	Cancer mortality	Vinceti 2018a [7]	Selenium	Cancer mortality	2/2	1/1
Wolf 2017 [8]	Multivitamins	Preterm birth	Keats 2019 [367]/ Wolf 2017 [8]	Micronutrients/ Multivitamins	Preterm birth	0/1	4/4
Yao 2019 [228]	Vitamin D	Any fracture	Avenell 2014 [368]/ Feng 2017 [369]	Vitamin D	Any fracture	8/11	4/10
Zhang 2016 [9]	Selenium	Cardiovascular disease	Rees 2013b [370]/ Xiang 2019 [371]	Selenium	Cardiovascular mortality	2/9	1/14
			Rees 2013b [370]/ Zhang 2016 [9]	Selenium	Combined cardiovascular events / Cardiovascular disease	2/9	14/14
Zhang 2018 [230]	Vitamin D	Gestational diabetes	Palacios 2019 [354]/ Hu 2018 [372]	Vitamin D	Gestational diabetes	3/4	9/24
Zhou 2017 [232]	Vitamin D	Preterm birth	Palacios 2019 [354]/ Tous 2020 [373]	Vitamin D	Preterm birth	0/5	8/12
**Continuous outcomes (n=5)**							
Ding 2017 [243]	Dairy	Systolic blood pressure	Usinger 2012 [374]/ Soedamah-Muthu 2012 [375]	Fermented milk	Systolic blood pressure/ Hypertension	0/8	2/27
Kastorini 2011 [6]	Mediterranean diet	HDL-cholesterol	Rees 2019 [364]/ Kastorini 2011 [6]	Mediterranean diet	HDL-cholesterol	2/27	1/1

Kastorini 2011 [6]	Mediterranean diet	Triglycerides	Rees 2019 [364]/ Kastorini 2011 [6]	Mediterranean diet	Triglycerides	2/27	1/1
Kastorini 2011 [6]	Mediterranean diet	Systolic blood pressure	Rees 2019 [364]/ Kastorini 2011 [6]	Mediterranean diet	Systolic blood pressure	2/14	1/1
			Rees 2013a [376]/ Kastorini 2011 [6]	Healthy diet/ Mediterranean diet	Systolic blood pressure	0/14	1/1
Thorne-Lyman 2012 [225]	Vitamin D	Birthweight	Palacios 2019 [354]/ Tous 2020 [373]	Vitamin D	Mean birthweight/ Birth weight	1/5	0/2

overlap defined as number of primary studies also included in Schwingshackl et al. 2021 [1], reference: total number of primary studies included in the BoE of the present replication study

BoE=bodies of evidence; CS=cohort study; HDL=high density lipoprotein; N/A=not applicable; PI/ECO=Population, Intervention/Exposure, Comparator, Outcome; RCT=randomised controlled trial

Reference	Intervention/Exposure in SR	Type of intake (RCTs)	Type of exposure (cohorts)	Outcome in SR	SMD (RCTs)	95%−CI	SMD (cohorts)	95%−CI	DSMD	95%−CI	Weight
Amegah 2017	Vitamin D	Supplementation	Status	Gestational Length	0.08	[−0.14; 0.31]	0.43	[0.03; 0.82]	−0.35	[−0.80; 0.10]	11.0%
Azad 2017	Nonnutritive sweeteners	Intake + Supplementation	Intake	Body Mass Index	−0.14	[−0.43; 0.16]	0.27	[0.17; 0.38]	−0.41	[−0.72; −0.10]	17.9%
Jonker 2020	Folic acid	Supplementation	Supplementation	Mean Birthweight	0.56	[0.15; 0.97]	0.36	[0.08; 0.65]	0.20	[−0.30; 0.70]	9.6%
Picasso 2019	Vegetarian diet	Intake	Intake	Trigylcerides	0.08	[−0.07; 0.23]	0.03	[−0.19; 0.26]	0.05	[−0.22; 0.32]	20.8%
Picasso 2019	Vegetarian diet	Intake	Intake	High Density Lipoprotein	−0.13	[−0.29; 0.04]	−0.09	[−0.32; 0.13]	−0.04	[−0.32; 0.24]	20.2%
Thorne−Lyman 2012	Vitamin D	Supplementation	Supplementation + Intake	Mean Birthweight	0.13	[−0.12; 0.39]	0.11	[0.01; 0.21]	0.02	[−0.25; 0.29]	20.5%
**Random effects model**									**−0.09**	**[−0.26; 0.09]**	**100.0%**
**Prediction interval**										**[−0.55; 0.38]**	

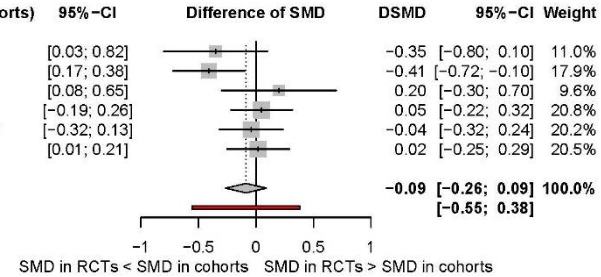

Supplementary Fig. S1 Forest plot, analysis of BoE-pairs with continuous outcomes and standardised mean difference

Reference	Intervention/Exposure in SR	Type of intake (RCTs)	Type of exposure (cohorts)	Outcome in SR	RR (RCTs)	95%-CI	RR (cohorts)	95%-CI	RRR	95%-CI
**Micronutrients**										
Aburto 2013	Low-sodium	Intake	Intake	All-cause mortality	0.70	[0.44; 1.14]	0.95	[0.71; 1.27]	0.74	[0.42; 1.29]
Aburto 2013	Low-sodium	Intake	Intake	Cardiovascular disease	0.84	[0.57; 1.23]	0.87	[0.64; 1.18]	0.97	[0.59; 1.58]
Aguilar-Cordero 2020	Vitamin D	Supplementation	Status	Pre-eclampsia	0.66	[0.42; 1.05]	0.82	[0.60; 1.11]	0.80	[0.46; 1.40]
Bolland 2015	Calcium	Supplementation	Supplementation	Fractures	0.89	[0.81; 0.96]	1.02	[0.93; 1.11]	0.87	[0.77; 0.99]
Chowdhury 2014b	Vitamin D	Supplementation	Status	All-cause mortality	0.98	[0.94; 1.03]	0.69	[0.65; 0.75]	1.42	[1.30; 1.55]
Chung 2011	Vitamin D	Supplementation	Status	Colorectal cancer	1.02	[0.60; 1.74]	0.94	[0.91; 0.97]	1.09	[0.64; 1.85]
Chung 2011	Vitamin D	Supplementation	Status	Breast cancer	0.99	[0.25; 3.99]	0.99	[0.97; 1.01]	1.00	[0.25; 4.00]
Feng 2015	Folic acid	Supplementation	Supplementation	Congenital heart defects	0.58	[0.33; 1.02]	0.60	[0.38; 0.96]	0.97	[0.47; 2.01]
Fu 2021	Vitamin C	Supplementation	Supplementation	Lung cancer	1.30	[0.68; 2.48]	1.02	[0.85; 1.23]	1.27	[0.65; 2.50]
Fu 2021	Vitamin C	Supplementation	Supplementation	Breast cancer	1.11	[0.87; 1.41]	1.02	[0.88; 1.18]	1.09	[0.82; 1.44]
Fu 2021	Vitamin C	Supplementation	Supplementation	Colorectal cancer	0.84	[0.64; 1.10]	0.80	[0.42; 1.52]	1.05	[0.52; 2.11]
Jiang 2019	Vitamin E	Supplementation	Intake	Age-related cataract	0.97	[0.91; 1.03]	0.90	[0.80; 1.00]	1.08	[0.95; 1.22]
Jiang 2019	β-Carotene	Supplementation	Intake	Age-related cataract	0.99	[0.92; 1.07]	0.90	[0.83; 0.99]	1.10	[0.98; 1.24]
Jonker 2020	Folic acid	Supplementation	Supplementation	Low birthweight	0.96	[0.88; 1.04]	0.71	[0.60; 0.83]	1.35	[1.13; 1.62]
Jonker 2020	Folic acid	Supplementation	Supplementation+Intake	Small for gestational age	0.98	[0.92; 1.06]	0.80	[0.50; 1.11]	1.22	[0.82; 1.84]
Kim 2018	Multivitamin/Minerals	Supplementation	Supplementation	Mortality	0.95	[0.83; 1.09]	1.00	[0.96; 1.05]	0.95	[0.82; 1.10]
Kim 2018	Multivitamin/Minerals	Supplementation	Supplementation	Coronary heart disease	0.97	[0.79; 1.19]	0.86	[0.76; 0.97]	1.13	[0.89; 1.43]
Kim 2018	Multivitamin/Minerals	Supplementation	Supplementation	Stroke	1.05	[0.70; 1.56]	0.98	[0.91; 1.04]	1.07	[0.71; 1.61]
Kong 2014	Vitamins	Supplementation	Intake	Gastric cancer	1.11	[0.94; 1.31]	0.85	[0.66; 1.08]	1.31	[0.97; 1.76]
Lin 2019	Vitamin E	Supplementation	Intake	Bladder cancer	1.10	[0.86; 1.35]	0.84	[0.72; 0.96]	1.31	[1.00; 1.71]
Lin 2020	Vitamin D	Supplementation	Supplementation	Nephrolithiasis	0.84	[0.65; 1.10]	1.24	[1.01; 1.51]	0.68	[0.49; 0.94]
Lin 2020	Calcium	Supplementation	Supplementation	Nephrolithiasis	1.20	[0.65; 2.21]	1.16	[1.00; 1.35]	1.03	[0.55; 1.94]
Moazzen 2018	Folic acid	Supplementation	Supplementation	Colorectal cancer	1.07	[0.86; 1.43]	0.96	[0.76; 1.21]	1.11	[0.79; 1.57]
Mocellin 2017	Vitamin B6	Supplementation	Intake	Cancer	1.03	[0.94; 1.13]	0.95	[0.90; 1.01]	1.08	[0.97; 1.21]
Sayehmiri 2018	Selenium	Supplementation	Supplementation+Intake	Prostate cancer	0.90	[0.74; 1.09]	0.89	[0.64; 1.23]	1.01	[0.69; 1.48]
Stratton 2011	Multivitamin	Supplementation	Supplementation	Prostate cancer	0.98	[0.81; 1.20]	1.05	[0.94; 1.16]	0.93	[0.75; 1.17]
Stratton 2011	Vitamin E	Supplementation	Supplementation	Prostate cancer	1.01	[0.90; 1.13]	1.09	[0.94; 1.27]	0.93	[0.77; 1.12]
Stratton 2011	Vitamin C	Supplementation	Supplementation	Prostate cancer	1.10	[0.93; 1.29]	0.96	[0.86; 1.06]	1.15	[0.94; 1.39]
Stratton 2011	β-Carotene	Supplementation	Supplementation	Prostate cancer	1.19	[0.87; 1.65]	0.70	[0.63; 0.78]	1.70	[1.21; 2.38]
Trikalinos 2012	Vitamin E	Supplementation	Supplementation+Intake	Mortality	0.97	[0.91; 1.03]	0.85	[0.78; 0.93]	1.14	[1.02; 1.27]
Vinceti 2018a	Selenium	Supplementation	Status	Cancer	0.99	[0.86; 1.14]	0.75	[0.59; 0.94]	1.32	[1.01; 1.73]
Vinceti 2018a	Selenium	Supplementation	Intake	Mortality	0.81	[0.49; 1.32]	0.93	[0.83; 1.04]	0.87	[0.52; 1.45]
Vinceti 2018b	Selenium	Supplementation	Intake	Type 2 diabetes	1.11	[1.01; 1.22]	2.28	[1.31; 3.89]	0.49	[0.28; 0.85]
Wien 2012	Folic acid	Supplementation	Supplementation	Cancer	1.07	[1.00; 1.14]	1.03	[0.90; 1.18]	1.04	[0.89; 1.21]
Wolf 2017	Multivitamin	Supplementation	Supplementation	Preterm birth	1.09	[0.43; 2.77]	0.84	[0.69; 1.03]	1.30	[0.50; 3.36]
Wolf 2017	Multivitamin	Supplementation	Supplementation	Stillbirth	2.43	[0.12; 50.05]	0.78	[0.59; 1.03]	3.12	[0.15; 64.44]
Yang 2016	Folic acid	Supplementation	Supplementation+Intake	Gestational hypertension	0.62	[0.04; 0.94]	1.05	[1.00; 1.11]	0.59	[0.38; 0.91]
Yang 2020	Calcium	Supplementation	Intake	Cardiovascular disease	0.91	[0.56; 1.46]	0.98	[0.90; 1.06]	0.93	[0.57; 1.51]
Yao 2019	Vitamin D	Supplementation	Status	Fractures	1.06	[0.97; 1.15]	0.89	[0.83; 0.96]	1.19	[1.06; 1.33]
Yu 2021	Folic acid	Supplementation	Supplementation+Intake	Pre-eclampsia	1.11	[0.75; 1.62]	0.85	[0.76; 0.94]	1.31	[0.88; 1.95]
Zhang 2016	Selenium	Supplementation	Status	Cardiovascular disease	0.91	[0.74; 1.10]	0.87	[0.76; 0.99]	1.05	[0.82; 1.33]
Zhang 2018	Vitamin D	Supplementation	Status	Gestational diabetes mellitus	0.72	[0.39; 1.31]	0.58	[0.44; 0.76]	1.24	[0.64; 2.40]
Zhao 2014	Multivitamin/Minerals	Supplementation	Supplementation	Nuclear cataract	0.73	[0.59; 0.90]	0.73	[0.64; 0.82]	1.00	[0.78; 1.28]
Zhao 2014	Multivitamin/Minerals	Supplementation	Supplementation	Cortical cataract	0.92	[0.69; 1.23]	0.81	[0.68; 0.94]	1.14	[0.82; 1.58]
Zhao 2014	Multivitamin/Minerals	Supplementation	Supplementation	Posterior subcapsular cataract	1.81	[1.30; 2.54]	0.96	[0.72; 1.20]	1.89	[1.24; 2.87]
Zhou 2017	Vitamin D	Supplementation	Status	Preterm birth	0.58	[0.30; 1.11]	0.88	[0.73; 1.05]	0.66	[0.33; 1.30]
**Random effects model**									1.08	[1.02; 1.15]
**Prediction interval**										[0.81; 1.45]
Heterogeneity: $I^2 = 62\%$, $\tau^2 = 0.0201$, $p < 0.01$										
**Food group**										
Afshin 2014	Nuts	Intake	Intake	Coronary heart disease	0.79	[0.55; 1.14]	0.76	[0.69; 0.83]	1.04	[0.71; 1.51]
Afshin 2014	Nuts	Intake	Intake	Stroke	0.62	[0.44; 0.87]	0.95	[0.83; 1.09]	0.65	[0.45; 0.94]
Afshin 2014	Nuts	Intake	Intake	Type 2 diabetes	0.86	[0.68; 1.08]	0.87	[0.80; 0.95]	0.99	[0.77; 1.27]
Martinez-Gonzalez 2014b	Olive oil	Supplementation+Intake	Intake	Coronary heart disease	0.81	[0.53; 1.24]	0.96	[0.78; 1.18]	0.84	[0.53; 1.35]
Martinez-Gonzalez 2014b	Olive oil	Supplementation+Intake	Intake	Stroke	0.69	[0.48; 0.98]	0.74	[0.60; 0.92]	0.93	[0.62; 1.41]
Schwingshackl 2017	Olive oil	Intake	Intake	Type 2 diabetes	0.60	[0.43; 0.84]	0.87	[0.80; 0.94]	0.69	[0.49; 0.97]
**Random effects model**									0.86	[0.73; 1.00]
**Prediction interval**										[0.63; 1.17]
Heterogeneity: $I^2 = 18\%$, $\tau^2 = 0.0062$, $p = 0.30$										
**Macronutrients**										
Alexander 2017	Omega-3 fatty acids	Intake+Supplementation	Intake	Cardiovascular disease	0.94	[0.85; 1.05]	0.82	[0.74; 0.92]	1.15	[0.98; 1.33]
Chowdhury 2012	Omega-3 fatty acids	Supplementation	Intake	Stroke	0.98	[0.89; 1.08]	0.90	[0.80; 1.01]	1.09	[0.94; 1.27]
Chowdhury 2014a	α-Linolenic acid	Supplementation	Intake	Coronary heart disease	0.97	[0.69; 1.36]	0.99	[0.86; 1.14]	0.98	[0.68; 1.41]
Chowdhury 2014a	Omega-6 fatty acids	Intake+Supplementation	Intake	Coronary heart disease	0.86	[0.69; 1.07]	0.98	[0.90; 1.06]	0.88	[0.69; 1.11]
**Random effects model**									1.06	[0.94; 1.18]
**Prediction interval**										[0.75; 1.48]
Heterogeneity: $I^2 = 21\%$, $\tau^2 = 0.0029$, $p = 0.28$										
**Green tea**										
Filippini 2020	Green tea	Supplementation	Intake	Prostate cancer	0.50	[0.18; 1.36]	1.09	[0.89; 1.32]	0.46	[0.16; 1.29]
Filippini 2020	Green tea	Supplementation	Intake	Endometrial cancer	0.33	[0.01; 8.15]	0.75	[0.43; 1.30]	0.44	[0.01; 13.14]
**Random effects model**									0.46	[0.17; 1.23]
**Prediction interval**										
Heterogeneity: $I^2 = 0\%$, $\tau^2 = 0$, $p = 0.98$										
**Dietary pattern**										
Grosso 2015	Mediterranean diet	Intake	Intake	Cardiovascular disease	0.55	[0.39; 0.76]	0.71	[0.65; 0.78]	0.77	[0.55; 1.09]
Johnston 2019	Low red meat	Intake	Intake	Mortality	1.00	[0.84; 1.19]	0.86	[0.79; 0.94]	1.16	[0.96; 1.41]
Johnston 2019	Low red meat	Intake	Intake	Type 2 diabetes	0.95	[0.89; 1.02]	0.76	[0.68; 0.86]	1.25	[1.09; 1.43]
Johnston 2019	Low red meat	Intake	Intake	Colorectal cancer	1.02	[0.89; 1.18]	0.94	[0.85; 1.05]	1.09	[0.91; 1.29]
Kastorini 2011	Mediterranean diet	Intake	Intake	Metabolic syndrome	0.45	[0.24; 0.84]	0.46	[0.11; 1.88]	0.98	[0.21; 4.62]
Morze 2021	Mediterranean diet	Intake	Intake	Mortality	0.75	[0.17; 3.33]	0.87	[0.82; 0.92]	0.86	[0.19; 3.82]
Morze 2021	Mediterranean diet	Intake	Intake	Breast cancer	0.41	[0.19; 0.87]	0.97	[0.94; 1.00]	0.42	[0.20; 0.91]
Schwingshackl 2015	Mediterranean diet	Intake	Intake	Type 2 diabetes	0.70	[0.54; 0.91]	0.83	[0.74; 0.92]	0.84	[0.64; 1.12]
**Random effects model**									0.99	[0.81; 1.20]
**Prediction interval**										[0.57; 1.70]
Heterogeneity: $I^2 = 59\%$, $\tau^2 = 0.0400$, $p = 0.02$										
Test for subgroup differences: $\chi^2_4 = 10.29$, df = 4 ($p = 0.04$)										

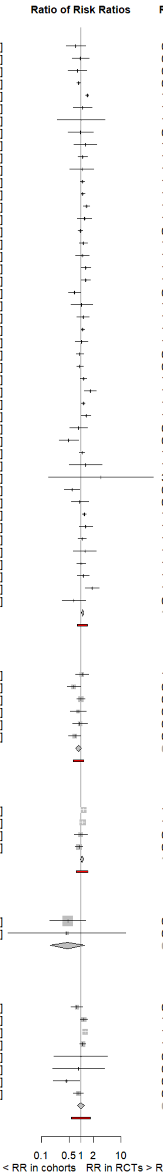

Supplementary Fig. S2 Forest plot, subgroup analysis of BoE-pairs with binary outcomes by type of intervention/exposure

Reference	Intervention/Exposure in SR	Outcome in SR	RR (RCTs)	95%-CI	RR (cohorts)	95%-CI	RRR	95%-CI
**Intake vs. Intake**								
Aburto 2013	Low-sodium	All-cause mortality	0.70	[0.44; 1.14]	0.95	[0.71; 1.27]	0.74	[0.42; 1.29]
Aburto 2013	Low-sodium	Cardiovascular disease	0.84	[0.57; 1.23]	0.87	[0.64; 1.18]	0.97	[0.59; 1.58]
Afshin 2014	Nuts	Coronary heart disease	0.79	[0.55; 1.14]	0.76	[0.69; 0.83]	1.04	[0.71; 1.51]
Afshin 2014	Nuts	Stroke	0.62	[0.44; 0.87]	0.95	[0.83; 1.09]	0.65	[0.45; 0.94]
Afshin 2014	Nuts	Type 2 diabetes	0.86	[0.68; 1.08]	0.87	[0.80; 0.95]	0.99	[0.77; 1.27]
Grosso 2015	Mediterranean diet	Cardiovascular disease	0.55	[0.39; 0.76]	0.71	[0.65; 0.78]	0.77	[0.55; 1.09]
Johnston 2019	Low red meat	Mortality	1.00	[0.84; 1.19]	0.86	[0.79; 0.94]	1.16	[0.96; 1.41]
Johnston 2019	Low red meat	Type 2 diabetes	0.95	[0.89; 1.02]	0.76	[0.68; 0.86]	1.25	[1.09; 1.43]
Johnston 2019	Low red meat	Colorectal cancer	1.02	[0.89; 1.18]	0.94	[0.85; 1.05]	1.09	[0.91; 1.29]
Kastorini 2011	Mediterranean diet	Metabolic syndrome	0.45	[0.24; 0.84]	0.46	[0.11; 1.88]	0.98	[0.21; 4.62]
Morze 2021	Mediterranean diet	Mortality	0.75	[0.17; 3.33]	0.87	[0.82; 0.92]	0.86	[0.19; 3.82]
Morze 2021	Mediterranean diet	Breast cancer	0.41	[0.19; 0.87]	0.97	[0.94; 1.00]	0.42	[0.20; 0.91]
Schwingshackl 2015	Mediterranean diet	Type 2 diabetes	0.70	[0.54; 0.91]	0.83	[0.74; 0.92]	0.84	[0.64; 1.12]
Schwingshackl 2017	Olive oil	Type 2 diabetes	0.60	[0.43; 0.84]	0.87	[0.80; 0.94]	0.69	[0.49; 0.97]
Random effects model							0.93	[0.81; 1.06]
Prediction interval								[0.62; 1.39]
Heterogeneity: I^2 = 60%, τ^2 = 0.0288, p < 0.01								
**Supplementation vs. Supplementation**								
Bolland 2015	Calcium	Fractures	0.89	[0.81; 0.96]	1.02	[0.93; 1.11]	0.87	[0.77; 0.99]
Feng 2015	Folic acid	Congenital heart defects	0.58	[0.33; 1.02]	0.60	[0.38; 0.96]	0.97	[0.47; 2.01]
Fu 2021	Vitamin C	Lung cancer	1.30	[0.68; 2.48]	1.02	[0.85; 1.23]	1.27	[0.65; 2.50]
Fu 2021	Vitamin C	Breast cancer	1.11	[0.87; 1.41]	1.02	[0.88; 1.18]	1.09	[0.82; 1.44]
Fu 2021	Vitamin C	Colorectal cancer	0.84	[0.64; 1.10]	0.80	[0.42; 1.52]	1.05	[0.52; 2.11]
Jonker 2020	Folic acid	Low birthweight	0.96	[0.88; 1.04]	0.71	[0.60; 0.83]	1.35	[1.13; 1.62]
Kim 2018	Multivitamin/Minerals	Mortality	0.95	[0.83; 1.09]	1.00	[0.96; 1.05]	0.95	[0.82; 1.10]
Kim 2018	Multivitamin/Minerals	Coronary heart disease	0.97	[0.79; 1.19]	0.86	[0.76; 0.97]	1.13	[0.89; 1.43]
Kim 2018	Multivitamin/Minerals	Stroke	1.05	[0.70; 1.56]	0.98	[0.91; 1.04]	1.07	[0.71; 1.61]
Lin 2020	Vitamin D	Nephrolithiasis	0.84	[0.65; 1.10]	1.24	[1.01; 1.51]	0.68	[0.49; 0.94]
Lin 2020	Calcium	Nephrolithiasis	1.20	[0.65; 2.21]	1.16	[1.00; 1.35]	1.03	[0.55; 1.94]
Moazzen 2018	Folic acid	Colorectal cancer	1.07	[0.86; 1.43]	0.96	[0.76; 1.21]	1.11	[0.79; 1.57]
Stratton 2011	Multivitamin	Prostate cancer	0.98	[0.81; 1.20]	1.05	[0.94; 1.16]	0.93	[0.75; 1.17]
Stratton 2011	Vitamin E	Prostate cancer	1.01	[0.90; 1.13]	1.09	[0.94; 1.27]	0.93	[0.77; 1.12]
Stratton 2011	Vitamin C	Prostate cancer	1.10	[0.93; 1.29]	0.96	[0.86; 1.06]	1.15	[0.94; 1.39]
Stratton 2011	β-Carotene	Prostate cancer	1.19	[0.87; 1.65]	0.70	[0.63; 0.78]	1.70	[1.21; 2.38]
Wien 2012	Folic acid	Cancer	1.07	[1.00; 1.14]	1.03	[0.90; 1.18]	1.04	[0.89; 1.21]
Wolf 2017	Multivitamin	Preterm birth	1.09	[0.43; 2.77]	0.84	[0.69; 1.03]	1.30	[0.50; 3.36]
Wolf 2017	Multivitamin	Stillbirth	2.43	[0.12; 50.05]	0.78	[0.59; 1.03]	3.12	[0.15; 64.44]
Zhao 2014	Multivitamin/Minerals	Nuclear cataract	0.73	[0.59; 0.90]	0.73	[0.64; 0.82]	1.00	[0.78; 1.28]
Zhao 2014	Multivitamin/Minerals	Cortical cataract	0.92	[0.69; 1.23]	0.81	[0.68; 0.94]	1.14	[0.82; 1.58]
Zhao 2014	Multivitamin/Minerals	Posterior subcapsular cataract	1.81	[1.30; 2.54]	0.96	[0.72; 1.20]	1.89	[1.24; 2.87]
Random effects model							1.07	[0.98; 1.16]
Prediction interval								[0.79; 1.44]
Heterogeneity: I^2 = 53%, τ^2 = 0.0187, p < 0.01								
**Intake + Supplementation vs. Intake**								
Alexander 2017	Omega-3 fatty acids	Cardiovascular disease	0.94	[0.85; 1.05]	0.82	[0.74; 0.92]	1.15	[0.98; 1.33]
Chowdhury 2014a	Omega-6 fatty acids	Coronary heart disease	0.86	[0.69; 1.07]	0.98	[0.90; 1.06]	0.88	[0.69; 1.11]
Martinez-Gonzalez 2014b	Olive oil	Coronary heart disease	0.81	[0.53; 1.24]	0.96	[0.78; 1.18]	0.84	[0.53; 1.35]
Martinez-Gonzalez 2014b	Olive oil	Stroke	0.69	[0.48; 0.98]	0.74	[0.60; 0.92]	0.93	[0.62; 1.41]
Random effects model							1.01	[0.87; 1.17]
Prediction interval								[0.63; 1.60]
Heterogeneity: I^2 = 35%, τ^2 = 0.0057, p = 0.20								
**Supplementation vs. Intake**								
Chowdhury 2012	Omega-3 fatty acids	Stroke	0.98	[0.89; 1.08]	0.90	[0.80; 1.01]	1.09	[0.94; 1.27]
Chowdhury 2014a	α-Linolenic acid	Coronary heart disease	0.97	[0.69; 1.36]	0.99	[0.86; 1.14]	0.98	[0.68; 1.41]
Filippini 2020	Green tea	Prostate cancer	0.50	[0.18; 1.36]	1.09	[0.89; 1.32]	0.46	[0.16; 1.29]
Filippini 2020	Green tea	Endometrial cancer	0.33	[0.01; 8.15]	0.75	[0.43; 1.30]	0.44	[0.01; 13.14]
Jiang 2019	Vitamin E	Age-related cataract	0.97	[0.91; 1.03]	0.90	[0.80; 1.00]	1.08	[0.95; 1.22]
Jiang 2019	β-Carotene	Age-related cataract	0.99	[0.92; 1.07]	0.90	[0.83; 0.99]	1.10	[0.98; 1.24]
Kong 2014	Vitamins	Gastric cancer	1.11	[0.94; 1.31]	0.85	[0.66; 1.08]	1.31	[0.97; 1.76]
Lin 2019	Vitamin E	Bladder cancer	1.10	[0.86; 1.35]	0.84	[0.72; 0.96]	1.31	[1.00; 1.71]
Mocellin 2017	Vitamin B6	Cancer	1.03	[0.94; 1.13]	0.95	[0.90; 1.01]	1.08	[0.97; 1.21]
Vinceti 2018a	Selenium	Mortality	0.81	[0.49; 1.32]	0.93	[0.83; 1.04]	0.87	[0.52; 1.45]
Vinceti 2018b	Selenium	Type 2 diabetes	1.11	[1.01; 1.22]	2.28	[1.31; 3.89]	0.49	[0.28; 0.85]
Yang 2020	Calcium	Cardiovascular disease	0.91	[0.56; 1.46]	0.98	[0.90; 1.06]	0.93	[0.57; 1.51]
Random effects model							1.05	[0.92; 1.19]
Prediction interval								[0.71; 1.54]
Heterogeneity: I^2 = 31%, τ^2 = 0.0259, p = 0.14								
**Supplementation vs. Intake + Supplementation**								
Jonker 2020	Folic acid	Small for gestational age	0.98	[0.92; 1.06]	0.80	[0.50; 1.11]	1.22	[0.82; 1.84]
Sayehmiri 2018	Selenium	Prostate cancer	0.90	[0.74; 1.09]	0.89	[0.64; 1.23]	1.01	[0.69; 1.48]
Trikalinos 2012	Vitamin E	Mortality	0.97	[0.91; 1.03]	0.85	[0.78; 0.93]	1.14	[1.02; 1.27]
Yang 2016	Folic acid	Gestational hypertension	0.62	[0.04; 0.94]	1.05	[1.00; 1.11]	0.59	[0.38; 0.91]
Yu 2021	Folic acid	Pre-eclampsia	1.11	[0.75; 1.62]	0.85	[0.76; 0.94]	1.31	[0.88; 1.95]
Random effects model							1.04	[0.80; 1.34]
Prediction interval								[0.44; 2.47]
Heterogeneity: I^2 = 58%, τ^2 = 0.0568, p = 0.05								
**Supplementation vs. Status**								
Aguilar-Cordero 2020	Vitamin D	Pre-eclampsia	0.66	[0.42; 1.05]	0.82	[0.60; 1.11]	0.80	[0.46; 1.40]
Chowdhury 2014b	Vitamin D	All-cause mortality	0.98	[0.94; 1.03]	0.69	[0.65; 0.75]	1.42	[1.30; 1.55]
Chung 2011	Vitamin D	Colorectal cancer	1.02	[0.60; 1.74]	0.94	[0.91; 0.97]	1.09	[0.64; 1.85]
Chung 2011	Vitamin D	Breast cancer	0.99	[0.25; 3.99]	0.99	[0.97; 1.01]	1.00	[0.25; 4.00]
Vinceti 2018a	Selenium	Cancer	0.99	[0.86; 1.14]	0.75	[0.59; 0.94]	1.32	[1.01; 1.73]
Yao 2019	Vitamin D	Fractures	1.06	[0.97; 1.15]	0.89	[0.83; 0.96]	1.19	[1.06; 1.33]
Zhang 2016	Selenium	Cardiovascular disease	0.91	[0.74; 1.10]	0.87	[0.76; 0.99]	1.05	[0.82; 1.33]
Zhang 2018	Vitamin D	Gestational diabetes mellitus	0.72	[0.39; 1.31]	0.58	[0.44; 0.76]	1.24	[0.64; 2.40]
Zhou 2017	Vitamin D	Preterm birth	0.58	[0.30; 1.11]	0.88	[0.73; 1.05]	0.66	[0.33; 1.30]
Random effects model							1.20	[1.06; 1.36]
Prediction interval								[0.90; 1.60]
Heterogeneity: I^2 = 53%, τ^2 = 0.0108, p = 0.03								
Test for subgroup differences: χ^2_5 = 8.35, df = 5 (p = 0.14)								

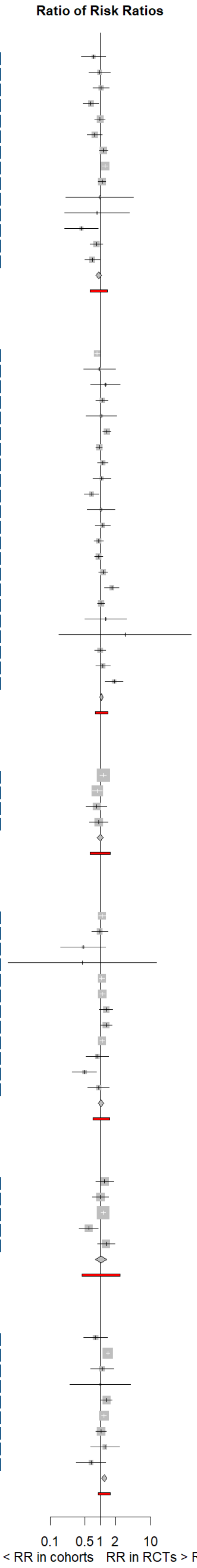

Supplementary Fig. S3 Forest plot, subgroup analysis of BoE-pairs with binary outcomes by type of intake/exposure

Reference	Intervention/Exposure in SR	Type of intake (RCTs)	Type of exposure (cohorts)	Outcome in SR	RR (RCTs)	95%-CI	RR (cohorts)	95%-CI	RRR	95%-CI
**All-cause mortality**										
Aburto 2013	Low-sodium	Intake	Intake	All-cause mortality	0.70	[0.44; 1.14]	0.95	[0.71; 1.27]	0.74	[0.42; 1.29]
Chowdhury 2014b	Vitamin D	Supplementation	Status	All-cause mortality	0.98	[0.94; 1.03]	0.69	[0.65; 0.75]	1.42	[1.30; 1.55]
Random effects model									1.09	[0.58; 2.04]
Prediction interval										
Heterogeneity: $I^2 = 81\%$, $\tau^2 = 0.1739$, $p = 0.02$										
**Cardiovascular disease**										
Aburto 2013	Low-sodium	Intake	Intake	Cardiovascular disease	0.84	[0.57; 1.23]	0.87	[0.64; 1.18]	0.97	[0.59; 1.58]
Afshin 2014	Nuts	Intake	Intake	Coronary heart disease	0.79	[0.55; 1.14]	0.76	[0.69; 0.83]	1.04	[0.71; 1.51]
Afshin 2014	Nuts	Intake	Intake	Stroke	0.62	[0.44; 0.87]	0.95	[0.83; 1.09]	0.65	[0.45; 0.94]
Alexander 2017	Omega-3 fatty acids	Intake+Supplementation	Intake	Cardiovascular disease	0.94	[0.85; 1.05]	0.82	[0.74; 0.92]	1.15	[0.98; 1.33]
Chowdhury 2012	Omega-3 fatty acids	Supplementation	Intake	Stroke	0.98	[0.89; 1.08]	0.90	[0.80; 1.01]	1.09	[0.94; 1.27]
Chowdhury 2014a	α-Linolenic acid	Supplementation	Intake	Coronary heart disease	0.97	[0.69; 1.36]	0.99	[0.86; 1.14]	0.98	[0.68; 1.41]
Chowdhury 2014a	Omega-6 fatty acids	Intake+Supplementation	Intake	Coronary heart disease	0.86	[0.69; 1.07]	0.98	[0.90; 1.06]	0.88	[0.69; 1.11]
Grosso 2015	Mediterranean diet	Intake	Intake	Cardiovascular disease	0.55	[0.39; 0.76]	0.71	[0.65; 0.78]	0.77	[0.55; 1.09]
Johnston 2019	Low red meat	Intake	Intake	Mortality	1.00	[0.84; 1.19]	0.86	[0.79; 0.94]	1.16	[0.96; 1.41]
Kim 2018	Multivitamin/Minerals	Supplementation	Supplementation	Mortality	0.95	[0.83; 1.09]	1.00	[0.96; 1.05]	0.95	[0.82; 1.10]
Kim 2018	Multivitamin/Minerals	Supplementation	Supplementation	Coronary heart disease	0.97	[0.79; 1.19]	0.86	[0.76; 0.97]	1.13	[0.89; 1.43]
Kim 2018	Multivitamin/Minerals	Supplementation	Supplementation	Stroke	1.05	[0.70; 1.56]	0.98	[0.91; 1.04]	1.07	[0.71; 1.61]
Martinez-Gonzalez 2014b	Olive oil	Supplementation+Intake	Intake	Coronary heart disease	0.81	[0.53; 1.24]	0.96	[0.78; 1.18]	0.84	[0.53; 1.35]
Martinez-Gonzalez 2014b	Olive oil	Supplementation+Intake	Intake	Stroke	0.69	[0.48; 0.98]	0.74	[0.60; 0.92]	0.93	[0.62; 1.41]
Trikalinos 2012	Vitamin E	Supplementation	Supplementation+Intake	Mortality	0.97	[0.91; 1.03]	0.85	[0.78; 0.93]	1.14	[1.02; 1.27]
Yang 2020	Calcium	Supplementation	Intake	Cardiovascular disease	0.91	[0.56; 1.46]	0.98	[0.90; 1.06]	0.93	[0.57; 1.51]
Zhang 2016	Selenium	Supplementation	Status	Cardiovascular disease	0.91	[0.74; 1.10]	0.87	[0.76; 0.99]	1.05	[0.82; 1.33]
Random effects model									1.03	[0.97; 1.10]
Prediction interval										[0.90; 1.19]
Heterogeneity: $I^2 = 22\%$, $\tau^2 = 0.0033$, $p = 0.20$										
**Endocrine / metabolic disease**										
Afshin 2014	Nuts	Intake	Intake	Type 2 diabetes	0.86	[0.68; 1.08]	0.87	[0.80; 0.95]	0.99	[0.77; 1.27]
Johnston 2019	Low red meat	Intake	Intake	Type 2 diabetes	0.95	[0.89; 1.02]	0.76	[0.68; 0.86]	1.25	[1.09; 1.43]
Kastorini 2011	Mediterranean diet	Intake	Intake	Metabolic syndrome	0.45	[0.24; 0.84]	0.46	[0.11; 1.88]	0.98	[0.21; 4.62]
Lin 2020	Vitamin D	Supplementation	Supplementation	Nephrolithiasis	0.84	[0.65; 1.10]	1.24	[1.01; 1.51]	0.68	[0.49; 0.94]
Lin 2020	Calcium	Supplementation	Supplementation	Nephrolithiasis	1.20	[0.65; 2.21]	1.16	[1.00; 1.35]	1.03	[0.55; 1.94]
Schwingshackl 2015	Mediterranean diet	Intake	Intake	Type 2 diabetes	0.70	[0.54; 0.91]	0.83	[0.74; 0.92]	0.84	[0.64; 1.12]
Schwingshackl 2017	Olive oil	Intake	Intake	Type 2 diabetes	0.60	[0.43; 0.84]	0.87	[0.80; 0.94]	0.69	[0.49; 0.97]
Vinceti 2018b	Selenium	Supplementation	Intake	Type 2 diabetes	1.11	[1.01; 1.22]	2.28	[1.31; 3.89]	0.49	[0.28; 0.85]
Random effects model									0.86	[0.70; 1.06]
Prediction interval										[0.47; 1.57]
Heterogeneity: $I^2 = 75\%$, $\tau^2 = 0.0492$, $p < 0.01$										
**Pregnancy outcomes**										
Aguilar-Cordero 2020	Vitamin D	Supplementation	Status	Pre-eclampsia	0.66	[0.42; 1.05]	0.82	[0.60; 1.11]	0.80	[0.46; 1.40]
Feng 2015	Folic acid	Supplementation	Supplementation	Congenital heart defects	0.58	[0.33; 1.02]	0.60	[0.38; 0.96]	0.97	[0.47; 2.01]
Jonker 2020	Folic acid	Supplementation	Supplementation	Low birthweight	0.96	[0.88; 1.04]	0.71	[0.60; 0.83]	1.35	[1.13; 1.62]
Jonker 2020	Folic acid	Supplementation	Supplementation+Intake	Small for gestational age	0.98	[0.92; 1.06]	0.80	[0.50; 1.11]	1.22	[0.82; 1.84]
Wolf 2017	Multivitamin	Supplementation	Supplementation	Preterm birth	1.09	[0.43; 2.77]	0.84	[0.69; 1.03]	1.30	[0.50; 3.36]
Wolf 2017	Multivitamin	Supplementation	Supplementation	Stillbirth	2.43	[0.12; 50.05]	0.78	[0.59; 1.03]	3.12	[0.15; 64.44]
Yang 2016	Folic acid	Supplementation	Supplementation+Intake	Gestational hypertension	0.62	[0.04; 0.94]	1.05	[1.00; 1.11]	0.59	[0.38; 0.91]
Yu 2021	Folic acid	Supplementation	Supplementation+Intake	Pre-eclampsia	1.11	[0.75; 1.62]	0.85	[0.76; 0.94]	1.31	[0.88; 1.95]
Zhang 2018	Vitamin D	Supplementation	Status	Gestational diabetes mellitus	0.72	[0.39; 1.31]	0.58	[0.44; 0.76]	1.24	[0.64; 2.40]
Zhou 2017	Vitamin D	Supplementation	Status	Preterm birth	0.58	[0.30; 1.11]	0.88	[0.73; 1.05]	0.66	[0.33; 1.30]
Random effects model									1.05	[0.85; 1.29]
Prediction interval										[0.63; 1.75]
Heterogeneity: $I^2 = 49\%$, $\tau^2 = 0.0375$, $p = 0.04$										
**Fractures**										
Bolland 2015	Calcium	Supplementation	Supplementation	Fractures	0.89	[0.81; 0.96]	1.02	[0.93; 1.11]	0.87	[0.77; 0.99]
Yao 2019	Vitamin D	Supplementation	Status	Fractures	1.06	[0.97; 1.15]	0.89	[0.83; 0.96]	1.19	[1.06; 1.33]
Random effects model									1.02	[0.75; 1.38]
Prediction interval										
Heterogeneity: $I^2 = 93\%$, $\tau^2 = 0.0448$, $p < 0.01$										
**Cancer**										
Chung 2011	Vitamin D	Supplementation	Status	Colorectal cancer	1.02	[0.60; 1.74]	0.94	[0.91; 0.97]	1.09	[0.64; 1.85]
Chung 2011	Vitamin D	Supplementation	Status	Breast cancer	0.99	[0.25; 3.99]	0.99	[0.97; 1.01]	1.00	[0.25; 4.00]
Filippini 2020	Green tea	Supplementation	Intake	Prostate cancer	0.50	[0.18; 1.36]	1.09	[0.89; 1.32]	0.46	[0.16; 1.29]
Filippini 2020	Green tea	Supplementation	Intake	Endometrial cancer	0.33	[0.01; 8.15]	0.75	[0.43; 1.30]	0.44	[0.01; 13.14]
Fu 2021	Vitamin C	Supplementation	Supplementation	Lung cancer	1.30	[0.68; 2.48]	1.02	[0.85; 1.23]	1.27	[0.65; 2.50]
Fu 2021	Vitamin C	Supplementation	Supplementation	Breast cancer	1.11	[0.87; 1.41]	1.02	[0.88; 1.18]	1.09	[0.82; 1.44]
Fu 2021	Vitamin C	Supplementation	Supplementation	Colorectal cancer	0.84	[0.64; 1.10]	0.80	[0.42; 1.52]	1.05	[0.52; 2.11]
Johnston 2019	Low red meat	Intake	Intake	Colorectal cancer	1.02	[0.89; 1.18]	0.94	[0.85; 1.05]	1.09	[0.91; 1.29]
Kong 2014	Vitamins	Supplementation	Intake	Gastric cancer	1.11	[0.94; 1.31]	0.85	[0.66; 1.08]	1.31	[0.97; 1.76]
Lin 2019	Vitamin E	Supplementation	Intake	Bladder cancer	1.10	[0.86; 1.35]	0.84	[0.72; 0.96]	1.31	[1.00; 1.71]
Moazzen 2018	Folic acid	Supplementation	Supplementation	Colorectal cancer	1.07	[0.86; 1.43]	0.96	[0.76; 1.21]	1.11	[0.79; 1.57]
Mocellin 2017	Vitamin B6	Supplementation	Intake	Cancer	1.03	[0.94; 1.13]	0.95	[0.90; 1.01]	1.08	[0.97; 1.21]
Morze 2021	Mediterranean diet	Intake	Intake	Mortality	0.75	[0.17; 3.33]	0.87	[0.82; 0.92]	0.86	[0.19; 3.82]
Morze 2021	Mediterranean diet	Intake	Intake	Breast cancer	0.41	[0.19; 0.87]	0.97	[0.94; 1.00]	0.42	[0.20; 0.91]
Sayehmiri 2018	Selenium	Supplementation	Supplementation+Intake	Prostate cancer	0.90	[0.74; 1.09]	0.89	[0.64; 1.23]	1.01	[0.69; 1.48]
Stratton 2011	Multivitamin	Supplementation	Supplementation	Prostate cancer	0.98	[0.81; 1.20]	1.05	[0.94; 1.16]	0.93	[0.75; 1.17]
Stratton 2011	Vitamin E	Supplementation	Supplementation	Prostate cancer	1.01	[0.90; 1.13]	1.09	[0.94; 1.27]	0.93	[0.77; 1.12]
Stratton 2011	Vitamin C	Supplementation	Supplementation	Prostate cancer	1.10	[0.93; 1.29]	0.96	[0.86; 1.06]	1.15	[0.94; 1.39]
Stratton 2011	β-Carotene	Supplementation	Supplementation	Prostate cancer	1.19	[0.87; 1.65]	0.70	[0.63; 0.78]	1.70	[1.21; 2.38]
Vinceti 2018a	Selenium	Supplementation	Status	Cancer	0.99	[0.86; 1.14]	0.75	[0.59; 0.94]	1.32	[1.01; 1.73]
Vinceti 2018a	Selenium	Supplementation	Intake	Mortality	0.81	[0.49; 1.32]	0.93	[0.83; 1.04]	0.87	[0.52; 1.45]
Wien 2012	Folic acid	Supplementation	Supplementation	Cancer	1.07	[1.00; 1.14]	1.03	[0.90; 1.18]	1.04	[0.89; 1.21]
Random effects model									1.09	[1.01; 1.18]
Prediction interval										[0.88; 1.36]
Heterogeneity: $I^2 = 23\%$, $\tau^2 = 0.0092$, $p = 0.16$										
**Eye disease**										
Jiang 2019	Vitamin E	Supplementation	Intake	Age-related cataract	0.97	[0.91; 1.03]	0.90	[0.80; 1.00]	1.08	[0.95; 1.22]
Jiang 2019	β-Carotene	Supplementation	Intake	Age-related cataract	0.99	[0.92; 1.07]	0.90	[0.83; 0.99]	1.10	[0.98; 1.24]
Zhao 2014	Multivitamin/Minerals	Supplementation	Supplementation	Nuclear cataract	0.73	[0.59; 0.90]	0.73	[0.64; 0.82]	1.00	[0.78; 1.28]
Zhao 2014	Multivitamin/Minerals	Supplementation	Supplementation	Cortical cataract	0.92	[0.69; 1.23]	0.81	[0.68; 0.94]	1.14	[0.82; 1.58]
Zhao 2014	Multivitamin/Minerals	Supplementation	Supplementation	Posterior subcapsular cataract	1.81	[1.30; 2.54]	0.96	[0.72; 1.20]	1.89	[1.24; 2.87]
Random effects model									1.15	[0.96; 1.37]
Prediction interval										[0.63; 2.09]
Heterogeneity: $I^2 = 43\%$, $\tau^2 = 0.0270$, $p = 0.14$										
Test for subgroup differences: $\chi^2_6 = 5.73$, df = 6 ($p = 0.45$)										

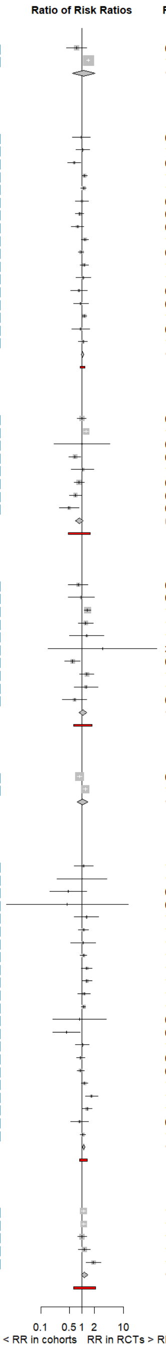

Supplementary Fig. S4 Forest plot, subgroup analysis of BoE-pairs with binary outcomes by type of outcome

Reference	Intervention/Exposure in SR	Type of intake (RCTs)	Type of exposure (cohorts)	Outcome in SR	RR (RCTs)	95%-CI	RR (cohorts)	95%-CI	RRR	95%-CI
**More or less identical**										
Feng 2015	Folic acid	Supplementation	Supplementation	Congenital heart defects	0.58	[0.33; 1.02]	0.60	[0.38; 0.96]	0.97	[0.47; 2.01]
Jonker 2020	Folic acid	Supplementation	Supplementation	Low birthweight	0.96	[0.88; 1.04]	0.71	[0.60; 0.83]	1.35	[1.13; 1.62]
Kim 2018	Multivitamin/Minerals	Supplementation	Supplementation	Coronary heart disease	0.97	[0.79; 1.19]	0.86	[0.76; 0.97]	1.13	[0.89; 1.43]
Lin 2020	Vitamin D	Supplementation	Supplementation	Nephrolithiasis	0.84	[0.65; 1.10]	1.24	[1.01; 1.51]	0.68	[0.49; 0.94]
Lin 2020	Calcium	Supplementation	Supplementation	Nephrolithiasis	1.20	[0.65; 2.21]	1.16	[1.00; 1.35]	1.03	[0.55; 1.94]
Stratton 2011	Multivitamin	Supplementation	Supplementation	Prostate cancer	0.98	[0.81; 1.20]	1.05	[0.94; 1.16]	0.93	[0.75; 1.17]
Stratton 2011	Vitamin E	Supplementation	Supplementation	Prostate cancer	1.01	[0.90; 1.13]	1.09	[0.94; 1.27]	0.93	[0.77; 1.12]
Wolf 2017	Multivitamin	Supplementation	Supplementation	Preterm birth	1.09	[0.43; 2.77]	0.84	[0.69; 1.03]	1.30	[0.50; 3.36]
**Random effects model**									1.01	[0.87; 1.18]
**Prediction interval**										[0.67; 1.52]
Heterogeneity: $I^2 = 60\%$, $\tau^2 = 0.0216$, $p = 0.01$										
**Similar but not identical**										
Aburto 2013	Low-sodium	Intake	Intake	All-cause mortality	0.70	[0.44; 1.14]	0.95	[0.71; 1.27]	0.74	[0.42; 1.29]
Aburto 2013	Low-sodium	Intake	Intake	Cardiovascular disease	0.84	[0.57; 1.23]	0.87	[0.64; 1.18]	0.97	[0.59; 1.58]
Afshin 2014	Nuts	Intake	Intake	Coronary heart disease	0.79	[0.55; 1.14]	0.76	[0.69; 0.83]	1.04	[0.71; 1.51]
Afshin 2014	Nuts	Intake	Intake	Stroke	0.62	[0.44; 0.87]	0.95	[0.83; 1.09]	0.65	[0.45; 0.94]
Afshin 2014	Nuts	Intake	Intake	Type 2 diabetes	0.86	[0.68; 1.08]	0.87	[0.80; 0.95]	0.99	[0.77; 1.27]
Alexander 2017	Omega-3 fatty acids	Intake+Supplementation	Intake	Cardiovascular disease	0.94	[0.85; 1.05]	0.82	[0.74; 0.92]	1.15	[0.98; 1.33]
Bolland 2015	Calcium	Supplementation	Supplementation	Fractures	0.89	[0.81; 0.96]	1.02	[0.93; 1.11]	0.87	[0.77; 0.99]
Chowdhury 2012	Omega-3 fatty acids	Supplementation	Intake	Stroke	0.98	[0.89; 1.08]	0.90	[0.80; 1.01]	1.09	[0.94; 1.27]
Chowdhury 2014a	α-Linolenic acid	Supplementation	Intake	Coronary heart disease	0.97	[0.69; 1.36]	0.99	[0.86; 1.14]	0.98	[0.68; 1.41]
Chowdhury 2014a	Omega-6 fatty acids	Intake+Supplementation	Intake	Coronary heart disease	0.86	[0.69; 1.07]	0.98	[0.90; 1.06]	0.88	[0.69; 1.11]
Filippini 2020	Green tea	Supplementation	Intake	Endometrial cancer	0.33	[0.01; 8.15]	0.75	[0.43; 1.30]	0.44	[0.01; 13.14]
Fu 2021	Vitamin C	Supplementation	Supplementation	Lung cancer	1.30	[0.68; 2.48]	1.02	[0.85; 1.23]	1.27	[0.65; 2.50]
Fu 2021	Vitamin C	Supplementation	Supplementation	Breast cancer	1.11	[0.87; 1.41]	1.02	[0.88; 1.18]	1.09	[0.82; 1.44]
Fu 2021	Vitamin C	Supplementation	Supplementation	Colorectal cancer	0.84	[0.64; 1.10]	0.80	[0.42; 1.52]	1.05	[0.52; 2.11]
Grosso 2015	Mediterranean diet	Intake	Intake	Cardiovascular disease	0.55	[0.39; 0.76]	0.71	[0.65; 0.78]	0.77	[0.55; 1.09]
Jiang 2019	Vitamin E	Supplementation	Intake	Age-related cataract	0.97	[0.91; 1.03]	0.90	[0.80; 1.00]	1.08	[0.95; 1.22]
Jiang 2019	β-Carotene	Supplementation	Intake	Age-related cataract	0.99	[0.92; 1.07]	0.90	[0.83; 0.99]	1.10	[0.98; 1.24]
Johnston 2019	Low red meat	Intake	Intake	Mortality	1.00	[0.84; 1.19]	0.86	[0.79; 0.94]	1.16	[0.96; 1.41]
Johnston 2019	Low red meat	Intake	Intake	Type 2 diabetes	0.95	[0.89; 1.02]	0.76	[0.68; 0.86]	1.25	[1.09; 1.43]
Johnston 2019	Low red meat	Intake	Intake	Colorectal cancer	1.02	[0.89; 1.18]	0.94	[0.85; 1.05]	1.09	[0.91; 1.29]
Jonker 2020	Folic acid	Supplementation	Supplementation+Intake	Small for gestational age	0.98	[0.92; 1.06]	0.80	[0.50; 1.11]	1.22	[0.82; 1.84]
Kastorini 2011	Mediterranean diet	Intake	Intake	Metabolic syndrome	0.45	[0.24; 0.84]	0.46	[0.11; 1.88]	0.98	[0.21; 4.62]
Kim 2018	Multivitamin/Minerals	Supplementation	Supplementation	Mortality	0.95	[0.83; 1.09]	1.00	[0.96; 1.05]	0.95	[0.82; 1.10]
Kim 2018	Multivitamin/Minerals	Supplementation	Supplementation	Stroke	1.05	[0.70; 1.56]	0.98	[0.91; 1.04]	1.07	[0.71; 1.61]
Kong 2014	Vitamins	Supplementation	Intake	Gastric cancer	1.11	[0.94; 1.31]	0.85	[0.66; 1.08]	1.31	[0.97; 1.76]
Lin 2019	Vitamin E	Supplementation	Intake	Bladder cancer	1.10	[0.86; 1.35]	0.84	[0.72; 0.96]	1.31	[1.00; 1.71]
Martinez-Gonzalez 2014b	Olive oil	Supplementation+Intake	Intake	Coronary heart disease	0.81	[0.53; 1.24]	0.96	[0.78; 1.18]	0.84	[0.53; 1.35]
Martinez-Gonzalez 2014b	Olive oil	Supplementation+Intake	Intake	Stroke	0.69	[0.48; 0.98]	0.74	[0.60; 0.92]	0.93	[0.62; 1.41]
Moazzen 2018	Folic acid	Supplementation	Supplementation	Colorectal cancer	1.07	[0.86; 1.43]	0.96	[0.76; 1.21]	1.11	[0.79; 1.57]
Mocellin 2017	Vitamin B6	Supplementation	Intake	Cancer	1.03	[0.94; 1.13]	0.95	[0.90; 1.01]	1.08	[0.97; 1.21]
Morze 2021	Mediterranean diet	Intake	Intake	Mortality	0.75	[0.17; 3.33]	0.87	[0.82; 0.92]	0.86	[0.19; 3.82]
Morze 2021	Mediterranean diet	Intake	Intake	Breast cancer	0.41	[0.19; 0.87]	0.97	[0.94; 1.00]	0.42	[0.20; 0.91]
Sayehmiri 2018	Selenium	Supplementation	Supplementation+Intake	Prostate cancer	0.90	[0.74; 1.09]	0.89	[0.64; 1.23]	1.01	[0.69; 1.48]
Schwingshackl 2015	Mediterranean diet	Intake	Intake	Type 2 diabetes	0.70	[0.54; 0.91]	0.83	[0.74; 0.92]	0.84	[0.64; 1.12]
Schwingshackl 2017	Olive oil	Intake	Intake	Type 2 diabetes	0.60	[0.43; 0.84]	0.87	[0.80; 0.94]	0.69	[0.49; 0.97]
Stratton 2011	Vitamin C	Supplementation	Supplementation	Prostate cancer	1.10	[0.93; 1.29]	0.96	[0.86; 1.06]	1.15	[0.94; 1.39]
Stratton 2011	β-Carotene	Supplementation	Supplementation	Prostate cancer	1.19	[0.87; 1.65]	0.70	[0.63; 0.78]	1.70	[1.21; 2.38]
Trikalinos 2012	Vitamin E	Supplementation	Supplementation+Intake	Mortality	0.97	[0.91; 1.03]	0.85	[0.78; 0.93]	1.14	[1.02; 1.27]
Vinceti 2018a	Selenium	Supplementation	Intake	Mortality	0.81	[0.49; 1.32]	0.93	[0.83; 1.04]	0.87	[0.52; 1.45]
Vinceti 2018b	Selenium	Supplementation	Intake	Type 2 diabetes	1.11	[1.01; 1.22]	2.28	[1.31; 3.89]	0.49	[0.28; 0.85]
Wien 2012	Folic acid	Supplementation	Supplementation	Cancer	1.07	[1.00; 1.14]	1.03	[0.90; 1.18]	1.04	[0.89; 1.21]
Wolf 2017	Multivitamin	Supplementation	Supplementation	Stillbirth	2.43	[0.12; 50.05]	0.78	[0.59; 1.03]	3.12	[0.15; 64.44]
Yang 2016	Folic acid	Supplementation	Supplementation+Intake	Gestational hypertension	0.62	[0.04; 0.94]	1.05	[1.00; 1.11]	0.59	[0.38; 0.91]
Yang 2020	Calcium	Supplementation	Intake	Cardiovascular disease	0.91	[0.56; 1.46]	0.98	[0.90; 1.06]	0.93	[0.57; 1.51]
Yu 2021	Folic acid	Supplementation	Supplementation+Intake	Pre-eclampsia	1.11	[0.75; 1.62]	0.85	[0.76; 0.94]	1.31	[0.88; 1.95]
Zhao 2014	Multivitamin/Minerals	Supplementation	Supplementation	Nuclear cataract	0.73	[0.59; 0.90]	0.73	[0.64; 0.82]	1.00	[0.78; 1.28]
Zhao 2014	Multivitamin/Minerals	Supplementation	Supplementation	Cortical cataract	0.92	[0.69; 1.23]	0.81	[0.68; 0.94]	1.14	[0.82; 1.58]
Zhao 2014	Multivitamin/Minerals	Supplementation	Supplementation	Posterior subcapsular cataract	1.81	[1.30; 2.54]	0.96	[0.72; 1.20]	1.89	[1.24; 2.87]
**Random effects model**									1.03	[0.97; 1.10]
**Prediction interval**										[0.76; 1.40]
Heterogeneity: $I^2 = 48\%$, $\tau^2 = 0.0225$, $p < 0.01$										
**Broadly similar**										
Aguilar-Cordero 2020	Vitamin D	Supplementation	Status	Pre-eclampsia	0.66	[0.42; 1.05]	0.82	[0.60; 1.11]	0.80	[0.46; 1.40]
Chowdhury 2014b	Vitamin D	Supplementation	Status	All-cause mortality	0.98	[0.94; 1.03]	0.69	[0.65; 0.75]	1.42	[1.30; 1.55]
Chung 2011	Vitamin D	Supplementation	Status	Colorectal cancer	1.02	[0.60; 1.74]	0.94	[0.91; 0.97]	1.09	[0.64; 1.85]
Chung 2011	Vitamin D	Supplementation	Status	Breast cancer	0.99	[0.25; 3.99]	0.99	[0.97; 1.01]	1.00	[0.25; 4.00]
Filippini 2020	Green tea	Supplementation	Intake	Prostate cancer	0.50	[0.18; 1.36]	1.09	[0.89; 1.32]	0.46	[0.16; 1.29]
Vinceti 2018a	Selenium	Supplementation	Status	Cancer	0.99	[0.86; 1.14]	0.75	[0.59; 0.94]	1.32	[1.01; 1.73]
Yao 2019	Vitamin D	Supplementation	Status	Fractures	1.06	[0.97; 1.15]	0.89	[0.83; 0.96]	1.19	[1.06; 1.33]
Zhang 2016	Selenium	Supplementation	Status	Cardiovascular disease	0.91	[0.74; 1.10]	0.87	[0.76; 0.99]	1.05	[0.82; 1.33]
Zhang 2018	Vitamin D	Supplementation	Status	Gestational diabetes mellitus	0.72	[0.39; 1.31]	0.58	[0.44; 0.76]	1.24	[0.64; 2.40]
Zhou 2017	Vitamin D	Supplementation	Status	Preterm birth	0.58	[0.30; 1.11]	0.88	[0.73; 1.05]	0.66	[0.33; 1.30]
**Random effects model**									1.15	[0.99; 1.34]
**Prediction interval**										[0.78; 1.69]
Heterogeneity: $I^2 = 57\%$, $\tau^2 = 0.0220$, $p = 0.01$										
Test for subgroup differences: $\chi^2_2 = 1.92$, df = 2 ($p = 0.38$)										

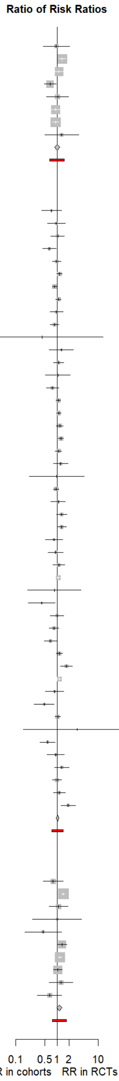

Supplementary Fig. S5 Forest plot, subgroup analysis of BoE-pairs with binary outcomes by overall PI/ECO similarity degree

Reference	Intervention/Exposure in SR	Type of intake (RCTs)	Type of exposure (cohorts)	Outcome in SR	RR (RCTs)	95%-CI	RR (cohorts)	95%-CI	RRR	95%-CI
**More or less identical**										
Aguilar-Cordero 2020	Vitamin D	Supplementation	Status	Pre-eclampsia	0.66	[0.42; 1.05]	0.82	[0.60; 1.11]	0.80	[0.46; 1.40]
Bolland 2015	Calcium	Supplementation	Supplementation	Fractures	0.89	[0.81; 0.96]	1.02	[0.93; 1.11]	0.87	[0.77; 0.99]
Feng 2015	Folic acid	Supplementation	Supplementation	Congenital heart defects	0.58	[0.33; 1.02]	0.60	[0.38; 0.96]	0.97	[0.47; 2.01]
Jiang 2019	Vitamin E	Supplementation	Intake	Age-related cataract	0.97	[0.91; 1.03]	0.90	[0.80; 1.00]	1.08	[0.95; 1.22]
Jiang 2019	β-Carotene	Supplementation	Intake	Age-related cataract	0.99	[0.92; 1.07]	0.90	[0.83; 0.99]	1.10	[0.98; 1.24]
Jonker 2020	Folic acid	Supplementation	Supplementation	Low birthweight	0.96	[0.88; 1.04]	0.71	[0.60; 0.83]	1.35	[1.13; 1.62]
Jonker 2020	Folic acid	Supplementation	Supplementation+Intake	Small for gestational age	0.98	[0.92; 1.06]	0.80	[0.50; 1.11]	1.22	[0.82; 1.84]
Kim 2018	Multivitamin/Minerals	Supplementation	Supplementation	Coronary heart disease	0.97	[0.79; 1.19]	0.86	[0.76; 0.97]	1.13	[0.89; 1.43]
Lin 2019	Vitamin E	Supplementation	Intake	Bladder cancer	1.10	[0.86; 1.35]	0.84	[0.72; 0.96]	1.31	[1.00; 1.71]
Lin 2020	Vitamin D	Supplementation	Supplementation	Nephrolithiasis	0.84	[0.65; 1.10]	1.24	[1.01; 1.51]	0.68	[0.49; 0.94]
Lin 2020	Calcium	Supplementation	Supplementation	Nephrolithiasis	1.20	[0.65; 2.21]	1.16	[1.00; 1.35]	1.03	[0.55; 1.94]
Stratton 2011	Multivitamin	Supplementation	Supplementation	Prostate cancer	0.98	[0.81; 1.20]	1.05	[0.94; 1.16]	0.93	[0.75; 1.17]
Stratton 2011	Vitamin E	Supplementation	Supplementation	Prostate cancer	1.01	[0.90; 1.13]	1.09	[0.94; 1.27]	0.93	[0.77; 1.12]
Wolf 2017	Multivitamin	Supplementation	Supplementation	Preterm birth	1.09	[0.43; 2.77]	0.84	[0.69; 1.03]	1.30	[0.50; 3.36]
Yang 2020	Calcium	Supplementation	Intake	Cardiovascular disease	0.91	[0.56; 1.46]	0.98	[0.90; 1.06]	0.93	[0.57; 1.51]
**Random effects model**									1.03	[0.94; 1.13]
**Prediction interval**										[0.79; 1.35]
Heterogeneity: $I^2 = 55\%$, $\tau^2 = 0.0135$, $p < 0.01$										
**Similar but not identical**										
Aburto 2013	Low-sodium	Intake	Intake	All-cause mortality	0.70	[0.44; 1.14]	0.95	[0.71; 1.27]	0.74	[0.42; 1.29]
Aburto 2013	Low-sodium	Intake	Intake	Cardiovascular disease	0.84	[0.57; 1.23]	0.87	[0.64; 1.18]	0.97	[0.59; 1.58]
Afshin 2014	Nuts	Intake	Intake	Coronary heart disease	0.79	[0.55; 1.14]	0.76	[0.69; 0.83]	1.04	[0.71; 1.51]
Afshin 2014	Nuts	Intake	Intake	Stroke	0.62	[0.44; 0.87]	0.95	[0.83; 1.09]	0.65	[0.45; 0.94]
Afshin 2014	Nuts	Intake	Intake	Type 2 diabetes	0.86	[0.68; 1.08]	0.87	[0.80; 0.95]	0.99	[0.77; 1.27]
Alexander 2017	Omega-3 fatty acids	Intake+Supplementation	Intake	Cardiovascular disease	0.94	[0.85; 1.05]	0.82	[0.74; 0.92]	1.15	[0.98; 1.33]
Chowdhury 2012	Omega-3 fatty acids	Supplementation	Intake	Stroke	0.98	[0.89; 1.08]	0.90	[0.80; 1.01]	1.09	[0.94; 1.27]
Chowdhury 2014a	α-Linolenic acid	Supplementation	Intake	Coronary heart disease	0.97	[0.69; 1.36]	0.99	[0.86; 1.14]	0.98	[0.68; 1.41]
Chowdhury 2014a	Omega-6 fatty acids	Intake+Supplementation	Intake	Coronary heart disease	0.86	[0.69; 1.07]	0.98	[0.90; 1.06]	0.88	[0.69; 1.11]
Chowdhury 2014b	Vitamin D	Supplementation	Status	All-cause mortality	0.98	[0.94; 1.03]	0.69	[0.65; 0.75]	1.42	[1.30; 1.55]
Chung 2011	Vitamin D	Supplementation	Status	Colorectal cancer	1.02	[0.60; 1.74]	0.94	[0.91; 0.97]	1.09	[0.64; 1.85]
Chung 2011	Vitamin D	Supplementation	Status	Breast cancer	0.99	[0.25; 3.99]	0.99	[0.97; 1.01]	1.00	[0.25; 4.00]
Filippini 2020	Green tea	Supplementation	Intake	Endometrial cancer	0.33	[0.01; 8.15]	0.75	[0.43; 1.30]	0.44	[0.01; 13.14]
Fu 2021	Vitamin C	Supplementation	Supplementation	Lung cancer	1.30	[0.68; 2.48]	1.02	[0.85; 1.23]	1.27	[0.65; 2.50]
Fu 2021	Vitamin C	Supplementation	Supplementation	Breast cancer	1.11	[0.87; 1.41]	1.02	[0.88; 1.18]	1.09	[0.82; 1.44]
Fu 2021	Vitamin C	Supplementation	Supplementation	Colorectal cancer	0.84	[0.64; 1.10]	0.80	[0.42; 1.52]	1.05	[0.52; 2.11]
Grosso 2015	Mediterranean diet	Intake	Intake	Cardiovascular disease	0.55	[0.39; 0.76]	0.71	[0.65; 0.78]	0.77	[0.55; 1.09]
Johnston 2019	Low red meat	Intake	Intake	Mortality	1.00	[0.84; 1.19]	0.86	[0.79; 0.94]	1.16	[0.96; 1.41]
Johnston 2019	Low red meat	Intake	Intake	Type 2 diabetes	0.95	[0.89; 1.02]	0.76	[0.68; 0.86]	1.25	[1.09; 1.43]
Johnston 2019	Low red meat	Intake	Intake	Colorectal cancer	1.02	[0.89; 1.18]	0.94	[0.85; 1.05]	1.09	[0.91; 1.29]
Kastorini 2011	Mediterranean diet	Intake	Intake	Metabolic syndrome	0.45	[0.24; 0.84]	0.46	[0.11; 1.88]	0.98	[0.21; 4.62]
Kim 2018	Multivitamin/Minerals	Supplementation	Supplementation	Mortality	0.95	[0.83; 1.09]	1.00	[0.96; 1.05]	0.95	[0.82; 1.10]
Kim 2018	Multivitamin/Minerals	Supplementation	Supplementation	Stroke	1.05	[0.70; 1.56]	0.98	[0.91; 1.04]	1.07	[0.71; 1.61]
Kong 2014	Vitamins	Supplementation	Intake	Gastric cancer	1.11	[0.94; 1.31]	0.85	[0.66; 1.08]	1.31	[0.97; 1.76]
Martinez-Gonzalez 2014b	Olive oil	Supplementation+Intake	Intake	Coronary heart disease	0.81	[0.53; 1.24]	0.96	[0.78; 1.18]	0.84	[0.53; 1.35]
Martinez-Gonzalez 2014b	Olive oil	Supplementation+Intake	Intake	Stroke	0.69	[0.48; 0.98]	0.74	[0.60; 0.92]	0.93	[0.62; 1.41]
Moazzen 2018	Folic acid	Supplementation	Supplementation	Colorectal cancer	1.07	[0.86; 1.43]	0.96	[0.76; 1.21]	1.11	[0.79; 1.57]
Mocellin 2017	Vitamin B6	Supplementation	Intake	Cancer	1.03	[0.94; 1.13]	0.95	[0.90; 1.01]	1.08	[0.97; 1.21]
Morze 2021	Mediterranean diet	Intake	Intake	Mortality	0.75	[0.17; 3.33]	0.87	[0.82; 0.92]	0.86	[0.19; 3.82]
Morze 2021	Mediterranean diet	Intake	Intake	Breast cancer	0.41	[0.19; 0.87]	0.97	[0.94; 1.00]	0.42	[0.20; 0.91]
Sayehmiri 2018	Selenium	Supplementation	Supplementation+Intake	Prostate cancer	0.90	[0.74; 1.09]	0.89	[0.64; 1.23]	1.01	[0.69; 1.48]
Schwingshackl 2015	Mediterranean diet	Intake	Intake	Type 2 diabetes	0.70	[0.54; 0.91]	0.83	[0.74; 0.92]	0.84	[0.64; 1.12]
Schwingshackl 2017	Olive oil	Intake	Intake	Type 2 diabetes	0.60	[0.43; 0.84]	0.87	[0.80; 0.94]	0.69	[0.49; 0.97]
Stratton 2011	Vitamin C	Supplementation	Supplementation	Prostate cancer	1.10	[0.93; 1.29]	0.96	[0.86; 1.06]	1.15	[0.94; 1.39]
Stratton 2011	β-Carotene	Supplementation	Supplementation	Prostate cancer	1.19	[0.87; 1.65]	0.70	[0.63; 0.78]	1.70	[1.21; 2.38]
Trikalinos 2012	Vitamin E	Supplementation	Supplementation+Intake	Mortality	0.97	[0.91; 1.03]	0.85	[0.78; 0.93]	1.14	[1.02; 1.27]
Vinceti 2018a	Selenium	Supplementation	Status	Cancer	0.99	[0.86; 1.14]	0.75	[0.59; 0.94]	1.32	[1.01; 1.73]
Vinceti 2018a	Selenium	Supplementation	Intake	Mortality	0.81	[0.49; 1.32]	0.93	[0.83; 1.04]	0.87	[0.52; 1.45]
Vinceti 2018b	Selenium	Supplementation	Intake	Type 2 diabetes	1.11	[1.01; 1.22]	2.28	[1.31; 3.89]	0.49	[0.28; 0.85]
Wien 2012	Folic acid	Supplementation	Supplementation	Cancer	1.07	[1.00; 1.14]	1.03	[0.90; 1.18]	1.04	[0.89; 1.21]
Wolf 2017	Multivitamin	Supplementation	Supplementation	Stillbirth	2.43	[0.12; 50.05]	0.78	[0.59; 1.03]	3.12	[0.15; 64.44]
Yang 2016	Folic acid	Supplementation	Supplementation+Intake	Gestational hypertension	0.62	[0.04; 0.94]	1.05	[1.00; 1.11]	0.59	[0.38; 0.91]
Yao 2019	Vitamin D	Supplementation	Status	Fractures	1.06	[0.97; 1.15]	0.89	[0.83; 0.96]	1.19	[1.06; 1.33]
Yu 2021	Folic acid	Supplementation	Supplementation+Intake	Pre-eclampsia	1.11	[0.75; 1.62]	0.85	[0.76; 0.94]	1.31	[0.88; 1.95]
Zhang 2016	Selenium	Supplementation	Status	Cardiovascular disease	0.91	[0.74; 1.10]	0.87	[0.76; 0.99]	1.05	[0.82; 1.33]
Zhang 2018	Vitamin D	Supplementation	Status	Gestational diabetes mellitus	0.72	[0.39; 1.31]	0.58	[0.44; 0.76]	1.24	[0.64; 2.40]
Zhao 2014	Multivitamin/Minerals	Supplementation	Supplementation	Nuclear cataract	0.73	[0.59; 0.90]	0.73	[0.64; 0.82]	1.00	[0.78; 1.28]
Zhao 2014	Multivitamin/Minerals	Supplementation	Supplementation	Cortical cataract	0.92	[0.69; 1.23]	0.81	[0.68; 0.94]	1.14	[0.82; 1.58]
Zhao 2014	Multivitamin/Minerals	Supplementation	Supplementation	Posterior subcapsular cataract	1.81	[1.30; 2.54]	0.96	[0.72; 1.20]	1.89	[1.24; 2.87]
Zhou 2017	Vitamin D	Supplementation	Status	Preterm birth	0.58	[0.30; 1.11]	0.88	[0.73; 1.05]	0.66	[0.33; 1.30]
**Random effects model**									1.05	[0.98; 1.12]
**Prediction interval**										[0.76; 1.46]
Heterogeneity: $I^2 = 59\%$, $\tau^2 = 0.0255$, $p < 0.01$										
**Broadly similar**										
Filippini 2020	Green tea	Supplementation	Intake	Prostate cancer	0.50	[0.18; 1.36]	1.09	[0.89; 1.32]	0.46	[0.16; 1.29]
**Prediction interval**										
Test for subgroup differences: $\chi^2_2 = 2.51$, df = 2 ($p = 0.28$)										

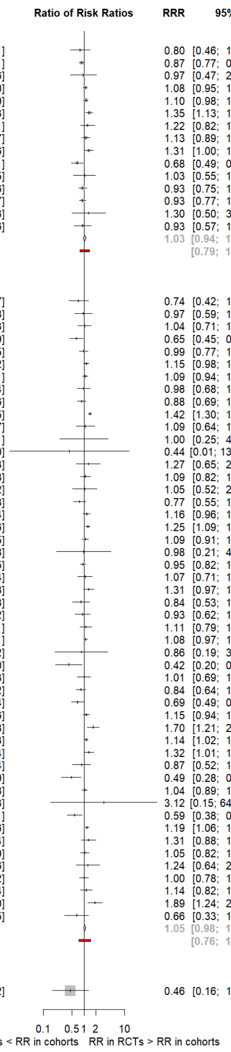

Supplementary Fig. S6 Forest plot, subgroup analysis of BoE-pairs with binary outcomes by population similarity degree

Reference	Intervention/Exposure in SR	Type of intake (RCTs)	Type of exposure (cohorts)	Outcome in SR	RR (RCTs)	95%-CI	RR (cohorts)	95%-CI	Ratio of Risk Ratios	RRR	95%-CI
**More or less identical**											
Aburto 2013	Low-sodium	Intake	Intake	All-cause mortality	0.70	[0.44; 1.14]	0.95	[0.71; 1.27]		0.74	[0.42; 1.29]
Aburto 2013	Low-sodium	Intake	Intake	Cardiovascular disease	0.84	[0.57; 1.23]	0.87	[0.64; 1.18]		0.97	[0.59; 1.58]
Afshin 2014	Nuts	Intake	Intake	Coronary heart disease	0.79	[0.55; 1.14]	0.76	[0.69; 0.83]		1.04	[0.71; 1.51]
Afshin 2014	Nuts	Intake	Intake	Stroke	0.62	[0.44; 0.87]	0.95	[0.83; 1.09]		0.65	[0.45; 0.94]
Afshin 2014	Nuts	Intake	Intake	Type 2 diabetes	0.86	[0.68; 1.08]	0.87	[0.80; 0.95]		0.99	[0.77; 1.27]
Feng 2015	Folic acid	Supplementation	Supplementation	Congenital heart defects	0.58	[0.33; 1.02]	0.60	[0.38; 0.96]		0.97	[0.47; 2.01]
Fu 2021	Vitamin C	Supplementation	Supplementation	Lung cancer	1.30	[0.68; 2.48]	1.02	[0.85; 1.23]		1.27	[0.65; 2.50]
Fu 2021	Vitamin C	Supplementation	Supplementation	Breast cancer	1.11	[0.87; 1.41]	1.02	[0.88; 1.18]		1.09	[0.82; 1.44]
Fu 2021	Vitamin C	Supplementation	Supplementation	Colorectal cancer	0.84	[0.64; 1.10]	0.80	[0.42; 1.52]		1.05	[0.52; 2.11]
Grosso 2015	Mediterranean diet	Intake	Intake	Cardiovascular disease	0.55	[0.39; 0.76]	0.71	[0.65; 0.78]		0.77	[0.55; 1.09]
Jonker 2020	Folic acid	Supplementation	Supplementation	Low birthweight	0.96	[0.88; 1.04]	0.71	[0.60; 0.83]		1.35	[1.13; 1.62]
Kastorini 2011	Mediterranean diet	Intake	Intake	Metabolic syndrome	0.45	[0.24; 0.84]	0.46	[0.11; 1.88]		0.98	[0.21; 4.62]
Kim 2018	Multivitamin/Minerals	Supplementation	Supplementation	Mortality	0.95	[0.83; 1.09]	1.00	[0.96; 1.05]		0.95	[0.82; 1.10]
Kim 2018	Multivitamin/Minerals	Supplementation	Supplementation	Coronary heart disease	0.97	[0.79; 1.19]	0.86	[0.76; 0.97]		1.13	[0.89; 1.43]
Kim 2018	Multivitamin/Minerals	Supplementation	Supplementation	Stroke	1.05	[0.70; 1.56]	0.98	[0.91; 1.04]		1.07	[0.71; 1.61]
Lin 2020	Vitamin D	Supplementation	Supplementation	Nephrolithiasis	0.84	[0.65; 1.10]	1.24	[1.01; 1.51]		0.68	[0.49; 0.94]
Lin 2020	Calcium	Supplementation	Supplementation	Nephrolithiasis	1.20	[0.65; 2.21]	1.16	[1.00; 1.35]		1.03	[0.55; 1.94]
Moazzen 2018	Folic acid	Supplementation	Supplementation	Colorectal cancer	1.07	[0.86; 1.43]	0.96	[0.76; 1.21]		1.11	[0.79; 1.57]
Morze 2021	Mediterranean diet	Intake	Intake	Mortality	0.75	[0.17; 3.33]	0.87	[0.82; 0.92]		0.86	[0.19; 3.82]
Morze 2021	Mediterranean diet	Intake	Intake	Breast cancer	0.41	[0.19; 0.87]	0.97	[0.94; 1.00]		0.42	[0.20; 0.91]
Schwingshackl 2015	Mediterranean diet	Intake	Intake	Type 2 diabetes	0.70	[0.54; 0.91]	0.83	[0.74; 0.92]		0.84	[0.64; 1.12]
Schwingshackl 2017	Olive oil	Intake	Intake	Type 2 diabetes	0.60	[0.43; 0.84]	0.87	[0.80; 0.94]		0.69	[0.49; 0.97]
Stratton 2011	Multivitamin	Supplementation	Supplementation	Prostate cancer	0.98	[0.81; 1.20]	1.05	[0.94; 1.16]		0.93	[0.75; 1.17]
Stratton 2011	Vitamin E	Supplementation	Supplementation	Prostate cancer	1.01	[0.90; 1.13]	1.09	[0.94; 1.27]		0.93	[0.77; 1.12]
Stratton 2011	Vitamin C	Supplementation	Supplementation	Prostate cancer	1.10	[0.93; 1.29]	0.96	[0.86; 1.06]		1.15	[0.94; 1.39]
Stratton 2011	β-Carotene	Supplementation	Supplementation	Prostate cancer	1.19	[0.87; 1.65]	0.70	[0.63; 0.78]		1.70	[1.21; 2.38]
Wolf 2017	Multivitamin	Supplementation	Supplementation	Preterm birth	1.09	[0.43; 2.77]	0.84	[0.69; 1.03]		1.30	[0.50; 3.36]
Wolf 2017	Multivitamin	Supplementation	Supplementation	Stillbirth	2.43	[0.12; 50.05]	0.78	[0.59; 1.03]		3.12	[0.15; 64.44]
Zhao 2014	Multivitamin/Minerals	Supplementation	Supplementation	Nuclear cataract	0.73	[0.59; 0.90]	0.73	[0.64; 0.82]		1.00	[0.78; 1.28]
Zhao 2014	Multivitamin/Minerals	Supplementation	Supplementation	Cortical cataract	0.92	[0.69; 1.23]	0.81	[0.68; 0.94]		1.14	[0.82; 1.58]
Zhao 2014	Multivitamin/Minerals	Supplementation	Supplementation	Posterior subcapsular cataract	1.81	[1.30; 2.54]	0.96	[0.72; 1.20]		1.89	[1.24; 2.87]
**Random effects model**										1.00	[0.92; 1.10]
**Prediction interval**											[0.70; 1.44]
Heterogeneity: $I^2 = 50\%$, $\tau^2 = 0.0293$, $p < 0.01$											
**Similar but not identical**											
Alexander 2017	Omega-3 fatty acids	Intake+Supplementation	Intake	Cardiovascular disease	0.94	[0.85; 1.05]	0.82	[0.74; 0.92]		1.15	[0.98; 1.33]
Bolland 2015	Calcium	Supplementation	Supplementation	Fractures	0.89	[0.81; 0.96]	1.02	[0.93; 1.11]		0.87	[0.77; 0.99]
Chowdhury 2012	Omega-3 fatty acids	Supplementation	Intake	Stroke	0.98	[0.89; 1.08]	0.90	[0.80; 1.01]		1.09	[0.94; 1.27]
Chowdhury 2014a	α-Linolenic acid	Supplementation	Intake	Coronary heart disease	0.97	[0.69; 1.36]	0.99	[0.86; 1.14]		0.98	[0.68; 1.41]
Chowdhury 2014a	Omega-6 fatty acids	Intake+Supplementation	Intake	Coronary heart disease	0.86	[0.69; 1.07]	0.98	[0.90; 1.06]		0.88	[0.69; 1.11]
Filippini 2020	Green tea	Supplementation	Intake	Prostate cancer	0.50	[0.18; 1.36]	1.09	[0.89; 1.32]		0.46	[0.16; 1.29]
Filippini 2020	Green tea	Supplementation	Intake	Endometrial cancer	0.33	[0.01; 8.15]	0.75	[0.43; 1.30]		0.44	[0.01; 13.14]
Jiang 2019	Vitamin E	Supplementation	Intake	Age-related cataract	0.97	[0.91; 1.03]	0.90	[0.80; 1.00]		1.08	[0.95; 1.22]
Jiang 2019	β-Carotene	Supplementation	Intake	Age-related cataract	0.99	[0.92; 1.07]	0.90	[0.83; 0.99]		1.10	[0.98; 1.24]
Johnston 2019	Low red meat	Intake	Intake	Mortality	1.00	[0.84; 1.19]	0.86	[0.79; 0.94]		1.16	[0.96; 1.41]
Johnston 2019	Low red meat	Intake	Intake	Type 2 diabetes	0.95	[0.89; 1.02]	0.76	[0.68; 0.86]		1.25	[1.09; 1.43]
Johnston 2019	Low red meat	Intake	Intake	Colorectal cancer	1.02	[0.89; 1.18]	0.94	[0.85; 1.05]		1.09	[0.91; 1.29]
Jonker 2020	Folic acid	Supplementation	Supplementation+Intake	Small for gestational age	0.98	[0.92; 1.06]	0.80	[0.50; 1.11]		1.22	[0.82; 1.84]
Kong 2014	Vitamins	Supplementation	Intake	Gastric cancer	1.11	[0.94; 1.31]	0.85	[0.66; 1.08]		1.31	[0.97; 1.76]
Lin 2019	Vitamin E	Supplementation	Intake	Bladder cancer	1.10	[0.86; 1.35]	0.84	[0.72; 0.96]		1.31	[1.00; 1.71]
Martinez-Gonzalez 2014b	Olive oil	Supplementation+Intake	Intake	Coronary heart disease	0.81	[0.53; 1.24]	0.96	[0.78; 1.18]		0.84	[0.53; 1.35]
Martinez-Gonzalez 2014b	Olive oil	Supplementation+Intake	Intake	Stroke	0.69	[0.48; 0.98]	0.74	[0.60; 0.92]		0.93	[0.62; 1.41]
Mocellin 2017	Vitamin B6	Supplementation	Intake	Cancer	1.03	[0.94; 1.13]	0.95	[0.90; 1.01]		1.08	[0.97; 1.21]
Sayehmiri 2018	Selenium	Supplementation	Supplementation+Intake	Prostate cancer	0.90	[0.74; 1.09]	0.89	[0.64; 1.23]		1.01	[0.69; 1.48]
Trikalinos 2012	Vitamin E	Supplementation	Supplementation+Intake	Mortality	0.97	[0.91; 1.03]	0.85	[0.78; 0.93]		1.14	[1.02; 1.27]
Vinceti 2018a	Selenium	Supplementation	Intake	Mortality	0.81	[0.49; 1.32]	0.93	[0.83; 1.04]		0.87	[0.52; 1.45]
Vinceti 2018b	Selenium	Supplementation	Intake	Type 2 diabetes	1.11	[1.01; 1.22]	2.28	[1.31; 3.89]		0.49	[0.28; 0.85]
Wien 2012	Folic acid	Supplementation	Supplementation	Cancer	1.07	[1.00; 1.14]	1.03	[0.90; 1.18]		1.04	[0.89; 1.21]
Yang 2016	Folic acid	Supplementation	Supplementation+Intake	Gestational hypertension	0.62	[0.04; 0.94]	1.05	[1.00; 1.11]		0.59	[0.38; 0.91]
Yang 2020	Calcium	Supplementation	Intake	Cardiovascular disease	0.91	[0.56; 1.46]	0.98	[0.90; 1.06]		0.93	[0.57; 1.51]
Yu 2021	Folic acid	Supplementation	Supplementation+Intake	Pre-eclampsia	1.11	[0.75; 1.62]	0.85	[0.76; 0.94]		1.31	[0.88; 1.95]
**Random effects model**										1.05	[0.98; 1.13]
**Prediction interval**											[0.80; 1.38]
Heterogeneity: $I^2 = 48\%$, $\tau^2 = 0.0162$, $p < 0.01$											
**Broadly similar**											
Aguilar-Cordero 2020	Vitamin D	Supplementation	Status	Pre-eclampsia	0.66	[0.42; 1.05]	0.82	[0.60; 1.11]		0.80	[0.46; 1.40]
Chowdhury 2014b	Vitamin D	Supplementation	Status	All-cause mortality	0.98	[0.94; 1.03]	0.69	[0.65; 0.75]		1.42	[1.30; 1.55]
Chung 2011	Vitamin D	Supplementation	Status	Colorectal cancer	1.02	[0.60; 1.74]	0.94	[0.91; 0.97]		1.09	[0.64; 1.85]
Chung 2011	Vitamin D	Supplementation	Status	Breast cancer	0.99	[0.25; 3.99]	0.99	[0.97; 1.01]		1.00	[0.25; 4.00]
Vinceti 2018a	Selenium	Supplementation	Status	Cancer	0.99	[0.86; 1.14]	0.75	[0.59; 0.94]		1.32	[1.01; 1.73]
Yao 2019	Vitamin D	Supplementation	Status	Fractures	1.06	[0.97; 1.15]	0.89	[0.83; 0.96]		1.19	[1.06; 1.33]
Zhang 2016	Selenium	Supplementation	Status	Cardiovascular disease	0.91	[0.74; 1.10]	0.87	[0.76; 0.99]		1.05	[0.82; 1.33]
Zhang 2018	Vitamin D	Supplementation	Status	Gestational diabetes mellitus	0.72	[0.39; 1.31]	0.58	[0.44; 0.76]		1.24	[0.64; 2.40]
Zhou 2017	Vitamin D	Supplementation	Status	Preterm birth	0.58	[0.30; 1.11]	0.88	[0.73; 1.05]		0.66	[0.33; 1.30]
**Random effects model**										1.20	[1.06; 1.36]
**Prediction interval**											[0.90; 1.60]
Heterogeneity: $I^2 = 53\%$, $\tau^2 = 0.0108$, $p = 0.03$											
Test for subgroup differences: $\chi^2_2 = 5.54$, df = 2 ($p = 0.06$)											

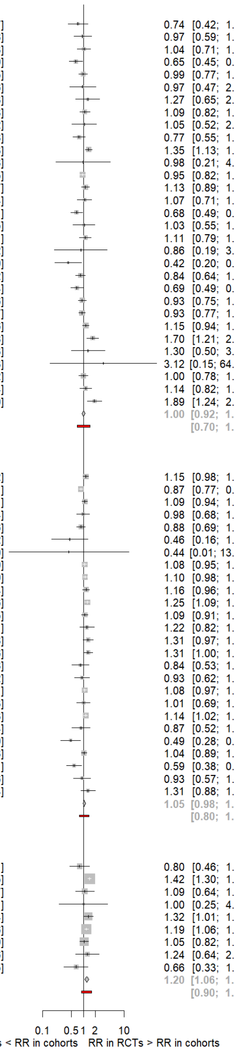

Supplementary Fig. S7 Forest plot, subgroup analysis of BoE-pairs with binary outcomes by intervention/exposure similarity degree

Reference	Intervention/Exposure in SR	Type of intake (RCTs)	Type of exposure (cohorts)	Outcome in SR	RR (RCTs)	95%-CI	RR (cohorts)	95%-CI	Ratio of Risk Ratios	RRR	95%-CI
**More or less identical**											
Aburto 2013	Low-sodium	Intake	Intake	All-cause mortality	0.70	[0.44; 1.14]	0.95	[0.71; 1.27]		0.74	[0.42; 1.29]
Aburto 2013	Low-sodium	Intake	Intake	Cardiovascular disease	0.84	[0.57; 1.23]	0.87	[0.64; 1.18]		0.97	[0.59; 1.58]
Afshin 2014	Nuts	Intake	Intake	Coronary heart disease	0.79	[0.55; 1.14]	0.76	[0.69; 0.83]		1.04	[0.71; 1.51]
Afshin 2014	Nuts	Intake	Intake	Stroke	0.62	[0.44; 0.87]	0.95	[0.83; 1.09]		0.65	[0.45; 0.94]
Afshin 2014	Nuts	Intake	Intake	Type 2 diabetes	0.86	[0.68; 1.08]	0.87	[0.80; 0.95]		0.99	[0.77; 1.27]
Bolland 2015	Calcium	Supplementation	Supplementation	Fractures	0.89	[0.81; 0.96]	1.02	[0.93; 1.11]		0.87	[0.77; 0.99]
Feng 2015	Folic acid	Supplementation	Supplementation	Congenital heart defects	0.58	[0.33; 1.02]	0.60	[0.38; 0.96]		0.97	[0.47; 2.01]
Fu 2021	Vitamin C	Supplementation	Supplementation	Lung cancer	1.30	[0.68; 2.48]	1.02	[0.85; 1.23]		1.27	[0.65; 2.50]
Fu 2021	Vitamin C	Supplementation	Supplementation	Breast cancer	1.11	[0.87; 1.41]	1.02	[0.88; 1.18]		1.09	[0.82; 1.44]
Fu 2021	Vitamin C	Supplementation	Supplementation	Colorectal cancer	0.84	[0.64; 1.10]	0.80	[0.42; 1.52]		1.05	[0.52; 2.11]
Grosso 2015	Mediterranean diet	Intake	Intake	Cardiovascular disease	0.55	[0.39; 0.76]	0.71	[0.65; 0.78]		0.77	[0.55; 1.09]
Jonker 2020	Folic acid	Supplementation	Supplementation	Low birthweight	0.96	[0.88; 1.04]	0.71	[0.60; 0.83]		1.35	[1.13; 1.62]
Kastorini 2011	Mediterranean diet	Intake	Intake	Metabolic syndrome	0.45	[0.24; 0.84]	0.46	[0.11; 1.88]		0.98	[0.21; 4.62]
Kim 2018	Multivitamin/Minerals	Supplementation	Supplementation	Mortality	0.95	[0.83; 1.09]	1.00	[0.96; 1.05]		0.95	[0.82; 1.10]
Kim 2018	Multivitamin/Minerals	Supplementation	Supplementation	Coronary heart disease	0.97	[0.79; 1.19]	0.86	[0.76; 0.97]		1.13	[0.89; 1.43]
Kim 2018	Multivitamin/Minerals	Supplementation	Supplementation	Stroke	1.05	[0.70; 1.56]	0.98	[0.91; 1.04]		1.07	[0.71; 1.61]
Lin 2019	Vitamin E	Supplementation	Intake	Bladder cancer	1.10	[0.86; 1.35]	0.84	[0.72; 0.96]		1.31	[1.00; 1.71]
Lin 2020	Vitamin D	Supplementation	Supplementation	Nephrolithiasis	0.84	[0.65; 1.10]	1.24	[1.01; 1.51]		0.68	[0.49; 0.94]
Lin 2020	Calcium	Supplementation	Supplementation	Nephrolithiasis	1.20	[0.65; 2.21]	1.16	[1.00; 1.35]		1.03	[0.55; 1.94]
Moazzen 2018	Folic acid	Supplementation	Supplementation	Colorectal cancer	1.07	[0.86; 1.43]	0.96	[0.76; 1.21]		1.11	[0.79; 1.57]
Morze 2021	Mediterranean diet	Intake	Intake	Mortality	0.75	[0.17; 3.33]	0.87	[0.82; 0.92]		0.86	[0.19; 3.82]
Morze 2021	Mediterranean diet	Intake	Intake	Breast cancer	0.41	[0.19; 0.87]	0.97	[0.94; 1.00]		0.42	[0.20; 0.91]
Schwingshackl 2015	Mediterranean diet	Intake	Intake	Type 2 diabetes	0.70	[0.54; 0.91]	0.83	[0.74; 0.92]		0.84	[0.64; 1.12]
Schwingshackl 2017	Olive oil	Intake	Intake	Type 2 diabetes	0.60	[0.43; 0.84]	0.87	[0.80; 0.94]		0.69	[0.49; 0.97]
Stratton 2011	Multivitamin	Supplementation	Supplementation	Prostate cancer	0.98	[0.81; 1.20]	1.05	[0.94; 1.16]		0.93	[0.75; 1.17]
Stratton 2011	Vitamin E	Supplementation	Supplementation	Prostate cancer	1.01	[0.90; 1.13]	1.09	[0.94; 1.27]		0.93	[0.77; 1.12]
Stratton 2011	Vitamin C	Supplementation	Supplementation	Prostate cancer	1.10	[0.93; 1.29]	0.96	[0.86; 1.06]		1.15	[0.94; 1.39]
Stratton 2011	β-Carotene	Supplementation	Supplementation	Prostate cancer	1.19	[0.87; 1.65]	0.70	[0.63; 0.78]		1.70	[1.21; 2.38]
Wolf 2017	Multivitamin	Supplementation	Supplementation	Preterm birth	1.09	[0.43; 2.77]	0.84	[0.69; 1.03]		1.30	[0.50; 3.36]
Wolf 2017	Multivitamin	Supplementation	Supplementation	Stillbirth	2.43	[0.12; 50.05]	0.78	[0.59; 1.03]		3.12	[0.15; 64.44]
Zhao 2014	Multivitamin/Minerals	Supplementation	Supplementation	Nuclear cataract	0.73	[0.59; 0.90]	0.73	[0.64; 0.82]		1.00	[0.78; 1.28]
Zhao 2014	Multivitamin/Minerals	Supplementation	Supplementation	Cortical cataract	0.92	[0.69; 1.23]	0.81	[0.68; 0.94]		1.14	[0.82; 1.58]
Zhao 2014	Multivitamin/Minerals	Supplementation	Supplementation	Posterior subcapsular cataract	1.81	[1.30; 2.54]	0.96	[0.72; 1.20]		1.89	[1.24; 2.87]
Random effects model										1.01	[0.92; 1.10]
Prediction interval											[0.70; 1.44]
Heterogeneity: $I^2 = 54\%$, $\tau^2 = 0.0294$, $p < 0.01$											
**Similar but not identical**											
Alexander 2017	Omega-3 fatty acids	Intake+Supplementation	Intake	Cardiovascular disease	0.94	[0.85; 1.05]	0.82	[0.74; 0.92]		1.15	[0.98; 1.33]
Chowdhury 2012	Omega-3 fatty acids	Supplementation	Intake	Stroke	0.98	[0.89; 1.08]	0.90	[0.80; 1.01]		1.09	[0.94; 1.27]
Chowdhury 2014a	α-Linolenic acid	Supplementation	Intake	Coronary heart disease	0.97	[0.69; 1.36]	0.99	[0.86; 1.14]		0.98	[0.68; 1.41]
Chowdhury 2014a	Omega-6 fatty acids	Intake+Supplementation	Intake	Coronary heart disease	0.86	[0.69; 1.07]	0.98	[0.90; 1.06]		0.88	[0.69; 1.11]
Filippini 2020	Green tea	Supplementation	Intake	Prostate cancer	0.50	[0.18; 1.36]	1.09	[0.89; 1.32]		0.46	[0.16; 1.29]
Filippini 2020	Green tea	Supplementation	Intake	Endometrial cancer	0.33	[0.01; 8.15]	0.75	[0.43; 1.30]		0.44	[0.01; 13.14]
Jiang 2019	Vitamin E	Supplementation	Intake	Age-related cataract	0.97	[0.91; 1.03]	0.90	[0.80; 1.00]		1.08	[0.95; 1.22]
Jiang 2019	β-Carotene	Supplementation	Intake	Age-related cataract	0.99	[0.92; 1.07]	0.90	[0.83; 0.99]		1.10	[0.98; 1.24]
Johnston 2019	Low red meat	Intake	Intake	Mortality	1.00	[0.84; 1.19]	0.86	[0.79; 0.94]		1.16	[0.96; 1.41]
Johnston 2019	Low red meat	Intake	Intake	Type 2 diabetes	0.95	[0.89; 1.02]	0.76	[0.68; 0.86]		1.25	[1.09; 1.43]
Johnston 2019	Low red meat	Intake	Intake	Colorectal cancer	1.02	[0.89; 1.18]	0.94	[0.85; 1.05]		1.09	[0.91; 1.29]
Jonker 2020	Folic acid	Supplementation	Supplementation+Intake	Small for gestational age	0.98	[0.92; 1.06]	0.80	[0.50; 1.11]		1.22	[0.82; 1.84]
Kong 2014	Vitamins	Supplementation	Intake	Gastric cancer	1.11	[0.94; 1.31]	0.85	[0.66; 1.08]		1.31	[0.97; 1.76]
Martinez-Gonzalez 2014b	Olive oil	Supplementation+Intake	Intake	Coronary heart disease	0.81	[0.53; 1.24]	0.96	[0.78; 1.18]		0.84	[0.53; 1.35]
Martinez-Gonzalez 2014b	Olive oil	Supplementation+Intake	Intake	Stroke	0.69	[0.48; 0.98]	0.74	[0.60; 0.92]		0.93	[0.62; 1.41]
Mocellin 2017	Vitamin B6	Supplementation	Intake	Cancer	1.03	[0.94; 1.13]	0.95	[0.90; 1.01]		1.08	[0.97; 1.21]
Sayehmiri 2018	Selenium	Supplementation	Supplementation+Intake	Prostate cancer	0.90	[0.74; 1.09]	0.89	[0.64; 1.23]		1.01	[0.69; 1.48]
Trikalinos 2012	Vitamin E	Supplementation	Supplementation+Intake	Mortality	0.97	[0.91; 1.03]	0.85	[0.78; 0.93]		1.14	[1.02; 1.27]
Vinceti 2018a	Selenium	Supplementation	Intake	Mortality	0.81	[0.49; 1.32]	0.93	[0.83; 1.04]		0.87	[0.52; 1.45]
Vinceti 2018b	Selenium	Supplementation	Intake	Type 2 diabetes	1.11	[1.01; 1.22]	2.28	[1.31; 3.89]		0.49	[0.28; 0.85]
Wien 2012	Folic acid	Supplementation	Supplementation	Cancer	1.07	[1.00; 1.14]	1.03	[0.90; 1.18]		1.04	[0.89; 1.21]
Yang 2016	Folic acid	Supplementation	Supplementation+Intake	Gestational hypertension	0.62	[0.04; 0.94]	1.05	[1.00; 1.11]		0.59	[0.38; 0.91]
Yang 2020	Calcium	Supplementation	Intake	Cardiovascular disease	0.91	[0.56; 1.46]	0.98	[0.90; 1.06]		0.93	[0.57; 1.51]
Yu 2021	Folic acid	Supplementation	Supplementation+Intake	Pre-eclampsia	1.11	[0.75; 1.62]	0.85	[0.76; 0.94]		1.31	[0.88; 1.95]
Random effects model										1.06	[0.99; 1.14]
Prediction interval											[0.82; 1.36]
Heterogeneity: $I^2 = 32\%$, $\tau^2 = 0.0135$, $p = 0.07$											
**Broadly similar**											
Aguilar-Cordero 2020	Vitamin D	Supplementation	Status	Pre-eclampsia	0.66	[0.42; 1.05]	0.82	[0.60; 1.11]		0.80	[0.46; 1.40]
Chowdhury 2014b	Vitamin D	Supplementation	Status	All-cause mortality	0.98	[0.94; 1.03]	0.69	[0.65; 0.75]		1.42	[1.30; 1.55]
Chung 2011	Vitamin D	Supplementation	Status	Colorectal cancer	1.02	[0.60; 1.74]	0.94	[0.91; 0.97]		1.09	[0.64; 1.85]
Chung 2011	Vitamin D	Supplementation	Status	Breast cancer	0.99	[0.25; 3.99]	0.99	[0.97; 1.01]		1.00	[0.25; 4.00]
Vinceti 2018a	Selenium	Supplementation	Status	Cancer	0.99	[0.86; 1.14]	0.75	[0.59; 0.94]		1.32	[1.01; 1.73]
Yao 2019	Vitamin D	Supplementation	Status	Fractures	1.06	[0.97; 1.15]	0.89	[0.83; 0.96]		1.19	[1.06; 1.33]
Zhang 2016	Selenium	Supplementation	Status	Cardiovascular disease	0.91	[0.74; 1.10]	0.87	[0.76; 0.99]		1.05	[0.82; 1.33]
Zhang 2018	Vitamin D	Supplementation	Status	Gestational diabetes mellitus	0.72	[0.39; 1.31]	0.58	[0.44; 0.76]		1.24	[0.64; 2.40]
Zhou 2017	Vitamin D	Supplementation	Status	Preterm birth	0.58	[0.30; 1.11]	0.88	[0.73; 1.05]		0.66	[0.33; 1.30]
Random effects model										1.20	[1.06; 1.36]
Prediction interval											[0.90; 1.60]
Heterogeneity: $I^2 = 53\%$, $\tau^2 = 0.0108$, $p = 0.03$											
Test for subgroup differences: $\chi^2_2 = 5.47$, df = 2 ($p = 0.06$)											

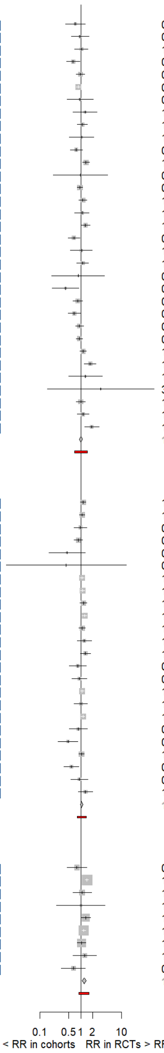

Supplementary Fig. S8 Forest plot, subgroup analysis of BoE-pairs with binary outcomes by comparator similarity degree

Reference	Intervention/Exposure in SR	Type of intake (RCTs)	Type of exposure (cohorts)	Outcome in SR	RR (RCTs)	95%-CI	RR (cohorts)	95%-CI	Ratio of Risk Ratios	RRR	95%-CI
More or less identical											
Aburto 2013	Low-sodium	Intake	Intake	All-cause mortality	0.70	[0.44; 1.14]	0.95	[0.71; 1.27]		0.74	[0.42; 1.29]
Afshin 2014	Nuts	Intake	Intake	Coronary heart disease	0.79	[0.55; 1.14]	0.76	[0.69; 0.83]		1.04	[0.71; 1.51]
Afshin 2014	Nuts	Intake	Intake	Stroke	0.62	[0.44; 0.87]	0.95	[0.83; 1.09]		0.65	[0.45; 0.94]
Afshin 2014	Nuts	Intake	Intake	Type 2 diabetes	0.86	[0.68; 1.08]	0.87	[0.80; 0.95]		0.99	[0.77; 1.27]
Aguilar-Cordero 2020	Vitamin D	Supplementation	Status	Pre-eclampsia	0.66	[0.42; 1.05]	0.82	[0.60; 1.11]		0.80	[0.46; 1.40]
Alexander 2017	Omega-3 fatty acids	Intake+Supplementation	Intake	Cardiovascular disease	0.94	[0.85; 1.05]	0.82	[0.74; 0.92]		1.15	[0.98; 1.33]
Bolland 2015	Calcium	Supplementation	Supplementation	Fractures	0.89	[0.81; 0.96]	1.02	[0.93; 1.11]		0.87	[0.77; 0.99]
Chowdhury 2012	Omega-3 fatty acids	Supplementation	Intake	Stroke	0.98	[0.89; 1.08]	0.90	[0.80; 1.01]		1.09	[0.94; 1.27]
Chowdhury 2014a	α-Linolenic acid	Supplementation	Intake	Coronary heart disease	0.97	[0.69; 1.36]	0.99	[0.86; 1.14]		0.98	[0.68; 1.41]
Chowdhury 2014a	Omega-6 fatty acids	Intake+Supplementation	Intake	Coronary heart disease	0.86	[0.69; 1.07]	0.98	[0.90; 1.06]		0.88	[0.69; 1.11]
Chowdhury 2014b	Vitamin D	Supplementation	Status	All-cause mortality	0.98	[0.94; 1.03]	0.69	[0.65; 0.75]		1.42	[1.30; 1.55]
Chung 2011	Vitamin D	Supplementation	Status	Colorectal cancer	1.02	[0.60; 1.74]	0.94	[0.91; 0.97]		1.09	[0.64; 1.85]
Chung 2011	Vitamin D	Supplementation	Status	Breast cancer	0.99	[0.25; 3.99]	0.99	[0.97; 1.01]		1.00	[0.25; 4.00]
Feng 2015	Folic acid	Supplementation	Supplementation	Congenital heart defects	0.58	[0.33; 1.02]	0.60	[0.38; 0.96]		0.97	[0.47; 2.01]
Filippini 2020	Green tea	Supplementation	Intake	Prostate cancer	0.50	[0.18; 1.36]	1.09	[0.89; 1.32]		0.46	[0.16; 1.29]
Filippini 2020	Green tea	Supplementation	Intake	Endometrial cancer	0.33	[0.01; 8.15]	0.75	[0.43; 1.30]		0.44	[0.01; 13.14]
Fu 2021	Vitamin C	Supplementation	Supplementation	Lung cancer	1.30	[0.68; 2.48]	1.02	[0.85; 1.23]		1.27	[0.65; 2.50]
Fu 2021	Vitamin C	Supplementation	Supplementation	Breast cancer	1.11	[0.87; 1.41]	1.02	[0.88; 1.18]		1.09	[0.82; 1.44]
Fu 2021	Vitamin C	Supplementation	Supplementation	Colorectal cancer	0.84	[0.64; 1.10]	0.80	[0.42; 1.52]		1.05	[0.52; 2.11]
Grosso 2015	Mediterranean diet	Intake	Intake	Cardiovascular disease	0.55	[0.39; 0.76]	0.71	[0.65; 0.78]		0.77	[0.55; 1.09]
Jiang 2019	Vitamin E	Supplementation	Intake	Age-related cataract	0.97	[0.91; 1.03]	0.90	[0.80; 1.00]		1.08	[0.95; 1.22]
Jiang 2019	β-Carotene	Supplementation	Intake	Age-related cataract	0.99	[0.92; 1.07]	0.90	[0.83; 0.99]		1.10	[0.98; 1.24]
Johnston 2019	Low red meat	Intake	Intake	Mortality	1.00	[0.84; 1.19]	0.86	[0.79; 0.94]		1.16	[0.96; 1.41]
Johnston 2019	Low red meat	Intake	Intake	Type 2 diabetes	0.95	[0.89; 1.02]	0.76	[0.68; 0.86]		1.25	[1.09; 1.43]
Johnston 2019	Low red meat	Intake	Intake	Colorectal cancer	1.02	[0.89; 1.18]	0.94	[0.85; 1.05]		1.09	[0.91; 1.29]
Jonker 2020	Folic acid	Supplementation	Supplementation	Low birthweight	0.96	[0.88; 1.04]	0.71	[0.60; 0.83]		1.35	[1.13; 1.62]
Jonker 2020	Folic acid	Supplementation	Supplementation+Intake	Small for gestational age	0.98	[0.92; 1.06]	0.80	[0.50; 1.11]		1.22	[0.82; 1.84]
Kastorini 2011	Mediterranean diet	Intake	Intake	Metabolic syndrome	0.45	[0.24; 0.84]	0.46	[0.11; 1.88]		0.98	[0.21; 4.62]
Kim 2018	Multivitamin/Minerals	Supplementation	Supplementation	Mortality	0.95	[0.83; 1.09]	1.00	[0.96; 1.05]		0.95	[0.82; 1.10]
Kim 2018	Multivitamin/Minerals	Supplementation	Supplementation	Coronary heart disease	0.97	[0.79; 1.19]	0.86	[0.76; 0.97]		1.13	[0.89; 1.43]
Kim 2018	Multivitamin/Minerals	Supplementation	Supplementation	Stroke	1.05	[0.70; 1.56]	0.98	[0.91; 1.04]		1.07	[0.71; 1.61]
Kong 2014	Vitamins	Supplementation	Intake	Gastric cancer	1.11	[0.94; 1.31]	0.85	[0.66; 1.08]		1.31	[0.97; 1.76]
Lin 2019	Vitamin E	Supplementation	Intake	Bladder cancer	1.10	[0.86; 1.35]	0.84	[0.72; 0.96]		1.31	[1.00; 1.71]
Lin 2020	Vitamin D	Supplementation	Supplementation	Nephrolithiasis	0.84	[0.65; 1.10]	1.24	[1.01; 1.51]		0.68	[0.49; 0.94]
Lin 2020	Calcium	Supplementation	Supplementation	Nephrolithiasis	1.20	[0.65; 2.21]	1.16	[1.00; 1.35]		1.03	[0.55; 1.94]
Martinez-Gonzalez 2014b	Olive oil	Supplementation+Intake	Intake	Stroke	0.69	[0.48; 0.98]	0.74	[0.60; 0.92]		0.93	[0.62; 1.41]
Moazzen 2018	Folic acid	Supplementation	Supplementation	Colorectal cancer	1.07	[0.86; 1.43]	0.96	[0.76; 1.21]		1.11	[0.79; 1.57]
Mocellin 2017	Vitamin B6	Supplementation	Intake	Cancer	1.03	[0.94; 1.13]	0.95	[0.90; 1.01]		1.08	[0.97; 1.21]
Morze 2021	Mediterranean diet	Intake	Intake	Mortality	0.75	[0.17; 3.33]	0.87	[0.82; 0.92]		0.86	[0.19; 3.82]
Morze 2021	Mediterranean diet	Intake	Intake	Breast cancer	0.41	[0.19; 0.87]	0.97	[0.94; 1.00]		0.42	[0.20; 0.91]
Sayehmiri 2018	Selenium	Supplementation	Supplementation+Intake	Prostate cancer	0.90	[0.74; 1.09]	0.89	[0.64; 1.23]		1.01	[0.69; 1.48]
Schwingshackl 2015	Mediterranean diet	Intake	Intake	Type 2 diabetes	0.70	[0.54; 0.91]	0.83	[0.74; 0.92]		0.84	[0.64; 1.12]
Schwingshackl 2017	Olive oil	Intake	Intake	Type 2 diabetes	0.60	[0.43; 0.84]	0.87	[0.80; 0.94]		0.69	[0.49; 0.97]
Stratton 2011	Multivitamin	Supplementation	Supplementation	Prostate cancer	0.98	[0.81; 1.20]	1.05	[0.94; 1.16]		0.93	[0.75; 1.17]
Stratton 2011	Vitamin E	Supplementation	Supplementation	Prostate cancer	1.01	[0.90; 1.13]	1.09	[0.94; 1.27]		0.93	[0.77; 1.12]
Stratton 2011	Vitamin C	Supplementation	Supplementation	Prostate cancer	1.10	[0.93; 1.29]	0.96	[0.86; 1.06]		1.15	[0.94; 1.39]
Stratton 2011	β-Carotene	Supplementation	Supplementation	Prostate cancer	1.19	[0.87; 1.65]	0.70	[0.63; 0.78]		1.70	[1.21; 2.38]
Trikalinos 2012	Vitamin E	Supplementation	Supplementation+Intake	Mortality	0.97	[0.91; 1.03]	0.85	[0.78; 0.93]		1.14	[1.02; 1.27]
Vinceti 2018a	Selenium	Supplementation	Status	Cancer	0.99	[0.86; 1.14]	0.75	[0.59; 0.94]		1.32	[1.01; 1.73]
Vinceti 2018a	Selenium	Supplementation	Intake	Mortality	0.81	[0.49; 1.32]	0.93	[0.83; 1.04]		0.87	[0.52; 1.45]
Vinceti 2018b	Selenium	Supplementation	Intake	Type 2 diabetes	1.11	[1.01; 1.22]	2.28	[1.31; 3.89]		0.49	[0.28; 0.85]
Wolf 2017	Multivitamin	Supplementation	Supplementation	Preterm birth	1.09	[0.43; 2.77]	0.84	[0.69; 1.03]		1.30	[0.50; 3.36]
Wolf 2017	Multivitamin	Supplementation	Supplementation	Stillbirth	2.43	[0.12; 50.05]	0.78	[0.59; 1.03]		3.12	[0.15; 64.44]
Yang 2016	Folic acid	Supplementation	Supplementation+Intake	Gestational hypertension	0.62	[0.04; 0.94]	1.05	[1.00; 1.11]		0.59	[0.38; 0.91]
Yang 2020	Calcium	Supplementation	Intake	Cardiovascular disease	0.91	[0.56; 1.46]	0.98	[0.90; 1.06]		0.93	[0.57; 1.51]
Yao 2019	Vitamin D	Supplementation	Status	Fractures	1.06	[0.97; 1.15]	0.89	[0.83; 0.96]		1.19	[1.06; 1.33]
Yu 2021	Folic acid	Supplementation	Supplementation+Intake	Pre-eclampsia	1.11	[0.75; 1.62]	0.85	[0.76; 0.94]		1.31	[0.88; 1.95]
Zhang 2016	Selenium	Supplementation	Status	Cardiovascular disease	0.91	[0.74; 1.10]	0.87	[0.76; 0.99]		1.05	[0.82; 1.33]
Zhang 2018	Vitamin D	Supplementation	Status	Gestational diabetes mellitus	0.72	[0.39; 1.31]	0.58	[0.44; 0.76]		1.24	[0.64; 2.40]
Zhao 2014	Multivitamin/Minerals	Supplementation	Supplementation	Nuclear cataract	0.73	[0.59; 0.90]	0.73	[0.64; 0.82]		1.00	[0.78; 1.28]
Zhao 2014	Multivitamin/Minerals	Supplementation	Supplementation	Cortical cataract	0.92	[0.69; 1.23]	0.81	[0.68; 0.94]		1.14	[0.82; 1.58]
Zhao 2014	Multivitamin/Minerals	Supplementation	Supplementation	Posterior subcapsular cataract	1.81	[1.30; 2.54]	0.96	[0.72; 1.20]		1.89	[1.24; 2.87]
Zhou 2017	Vitamin D	Supplementation	Status	Preterm birth	0.58	[0.30; 1.11]	0.88	[0.73; 1.05]		0.66	[0.33; 1.30]
Random effects model										1.05	[0.99; 1.11]
Prediction interval											[0.76; 1.44]
Heterogeneity: $I^2 = 61\%$, $\tau^2 = 0.0243$, $p < 0.01$											
Similar but not identical											
Aburto 2013	Low-sodium	Intake	Intake	Cardiovascular disease	0.84	[0.57; 1.23]	0.87	[0.64; 1.18]		0.97	[0.59; 1.58]
Martinez-Gonzalez 2014b	Olive oil	Supplementation+Intake	Intake	Coronary heart disease	0.81	[0.53; 1.24]	0.96	[0.78; 1.18]		0.84	[0.53; 1.35]
Wien 2012	Folic acid	Supplementation	Supplementation	Cancer	1.07	[1.00; 1.14]	1.03	[0.90; 1.18]		1.04	[0.89; 1.21]
Random effects model										1.02	[0.89; 1.16]
Prediction interval											[0.42; 2.46]
Heterogeneity: $I^2 = 0\%$, $\tau^2 = 0$, $p = 0.70$											
Test for subgroup differences: $\chi^2_1 = 0.15$, df = 1 ($p = 0.69$)											

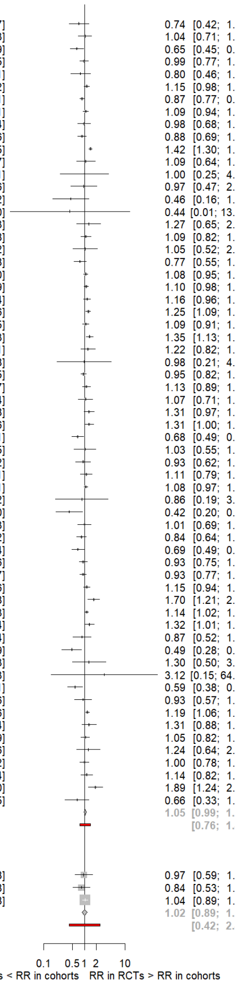

Supplementary Fig. S9 Forest plot, subgroup analysis of BoE-pairs with binary outcomes by outcome similarity degree

Reference	Intervention/Exposure in SR	Type of intake (RCTs)	Type of exposure (cohorts)	Outcome in SR	RR (RCTs)	95%-CI	RR (cohorts)	95%-CI	RRR	95%-CI
**Moderate**										
Filippini 2020	Green tea	Prostate cancer	Supplementation	Intake	0.50	[0.18; 1.36]	1.09	[0.89; 1.32]	0.46	[0.16; 1.29]
Filippini 2020	Green tea	Endometrial cancer	Supplementation	Intake	0.33	[0.01; 8.15]	0.75	[0.43; 1.30]	0.44	[0.01; 13.14]
Vinceti 2018a	Selenium	Cancer	Supplementation	Status	0.99	[0.86; 1.14]	0.75	[0.59; 0.94]	1.32	[1.01; 1.73]
Vinceti 2018a	Selenium	Mortality	Supplementation	Intake	0.81	[0.49; 1.32]	0.93	[0.83; 1.04]	0.87	[0.52; 1.45]
Random effects model									0.98	[0.65; 1.49]
Prediction interval										[0.23; 4.19]
Heterogeneity: $I^2 = 45\%$, $\tau^2 = 0.0681$, $p = 0.14$										
**Low**										
Johnston 2019	Low red meat	Mortality	Intake	Intake	1.00	[0.84; 1.19]	0.86	[0.79; 0.94]	1.16	[0.96; 1.41]
Johnston 2019	Low red meat	Type 2 diabetes	Intake	Intake	0.95	[0.89; 1.02]	0.76	[0.68; 0.86]	1.25	[1.09; 1.43]
Johnston 2019	Low red meat	Colorectal cancer	Intake	Intake	1.02	[0.89; 1.18]	0.94	[0.85; 1.05]	1.09	[0.91; 1.29]
Morze 2021	Mediterranean diet	Mortality	Intake	Intake	0.75	[0.17; 3.33]	0.87	[0.82; 0.92]	0.86	[0.19; 3.82]
Morze 2021	Mediterranean diet	Breast cancer	Intake	Intake	0.41	[0.19; 0.87]	0.97	[0.94; 1.00]	0.42	[0.20; 0.91]
Schwingshackl 2015	Mediterranean diet	Type 2 diabetes	Intake	Intake	0.70	[0.54; 0.91]	0.83	[0.74; 0.92]	0.84	[0.64; 1.12]
Wien 2012	Folic acid	Cancer	Supplementation	Supplementation	1.07	[1.00; 1.14]	1.03	[0.90; 1.18]	1.04	[0.89; 1.21]
Yao 2019	Vitamin D	Fractures	Supplementation	Status	1.06	[0.97; 1.15]	0.89	[0.83; 0.96]	1.19	[1.06; 1.33]
Random effects model									1.06	[0.91; 1.24]
Prediction interval										[0.66; 1.72]
Heterogeneity: $I^2 = 54\%$, $\tau^2 = 0.0324$, $p = 0.03$										
**Critically low**										
Aburto 2013	Low-sodium	All-cause mortality	Intake	Intake	0.70	[0.44; 1.14]	0.95	[0.71; 1.27]	0.74	[0.42; 1.29]
Aburto 2013	Low-sodium	Cardiovascular disease	Intake	Intake	0.84	[0.57; 1.23]	0.87	[0.64; 1.18]	0.97	[0.59; 1.58]
Afshin 2014	Nuts	Coronary heart disease	Intake	Intake	0.79	[0.55; 1.14]	0.76	[0.69; 0.83]	1.04	[0.71; 1.51]
Afshin 2014	Nuts	Stroke	Intake	Intake	0.62	[0.44; 0.87]	0.95	[0.83; 1.09]	0.65	[0.45; 0.94]
Afshin 2014	Nuts	Type 2 diabetes	Intake	Intake	0.86	[0.68; 1.08]	0.87	[0.80; 0.95]	0.99	[0.77; 1.27]
Aguilar-Cordero 2020	Vitamin D	Pre-eclampsia	Supplementation	Status	0.66	[0.42; 1.05]	0.82	[0.60; 1.11]	0.80	[0.46; 1.40]
Alexander 2017	Omega-3 fatty acids	Cardiovascular disease	Intake+Supplementation	Intake	0.94	[0.85; 1.05]	0.82	[0.74; 0.92]	1.15	[0.98; 1.33]
Bolland 2015	Calcium	Fractures	Supplementation	Supplementation	0.89	[0.81; 0.96]	1.02	[0.93; 1.11]	0.87	[0.77; 0.99]
Chowdhury 2012	Omega-3 fatty acids	Stroke	Supplementation	Intake	0.98	[0.89; 1.08]	0.90	[0.80; 1.01]	1.09	[0.94; 1.27]
Chowdhury 2014a	α-Linolenic acid	Coronary heart disease	Supplementation	Intake	0.97	[0.69; 1.36]	0.99	[0.86; 1.14]	0.98	[0.68; 1.41]
Chowdhury 2014a	Omega-6 fatty acids	Coronary heart disease	Intake+Supplementation	Intake	0.86	[0.69; 1.07]	0.98	[0.90; 1.06]	0.88	[0.69; 1.11]
Chowdhury 2014b	Vitamin D	All-cause mortality	Supplementation	Status	0.98	[0.94; 1.03]	0.69	[0.65; 0.75]	1.42	[1.30; 1.55]
Chung 2011	Vitamin D	Colorectal cancer	Supplementation	Status	1.02	[0.60; 1.74]	0.94	[0.91; 0.97]	1.09	[0.64; 1.85]
Chung 2011	Vitamin D	Breast cancer	Supplementation	Status	0.99	[0.25; 3.99]	0.99	[0.97; 1.01]	1.00	[0.25; 4.00]
Feng 2015	Folic acid	Congenital heart defects	Supplementation	Supplementation	0.58	[0.33; 1.02]	0.60	[0.38; 0.96]	0.97	[0.47; 2.01]
Fu 2021	Vitamin C	Lung cancer	Supplementation	Supplementation	1.30	[0.68; 2.48]	1.02	[0.85; 1.23]	1.27	[0.65; 2.50]
Fu 2021	Vitamin C	Breast cancer	Supplementation	Supplementation	1.11	[0.87; 1.41]	1.02	[0.88; 1.18]	1.09	[0.82; 1.44]
Fu 2021	Vitamin C	Colorectal cancer	Supplementation	Supplementation	0.84	[0.64; 1.10]	0.80	[0.42; 1.52]	1.05	[0.52; 2.11]
Grosso 2015	Mediterranean diet	Cardiovascular disease	Intake	Intake	0.55	[0.39; 0.76]	0.71	[0.65; 0.78]	0.77	[0.55; 1.09]
Jiang 2019	Vitamin E	Age-related cataract	Supplementation	Intake	0.97	[0.91; 1.03]	0.90	[0.80; 1.00]	1.08	[0.95; 1.22]
Jiang 2019	β-Carotene	Age-related cataract	Supplementation	Intake	0.99	[0.92; 1.07]	0.90	[0.83; 0.99]	1.10	[0.98; 1.24]
Jonker 2020	Folic acid	Low birthweight	Supplementation	Supplementation	0.96	[0.88; 1.04]	0.71	[0.60; 0.83]	1.35	[1.13; 1.62]
Jonker 2020	Folic acid	Small for gestational age	Supplementation	Supplementation+Intake	0.98	[0.92; 1.06]	0.80	[0.50; 1.11]	1.22	[0.82; 1.84]
Kastorini 2011	Mediterranean diet	Metabolic syndrome	Intake	Intake	0.45	[0.24; 0.84]	0.46	[0.11; 1.88]	0.98	[0.21; 4.62]
Kim 2018	Multivitamin/Minerals	Mortality	Supplementation	Supplementation	0.95	[0.83; 1.09]	1.00	[0.96; 1.05]	0.95	[0.82; 1.10]
Kim 2018	Multivitamin/Minerals	Coronary heart disease	Supplementation	Supplementation	0.97	[0.79; 1.19]	0.86	[0.76; 0.97]	1.13	[0.89; 1.43]
Kim 2018	Multivitamin/Minerals	Stroke	Supplementation	Supplementation	1.05	[0.70; 1.56]	0.98	[0.91; 1.04]	1.07	[0.71; 1.61]
Kong 2014	Vitamins	Gastric cancer	Supplementation	Intake	1.11	[0.94; 1.31]	0.85	[0.66; 1.08]	1.31	[0.97; 1.76]
Lin 2019	Vitamin E	Bladder cancer	Supplementation	Intake	1.10	[0.86; 1.35]	0.84	[0.72; 0.96]	1.31	[1.00; 1.71]
Lin 2020	Vitamin D	Nephrolithiasis	Supplementation	Supplementation	0.84	[0.65; 1.10]	1.24	[1.01; 1.51]	0.68	[0.49; 0.94]
Lin 2020	Calcium	Nephrolithiasis	Supplementation	Supplementation	1.20	[0.65; 2.21]	1.16	[1.00; 1.35]	1.03	[0.55; 1.94]
Martinez-Gonzalez 2014b	Olive oil	Coronary heart disease	Supplementation+Intake	Intake	0.81	[0.53; 1.24]	0.96	[0.78; 1.18]	0.84	[0.53; 1.35]
Martinez-Gonzalez 2014b	Olive oil	Stroke	Supplementation+Intake	Intake	0.69	[0.48; 0.98]	0.74	[0.60; 0.92]	0.93	[0.62; 1.41]
Moazzen 2018	Folic acid	Colorectal cancer	Supplementation	Supplementation	1.07	[0.86; 1.43]	0.96	[0.76; 1.21]	1.11	[0.79; 1.57]
Mocellin 2017	Vitamin B6	Cancer	Supplementation	Intake	1.03	[0.94; 1.13]	0.95	[0.90; 1.01]	1.08	[0.97; 1.21]
Sayehmiri 2018	Selenium	Prostate cancer	Supplementation	Supplementation+Intake	0.90	[0.74; 1.09]	0.89	[0.64; 1.23]	1.01	[0.69; 1.48]
Schwingshackl 2017	Olive oil	Type 2 diabetes	Intake	Intake	0.60	[0.43; 0.84]	0.87	[0.80; 0.94]	0.69	[0.49; 0.97]
Stratton 2011	Multivitamin	Prostate cancer	Supplementation	Supplementation	0.98	[0.81; 1.20]	1.05	[0.94; 1.16]	0.93	[0.75; 1.17]
Stratton 2011	Vitamin E	Prostate cancer	Supplementation	Supplementation	1.01	[0.90; 1.13]	1.09	[0.94; 1.27]	0.93	[0.77; 1.12]
Stratton 2011	Vitamin C	Prostate cancer	Supplementation	Supplementation	1.10	[0.93; 1.29]	0.96	[0.86; 1.06]	1.15	[0.94; 1.39]
Stratton 2011	β-Carotene	Prostate cancer	Supplementation	Supplementation	1.19	[0.87; 1.65]	0.70	[0.63; 0.78]	1.70	[1.21; 2.38]
Trikalinos 2012	Vitamin E	Mortality	Supplementation	Supplementation+Intake	0.97	[0.91; 1.03]	0.85	[0.78; 0.93]	1.14	[1.02; 1.27]
Vinceti 2018b	Selenium	Type 2 diabetes	Supplementation	Intake	1.11	[1.01; 1.22]	2.28	[1.31; 3.89]	0.49	[0.28; 0.85]
Wolf 2017	Multivitamin	Preterm birth	Supplementation	Supplementation	1.09	[0.43; 2.77]	0.84	[0.69; 1.03]	1.30	[0.50; 3.36]
Wolf 2017	Multivitamin	Stillbirth	Supplementation	Supplementation	2.43	[0.12; 50.05]	0.78	[0.59; 1.03]	3.12	[0.15; 64.44]
Yang 2016	Folic acid	Gestational hypertension	Supplementation	Supplementation+Intake	0.62	[0.04; 0.94]	1.05	[1.00; 1.11]	0.59	[0.38; 0.91]
Yang 2020	Calcium	Cardiovascular disease	Supplementation	Intake	0.91	[0.56; 1.46]	0.98	[0.90; 1.06]	0.93	[0.57; 1.51]
Yu 2021	Folic acid	Pre-eclampsia	Supplementation	Supplementation+Intake	1.11	[0.75; 1.62]	0.85	[0.76; 0.94]	1.31	[0.88; 1.95]
Zhang 2016	Selenium	Cardiovascular disease	Supplementation	Status	0.91	[0.74; 1.10]	0.87	[0.76; 0.99]	1.05	[0.82; 1.33]
Zhang 2018	Vitamin D	Gestational diabetes mellitus	Supplementation	Status	0.72	[0.39; 1.31]	0.58	[0.44; 0.76]	1.24	[0.64; 2.40]
Zhao 2014	Multivitamin/Minerals	Nuclear cataract	Supplementation	Supplementation	0.73	[0.59; 0.90]	0.73	[0.64; 0.82]	1.00	[0.78; 1.28]
Zhao 2014	Multivitamin/Minerals	Cortical cataract	Supplementation	Supplementation	0.92	[0.69; 1.23]	0.81	[0.68; 0.94]	1.14	[0.82; 1.58]
Zhao 2014	Multivitamin/Minerals	Posterior subcapsular cataract	Supplementation	Supplementation	1.81	[1.30; 2.54]	0.96	[0.72; 1.20]	1.89	[1.24; 2.87]
Zhou 2017	Vitamin D	Preterm birth	Supplementation	Status	0.58	[0.30; 1.11]	0.88	[0.73; 1.05]	0.66	[0.33; 1.30]
Random effects model									1.04	[0.98; 1.10]
Prediction interval										[0.77; 1.41]
Heterogeneity: $I^2 = 62\%$, $\tau^2 = 0.0219$, $p < 0.01$										
Test for subgroup differences: $\chi^2_2 = 0.14$, df = 2 ($p = 0.93$)										

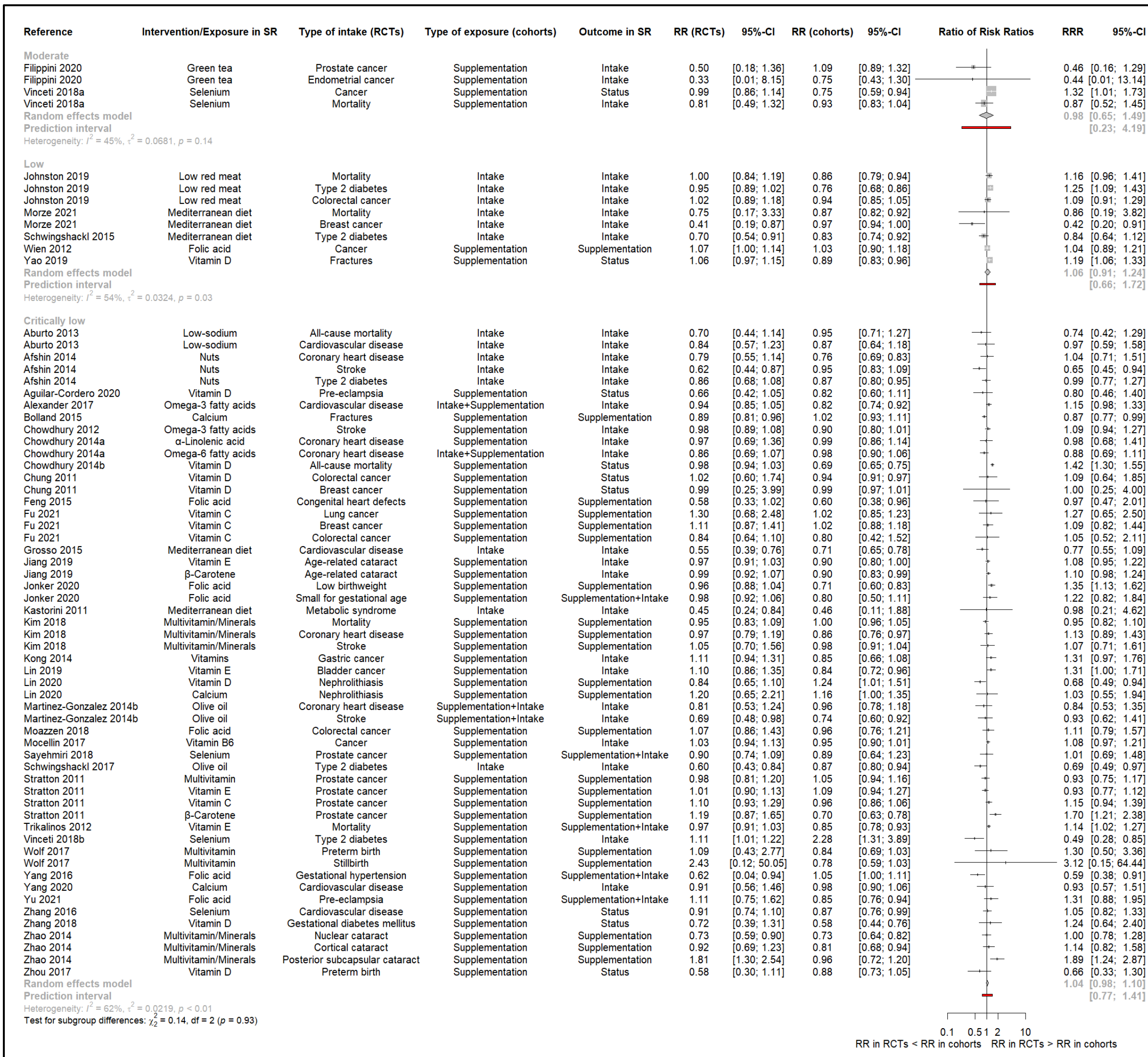

Supplementary Fig. S10 Forest plot, subgroup analysis of BoE-pairs with binary outcomes by AMSTAR 2 rating

Reference	Intervention/Exposure in SR	Type of intake (RCTs)	Type of exposure (cohorts)	Outcome in SR	RR (RCTs)	95%-CI	RR (cohorts)	95%-CI	Ratio of Risk Ratios	RRR	95%-CI	Weight
Aburto 2013	Low-sodium	Intake	Intake	All-cause mortality	0.70	[0.44; 1.14]	0.95	[0.71; 1.27]		0.74	[0.42; 1.29]	1.3%
Afshin 2014	Nuts	Intake	Intake	Stroke	0.62	[0.44; 0.87]	0.95	[0.83; 1.09]		0.65	[0.45; 0.94]	2.2%
Aguilar-Cordero 2020	Vitamin D	Supplementation	Status	Pre-eclampsia	0.66	[0.42; 1.05]	0.82	[0.60; 1.11]		0.80	[0.46; 1.40]	1.3%
Alexander 2017	Omega-3 fatty acids	Intake+Supplementation	Intake	Cardiovascular disease	0.94	[0.85; 1.05]	0.82	[0.74; 0.92]		1.15	[0.98; 1.33]	3.7%
Bolland 2015	Calcium	Supplementation	Supplementation	Fractures	0.89	[0.81; 0.96]	1.02	[0.93; 1.11]		0.87	[0.77; 0.99]	3.9%
Chowdhury 2012	Omega-3 fatty acids	Supplementation	Intake	Stroke	0.98	[0.89; 1.08]	0.90	[0.80; 1.01]		1.09	[0.94; 1.27]	3.7%
Chowdhury 2014a	Omega-6 fatty acids	Intake+Supplementation	Intake	Coronary heart disease	0.86	[0.69; 1.07]	0.98	[0.90; 1.06]		0.88	[0.69; 1.11]	3.1%
Chowdhury 2014b	Vitamin D	Supplementation	Status	All-cause mortality	0.98	[0.94; 1.03]	0.69	[0.65; 0.75]		1.42	[1.30; 1.55]	4.1%
Chung 2011	Vitamin D	Supplementation	Status	Colorectal cancer	1.02	[0.60; 1.74]	0.94	[0.91; 0.97]		1.09	[0.64; 1.85]	1.4%
Feng 2015	Folic acid	Supplementation	Supplementation	Congenital heart defects	0.58	[0.33; 1.02]	0.60	[0.38; 0.96]		0.97	[0.47; 2.01]	0.9%
Filippini 2020	Green tea	Supplementation	Intake	Prostate cancer	0.50	[0.18; 1.36]	1.09	[0.89; 1.32]		0.46	[0.16; 1.29]	0.5%
Fu 2021	Vitamin C	Supplementation	Supplementation	Lung cancer	1.30	[0.68; 2.48]	1.02	[0.85; 1.23]		1.27	[0.65; 2.50]	1.0%
Grosso 2015	Mediterranean diet	Intake	Intake	Cardiovascular disease	0.55	[0.39; 0.76]	0.71	[0.65; 0.78]		0.77	[0.55; 1.09]	2.3%
Jiang 2019	Vitamin E	Supplementation	Intake	Age-related cataract	0.97	[0.91; 1.03]	0.90	[0.80; 1.00]		1.08	[0.95; 1.22]	3.9%
Johnston 2019	Low red meat	Intake	Intake	Mortality	1.00	[0.84; 1.19]	0.86	[0.79; 0.94]		1.16	[0.96; 1.41]	3.4%
Jonker 2020	Folic acid	Supplementation	Supplementation	Low birthweight	0.96	[0.88; 1.04]	0.71	[0.60; 0.83]		1.35	[1.13; 1.62]	3.5%
Kastorini 2011	Mediterranean diet	Intake	Intake	Metabolic syndrome	0.45	[0.24; 0.84]	0.46	[0.11; 1.88]		0.98	[0.21; 4.62]	0.2%
Kim 2018	Multivitamin/Minerals	Supplementation	Supplementation	Coronary heart disease	0.97	[0.79; 1.19]	0.86	[0.76; 0.97]		1.13	[0.89; 1.43]	3.1%
Kong 2014	Vitamins	Supplementation	Intake	Gastric cancer	1.11	[0.94; 1.31]	0.85	[0.66; 1.08]		1.31	[0.97; 1.76]	2.6%
Lin 2019	Vitamin E	Supplementation	Intake	Bladder cancer	1.10	[0.86; 1.35]	0.84	[0.72; 0.96]		1.31	[1.00; 1.71]	2.8%
Lin 2020	Vitamin D	Supplementation	Supplementation	Nephrolithiasis	0.84	[0.65; 1.10]	1.24	[1.01; 1.51]		0.68	[0.49; 0.94]	2.4%
Martinez-Gonzalez 2014b	Olive oil	Supplementation+Intake	Intake	Stroke	0.69	[0.48; 0.98]	0.74	[0.60; 0.92]		0.93	[0.62; 1.41]	1.9%
Moazzen 2018	Folic acid	Supplementation	Supplementation	Colorectal cancer	1.07	[0.86; 1.43]	0.96	[0.76; 1.21]		1.11	[0.79; 1.57]	2.3%
Mocellin 2017	Vitamin B6	Supplementation	Intake	Cancer	1.03	[0.94; 1.13]	0.95	[0.90; 1.01]		1.08	[0.97; 1.21]	4.0%
Morze 2021	Mediterranean diet	Intake	Intake	Breast cancer	0.41	[0.19; 0.87]	0.97	[0.94; 1.00]		0.42	[0.20; 0.91]	0.8%
Sayehmiri 2018	Selenium	Supplementation	Supplementation+Intake	Prostate cancer	0.90	[0.74; 1.09]	0.89	[0.64; 1.23]		1.01	[0.69; 1.48]	2.1%
Schwingshackl 2015	Mediterranean diet	Intake	Intake	Type 2 diabetes	0.70	[0.54; 0.91]	0.83	[0.74; 0.92]		0.84	[0.64; 1.12]	2.7%
Schwingshackl 2017	Olive oil	Intake	Intake	Type 2 diabetes	0.60	[0.43; 0.84]	0.87	[0.80; 0.94]		0.69	[0.49; 0.97]	2.3%
Stratton 2011	Vitamin E	Supplementation	Supplementation	Prostate cancer	1.01	[0.90; 1.13]	1.09	[0.94; 1.27]		0.93	[0.77; 1.12]	3.4%
Trikalinos 2012	Vitamin E	Supplementation	Supplementation+Intake	Mortality	0.97	[0.91; 1.03]	0.85	[0.78; 0.93]		1.14	[1.02; 1.27]	4.0%
Vinceti 2018a	Selenium	Supplementation	Status	Cancer	0.99	[0.86; 1.14]	0.75	[0.59; 0.94]		1.32	[1.01; 1.73]	2.8%
Vinceti 2018b	Selenium	Supplementation	Intake	Type 2 diabetes	1.11	[1.01; 1.22]	2.28	[1.31; 3.89]		0.49	[0.28; 0.85]	1.3%
Wien 2012	Folic acid	Supplementation	Supplementation	Cancer	1.07	[1.00; 1.14]	1.03	[0.90; 1.18]		1.04	[0.89; 1.21]	3.7%
Wolf 2017	Multivitamin	Supplementation	Supplementation	Preterm birth	1.09	[0.43; 2.77]	0.84	[0.69; 1.03]		1.30	[0.50; 3.36]	0.6%
Yang 2016	Folic acid	Supplementation	Supplementation+Intake	Gestational hypertension	0.62	[0.04; 0.94]	1.05	[1.00; 1.11]		0.59	[0.38; 0.91]	1.8%
Yang 2020	Calcium	Supplementation	Intake	Cardiovascular disease	0.91	[0.56; 1.46]	0.98	[0.90; 1.06]		0.93	[0.57; 1.51]	1.6%
Yao 2019	Vitamin D	Supplementation	Status	Fractures	1.06	[0.97; 1.15]	0.89	[0.83; 0.96]		1.19	[1.06; 1.33]	4.0%
Yu 2021	Folic acid	Supplementation	Supplementation+Intake	Pre-eclampsia	1.11	[0.75; 1.62]	0.85	[0.76; 0.94]		1.31	[0.88; 1.95]	2.0%
Zhang 2016	Selenium	Supplementation	Status	Cardiovascular disease	0.91	[0.74; 1.10]	0.87	[0.76; 0.99]		1.05	[0.82; 1.33]	3.1%
Zhang 2018	Vitamin D	Supplementation	Status	Gestational diabetes mellitus	0.72	[0.39; 1.31]	0.58	[0.44; 0.76]		1.24	[0.64; 2.40]	1.0%
Zhao 2014	Multivitamin/Minerals	Supplementation	Supplementation	Cortical cataract	0.92	[0.69; 1.23]	0.81	[0.68; 0.94]		1.14	[0.82; 1.58]	2.4%
Zhou 2017	Vitamin D	Supplementation	Status	Preterm birth	0.58	[0.30; 1.11]	0.88	[0.73; 1.05]		0.66	[0.33; 1.30]	1.0%
**Random effects model**										**1.01**	**[0.94; 1.09]**	**100.0%**
**Prediction interval**											**[0.69; 1.48]**	

Heterogeneity: $I^2 = 69\%$, $\tau^2 = 0.0343$, $p < 0.01$

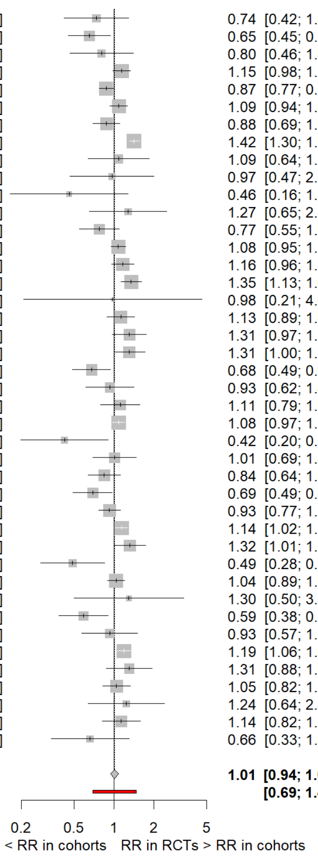

Supplementary Fig. S11 Forest plot, sensitivity analysis including one BoE-pair per systematic review

Reference	Intervention/Exposure in SR	Type of intake (RCTs)	Type of exposure (cohorts)	Outcome in SR	RR (RCTs)	95%-CI	RR (cohorts)	95%-CI	RRR	95%-CI	Weight
Aburto 2013	Low-sodium	Intake	Intake	All-cause mortality	0.70	[0.44; 1.14]	0.95	[0.71; 1.27]	0.74	[0.42; 1.29]	0.8%
Aburto 2013	Low-sodium	Intake	Intake	Cardiovascular disease	0.84	[0.57; 1.23]	0.87	[0.64; 1.18]	0.97	[0.59; 1.58]	0.9%
Afshin 2014	Nuts	Intake	Intake	Coronary heart disease	0.79	[0.55; 1.14]	0.76	[0.69; 0.83]	1.04	[0.71; 1.51]	1.4%
Afshin 2014	Nuts	Intake	Intake	Stroke	0.62	[0.44; 0.87]	0.95	[0.83; 1.09]	0.65	[0.45; 0.94]	1.5%
Afshin 2014	Nuts	Intake	Intake	Type 2 diabetes	0.86	[0.68; 1.08]	0.87	[0.80; 0.95]	0.99	[0.77; 1.27]	2.4%
Aguilar-Cordero 2020	Vitamin D	Supplementation	Status	Pre-eclampsia	0.66	[0.42; 1.05]	0.82	[0.60; 1.11]	0.80	[0.46; 1.40]	0.8%
Alexander 2017	Omega-3 fatty acids	Intake+Supplementation	Intake	Cardiovascular disease	0.94	[0.85; 1.05]	0.82	[0.74; 0.92]	1.15	[0.98; 1.33]	3.6%
Chowdhury 2012	Omega-3 fatty acids	Supplementation	Intake	Stroke	0.98	[0.89; 1.08]	0.90	[0.80; 1.01]	1.09	[0.94; 1.27]	3.7%
Chowdhury 2014a	α-Linolenic acid	Supplementation	Intake	Coronary heart disease	0.97	[0.69; 1.36]	0.99	[0.86; 1.14]	0.98	[0.68; 1.41]	1.5%
Chowdhury 2014a	Omega-6 fatty acids	Intake+Supplementation	Intake	Coronary heart disease	0.86	[0.69; 1.07]	0.98	[0.90; 1.06]	0.88	[0.69; 1.11]	2.6%
Chowdhury 2014b	Vitamin D	Supplementation	Status	All-cause mortality	0.98	[0.94; 1.03]	0.69	[0.65; 0.75]	1.42	[1.30; 1.55]	4.6%
Chung 2011	Vitamin D	Supplementation	Status	Colorectal cancer	1.02	[0.60; 1.74]	0.94	[0.91; 0.97]	1.09	[0.64; 1.85]	0.8%
Chung 2011	Vitamin D	Supplementation	Status	Breast cancer	0.99	[0.25; 3.99]	0.99	[0.97; 1.01]	1.00	[0.25; 4.00]	0.1%
Feng 2015	Folic acid	Supplementation	Supplementation	Congenital heart defects	0.58	[0.33; 1.02]	0.60	[0.38; 0.96]	0.97	[0.47; 2.01]	0.5%
Filippini 2020	Green tea	Supplementation	Intake	Endometrial cancer	0.33	[0.01; 8.15]	0.75	[0.43; 1.30]	0.44	[0.01; 13.14]	0.0%
Fu 2021	Vitamin C	Supplementation	Supplementation	Colorectal cancer	0.84	[0.64; 1.10]	0.80	[0.42; 1.52]	1.05	[0.52; 2.11]	0.5%
Grosso 2015	Mediterranean diet	Intake	Intake	Cardiovascular disease	0.55	[0.39; 0.76]	0.71	[0.65; 0.78]	0.77	[0.55; 1.09]	1.6%
Jiang 2019	Vitamin E	Supplementation	Intake	Age-related cataract	0.97	[0.91; 1.03]	0.90	[0.80; 1.00]	1.08	[0.95; 1.22]	4.0%
Jiang 2019	β-Carotene	Supplementation	Intake	Age-related cataract	0.99	[0.92; 1.07]	0.90	[0.83; 0.99]	1.10	[0.98; 1.24]	4.2%
Johnston 2019	Low red meat	Intake	Intake	Mortality	1.00	[0.84; 1.19]	0.86	[0.79; 0.94]	1.16	[0.96; 1.41]	3.1%
Johnston 2019	Low red meat	Intake	Intake	Type 2 diabetes	0.95	[0.89; 1.02]	0.76	[0.68; 0.86]	1.25	[1.09; 1.43]	3.9%
Johnston 2019	Low red meat	Intake	Intake	Colorectal cancer	1.02	[0.89; 1.18]	0.94	[0.85; 1.05]	1.09	[0.91; 1.29]	3.3%
Jonker 2020	Folic acid	Supplementation	Supplementation	Low birthweight	0.96	[0.88; 1.04]	0.71	[0.60; 0.83]	1.35	[1.13; 1.62]	3.2%
Jonker 2020	Folic acid	Supplementation	Supplementation+Intake	Small for gestational age	0.98	[0.92; 1.06]	0.80	[0.50; 1.11]	1.22	[0.82; 1.84]	1.3%
Kastorini 2011	Mediterranean diet	Intake	Intake	Metabolic syndrome	0.45	[0.24; 0.84]	0.46	[0.11; 1.88]	0.98	[0.21; 4.62]	0.1%
Kim 2018	Multivitamin/Minerals	Supplementation	Supplementation	Coronary heart disease	0.97	[0.79; 1.19]	0.86	[0.76; 0.97]	1.13	[0.89; 1.43]	2.5%
Kim 2018	Multivitamin/Minerals	Supplementation	Supplementation	Stroke	1.05	[0.70; 1.56]	0.98	[0.91; 1.04]	1.07	[0.71; 1.61]	1.3%
Kong 2014	Vitamins	Supplementation	Intake	Gastric cancer	1.11	[0.94; 1.31]	0.85	[0.66; 1.08]	1.31	[0.97; 1.76]	2.0%
Lin 2019	Vitamin E	Supplementation	Intake	Bladder cancer	1.10	[0.86; 1.35]	0.84	[0.72; 0.96]	1.31	[1.00; 1.71]	2.2%
Martinez-Gonzalez 2014b	Olive oil	Supplementation+Intake	Intake	Coronary heart disease	0.81	[0.53; 1.24]	0.96	[0.78; 1.18]	0.84	[0.53; 1.35]	1.0%
Martinez-Gonzalez 2014b	Olive oil	Supplementation+Intake	Intake	Stroke	0.69	[0.48; 0.98]	0.74	[0.60; 0.92]	0.93	[0.62; 1.41]	1.2%
Moazzen 2018	Folic acid	Supplementation	Supplementation	Colorectal cancer	1.07	[0.86; 1.43]	0.96	[0.76; 1.21]	1.11	[0.79; 1.57]	1.6%
Mocellin 2017	Vitamin B6	Supplementation	Intake	Cancer	1.03	[0.94; 1.13]	0.95	[0.90; 1.01]	1.08	[0.97; 1.21]	4.3%
Morze 2021	Mediterranean diet	Intake	Intake	Mortality	0.75	[0.17; 3.33]	0.87	[0.82; 0.92]	0.86	[0.19; 3.82]	0.1%
Morze 2021	Mediterranean diet	Intake	Intake	Breast cancer	0.41	[0.19; 0.87]	0.97	[0.94; 1.00]	0.42	[0.20; 0.91]	0.4%
Sayehmiri 2018	Selenium	Supplementation	Supplementation+Intake	Prostate cancer	0.90	[0.74; 1.09]	0.89	[0.64; 1.23]	1.01	[0.69; 1.48]	1.4%
Schwingshackl 2015	Mediterranean diet	Intake	Intake	Type 2 diabetes	0.70	[0.54; 0.91]	0.83	[0.74; 0.92]	0.84	[0.64; 1.12]	2.1%
Schwingshackl 2017	Olive oil	Intake	Intake	Type 2 diabetes	0.60	[0.43; 0.84]	0.87	[0.80; 0.94]	0.69	[0.49; 0.97]	1.6%
Stratton 2011	Vitamin C	Supplementation	Supplementation	Prostate cancer	1.10	[0.93; 1.29]	0.96	[0.86; 1.06]	1.15	[0.94; 1.39]	3.1%
Stratton 2011	β-Carotene	Supplementation	Supplementation	Prostate cancer	1.19	[0.87; 1.65]	0.70	[0.63; 0.78]	1.70	[1.21; 2.38]	1.7%
Trikalinos 2012	Vitamin E	Supplementation	Supplementation+Intake	Mortality	0.97	[0.91; 1.03]	0.85	[0.78; 0.93]	1.14	[1.02; 1.27]	4.3%
Vinceti 2018a	Selenium	Supplementation	Status	Cancer	0.99	[0.86; 1.14]	0.75	[0.59; 0.94]	1.32	[1.01; 1.73]	2.2%
Vinceti 2018a	Selenium	Supplementation	Intake	Mortality	0.81	[0.49; 1.32]	0.93	[0.83; 1.04]	0.87	[0.52; 1.45]	0.9%
Wolf 2017	Multivitamin	Supplementation	Supplementation	Preterm birth	1.09	[0.43; 2.77]	0.84	[0.69; 1.03]	1.30	[0.50; 3.36]	0.3%
Wolf 2017	Multivitamin	Supplementation	Supplementation	Stillbirth	2.43	[0.12; 50.05]	0.78	[0.59; 1.03]	3.12	[0.15; 64.44]	0.0%
Yang 2020	Calcium	Supplementation	Intake	Cardiovascular disease	0.91	[0.56; 1.46]	0.98	[0.90; 1.06]	0.93	[0.57; 1.51]	1.0%
Yao 2019	Vitamin D	Supplementation	Status	Fractures	1.06	[0.97; 1.15]	0.89	[0.83; 0.96]	1.19	[1.06; 1.33]	4.2%
Yu 2021	Folic acid	Supplementation	Supplementation+Intake	Pre-eclampsia	1.11	[0.75; 1.62]	0.85	[0.76; 0.94]	1.31	[0.88; 1.95]	1.3%
Zhang 2016	Selenium	Supplementation	Status	Cardiovascular disease	0.91	[0.74; 1.10]	0.87	[0.76; 0.99]	1.05	[0.82; 1.33]	2.5%
Zhang 2018	Vitamin D	Supplementation	Status	Gestational diabetes mellitus	0.72	[0.39; 1.31]	0.58	[0.44; 0.76]	1.24	[0.64; 2.40]	0.6%
Zhao 2014	Multivitamin/Minerals	Supplementation	Supplementation	Nuclear cataract	0.73	[0.59; 0.90]	0.73	[0.64; 0.82]	1.00	[0.78; 1.28]	2.5%
Zhao 2014	Multivitamin/Minerals	Supplementation	Supplementation	Cortical cataract	0.92	[0.69; 1.23]	0.81	[0.68; 0.94]	1.14	[0.82; 1.58]	1.7%
Zhao 2014	Multivitamin/Minerals	Supplementation	Supplementation	Posterior subcapsular cataract	1.81	[1.30; 2.54]	0.96	[0.72; 1.20]	1.89	[1.24; 2.87]	1.2%
Zhou 2017	Vitamin D	Supplementation	Status	Preterm birth	0.58	[0.30; 1.11]	0.88	[0.73; 1.05]	0.66	[0.33; 1.30]	0.5%
**Random effects model**									**1.10**	**[1.04; 1.15]**	**100.0%**
**Prediction interval**										**[0.86; 1.39]**	

Heterogeneity: $I^2 = 48\%$, $\tau^2 = 0.0138$, $p < 0.01$

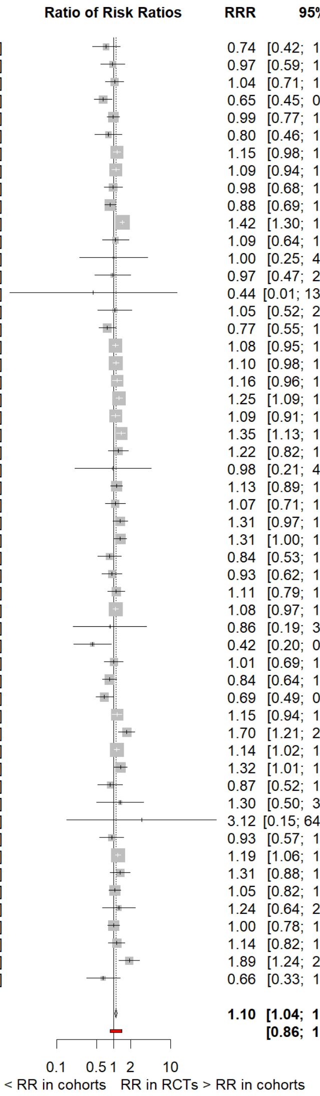

Supplementary Fig. S12 Forest plot, sensitivity analysis by direction of cohort study summary effect estimate (cohort studies with risk ratio [RR] <1)

Reference	Intervention/Exposure in SR	Type of intake (RCTs)	Type of exposure (cohorts)	Outcome in SR	RR (RCTs)	95%-CI	RR (cohorts)	95%-CI	RRR	95%-CI	Weight
Bolland 2015	Calcium	Supplementation	Supplementation	Fractures	0.89	[0.81; 0.96]	1.02	[0.93; 1.11]	0.87	[0.77; 0.99]	14.1%
Filippini 2020	Green tea	Supplementation	Intake	Prostate cancer	0.50	[0.18; 1.36]	1.09	[0.89; 1.32]	0.46	[0.16; 1.29]	1.5%
Fu 2021	Vitamin C	Supplementation	Supplementation	Lung cancer	1.30	[0.68; 2.48]	1.02	[0.85; 1.23]	1.27	[0.65; 2.50]	3.2%
Fu 2021	Vitamin C	Supplementation	Supplementation	Breast cancer	1.11	[0.87; 1.41]	1.02	[0.88; 1.18]	1.09	[0.82; 1.44]	9.3%
Kim 2018	Multivitamin/Minerals	Supplementation	Supplementation	Mortality	0.95	[0.83; 1.09]	1.00	[0.96; 1.05]	0.95	[0.82; 1.10]	13.5%
Lin 2020	Vitamin D	Supplementation	Supplementation	Nephrolithiasis	0.84	[0.65; 1.10]	1.24	[1.01; 1.51]	0.68	[0.49; 0.94]	8.1%
Lin 2020	Calcium	Supplementation	Supplementation	Nephrolithiasis	1.20	[0.65; 2.21]	1.16	[1.00; 1.35]	1.03	[0.55; 1.94]	3.5%
Stratton 2011	Multivitamin	Supplementation	Supplementation	Prostate cancer	0.98	[0.81; 1.20]	1.05	[0.94; 1.16]	0.93	[0.75; 1.17]	11.1%
Stratton 2011	Vitamin E	Supplementation	Supplementation	Prostate cancer	1.01	[0.90; 1.13]	1.09	[0.94; 1.27]	0.93	[0.77; 1.12]	12.1%
Vinceti 2018b	Selenium	Supplementation	Intake	Type 2 diabetes	1.11	[1.01; 1.22]	2.28	[1.31; 3.89]	0.49	[0.28; 0.85]	4.3%
Wien 2012	Folic acid	Supplementation	Supplementation	Cancer	1.07	[1.00; 1.14]	1.03	[0.90; 1.18]	1.04	[0.89; 1.21]	13.3%
Yang 2016	Folic acid	Supplementation	Supplementation+Intake	Gestational hypertension	0.62	[0.04; 0.94]	1.05	[1.00; 1.11]	0.59	[0.38; 0.91]	6.0%
**Random effects model**									**0.88**	**[0.77; 1.01]**	**100.0%**
**Prediction interval**										**[0.58; 1.33]**	

Heterogeneity: $I^2 = 45\%$, $\tau^2 = 0.0292$, $p = 0.04$

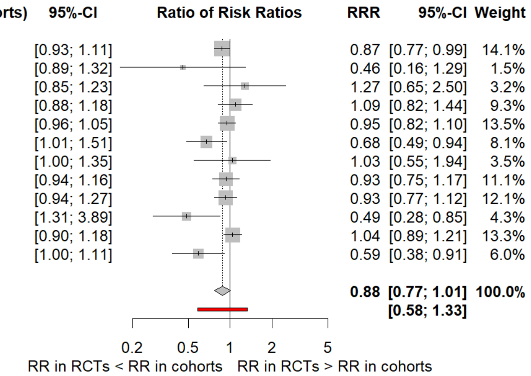

Supplementary Fig. S13 Forest plot, sensitivity analysis by direction of cohort study summary effect estimate (cohort studies with risk ratio [RR] ≥1)

Reference	Intervention/Exposure in SR	Type of intake (RCTs)	Type of exposure (cohorts)	Outcome in SR	RR (RCTs)	95%-CI	RR (cohorts)	95%-CI	RRR	95%-CI
**Vitamin D**										
Aguilar-Cordero 2020	Vitamin D	Supplementation	Status	Pre-eclampsia	0.66	[0.42; 1.05]	0.82	[0.60; 1.11]	0.80	[0.46; 1.40]
Chowdhury 2014b	Vitamin D	Supplementation	Status	All-cause mortality	0.98	[0.94; 1.03]	0.69	[0.65; 0.75]	1.42	[1.30; 1.55]
Chung 2011	Vitamin D	Supplementation	Status	Colorectal cancer	1.02	[0.60; 1.74]	0.94	[0.91; 0.97]	1.09	[0.64; 1.85]
Chung 2011	Vitamin D	Supplementation	Status	Breast cancer	0.99	[0.25; 3.99]	0.99	[0.97; 1.01]	1.00	[0.25; 4.00]
Lin 2020	Vitamin D	Supplementation	Supplementation	Nephrolithiasis	0.84	[0.65; 1.10]	1.24	[1.01; 1.51]	0.68	[0.49; 0.94]
Yao 2019	Vitamin D	Supplementation	Status	Fractures	1.06	[0.97; 1.15]	0.89	[0.83; 0.96]	1.19	[1.06; 1.33]
Zhang 2018	Vitamin D	Supplementation	Status	Gestational diabetes mellitus	0.72	[0.39; 1.31]	0.58	[0.44; 0.76]	1.24	[0.64; 2.40]
Zhou 2017	Vitamin D	Supplementation	Status	Preterm birth	0.58	[0.30; 1.11]	0.88	[0.73; 1.05]	0.66	[0.33; 1.30]
**Random effects model**									1.04	[0.85; 1.29]
**Prediction interval**										[0.58; 1.86]
Heterogeneity: $I^2 = 75\%$, $\tau^2 = 0.0445$, $p < 0.01$										
**Other Micronutrients**										
Aburto 2013	Low-sodium	Intake	Intake	All-cause mortality	0.70	[0.44; 1.14]	0.95	[0.71; 1.27]	0.74	[0.42; 1.29]
Aburto 2013	Low-sodium	Intake	Intake	Cardiovascular disease	0.84	[0.57; 1.23]	0.87	[0.64; 1.18]	0.97	[0.59; 1.58]
Bolland 2015	Calcium	Supplementation	Supplementation	Fractures	0.89	[0.81; 0.96]	1.02	[0.93; 1.11]	0.87	[0.77; 0.99]
Feng 2015	Folic acid	Supplementation	Supplementation	Congenital heart defects	0.58	[0.33; 1.02]	0.60	[0.38; 0.96]	0.97	[0.47; 2.01]
Fu 2021	Vitamin C	Supplementation	Supplementation	Lung cancer	1.30	[0.68; 2.48]	1.02	[0.85; 1.23]	1.27	[0.65; 2.50]
Fu 2021	Vitamin C	Supplementation	Supplementation	Breast cancer	1.11	[0.87; 1.41]	1.02	[0.88; 1.18]	1.09	[0.82; 1.44]
Fu 2021	Vitamin C	Supplementation	Supplementation	Colorectal cancer	0.84	[0.64; 1.10]	0.80	[0.42; 1.52]	1.05	[0.52; 2.11]
Jiang 2019	Vitamin E	Supplementation	Intake	Age-related cataract	0.97	[0.91; 1.03]	0.90	[0.80; 1.00]	1.08	[0.95; 1.22]
Jiang 2019	β-Carotene	Supplementation	Intake	Age-related cataract	0.99	[0.92; 1.07]	0.90	[0.83; 0.99]	1.10	[0.98; 1.24]
Jonker 2020	Folic acid	Supplementation	Supplementation	Low birthweight	0.96	[0.88; 1.04]	0.71	[0.60; 0.83]	1.35	[1.13; 1.62]
Jonker 2020	Folic acid	Supplementation	Supplementation+Intake	Small for gestational age	0.98	[0.92; 1.06]	0.80	[0.50; 1.11]	1.22	[0.82; 1.84]
Kim 2018	Multivitamin/Minerals	Supplementation	Supplementation	Mortality	0.95	[0.83; 1.09]	1.00	[0.96; 1.05]	0.95	[0.82; 1.10]
Kim 2018	Multivitamin/Minerals	Supplementation	Supplementation	Coronary heart disease	0.97	[0.79; 1.19]	0.86	[0.76; 0.97]	1.13	[0.89; 1.43]
Kim 2018	Multivitamin/Minerals	Supplementation	Supplementation	Stroke	1.05	[0.70; 1.56]	0.98	[0.91; 1.04]	1.07	[0.71; 1.61]
Kong 2014	Vitamins	Supplementation	Intake	Gastric cancer	1.11	[0.94; 1.31]	0.85	[0.66; 1.08]	1.31	[0.97; 1.76]
Lin 2019	Vitamin E	Supplementation	Intake	Bladder cancer	1.10	[0.86; 1.35]	0.84	[0.72; 0.96]	1.31	[1.00; 1.71]
Lin 2020	Calcium	Supplementation	Supplementation	Nephrolithiasis	1.20	[0.65; 2.21]	1.16	[1.00; 1.35]	1.03	[0.55; 1.94]
Moazzen 2018	Folic acid	Supplementation	Supplementation	Colorectal cancer	1.07	[0.86; 1.43]	0.96	[0.76; 1.21]	1.11	[0.79; 1.57]
Mocellin 2017	Vitamin B6	Supplementation	Intake	Cancer	1.03	[0.94; 1.13]	0.95	[0.90; 1.01]	1.08	[0.97; 1.21]
Sayehmiri 2018	Selenium	Supplementation	Supplementation+Intake	Prostate cancer	0.90	[0.74; 1.09]	0.89	[0.64; 1.23]	1.01	[0.69; 1.48]
Stratton 2011	Multivitamin	Supplementation	Supplementation	Prostate cancer	0.98	[0.81; 1.20]	1.05	[0.94; 1.16]	0.93	[0.75; 1.17]
Stratton 2011	Vitamin E	Supplementation	Supplementation	Prostate cancer	1.01	[0.90; 1.13]	1.09	[0.94; 1.27]	0.93	[0.77; 1.12]
Stratton 2011	Vitamin C	Supplementation	Supplementation	Prostate cancer	1.10	[0.93; 1.29]	0.96	[0.86; 1.06]	1.15	[0.94; 1.39]
Stratton 2011	β-Carotene	Supplementation	Supplementation	Prostate cancer	1.19	[0.87; 1.65]	0.70	[0.63; 0.78]	1.70	[1.21; 2.38]
Trikalinos 2012	Vitamin E	Supplementation	Supplementation+Intake	Mortality	0.97	[0.91; 1.03]	0.85	[0.78; 0.93]	1.14	[1.02; 1.27]
Vinceti 2018a	Selenium	Supplementation	Status	Cancer	0.99	[0.86; 1.14]	0.75	[0.59; 0.94]	1.32	[1.01; 1.73]
Vinceti 2018a	Selenium	Supplementation	Intake	Mortality	0.81	[0.49; 1.32]	0.93	[0.83; 1.04]	0.87	[0.52; 1.45]
Vinceti 2018b	Selenium	Supplementation	Intake	Type 2 diabetes	1.11	[1.01; 1.22]	2.28	[1.31; 3.89]	0.49	[0.28; 0.85]
Wien 2012	Folic acid	Supplementation	Supplementation	Cancer	1.07	[1.00; 1.14]	1.03	[0.90; 1.18]	1.04	[0.89; 1.21]
Wolf 2017	Multivitamin	Supplementation	Supplementation	Preterm birth	1.09	[0.43; 2.77]	0.84	[0.69; 1.03]	1.30	[0.50; 3.36]
Wolf 2017	Multivitamin	Supplementation	Supplementation	Stillbirth	2.43	[0.12; 50.05]	0.78	[0.59; 1.03]	3.12	[0.15; 64.44]
Yang 2016	Folic acid	Supplementation	Supplementation+Intake	Gestational hypertension	0.62	[0.04; 0.94]	1.05	[1.00; 1.11]	0.59	[0.38; 0.91]
Yang 2020	Calcium	Supplementation	Intake	Cardiovascular disease	0.91	[0.56; 1.46]	0.98	[0.90; 1.06]	0.93	[0.57; 1.51]
Yu 2021	Folic acid	Supplementation	Supplementation+Intake	Pre-eclampsia	1.11	[0.75; 1.62]	0.85	[0.76; 0.94]	1.31	[0.88; 1.95]
Zhang 2016	Selenium	Supplementation	Status	Cardiovascular disease	0.91	[0.74; 1.10]	0.87	[0.76; 0.99]	1.05	[0.82; 1.33]
Zhao 2014	Multivitamin/Minerals	Supplementation	Supplementation	Nuclear cataract	0.73	[0.59; 0.90]	0.73	[0.64; 0.82]	1.00	[0.78; 1.28]
Zhao 2014	Multivitamin/Minerals	Supplementation	Supplementation	Cortical cataract	0.92	[0.69; 1.23]	0.81	[0.68; 0.94]	1.14	[0.82; 1.58]
Zhao 2014	Multivitamin/Minerals	Supplementation	Supplementation	Posterior subcapsular cataract	1.81	[1.30; 2.54]	0.96	[0.72; 1.20]	1.89	[1.24; 2.87]
**Random effects model**									1.08	[1.01; 1.15]
**Prediction interval**										[0.83; 1.40]
Heterogeneity: $I^2 = 45\%$, $\tau^2 = 0.0159$, $p < 0.01$										
Test for subgroup differences: $\chi^2_1 = 0.09$, df = 1 ($p = 0.77$)										

Ratio of Risk Ratios

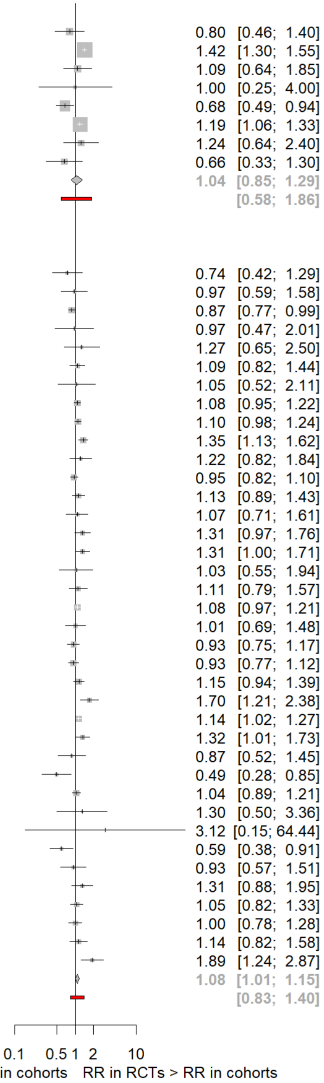

Supplementary Fig. S14 Forest plot, sensitivity analysis for interventions with micronutrients

Reference	Intervention/Exposure in SR	Type of intake (RCTs)	Type of exposure (cohorts)	Outcome in SR	RR (RCTs)	95%-CI	RR (cohorts)	95%-CI	RRR	95%-CI
**Colorectal cancer**										
Chung 2011	Vitamin D	Supplementation	Status	Colorectal cancer	1.02	[0.60; 1.74]	0.94	[0.91; 0.97]	1.09	[0.64; 1.85]
Fu 2021	Vitamin C	Supplementation	Supplementation	Colorectal cancer	0.84	[0.64; 1.10]	0.80	[0.42; 1.52]	1.05	[0.52; 2.11]
Johnston 2019	Low red meat	Intake	Intake	Colorectal cancer	1.02	[0.89; 1.18]	0.94	[0.85; 1.05]	1.09	[0.91; 1.29]
Moazzen 2018	Folic acid	Supplementation	Supplementation	Colorectal cancer	1.07	[0.86; 1.43]	0.96	[0.76; 1.21]	1.11	[0.79; 1.57]
**Random effects model**									1.09	[0.94; 1.26]
**Prediction interval**										[0.79; 1.50]
Heterogeneity: $I^2 = 0\%$, $\tau^2 = 0$, $p = 1.00$										
**Breast cancer**										
Chung 2011	Vitamin D	Supplementation	Status	Breast cancer	0.99	[0.25; 3.99]	0.99	[0.97; 1.01]	1.00	[0.25; 4.00]
Fu 2021	Vitamin C	Supplementation	Supplementation	Breast cancer	1.11	[0.87; 1.41]	1.02	[0.88; 1.18]	1.09	[0.82; 1.44]
Morze 2021	Mediterranean diet	Intake	Intake	Breast cancer	0.41	[0.19; 0.87]	0.97	[0.94; 1.00]	0.42	[0.20; 0.91]
**Random effects model**									0.80	[0.44; 1.46]
**Prediction interval**										[0.00; 407.35]
Heterogeneity: $I^2 = 62\%$, $\tau^2 = 0.1479$, $p = 0.07$										
**Prostate cancer**										
Filippini 2020	Green tea	Supplementation	Intake	Prostate cancer	0.50	[0.18; 1.36]	1.09	[0.89; 1.32]	0.46	[0.16; 1.29]
Sayehmiri 2018	Selenium	Supplementation	Supplementation+Intake	Prostate cancer	0.90	[0.74; 1.09]	0.89	[0.64; 1.23]	1.01	[0.69; 1.48]
Stratton 2011	Multivitamin	Supplementation	Supplementation	Prostate cancer	0.98	[0.81; 1.20]	1.05	[0.94; 1.16]	0.93	[0.75; 1.17]
Stratton 2011	Vitamin E	Supplementation	Supplementation	Prostate cancer	1.01	[0.90; 1.13]	1.09	[0.94; 1.27]	0.93	[0.77; 1.12]
Stratton 2011	Vitamin C	Supplementation	Supplementation	Prostate cancer	1.10	[0.93; 1.29]	0.96	[0.86; 1.06]	1.15	[0.94; 1.39]
Stratton 2011	β-Carotene	Supplementation	Supplementation	Prostate cancer	1.19	[0.87; 1.65]	0.70	[0.63; 0.78]	1.70	[1.21; 2.38]
**Random effects model**									1.05	[0.82; 1.35]
**Prediction interval**										[0.48; 2.31]
Heterogeneity: $I^2 = 64\%$, $\tau^2 = 0.0641$, $p = 0.02$										
**Any or other forms of cancer**										
Filippini 2020	Green tea	Supplementation	Intake	Endometrial cancer	0.33	[0.01; 8.15]	0.75	[0.43; 1.30]	0.44	[0.01; 13.14]
Fu 2021	Vitamin C	Supplementation	Supplementation	Lung cancer	1.30	[0.68; 2.48]	1.02	[0.85; 1.23]	1.27	[0.65; 2.50]
Kong 2014	Vitamins	Supplementation	Intake	Gastric cancer	1.11	[0.94; 1.31]	0.85	[0.66; 1.08]	1.31	[0.97; 1.76]
Lin 2019	Vitamin E	Supplementation	Intake	Bladder cancer	1.10	[0.86; 1.35]	0.84	[0.72; 0.96]	1.31	[1.00; 1.71]
Mocellin 2017	Vitamin B6	Supplementation	Intake	Cancer	1.03	[0.94; 1.13]	0.95	[0.90; 1.01]	1.08	[0.97; 1.21]
Morze 2021	Mediterranean diet	Intake	Intake	Mortality	0.75	[0.17; 3.33]	0.87	[0.82; 0.92]	0.86	[0.19; 3.82]
Vinceti 2018a	Selenium	Supplementation	Status	Cancer	0.99	[0.86; 1.14]	0.75	[0.59; 0.94]	1.32	[1.01; 1.73]
Vinceti 2018a	Selenium	Supplementation	Intake	Mortality	0.81	[0.49; 1.32]	0.93	[0.83; 1.04]	0.87	[0.52; 1.45]
Wien 2012	Folic acid	Supplementation	Supplementation	Cancer	1.07	[1.00; 1.14]	1.03	[0.90; 1.18]	1.04	[0.89; 1.21]
**Random effects model**									1.11	[1.03; 1.20]
**Prediction interval**										[1.02; 1.22]
Heterogeneity: $I^2 = 0\%$, $\tau^2 = 0$, $p = 0.59$										
Test for subgroup differences: $\chi^2_3 = 1.32$, df = 3 ($p = 0.72$)										

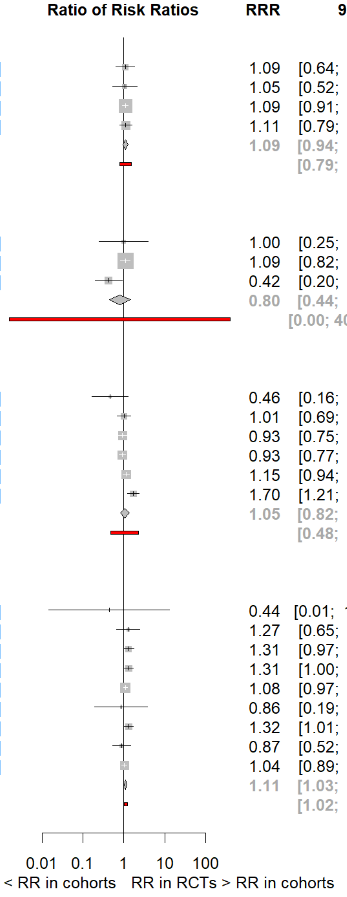

Supplementary Fig. S15 Forest plot, sensitivity analysis for cancer outcomes

Reference	Intervention/Exposure in SR	Type of intake (RCTs)	Type of exposure (cohorts)	Outcome in SR	RR (RCTs)	95%-CI	RR (cohorts)	95%-CI	RRR	95%-CI	Weight
Afshin 2014	Nuts	Intake	Intake	Coronary heart disease	0.79	[0.55; 1.14]	0.76	[0.69; 0.83]	1.04	[0.71; 1.51]	1.9%
Afshin 2014	Nuts	Intake	Intake	Stroke	0.62	[0.44; 0.87]	0.95	[0.83; 1.09]	0.65	[0.45; 0.94]	1.9%
Afshin 2014	Nuts	Intake	Intake	Type 2 diabetes	0.86	[0.68; 1.08]	0.87	[0.80; 0.95]	0.99	[0.77; 1.27]	2.8%
Bolland 2015	Calcium	Supplementation	Supplementation	Fractures	0.89	[0.81; 0.96]	1.02	[0.93; 1.11]	0.87	[0.77; 0.99]	3.8%
Chowdhury 2012	Omega-3 fatty acids	Supplementation	Intake	Stroke	0.98	[0.89; 1.08]	0.90	[0.80; 1.01]	1.09	[0.94; 1.27]	3.6%
Chung 2011	Vitamin D	Supplementation	Status	Colorectal cancer	1.02	[0.60; 1.74]	0.94	[0.91; 0.97]	1.09	[0.64; 1.85]	1.2%
Chung 2011	Vitamin D	Supplementation	Status	Breast cancer	0.99	[0.25; 3.99]	0.99	[0.97; 1.01]	1.00	[0.25; 4.00]	0.2%
Filippini 2020	Green tea	Supplementation	Intake	Prostate cancer	0.50	[0.18; 1.36]	1.09	[0.89; 1.32]	0.46	[0.16; 1.29]	0.4%
Filippini 2020	Green tea	Supplementation	Intake	Endometrial cancer	0.33	[0.01; 8.15]	0.75	[0.43; 1.30]	0.44	[0.01; 13.14]	0.0%
Fu 2021	Vitamin C	Supplementation	Supplementation	Lung cancer	1.30	[0.68; 2.48]	1.02	[0.85; 1.23]	1.27	[0.65; 2.50]	0.8%
Fu 2021	Vitamin C	Supplementation	Supplementation	Breast cancer	1.11	[0.87; 1.41]	1.02	[0.88; 1.18]	1.09	[0.82; 1.44]	2.5%
Fu 2021	Vitamin C	Supplementation	Supplementation	Colorectal cancer	0.84	[0.64; 1.10]	0.80	[0.42; 1.52]	1.05	[0.52; 2.11]	0.8%
Johnston 2019	Low red meat	Intake	Intake	Mortality	1.00	[0.84; 1.19]	0.86	[0.79; 0.94]	1.16	[0.96; 1.41]	3.2%
Johnston 2019	Low red meat	Intake	Intake	Type 2 diabetes	0.95	[0.89; 1.02]	0.76	[0.68; 0.86]	1.25	[1.09; 1.43]	3.7%
Johnston 2019	Low red meat	Intake	Intake	Colorectal cancer	1.02	[0.89; 1.18]	0.94	[0.85; 1.05]	1.09	[0.91; 1.29]	3.4%
Jonker 2020	Folic acid	Supplementation	Supplementation	Low birthweight	0.96	[0.88; 1.04]	0.71	[0.60; 0.83]	1.35	[1.13; 1.62]	3.3%
Jonker 2020	Folic acid	Supplementation	Supplementation+Intake	Small for gestational age	0.98	[0.92; 1.06]	0.80	[0.50; 1.11]	1.22	[0.82; 1.84]	1.7%
Kastorini 2011	Mediterranean diet	Intake	Intake	Metabolic syndrome	0.45	[0.24; 0.84]	0.46	[0.11; 1.88]	0.98	[0.21; 4.62]	0.2%
Kim 2018	Multivitamin/Minerals	Supplementation	Supplementation	Mortality	0.95	[0.83; 1.09]	1.00	[0.96; 1.05]	0.95	[0.82; 1.10]	3.7%
Kim 2018	Multivitamin/Minerals	Supplementation	Supplementation	Coronary heart disease	0.97	[0.79; 1.19]	0.86	[0.76; 0.97]	1.13	[0.89; 1.43]	2.9%
Kim 2018	Multivitamin/Minerals	Supplementation	Supplementation	Stroke	1.05	[0.70; 1.56]	0.98	[0.91; 1.04]	1.07	[0.71; 1.61]	1.7%
Kong 2014	Vitamins	Supplementation	Intake	Gastric cancer	1.11	[0.94; 1.31]	0.85	[0.66; 1.08]	1.31	[0.97; 1.76]	2.4%
Lin 2019	Vitamin E	Supplementation	Intake	Bladder cancer	1.10	[0.86; 1.35]	0.84	[0.72; 0.96]	1.31	[1.00; 1.71]	2.6%
Lin 2020	Vitamin D	Supplementation	Supplementation	Nephrolithiasis	0.84	[0.65; 1.10]	1.24	[1.01; 1.51]	0.68	[0.49; 0.94]	2.2%
Lin 2020	Calcium	Supplementation	Supplementation	Nephrolithiasis	1.20	[0.65; 2.21]	1.16	[1.00; 1.35]	1.03	[0.55; 1.94]	0.9%
Martinez-Gonzalez 2014b	Olive oil	Supplementation+Intake	Intake	Coronary heart disease	0.81	[0.53; 1.24]	0.96	[0.78; 1.18]	0.84	[0.53; 1.35]	1.4%
Martinez-Gonzalez 2014b	Olive oil	Supplementation+Intake	Intake	Stroke	0.69	[0.48; 0.98]	0.74	[0.60; 0.92]	0.93	[0.62; 1.41]	1.7%
Moazzen 2018	Folic acid	Supplementation	Supplementation	Colorectal cancer	1.07	[0.86; 1.43]	0.96	[0.76; 1.21]	1.11	[0.79; 1.57]	2.1%
Mocellin 2017	Vitamin B6	Supplementation	Intake	Cancer	1.03	[0.94; 1.13]	0.95	[0.90; 1.01]	1.08	[0.97; 1.21]	3.9%
Morze 2021	Mediterranean diet	Intake	Intake	Mortality	0.75	[0.17; 3.33]	0.87	[0.82; 0.92]	0.86	[0.19; 3.82]	0.2%
Morze 2021	Mediterranean diet	Intake	Intake	Breast cancer	0.41	[0.19; 0.87]	0.97	[0.94; 1.00]	0.42	[0.20; 0.91]	0.7%
Sayehmiri 2018	Selenium	Supplementation	Supplementation+Intake	Prostate cancer	0.90	[0.74; 1.09]	0.89	[0.64; 1.23]	1.01	[0.69; 1.48]	1.9%
Schwingshackl 2015	Mediterranean diet	Intake	Intake	Type 2 diabetes	0.70	[0.54; 0.91]	0.83	[0.74; 0.92]	0.84	[0.64; 1.12]	2.5%
Schwingshackl 2017	Olive oil	Intake	Intake	Type 2 diabetes	0.60	[0.43; 0.84]	0.87	[0.80; 0.94]	0.69	[0.49; 0.97]	2.1%
Stratton 2011	Multivitamin	Supplementation	Supplementation	Prostate cancer	0.98	[0.81; 1.20]	1.05	[0.94; 1.16]	0.93	[0.75; 1.17]	3.0%
Stratton 2011	Vitamin E	Supplementation	Supplementation	Prostate cancer	1.01	[0.90; 1.13]	1.09	[0.94; 1.27]	0.93	[0.77; 1.12]	3.3%
Stratton 2011	Vitamin C	Supplementation	Supplementation	Prostate cancer	1.10	[0.93; 1.29]	0.96	[0.86; 1.06]	1.15	[0.94; 1.39]	3.2%
Stratton 2011	β-Carotene	Supplementation	Supplementation	Prostate cancer	1.19	[0.87; 1.65]	0.70	[0.63; 0.78]	1.70	[1.21; 2.38]	2.1%
Trikalinos 2012	Vitamin E	Supplementation	Supplementation+Intake	Mortality	0.97	[0.91; 1.03]	0.85	[0.78; 0.93]	1.14	[1.02; 1.27]	3.9%
Vinceti 2018b	Selenium	Supplementation	Intake	Type 2 diabetes	1.11	[1.01; 1.22]	2.28	[1.31; 3.89]	0.49	[0.28; 0.85]	1.1%
Wien 2012	Folic acid	Supplementation	Supplementation	Cancer	1.07	[1.00; 1.14]	1.03	[0.90; 1.18]	1.04	[0.89; 1.21]	3.6%
Wolf 2017	Multivitamin	Supplementation	Supplementation	Stillbirth	2.43	[0.12; 50.05]	0.78	[0.59; 1.03]	3.12	[0.15; 64.44]	0.1%
Yang 2016	Folic acid	Supplementation	Supplementation+Intake	Gestational hypertension	0.62	[0.04; 0.94]	1.05	[1.00; 1.11]	0.59	[0.38; 0.91]	1.6%
Yang 2020	Calcium	Supplementation	Intake	Cardiovascular disease	0.91	[0.56; 1.46]	0.98	[0.90; 1.06]	0.93	[0.57; 1.51]	1.4%
Yu 2021	Folic acid	Supplementation	Supplementation+Intake	Pre-eclampsia	1.11	[0.75; 1.62]	0.85	[0.76; 0.94]	1.31	[0.88; 1.95]	1.8%
Zhao 2014	Multivitamin/Minerals	Supplementation	Supplementation	Nuclear cataract	0.73	[0.59; 0.90]	0.73	[0.64; 0.82]	1.00	[0.78; 1.28]	2.8%
Zhao 2014	Multivitamin/Minerals	Supplementation	Supplementation	Cortical cataract	0.92	[0.69; 1.23]	0.81	[0.68; 0.94]	1.14	[0.82; 1.58]	2.2%
Zhao 2014	Multivitamin/Minerals	Supplementation	Supplementation	Posterior subcapsular cataract	1.81	[1.30; 2.54]	0.96	[0.72; 1.20]	1.89	[1.24; 2.87]	1.6%
**Random effects model**									**1.03**	**[0.96; 1.11]**	**100.0%**
**Prediction interval**										**[0.73; 1.46]**	

Heterogeneity: $I^2 = 53\%$, $\tau^2 = 0.0283$, $p < 0.01$

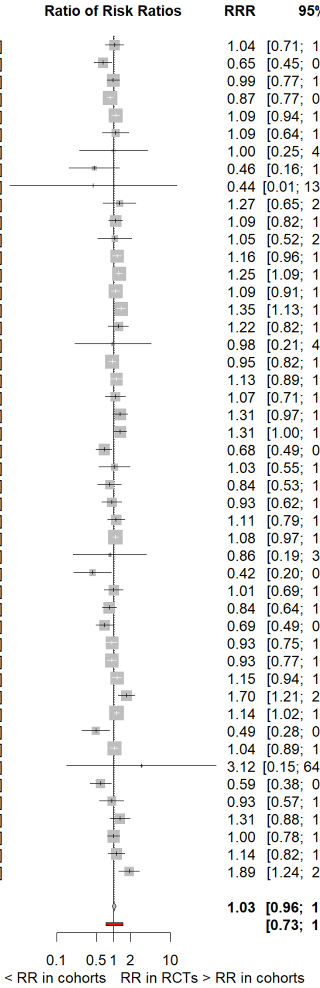

Supplementary Fig. S16 Forest plot, sensitivity analysis excluding BoE-pairs with highly similar PI/ECO questions and overlapping primary studies

Supplementary References

1. Schwingshackl L, Balduzzi S, Beyerbach J, et al. Evaluating agreement between bodies of evidence from randomised controlled trials and cohort studies in nutrition research: meta-epidemiological study. BMJ. 2021;374:n1864. doi:10.1136/bmj.n1864

2. Aburto NJ, Ziolkovska A, Hooper L, Elliott P, Cappuccio FP, Meerpohl JJ. Effect of lower sodium intake on health: systematic review and meta-analyses. BMJ. 2013;346:f1326. doi:10.1136/bmj.f1326

3. Chowdhury R, Kunutsor S, Vitezova A, et al. Vitamin D and risk of cause specific death: systematic review and meta-analysis of observational cohort and randomised intervention studies. BMJ. 2014;348:g1903. doi:10.1136/bmj.g1903

4. Feng Y, Wang S, Chen R, Tong X, Wu Z, Mo X. Maternal folic acid supplementation and the risk of congenital heart defects in offspring: a meta-analysis of epidemiological observational studies. Sci Rep. 2015;5:8506. doi:10.1038/srep08506

5. Jiang H, Yin Y, Wu CR, et al. Dietary vitamin and carotenoid intake and risk of age-related cataract. Am J Clin Nutr. 2019;109(1):43-54. doi:10.1093/ajcn/nqy270

6. Kastorini C-M, Milionis HJ, Esposito K, Giugliano D, Goudevenos JA, Panagiotakos DB. The effect of Mediterranean diet on metabolic syndrome and its components: a meta-analysis of 50 studies and 534,906 individuals. J Am Coll Cardiol. 2011;57(11):1299-313. doi:10.1016/j.jacc.2010.09.073

7. Vinceti M, Filippini T, Del Giovane C, et al. Selenium for preventing cancer. Cochrane Database Syst Rev. 2018;1:CD005195. doi:10.1002/14651858.CD005195.pub4

8. Wolf HT, Hegaard HK, Huusom LD, Pinborg AB. Multivitamin use and adverse birth outcomes in high-income countries: a systematic review and meta-analysis. Am J Obstet Gynecol. 2017;217(4):404.e1-.e30. doi:10.1016/j.ajog.2017.03.029

9. Zhang X, Liu C, Guo J, Song Y. Selenium status and cardiovascular diseases: meta-analysis of prospective observational studies and randomized controlled trials. Eur J Clin Nutr. 2016;70(2):162-9. doi:10.1038/ejcn.2015.78

10. Achon M, Ubeda N, Garcia-Gonzalez A, Partearroyo T, Varela-Moreiras G. Effects of Milk and Dairy Product Consumption on Pregnancy and Lactation Outcomes: A Systematic Review. Adv Nutr. 2019;10(suppl_2):S74-S87. doi:10.1093/advances/nmz009

11. Akesson A, Andersen LF, Kristjánsdóttir AG, et al. Health effects associated with foods characteristic of the Nordic diet: a systematic literature review. Food Nutr Res. 2013;57. doi:10.3402/fnr.v57i0.22790

12. Alexander DD, Weed DL, Chang ET, Miller PE, Mohamed MA, Elkayam L. A systematic review of multivitamin-multimineral use and cardiovascular disease and cancer incidence and total mortality. J Am Coll Nutr. 2013;32(5):339-54. doi:10.1080/07315724.2013.839909

13. Altun A, Brown H, Szoeke C, Goodwill AM. The Mediterranean dietary pattern and depression risk: A systematic review. Neurology, Psychiatry and Brain Research. 2019;33. doi:10.1016/j.npbr.2019.05.007

14. Aridi YS, Walker JL, Wright ORL. The Association between the Mediterranean Dietary Pattern and Cognitive Health: A Systematic Review. Nutrients. 2017;9(7). doi:10.3390/nu9070674

15. Aucoin M, Cooley K, Knee C, et al. Fish-Derived Omega-3 Fatty Acids and Prostate Cancer: A Systematic Review. Integr Cancer Ther. 2017;16(1):32-62. doi:10.1177/1534735416656052

16. Autier P, Boniol M, Pizot C, Mullie P. Vitamin D status and ill health: a systematic review. Lancet Diabetes Endocrinol. 2014;2(1):76-89. doi:10.1016/S2213-8587(13)70165-7

17. Behere RV, Deshmukh AS, Otiv S, Gupte MD, Yajnik CS. Maternal Vitamin B12 Status During Pregnancy and Its Association With Outcomes of Pregnancy and Health of the Offspring: A Systematic Review and Implications for Policy in India. Front Endocrinol (Lausanne). 2021;12:619176. doi:10.3389/fendo.2021.619176

18. Best KP, Gold M, Kennedy D, Martin J, Makrides M. Omega-3 long-chain PUFA intake during pregnancy and allergic disease outcomes in the offspring: a systematic review and meta-analysis of observational studies and randomized controlled trials. Am J Clin Nutr. 2016;103(1):128-43. doi:10.3945/ajcn.115.111104

19. Buyken AE, Goletzke J, Joslowski G, et al. Association between carbohydrate quality and inflammatory markers: systematic review of observational and interventional studies. Am J Clin Nutr. 2014;99(4):813-33. doi:10.3945/ajcn.113.074252

20. Cafe ACC, Lopes CAdO, Novais RLR, et al. Intake of Sugar-Sweetened Beverages, Milk and Its Association with Body Mass Index in Adolescence: A Systematic Review. Rev Paul Pediatr. 2018;36(1):91-9. doi:10.1590/1984-0462/;2018;36;1;00010

21. Cao Y, Winzenberg T, Nguo K, Lin J, Jones G, Ding C. Association between serum levels of 25-hydroxyvitamin D and osteoarthritis: a systematic review. Rheumatology (Oxford). 2013;52(7):1323-34. doi:10.1093/rheumatology/ket132

22. Castillo Lancellotti C, Tur Mari JA, Uauy Dagach R. [Effect of folate and related nutrients on cognitive function in older people; systematic review]. Nutr Hosp. 2012;27(1):90-102. doi:10.1590/S0212-16112012000100010

23. Castillo-L C, Tur JA, Uauy R. [Folate and breast cancer risk: a systematic review]. Rev Med Chil. 2012;140(2):251-60. doi:10.4067/S0034-98872012000200016

24. Christesen HT, Elvander C, Lamont RF, Jorgensen JS. The impact of vitamin D in pregnancy on extraskeletal health in children: a systematic review. Acta Obstet Gynecol Scand. 2012;91(12):1368-80. doi:10.1111/aogs.12006

25. Craddock JC, Neale EP, Probst YC, Peoples GE. Algal supplementation of vegetarian eating patterns improves plasma and serum docosahexaenoic acid concentrations and omega-3 indices: a systematic literature review. J Hum Nutr Diet. 2017;30(6):693-9. doi:10.1111/jhn.12474

26. Craig JV, Bunn DK, Hayhoe RP, Appleyard WO, Lenaghan EA, Welch AA. Relationship between the Mediterranean dietary pattern and musculoskeletal health in children, adolescents, and adults: systematic review and evidence map. Nutr Rev. 2017;75(10):830-57. doi:10.1093/nutrit/nux042

27. Cuesta-Triana F, Verdejo-Bravo C, Fernandez-Perez C, Martin-Sanchez FJ. Effect of Milk and Other Dairy Products on the Risk of Frailty, Sarcopenia, and Cognitive Performance Decline in the Elderly: A Systematic Review. Adv Nutr. 2019;10(suppl_2):S105-S19. doi:10.1093/advances/nmy105

28. Das A, Hsu MSH, Rangan A, Hirani V. Dietary or supplemental intake of antioxidants and the risk of mortality in older people: A systematic review. Nutr Diet. 2020;78(1):24-40. doi:10.1111/1747-0080.12611

29. de O Farias MC, Cavalcante TdLT, Assuncao ML, Bueno NB. Association between maternal or cord blood concentrations of 25-hydroxycholecalciferol or vitamin D supplementation during

pregnancy and the cytokines profile in the umbilical cord blood: Systematic literature review. J Steroid Biochem Mol Biol. 2020;203:105739. doi:10.1016/j.jsbmb.2020.105739

30. Dolinsky DH, Armstrong S, Mangarelli C, Kemper AR. The association between vitamin D and cardiometabolic risk factors in children: a systematic review. Clin Pediatr (Phila). 2013;52(3):210-23. doi:10.1177/0009922812470742

31. Eales J, Lenoir-Wijnkoop I, King S, et al. Is consuming yoghurt associated with weight management outcomes? Results from a systematic review. Int J Obes (Lond). 2016;40(5):731-46. doi:10.1038/ijo.2015.202

32. English LK, Ard JD, Bailey RL, et al. Evaluation of Dietary Patterns and All-Cause Mortality: A Systematic Review. JAMA Netw Open. 2021;4(8):e2122277. doi:10.1001/jamanetworkopen.2021.22277

33. Flower G, Fritz H, Balneaves LG, et al. Flax and Breast Cancer: A Systematic Review. Integr Cancer Ther. 2014;13(3):181-92. doi:10.1177/1534735413502076

34. Forsyth C, Kouvari M, D'Cunha NM, et al. The effects of the Mediterranean diet on rheumatoid arthritis prevention and treatment: a systematic review of human prospective studies. Rheumatol Int. 2018;38(5):737-47. doi:10.1007/s00296-017-3912-1

35. Fritz H, Kennedy D, Fergusson D, et al. Selenium and lung cancer: a systematic review and meta analysis. PLoS One. 2011;6(11):e26259. doi:10.1371/journal.pone.0026259

36. Fritz H, Seely D, Flower G, et al. Soy, red clover, and isoflavones and breast cancer: a systematic review. PLoS One. 2013;8(11):e81968. doi:10.1371/journal.pone.0081968

37. Galie S, Canudas S, Muralidharan J, Garcia-Gavilan J, Bullo M, Salas-Salvado J. Impact of Nutrition on Telomere Health: Systematic Review of Observational Cohort Studies and Randomized Clinical Trials. Adv Nutr. 2020;11(3):576-601. doi:10.1093/advances/nmz107

38. Gete DG, Waller M, Mishra GD. Effects of maternal diets on preterm birth and low birth weight: a systematic review. Br J Nutr. 2020;123(4):446-61. doi:10.1017/S0007114519002897

39. Goncalves C, Abreu S. Sodium and Potassium Intake and Cardiovascular Disease in Older People: A Systematic Review. Nutrients. 2020;12(11). doi:10.3390/nu12113447

40. Grosso G, Mistretta A, Frigiola A, et al. Mediterranean diet and cardiovascular risk factors: a systematic review. Crit Rev Food Sci Nutr. 2014;54(5):593-610. doi:10.1080/10408398.2011.596955

41. Guan VX, Mobasheri A, Probst YC. A systematic review of osteoarthritis prevention and management with dietary phytochemicals from foods. Maturitas. 2019;122:35-43. doi:10.1016/j.maturitas.2019.01.005

42. Guyenet SJ. Impact of Whole, Fresh Fruit Consumption on Energy Intake and Adiposity: A Systematic Review. Front Nutr. 2019;6:66. doi:10.3389/fnut.2019.00066

43. Habibi N, Bianco-Miotto T, Phoi YY, Jankovic-Karasoulos T, Roberts CT, Grieger JA. Maternal diet and offspring telomere length: a systematic review. Nutr Rev. 2021;79(2):148-59. doi:10.1093/nutrit/nuaa097

44. Hamidi M, Boucher BA, Cheung AM, Beyene J, Shah PS. Fruit and vegetable intake and bone health in women aged 45 years and over: a systematic review. Osteoporos Int. 2011;22(6):1681-93. doi:10.1007/s00198-010-1510-0

45. Hashemian M, Hekmatdoost A, Poustchi H, Mohammadi Nasrabadi F, Abnet CC, Malekzadeh R. Systematic review of zinc biomarkers and esophageal cancer risk. Middle East J Dig Dis. 2014;6(4):177-85.

46. Hauner D, Janni W, Rack B, Hauner H. The effect of overweight and nutrition on prognosis in breast cancer. Dtsch Arztebl Int. 2011;108(47):795-801. doi:10.3238/arztebl.2011.0795

47. Hebden L, O'Leary F, Rangan A, Singgih Lie E, Hirani V, Allman-Farinelli M. Fruit consumption and adiposity status in adults: A systematic review of current evidence. Crit Rev Food Sci Nutr. 2017;57(12):2526-40. doi:10.1080/10408398.2015.1012290

48. Hosseini B, Berthon BS, Saedisomeolia A, et al. Effects of fruit and vegetable consumption on inflammatory biomarkers and immune cell populations: a systematic literature review and meta-analysis. Am J Clin Nutr. 2018;108(1):136-55. doi:10.1093/ajcn/nqy082

49. Imhoff-Kunsch B, Briggs V, Goldenberg T, Ramakrishnan U. Effect of n-3 long-chain polyunsaturated fatty acid intake during pregnancy on maternal, infant, and child health outcomes: a systematic review. Paediatr Perinat Epidemiol. 2012;26 Suppl 1:91-107. doi:10.1111/j.1365-3016.2012.01292.x

50. Jamshidi F, Kelishadi R. A systematic review on the effects of maternal calcium supplementation on offspring's blood pressure. J Res Med Sci. 2015;20(10):994-9. doi:10.4103/1735-1995.172794

51. Kheirouri S, Alizadeh M. MIND diet and cognitive performance in older adults: a systematic review. Crit Rev Food Sci Nutr. 2021:1-19. doi:10.1080/10408398.2021.1925220

52. Kontogianni MD, Panagiotakos DB. Dietary patterns and stroke: a systematic review and re-meta-analysis. Maturitas. 2014;79(1):41-7. doi:10.1016/j.maturitas.2014.06.014

53. Lee J, Fu Z, Chung M, Jang D-J, Lee H-J. Role of milk and dairy intake in cognitive function in older adults: a systematic review and meta-analysis. Nutr J. 2018;17(1):82. doi:10.1186/s12937-018-0387-1

54. Li P, Fan C, Lu Y, Qi K. Effects of calcium supplementation on body weight: a meta-analysis. Am J Clin Nutr. 2016;104(5):1263-73. doi:10.3945/ajcn.116.136242

55. Limongi F, Siviero P, Bozanic A, Noale M, Veronese N, Maggi S. The Effect of Adherence to the Mediterranean Diet on Late-Life Cognitive Disorders: A Systematic Review. J Am Med Dir Assoc. 2020;21(10):1402-9. doi:10.1016/j.jamda.2020.08.020

56. Liska D, Kelley M, Mah E. 100% Fruit Juice and Dental Health: A Systematic Review of the Literature. Front Public Health. 2019;7:190. doi:10.3389/fpubh.2019.00190

57. Liu Y, He Y, Baorong H, Lingbo K. The anti-osteoporostic effects of Natto on Bone Mineral Density in Perimenopausal Women. Curr Med Chem. 2020. doi:10.2174/0929867327666200306123140

58. Lourida I, Soni M, Thompson-Coon J, et al. Mediterranean diet, cognitive function, and dementia: a systematic review. Epidemiology. 2013;24(4):479-89. doi:10.1097/EDE.0b013e3182944410

59. Luong R, Ribeiro RV, Cunningham J, Chen S, Hirani V. The short- and long-term effects of dietary patterns on cardiometabolic health in adults aged 65 years or older: a systematic review. Nutr Rev. 2021. doi:10.1093/nutrit/nuab032

60. Maki KC, Palacios OM, Koecher K, et al. The Relationship between Whole Grain Intake and Body Weight: Results of Meta-Analyses of Observational Studies and Randomized Controlled Trials. Nutrients. 2019;11(6). doi:10.3390/nu11061245

61. Martinez-Gonzalez MA, Gea A, Ruiz-Canela M. The Mediterranean Diet and Cardiovascular Health. Circ Res. 2019;124(5):779-98. doi:10.1161/CIRCRESAHA.118.313348

62. Masana MF, Koyanagi A, Haro JM, Tyrovolas S. n-3 Fatty acids, Mediterranean diet and cognitive function in normal aging: A systematic review. Exp Gerontol. 2017;91:39-50. doi:10.1016/j.exger.2017.02.008

63. McClure ST, Rebholz CM, Medabalimi S, et al. Dietary phosphorus intake and blood pressure in adults: a systematic review of randomized trials and prospective observational studies. Am J Clin Nutr. 2019;109(5):1264-72. doi:10.1093/ajcn/nqy343

64. Melo van Lent D, Leermakers ETM, Darweesh SKL, et al. The effects of lutein on respiratory health across the life course: A systematic review. Clin Nutr ESPEN. 2016;13:e1-e7. doi:10.1016/j.clnesp.2016.02.096

65. Mirone M, Giannetta E, Isidori AM. Selenium and reproductive function. A systematic review. J Endocrinol Invest. 2013;36(10 Suppl):28-36.

66. Mitri J, Muraru MD, Pittas AG. Vitamin D and type 2 diabetes: a systematic review. Eur J Clin Nutr. 2011;65(9):1005-15. doi:10.1038/ejcn.2011.118

67. Mutua AM, Mogire RM, Elliott AM, et al. Effects of vitamin D deficiency on neurobehavioural outcomes in children: a systematic review. Wellcome Open Res. 2020;5:28. doi:10.12688/wellcomeopenres.15730.1

68. Newberry SJ, Chung M, Shekelle PG, et al. Vitamin D and Calcium: A Systematic Review of Health Outcomes (Update). Evid Rep Technol Assess. 2014(217):1-929. doi:10.23970/AHRQEPCERTA217

69. Noel SE, Stoneham ACS, Olsen CM, Rhodes LE, Green AC. Consumption of omega-3 fatty acids and the risk of skin cancers: a systematic review and meta-analysis. Int J Cancer. 2014;135(1):149-56. doi:10.1002/ijc.28630

70. Palermo A, Tuccinardi D, D'Onofrio L, et al. Vitamin K and osteoporosis: Myth or reality? Metabolism. 2017;70:57-71. doi:10.1016/j.metabol.2017.01.032

71. Parizadeh SM, Rezayi M, Jafarzadeh-Esfehani R, et al. Association of vitamin D status with liver and kidney disease: A systematic review of clinical trials, and cross-sectional and cohort studies. Int J Vitam Nutr Res. 2021;91(1-2):175-87. doi:10.1024/0300-9831/a000540

72. Paula Bricarello L, Poltronieri F, Fernandes R, Retondario A, de Moraes Trindade EBS, de Vasconcelos FAG. Effects of the Dietary Approach to Stop Hypertension (DASH) diet on blood pressure, overweight and obesity in adolescents: A systematic review. Clin Nutr ESPEN. 2018;28:1-11. doi:10.1016/j.clnesp.2018.09.003

73. Perez V, Chang ET. Sodium-to-potassium ratio and blood pressure, hypertension, and related factors. Adv Nutr. 2014;5(6):712-41. doi:10.3945/an.114.006783

74. Petersson SD, Philippou E. Mediterranean Diet, Cognitive Function, and Dementia: A Systematic Review of the Evidence. Adv Nutr. 2016;7(5):889-904. doi:10.3945/an.116.012138

75. Pollakova D, Andreadi A, Pacifici F, Della-Morte D, Lauro D, Tubili C. The Impact of Vegan Diet in the Prevention and Treatment of Type 2 Diabetes: A Systematic Review. Nutrients. 2021;13(6). doi:10.3390/nu13062123

76. Reid AE, Chauhan BF, Rabbani R, et al. Early Exposure to Nonnutritive Sweeteners and Long-term Metabolic Health: A Systematic Review. Pediatrics. 2016;137(3):e20153603. doi:10.1542/peds.2015-3603

77. Ren X, Vilhjálmsdóttir BL, Rohde JF, et al. Systematic Literature Review and Meta-Analysis of the Relationship Between Polyunsaturated and Trans Fatty Acids During Pregnancy and Offspring Weight Development. Front Nutr. 2021;8:625596. doi:10.3389/fnut.2021.625596

78. Sanchez-Sanchez ML, Garcia-Vigara A, Hidalgo-Mora JJ, Garcia-Perez M-A, Tarin J, Cano A. Mediterranean diet and health: A systematic review of epidemiological studies and intervention trials. Maturitas. 2020;136:25-37. doi:10.1016/j.maturitas.2020.03.008

79. Schwab U, Lauritzen L, Tholstrup T, et al. Effect of the amount and type of dietary fat on cardiometabolic risk factors and risk of developing type 2 diabetes, cardiovascular diseases, and cancer: a systematic review. Food Nutr Res. 2014;58(no pagination). doi:10.3402/fnr.v58.25145

80. Shams-White MM, Chung M, Du M, et al. Dietary protein and bone health: a systematic review and meta-analysis from the National Osteoporosis Foundation. Am J Clin Nutr. 2017;105(6):1528-43. doi:10.3945/ajcn.116.145110

81. Shapiro MJ, Downs SM, Swartz HJ, et al. A Systematic Review Investigating the Relation Between Animal-Source Food Consumption and Stunting in Children Aged 6-60 Months in Low and Middle-Income Countries. Adv Nutr. 2019;10(5):827-47. doi:10.1093/advances/nmz018

82. Shen S-Y, Xiao W-Q, Lu J-H, et al. Early life vitamin D status and asthma and wheeze: a systematic review and meta-analysis. BMC Pulm Med. 2018;18(1):120. doi:10.1186/s12890-018-0679-4

83. Silano M, Agostoni C, Sanz Y, Guandalini S. Infant feeding and risk of developing celiac disease: a systematic review. BMJ Open. 2016;6(1):e009163. doi:10.1136/bmjopen-2015-009163

84. Simnadis TG, Tapsell LC, Beck EJ. Effect of sorghum consumption on health outcomes: a systematic review. Nutr Rev. 2016;74(11):690-707. doi:10.1093/nutrit/nuw036

85. Sparling TM, Henschke N, Nesbitt RC, Gabrysch S. The role of diet and nutritional supplementation in perinatal depression: a systematic review. Matern Child Nutr. 2017;13(1). doi:10.1111/mcn.12235

86. Tabatabaei-Malazy O, Nikfar S, Larijani B, Abdollahi M. Influence of ascorbic acid supplementation on type 2 diabetes mellitus in observational and randomized controlled trials; a systematic review with meta-analysis. J Pharm Pharm Sci. 2014;17(4):554-82. doi:10.18433/j3zg6r

87. Tada A, Miura H. The Relationship between Vitamin C and Periodontal Diseases: A Systematic Review. Int J Environ Res Public Health. 2019;16(14). doi:10.3390/ijerph16142472

88. Theodore LE, Kellow NJ, McNeil EA, Close EO, Coad EG, Cardoso BR. Nut Consumption for Cognitive Performance: A Systematic Review. Adv Nutr. 2021;12(3):777-92. doi:10.1093/advances/nmaa153

89. Tsirimiagkou C, Basdeki ED, Argyris A, et al. Current Data on Dietary Sodium, Arterial Structure and Function in Humans: A Systematic Review. Nutrients. 2019;12(1). doi:10.3390/nu12010005

90. Van Elswyk ME, Weatherford CA, McNeill SH. A Systematic Review of Renal Health in Healthy Individuals Associated with Protein Intake above the US Recommended Daily Allowance in Randomized Controlled Trials and Observational Studies. Adv Nutr. 2018;9(4):404-18. doi:10.1093/advances/nmy026

91. Venter C, Agostoni C, Arshad SH, et al. Dietary factors during pregnancy and atopic outcomes in childhood: A systematic review from the European Academy of Allergy and Clinical Immunology. Pediatr Allergy Immunol. 2020;31(8):889-912. doi:10.1111/pai.13303

92. Vishwanathan R, Chung M, Johnson EJ. A systematic review on zinc for the prevention and treatment of age-related macular degeneration. Invest Ophthalmol Vis Sci. 2013;54(6):3985-98. doi:10.1167/iovs.12-11552

93. Viswanathan M, Treiman KA, Kish-Doto J, Middleton JC, Coker-Schwimmer EJL, Nicholson WK. Folic Acid Supplementation for the Prevention of Neural Tube Defects: An Updated Evidence Report and Systematic Review for the US Preventive Services Task Force. JAMA. 2017;317(2):190-203. doi:10.1001/jama.2016.19193

94. Wojcicki JM, Heyman MB. Maternal omega-3 fatty acid supplementation and risk for perinatal maternal depression. J Matern Fetal Neonatal Med. 2011;24(5):680-6. doi:10.3109/14767058.2010.521873

95. Wong MYZ, Man REK, Fenwick EK, et al. Dietary intake and diabetic retinopathy: A systematic review. PLoS One. 2018;13(1):e0186582. doi:10.1371/journal.pone.0186582

96. Yagi A, Nouchi R, Butler L, Kawashima R. Lutein Has a Positive Impact on Brain Health in Healthy Older Adults: A Systematic Review of Randomized Controlled Trials and Cohort Studies. Nutrients. 2021;13(6). doi:10.3390/nu13061746

97. Ye EQ, Chacko SA, Chou EL, Kugizaki M, Liu S. Greater whole-grain intake is associated with lower risk of type 2 diabetes, cardiovascular disease, and weight gain. J Nutr. 2012;142(7):1304-13. doi:10.3945/jn.111.155325

98. Zhang Y, Xun P, Chen C, et al. Magnesium levels in relation to rates of preterm birth: a systematic review and meta-analysis of ecological, observational, and interventional studies. Nutr Rev. 2021;79(2):188-99. doi:10.1093/nutrit/nuaa028

99. Altobelli E, Angeletti PM, Verrotti A, Petrocelli R. The Impact of Human Milk on Necrotizing Enterocolitis: A Systematic Review and Meta-Analysis. Nutrients. 2020;12(5). doi:10.3390/nu12051322

100. Bougma K, Aboud FE, Harding KB, Marquis GS. Iodine and mental development of children 5 years old and under: a systematic review and meta-analysis. Nutrients. 2013;5(4):1384-416. doi:10.3390/nu5041384

101. Canudas S, Becerra-Tomas N, Hernandez-Alonso P, et al. Mediterranean Diet and Telomere Length: A Systematic Review and Meta-Analysis. Adv Nutr. 2020;11(6):1544-54. doi:10.1093/advances/nmaa079

102. Centeno Tablante E, Pachon H, Guetterman HM, Finkelstein JL. Fortification of wheat and maize flour with folic acid for population health outcomes. Cochrane Database Syst Rev. 2019;7:CD012150. doi:10.1002/14651858.CD012150.pub2

103. Christensen N, Sondergaard J, Fisker N, Christesen HT. Infant Respiratory Tract Infections or Wheeze and Maternal Vitamin D in Pregnancy: A Systematic Review. Pediatr Infect Dis J. 2017;36(4):384-91. doi:10.1097/INF.0000000000001452

104. Chung M, Ma J, Patel K, Berger S, Lau J, Lichtenstein AH. Fructose, high-fructose corn syrup, sucrose, and nonalcoholic fatty liver disease or indexes of liver health: a systematic review and meta-analysis. Am J Clin Nutr. 2014;100(3):833-49. doi:10.3945/ajcn.114.086314

105. Dai Y, Liu J. Omega-3 long-chain polyunsaturated fatty acid and sleep: a systematic review and meta-analysis of randomized controlled trials and longitudinal studies. Nutr Rev. 2020. doi:10.1093/nutrit/nuaa103

106. Dineva M, Fishpool H, Rayman MP, Mendis J, Bath SC. Systematic review and meta-analysis of the effects of iodine supplementation on thyroid function and child neurodevelopment in mildly-to-moderately iodine-deficient pregnant women. Am J Clin Nutr. 2020;112(2):389-412. doi:10.1093/ajcn/nqaa071

107. Dullemeijer C, Souverein OW, Doets EL, et al. Systematic review with dose-response meta-analyses between vitamin B-12 intake and European Micronutrient Recommendations Aligned's prioritized biomarkers of vitamin B-12 including randomized controlled trials and observational studies in adults and elderly persons. Am J Clin Nutr. 2013;97(2):390-402. doi:10.3945/ajcn.112.033951

108. Frantsve-Hawley J, Bader JD, Welsh JA, Wright JT. A systematic review of the association between consumption of sugar-containing beverages and excess weight gain among children under age 12. J Public Health Dent. 2017;77 Suppl 1:S43-S66. doi:10.1111/jphd.12222

109. Gray NA, Dhana A, Stein DJ, Khumalo NP. Zinc and atopic dermatitis: a systematic review and meta-analysis. J Eur Acad Dermatol Venereol. 2019;33(6):1042-50. doi:10.1111/jdv.15524

110. Griebler U, Bruckmuller MU, Kien C, et al. Health effects of cow's milk consumption in infants up to 3 years of age: a systematic review and meta-analysis. Public Health Nutr. 2016;19(2):293-307. doi:10.1017/S1368980015001354

111. Hajimohammadi M, Shab-Bidar S, Neyestani TR. Vitamin D and serum leptin: a systematic review and meta-analysis of observational studies and randomized controlled trials. Eur J Clin Nutr. 2017;71(10):1144-53. doi:10.1038/ejcn.2016.245

112. Hattangdi-Haridas SR, Lanham-New SA, Wong WHS, Ho MHK, Darling AL. Vitamin D Deficiency and Effects of Vitamin D Supplementation on Disease Severity in Patients with Atopic Dermatitis: A Systematic Review and Meta-Analysis in Adults and Children. Nutrients. 2019;11(8). doi:10.3390/nu11081854

113. Hou Q, Li Y, Li L, et al. The Metabolic Effects of Oats Intake in Patients with Type 2 Diabetes: A Systematic Review and Meta-Analysis. Nutrients. 2015;7(12):10369-87. doi:10.3390/nu7125536

114. Huang J, Zhang L, Tang J, et al. Human milk as a protective factor for bronchopulmonary dysplasia: a systematic review and meta-analysis. Arch Dis Child Fetal Neonatal Ed. 2019;104(2):F128-F36. doi:10.1136/archdischild-2017-314205

115. Kanellopoulou A, Riza E, Samoli E, Benetou V. Dietary Supplement Use after Cancer Diagnosis in Relation to Total Mortality, Cancer Mortality and Recurrence: A Systematic Review and Meta-Analysis. Nutr Cancer. 2021;73(1):16-30. doi:10.1080/01635581.2020.1734215

116. Kim MJ, Kim S-N, Lee YW, Choe YB, Ahn KJ. Vitamin D Status and Efficacy of Vitamin D Supplementation in Atopic Dermatitis: A Systematic Review and Meta-Analysis. Nutrients. 2016;8(12). doi:10.3390/nu8120789

117. Marti A, Echeverria R, Morell-Azanza L, Ojeda-Rodriguez A. [Telomeres and diet quality]. Nutr Hosp. 2017;34(5):1226-45. doi:10.20960/nh.1181

118. Mayr HL, Tierney AC, Thomas CJ, Ruiz-Canela M, Radcliffe J, Itsiopoulos C. Mediterranean-type diets and inflammatory markers in patients with coronary heart disease: a systematic review and meta-analysis. Nutr Res. 2018;50:10-24. doi:10.1016/j.nutres.2017.10.014

119. Miller J, Tonkin E, Damarell RA, et al. A Systematic Review and Meta-Analysis of Human Milk Feeding and Morbidity in Very Low Birth Weight Infants. Nutrients. 2018;10(6). doi:10.3390/nu10060707

120. Moridi I, Chen A, Tal O, Tal R. The Association between Vitamin D and Anti-Mullerian Hormone: A Systematic Review and Meta-Analysis. Nutrients. 2020;12(6). doi:10.3390/nu12061567

121. Naude CE, Visser ME, Nguyen KA, Durao S, Schoonees A. Effects of total fat intake on bodyweight in children. Cochrane Database Syst Rev. 2018;7:CD012960. doi:10.1002/14651858.CD012960.pub2

122. Ndanuko RN, Ibrahim R, Hapsari RA, Neale EP, Raubenheimer D, Charlton KE. Association between the Urinary Sodium to Potassium Ratio and Blood Pressure in Adults: A Systematic Review and Meta-Analysis. Adv Nutr. 2021;12(5):1751-67. doi:10.1093/advances/nmab036

123. Prego-Dominguez J, Hadrya F, Takkouche B. Polyunsaturated Fatty Acids and Chronic Pain: A Systematic Review and Meta-analysis. Pain Physician. 2016;19(8):521-35.

124. Rimmelzwaan LM, van Schoor NM, Lips P, Berendse HW, Eekhoff EMW. Systematic Review of the Relationship between Vitamin D and Parkinson's Disease. J Parkinsons Dis. 2016;6(1):29-37. doi:10.3233/JPD-150615

125. Ristic-Medic D, Dullemeijer C, Tepsic J, et al. Systematic review using meta-analyses to estimate dose-response relationships between iodine intake and biomarkers of iodine status in different population groups. Nutr Rev. 2014;72(3):143-61. doi:10.1111/nure.12092

126. Sangare L, van Eijk AM, Ter Kuile FO, Walson J, Stergachis A. The association between malaria and iron status or supplementation in pregnancy: a systematic review and meta-analysis. PLoS One. 2014;9(2):e87743. doi:10.1371/journal.pone.0087743

127. Te Morenga L, Montez JM. Health effects of saturated and trans-fatty acid intake in children and adolescents: Systematic review and meta-analysis. PLoS One. 2017;12(11):e0186672. doi:10.1371/journal.pone.0186672

128. Villamor-Martinez E, Pierro M, Cavallaro G, Mosca F, Kramer BW, Villamor E. Donor Human Milk Protects against Bronchopulmonary Dysplasia: A Systematic Review and Meta-Analysis. Nutrients. 2018;10(2). doi:10.3390/nu10020238

129. Westhoff E, Witjes JA, Fleshner NE, et al. Body Mass Index, Diet-Related Factors, and Bladder Cancer Prognosis: A Systematic Review and Meta-Analysis. Bladder Cancer. 2018;4(1):91-112. doi:10.3233/BLC-170147

130. Xing M-Y, Xu S-Z, Shen P. Effect of Low-fat Diet on Breast Cancer Survival: a Meta-analysis. Asian Pac J Cancer Prev. 2014;15(3):1141-4. doi:10.7314/apjcp.2014.15.3.1141

131. Aleksandrova K, Koelman L, Rodrigues CE. Dietary patterns and biomarkers of oxidative stress and inflammation: A systematic review of observational and intervention studies. Redox Biol. 2021;42:101869. doi:10.1016/j.redox.2021.101869

132. Arab A, Golpour-Hamedani S, Rafie N. The Association Between Vitamin D and Premenstrual Syndrome: A Systematic Review and Meta-Analysis of Current Literature. J Am Coll Nutr. 2019;38(7):648-56. doi:10.1080/07315724.2019.1566036

133. Cai C, Sivak A, Davenport MH. Effects of Prenatal Artificial Sweeteners Consumption on Birth Outcomes: A Systematic Review and Meta-Analysis. Public Health Nutr. 2021:1-26. doi:10.1017/S1368980021000173

134. Craddock JC, Neale EP, Peoples GE, Probst YC. Vegetarian-Based Dietary Patterns and their Relation with Inflammatory and Immune Biomarkers: A Systematic Review and Meta-Analysis. Adv Nutr. 2019;10(3):433-51. doi:10.1093/advances/nmy103

135. Grimes CA, Bolton KA, Booth AB, et al. The association between dietary sodium intake, adiposity and sugar-sweetened beverages in children and adults: a systematic review and meta-analysis. Br J Nutr. 2020:1-53. doi:10.1017/S0007114520004122

136. Fowler ME, Akinyemiju TF. Meta-analysis of the association between dietary inflammatory index (DII) and cancer outcomes. Int J Cancer. 2017;141(11):2215-27. doi:10.1002/ijc.30922

137. Jiang K, Tang K, Liu H, Xu H, Ye Z, Chen Z. Ascorbic Acid Supplements and Kidney Stones Incidence Among Men and Women: A systematic review and meta-analysis. Urol J. 2019;16(2):115-20. doi:10.22037/uj.v0i0.4275

138. Jin H, Leng Q, Li C. Dietary flavonoid for preventing colorectal neoplasms. Cochrane Database Syst Rev. 2012(8):CD009350. doi:10.1002/14651858.CD009350.pub2

139. Li Y, Lv M-R, Wei Y-J, et al. Dietary patterns and depression risk: A meta-analysis. Psychiatry Res. 2017;253:373-82. doi:10.1016/j.psychres.2017.04.020

140. Liu SL, Zhao YP, Dai MH, You L, Wen Z, Xu JW. Vitamin D status and the risk of pancreatic cancer: a meta-analysis. Chin Med J (Engl). 2013;126(17):3356-9. doi:10.3760/cma.j.issn.0366-6999.20122850

141. Mahmoud AM, Al-Alem U, Dabbous F, et al. Zinc Intake and Risk of Prostate Cancer: Case-Control Study and Meta-Analysis. PLoS One. 2016;11(11):e0165956. doi:10.1371/journal.pone.0165956

142. Orlando L, Savel KA, Madigan S, Colasanto M, Korczak DJ. Dietary patterns and internalizing symptoms in children and adolescents: A meta-analysis. Aust N Z J Psychiatry. 2021:48674211031486. doi:10.1177/00048674211031486

143. Pimpin L, Wu JHY, Haskelberg H, Del Gobbo L, Mozaffarian D. Is Butter Back? A Systematic Review and Meta-Analysis of Butter Consumption and Risk of Cardiovascular Disease, Diabetes, and Total Mortality. PLoS One. 2016;11(6):e0158118. doi:10.1371/journal.pone.0158118

144. Shams-White MM, Chung M, Fu Z, et al. Animal versus plant protein and adult bone health: A systematic review and meta-analysis from the National Osteoporosis Foundation. PLoS One. 2018;13(2):e0192459. doi:10.1371/journal.pone.0192459

145. Silk LN, Greene DA, Baker MK. The Effect of Calcium or Calcium and Vitamin D Supplementation on Bone Mineral Density in Healthy Males: A Systematic Review and Meta-Analysis. Int J Sport Nutr Exerc Metab. 2015;25(5):510-24. doi:10.1123/ijsnem.2014-0202

146. Thangaratinam S, Rogozińska E, Jolly K, et al. Interventions to reduce or prevent obesity in pregnant women: a systematic review. Health Technol Assess. 2012;16(31):iii-iv, 1. doi:10.3310/hta16310

147. Wang J, Liu N, Sun W, Chen D, Zhao J, Zhang W. Association between vitamin D deficiency and antepartum and postpartum depression: a systematic review and meta-analysis of longitudinal studies. Arch Gynecol Obstet. 2018;298(6):1045-59. doi:10.1007/s00404-018-4902-6

148. Warthon-Medina M, Moran VH, Stammers AL, et al. Zinc intake, status and indices of cognitive function in adults and children: a systematic review and meta-analysis. Eur J Clin Nutr. 2015;69(6):649-61. doi:10.1038/ejcn.2015.60

149. Yokoyama Y, Levin SM, Barnard ND. Association between plant-based diets and plasma lipids: a systematic review and meta-analysis. Nutr Rev. 2017;75(9):683-98. doi:10.1093/nutrit/nux030

150. Yokoyama Y, Nishimura K, Barnard ND, et al. Vegetarian diets and blood pressure: a meta-analysis. JAMA Intern Med. 2014;174(4):577-87. doi:10.1001/jamainternmed.2013.14547

151. Zhang H, Hardie L, Bawajeeh AO, Cade J. Meat Consumption, Cognitive Function and Disorders: A Systematic Review with Narrative Synthesis and Meta-Analysis. Nutrients. 2020;12(5). doi:10.3390/nu12051528

152. Aburto NJ, Hanson S, Gutierrez H, Hooper L, Elliott P, Cappuccio FP. Effect of increased potassium intake on cardiovascular risk factors and disease: systematic review and meta-analyses. BMJ. 2013;346:f1378. doi:10.1136/bmj.f1378

153. Brondani JE, Comim FV, Flores LM, Martini LA, Premaor MO. Fruit and vegetable intake and bones: A systematic review and meta-analysis. PLoS One. 2019;14(5):e0217223. doi:10.1371/journal.pone.0217223

154. Cao AL, Lai YW, Chen HG, Sheng LT, Pan A. [Research progress of relationship between vitamin K and type 2 diabetes]. Zhonghua Yu Fang Yi Xue Za Zhi. 2020;54(5):555-62. doi:10.3760/cma.j.cn112150-20190520-00407

155. Companys J, Pla-Paga L, Calderon-Perez L, et al. Fermented Dairy Products, Probiotic Supplementation, and Cardiometabolic Diseases: A Systematic Review and Meta-analysis. Adv Nutr. 2020;11(4):834-63. doi:10.1093/advances/nmaa030

156. Cowell OR, Mistry N, Deighton K, et al. Effects of a Mediterranean diet on blood pressure: a systematic review and meta-analysis of randomized controlled trials and observational studies. J Hypertens. 2021;39(4):729-39. doi:10.1097/HJH.0000000000002667

157. D'Elia L, Dinu M, Sofi F, et al. 100% Fruit juice intake and cardiovascular risk: a systematic review and meta-analysis of prospective and randomised controlled studies. Eur J Nutr. 2021;60(5):2449-67. doi:10.1007/s00394-020-02426-7

158. Glisic M, Kastrati N, Gonzalez-Jaramillo V, et al. Associations between Phytoestrogens, Glucose Homeostasis, and Risk of Diabetes in Women: A Systematic Review and Meta-Analysis. Adv Nutr. 2018;9(6):726-40. doi:10.1093/advances/nmy048

159. Groenendijk I, den Boeft L, van Loon LJC, de Groot L. High Versus low Dietary Protein Intake and Bone Health in Older Adults: a Systematic Review and Meta-Analysis. Comput Struct Biotechnol J. 2019;17:1101-12. doi:10.1016/j.csbj.2019.07.005

160. He C, Lin Z, Robb SW, Ezeamama AE. Serum Vitamin D Levels and Polycystic Ovary syndrome: A Systematic Review and Meta-Analysis. Nutrients. 2015;7(6):4555-77. doi:10.3390/nu7064555

161. Hooper L, Abdelhamid A, Bunn D, Brown T, Summerbell CD, Skeaff CM. Effects of total fat intake on body weight. Cochrane Database Syst Rev. 2015(8):CD011834. doi:10.1002/14651858.CD011834

162. Hooper L, Abdelhamid A, Moore HJ, Douthwaite W, Skeaff CM, Summerbell CD. Effect of reducing total fat intake on body weight: systematic review and meta-analysis of randomised controlled trials and cohort studies. BMJ. 2012;345:e7666. doi:10.1136/bmj.e7666

163. Iguacel I, Huybrechts I, Moreno LA, Michels N. Vegetarianism and veganism compared with mental health and cognitive outcomes: a systematic review and meta-analysis. Nutr Rev. 2021;79(4):361-81. doi:10.1093/nutrit/nuaa030

164. Lai JS, Hiles S, Bisquera A, Hure AJ, McEvoy M, Attia J. A systematic review and meta-analysis of dietary patterns and depression in community-dwelling adults. Am J Clin Nutr. 2014;99(1):181-97. doi:10.3945/ajcn.113.069880

165. Leermakers ET, Darweesh SK, Baena CP, et al. The effects of lutein on cardiometabolic health across the life course: a systematic review and meta-analysis. Am J Clin Nutr. 2016;103(2):481-94. doi:10.3945/ajcn.115.120931

166. Li M, Francis E, Hinkle SN, Ajjarapu AS, Zhang C. Preconception and Prenatal Nutrition and Neurodevelopmental Disorders: A Systematic Review and Meta-Analysis. Nutrients. 2019;11(7). doi:10.3390/nu11071628

167. Lv N, Xiao L, Ma J. Dietary pattern and asthma: a systematic review and meta-analysis. J Asthma Allergy. 2014;7(7):105-21. doi:10.2147/JAA.S49960

168. Malik VS, Pan A, Willett WC, Hu FB. Sugar-sweetened beverages and weight gain in children and adults: a systematic review and meta-analysis. Am J Clin Nutr. 2013;98(4):1084-102. doi:10.3945/ajcn.113.058362

169. Mesas AE, Leon-Muñoz LM, Rodriguez-Artalejo F, Lopez-Garcia E. The effect of coffee on blood pressure and cardiovascular disease in hypertensive individuals: a systematic review and meta-analysis. Am J Clin Nutr. 2011;94(4):1113-26. doi:10.3945/ajcn.111.016667

170. Netting MJ, Middleton PF, Makrides M. Does maternal diet during pregnancy and lactation affect outcomes in offspring? A systematic review of food-based approaches. Nutrition. 2014;30(11-12):1225-41. doi:10.1016/j.nut.2014.02.015

171. Ong AM, Kang K, Weiler HA, Morin SN. Fermented Milk Products and Bone Health in Postmenopausal Women: A Systematic Review of Randomized Controlled Trials, Prospective Cohorts, and Case-Control Studies. Adv Nutr. 2020;11(2):251-65. doi:10.1093/advances/nmz108

172. Raman G, Avendano EE, Chen S, et al. Dietary intakes of flavan-3-ols and cardiometabolic health: systematic review and meta-analysis of randomized trials and prospective cohort studies. Am J Clin Nutr. 2019;110(5):1067-78. doi:10.1093/ajcn/nqz178

173. Reynolds A, Mann J, Cummings J, Winter N, Mete E, Te Morenga L. Carbohydrate quality and human health: a series of systematic reviews and meta-analyses. Lancet. 2019;393(10170):434-45. doi:10.1016/S0140-6736(18)31809-9

174. Reynolds AN, Akerman AP, Mann J. Dietary fibre and whole grains in diabetes management: Systematic review and meta-analyses. PLoS Med. 2020;17(3):e1003053. doi:10.1371/journal.pmed.1003053

175. Rogers PJ, Hogenkamp PS, de Graaf C, et al. Does low-energy sweetener consumption affect energy intake and body weight? A systematic review, including meta-analyses, of the evidence from human and animal studies. Int J Obes (Lond). 2016;40(3):381-94. doi:10.1038/ijo.2015.177

176. Steffen M, Kuhle C, Hensrud D, Erwin PJ, Murad MH. The effect of coffee consumption on blood pressure and the development of hypertension: a systematic review and meta-analysis. J Hypertens. 2012;30(12):2245-54. doi:10.1097/HJH.0b013e3283588d73

177. Szajewska H, Shamir R, Chmielewska A, et al. Systematic review with meta-analysis: early infant feeding and coeliac disease--update 2015. Aliment Pharmacol Ther. 2015;41(11):1038-54. doi:10.1111/apt.13163

178. Tielemans SMAJ, Altorf-van der Kuil W, Engberink MF, et al. Intake of total protein, plant protein and animal protein in relation to blood pressure: a meta-analysis of observational and intervention studies. J Hum Hypertens. 2013;27(9):564-71. doi:10.1038/jhh.2013.16

179. Wang W, Li J, Zhang H, Wang X, Zhang X. Effects of vitamin E supplementation on the risk and progression of AD: a systematic review and meta-analysis. Nutr Neurosci. 2019;24(1):1-10. doi:10.1080/1028415X.2019.1585506

180. Wang Z, Zhu W, Xing Y, Jia J, Tang Y. B vitamins and prevention of cognitive decline and incident dementia: a systematic review and meta-analysis. Nutr Rev. 2021. doi:10.1093/nutrit/nuab057

181. World Health Organization, Aburto NJ, Abudou M, Candeias V, Wu T. Effect and safety of salt iodization to prevent iodine deficiency disorders: a systematic review with meta-analyses2014.

182. Xu M, Guo D, Gu H, Zhang L, Lv S. Selenium and Preeclampsia: a Systematic Review and Meta-analysis. Biol Trace Elem Res. 2016;171(2):283-92. doi:10.1007/s12011-015-0545-7

183. Yao N, Yan S, Guo Y, et al. The association between carotenoids and subjects with overweight or obesity: a systematic review and meta-analysis. Food Funct. 2021. doi:10.1039/d1fo00004g

184. Zhang D, Cheng C, Wang Y, et al. Effect of Vitamin D on Blood Pressure and Hypertension in the General Population: An Update Meta-Analysis of Cohort Studies and Randomized Controlled Trials. Prev Chronic Dis. 2020;17:E03. doi:10.5888/pcd17.190307

185. Zhang G-Q, Liu B, Li J, et al. Fish intake during pregnancy or infancy and allergic outcomes in children: A systematic review and meta-analysis. Pediatr Allergy Immunol. 2017;28(2):152-61. doi:10.1111/pai.12648

186. Zhao B, Deng H, Li B, et al. Association of magnesium consumption with type 2 diabetes and glucose metabolism: A systematic review and pooled study with trial sequential analysis. Diabetes Metab Res Rev. 2020;36(3):e3243. doi:10.1002/dmrr.3243

187. Zhu B, Zhu B, Xiao C, Zheng Z. Vitamin D deficiency is associated with the severity of COPD: a systematic review and meta-analysis. Int J Chron Obstruct Pulmon Dis. 2015;10:1907-16. doi:10.2147/COPD.S89763

188. Alsofyani AH, Alharbi AMM, Alanazi ABN, et al. Correlation between Vitamin D Deficiency and Depression. The Egyptian Journal of Hospital Medicine. 2017;69(8):3134-43. doi:10.12816/0042865

189. Balk EM, Adams GP, Langberg V, et al. Omega-3 Fatty Acids and Cardiovascular Disease: An Updated Systematic Review. Evid Rep Technol Assess. 2016(223):1-1252. doi:10.23970/AHRQEPCERTA223

190. Busby E, Bold J, Fellows L, Rostami K. Mood Disorders and Gluten: It's Not All in Your Mind! A Systematic Review with Meta-Analysis. Nutrients. 2018;10(11). doi:10.3390/nu10111708

191. Doets EL, van Wijngaarden JP, Szczecinska A, et al. Vitamin B12 intake and status and cognitive function in elderly people. Epidemiol Rev. 2013;35:2-21. doi:10.1093/epirev/mxs003

192. Fortmann SP, Burda BU, Senger CA, Lin JS, Whitlock EP. Vitamin and mineral supplements in the primary prevention of cardiovascular disease and cancer: An updated systematic evidence review for the U.S. Preventive Services Task Force. Ann Intern Med. 2013;159(12):824-34. doi:10.7326/0003-4819-159-12-201312170-00729

193. Golzarand M, Hollis BW, Mirmiran P, Wagner CL, Shab-Bidar S. Vitamin D supplementation and body fat mass: a systematic review and meta-analysis. Eur J Clin Nutr. 2018;72(10):1345-57. doi:10.1038/s41430-018-0132-z

194. Goodwill AM, Szoeke C. A Systematic Review and Meta-Analysis of The Effect of Low Vitamin D on Cognition. J Am Geriatr Soc. 2017;65(10):2161-8. doi:10.1111/jgs.15012

195. Huang M, Li J, Ha MA, Riccardi G, Liu S. A systematic review on the relations between pasta consumption and cardio-metabolic risk factors. Nutr Metab Cardiovasc Dis. 2017;27(11):939-48. doi:10.1016/j.numecd.2017.07.005

196. Hurst R, Hooper L, Norat T, et al. Selenium and prostate cancer: systematic review and meta-analysis. Am J Clin Nutr. 2012;96(1):111-22. doi:10.3945/ajcn.111.033373

197. Loughrey DG, Lavecchia S, Brennan S, Lawlor BA, Kelly ME. The Impact of the Mediterranean Diet on the Cognitive Functioning of Healthy Older Adults: A Systematic Review and Meta-Analysis. Adv Nutr. 2017;8(4):571-86. doi:10.3945/an.117.015495

198. Newberry SJ, Chung M, Anderson CAM, et al. Sodium and Potassium Intake: Effects on Chronic Disease Outcomes and Risks2018.

199. Newberry SJ, Chung M, Booth M, et al. Omega-3 Fatty Acids and Maternal and Child Health: An Updated Systematic Review. Evid Rep Technol Assess. 2016(224):1-826. doi:10.23970/AHRQEPCERTA224

200. Saidifard N, Tangestani H, Djafarian K, Shab-Bidar S. Serum Vitamin D Level and Carotid Intima-Media Thickness: A Systematic Review and Meta-Analysis of Observational Studies and Randomized Control Trials. Horm Metab Res. 2020;52(5):305-15. doi:10.1055/a-1153-0657

201. Toews I, Lohner S, Kullenberg de Gaudry D, Sommer H, Meerpohl JJ. Association between intake of non-sugar sweeteners and health outcomes: systematic review and meta-analyses of randomised and non-randomised controlled trials and observational studies. BMJ. 2019;364:k4718. doi:10.1136/bmj.k4718

202. Wallace TC, Frankenfeld CL. Dietary Protein Intake above the Current RDA and Bone Health: A Systematic Review and Meta-Analysis. J Am Coll Nutr. 2017;36(6):481-96. doi:10.1080/07315724.2017.1322924

203. Wu P-Y, Chen K-M, Tsai W-C. The Mediterranean Dietary Pattern and Inflammation in Older Adults: A Systematic Review and Meta-analysis. Adv Nutr. 2021;12(2):363-73. doi:10.1093/advances/nmaa116

204. Dennert G, Zwahlen M, Brinkman M, Vinceti M, Zeegers MPA, Horneber M. Selenium for preventing cancer. Cochrane Database Syst Rev. 2011(5):CD005195. doi:10.1002/14651858.CD005195.pub2

205. Schwingshackl L, Schwedhelm C, Galbete C, Hoffmann G. Adherence to Mediterranean Diet and Risk of Cancer: An Updated Systematic Review and Meta-Analysis. Nutrients. 2017;9(10). doi:10.3390/nu9101063

206. Vinceti M, Dennert G, Crespi CM, et al. Selenium for preventing cancer. Cochrane Database Syst Rev. 2014(3):CD005195. doi:10.1002/14651858.CD005195.pub3

207. Becerra-Tomas N, Blanco Mejia S, Viguiliouk E, et al. Mediterranean diet, cardiovascular disease and mortality in diabetes: A systematic review and meta-analysis of prospective cohort studies and randomized clinical trials. Crit Rev Food Sci Nutr. 2020;60(7):1207-27. doi:10.1080/10408398.2019.1565281

208. Bloomfield HE, Koeller E, Greer N, MacDonald R, Kane R, Wilt TJ. Effects on Health Outcomes of a Mediterranean Diet With No Restriction on Fat Intake: A Systematic Review and Meta-analysis. Ann Intern Med. 2016;165(7):491-500. doi:10.7326/M16-0361

209. Chung M, Tang AM, Fu Z, Wang DD, Newberry SJ. Calcium Intake and Cardiovascular Disease Risk: An Updated Systematic Review and Meta-analysis. Ann Intern Med. 2016;165(12):856-66. doi:10.7326/M16-1165

210. Hua X, Zhang J, Guo Y, et al. Effect of folic acid supplementation during pregnancy on gestational hypertension/preeclampsia: A systematic review and meta-analysis. Hypertens Pregnancy. 2016;35(4):447-60. doi:10.1080/10641955.2016.1183673

211. Hypponen E, Cavadino A, Williams D, et al. Vitamin D and pre-eclampsia: original data, systematic review and meta-analysis. Ann Nutr Metab. 2013;63(4):331-40. doi:10.1159/000358338

212. Kohler LN, Foote J, Kelley CP, et al. Selenium and Type 2 Diabetes: Systematic Review. Nutrients. 2018;10(12). doi:10.3390/nu10121924

213. Koloverou E, Esposito K, Giugliano D, Panagiotakos D. The effect of Mediterranean diet on the development of type 2 diabetes mellitus: a meta-analysis of 10 prospective studies and 136,846 participants. Metabolism. 2014;63(7):903-11. doi:10.1016/j.metabol.2014.04.010

214. Kuria A, Tian H, Li M, et al. Selenium status in the body and cardiovascular disease: a systematic review and meta-analysis. Crit Rev Food Sci Nutr. 2020:1-10. doi:10.1080/10408398.2020.1803200

215. Liu C, Liu C, Wang Q, Zhang Z. Supplementation of folic acid in pregnancy and the risk of preeclampsia and gestational hypertension: a meta-analysis. Arch Gynecol Obstet. 2018;298(4):697-704. doi:10.1007/s00404-018-4823-4

216. Martinez-Gonzalez MA, Bes-Rastrollo M. Dietary patterns, Mediterranean diet, and cardiovascular disease. Curr Opin Lipidol. 2014;25(1):20-6. doi:10.1097/MOL.0000000000000044

217. Shim SM, Yun YU, Kim YS. Folic acid alone or multivitamin containing folic acid intake during pregnancy and the risk of gestational hypertension and preeclampsia through meta-analyses. Obstet Gynecol Sci. 2016;59(2):110-5. doi:10.5468/ogs.2016.59.2.110

218. Aguilar-Cordero MJ, Lasserrot-Cuadrado A, Mur-Villar N, Leon-Rios XA, Rivero-Blanco T, Perez-Castillo IM. Vitamin D, preeclampsia and prematurity: A systematic review and meta-analysis of observational and interventional studies. Midwifery. 2020;87:102707. doi:10.1016/j.midw.2020.102707

219. Alexander DD, Miller PE, Van Elswyk ME, Kuratko CN, Bylsma LC. A Meta-Analysis of Randomized Controlled Trials and Prospective Cohort Studies of Eicosapentaenoic and Docosahexaenoic Long-Chain Omega-3 Fatty Acids and Coronary Heart Disease Risk. Mayo Clin Proc. 2017;92(1):15-29. doi:10.1016/j.mayocp.2016.10.018

220. Azad MB, Abou-Setta AM, Chauhan BF, et al. Nonnutritive sweeteners and cardiometabolic health: a systematic review and meta-analysis of randomized controlled trials and prospective cohort studies. CMAJ. 2017;189(28):E929-E39. doi:10.1503/cmaj.161390

221. Bolland MJ, Leung W, Tai V, et al. Calcium intake and risk of fracture: systematic review. BMJ. 2015;351:h4580. doi:10.1136/bmj.h4580

222. Grosso G, Marventano S, Yang J, et al. A comprehensive meta-analysis on evidence of Mediterranean diet and cardiovascular disease: Are individual components equal? Crit Rev Food Sci Nutr. 2017;57(15):3218-32. doi:10.1080/10408398.2015.1107021

223. Kim J, Choi J, Kwon SY, et al. Association of Multivitamin and Mineral Supplementation and Risk of Cardiovascular Disease: A Systematic Review and Meta-Analysis. Circ Cardiovasc Qual Outcomes. 2018;11(7):e004224. doi:10.1161/CIRCOUTCOMES.117.004224

224. Stratton J, Godwin M. The effect of supplemental vitamins and minerals on the development of prostate cancer: a systematic review and meta-analysis. Fam Pract. 2011;28(3):243-52. doi:10.1093/fampra/cmq115

225. Thorne-Lyman A, Fawzi WW. Vitamin D during pregnancy and maternal, neonatal and infant health outcomes: a systematic review and meta-analysis. Paediatr Perinat Epidemiol. 2012;26 Suppl 1:75-90. doi:10.1111/j.1365-3016.2012.01283.x

226. Wien TN, Pike E, Wisløff T, Staff A, Smeland S, Klemp M. Cancer risk with folic acid supplements: a systematic review and meta-analysis. BMJ Open. 2012;2(1):e000653. doi:10.1136/bmjopen-2011-000653

227. Yang C, Shi X, Xia H, et al. The Evidence and Controversy Between Dietary Calcium Intake and Calcium Supplementation and the Risk of Cardiovascular Disease: A Systematic Review and Meta-Analysis of Cohort Studies and Randomized Controlled Trials. J Am Coll Nutr. 2020;39(4):352-70. doi:10.1080/07315724.2019.1649219

228. Yao P, Bennett D, Mafham M, et al. Vitamin D and Calcium for the Prevention of Fracture: A Systematic Review and Meta-analysis. JAMA Netw Open. 2019;2(12):e1917789. doi:10.1001/jamanetworkopen.2019.17789

229. Yu Y, Sun X, Wang X, Feng X. The Association Between the Risk of Hypertensive Disorders of Pregnancy and Folic Acid: A Systematic Review and Meta-Analysis. J Pharm Pharm Sci. 2021;24:174-90. doi:10.18433/jpps31500

230. Zhang Y, Gong Y, Xue H, Xiong J, Cheng G. Vitamin D and gestational diabetes mellitus: a systematic review based on data free of Hawthorne effect. BJOG. 2018;125(7):784-93. doi:10.1111/1471-0528.15060

231. Amegah AK, Klevor MK, Wagner CL. Maternal vitamin D insufficiency and risk of adverse pregnancy and birth outcomes: A systematic review and meta-analysis of longitudinal studies. PLoS One. 2017;12(3):e0173605. doi:10.1371/journal.pone.0173605

232. Zhou S-S, Tao Y-H, Huang K, Zhu B-B, Tao F-B. Vitamin D and risk of preterm birth: Up-to-date meta-analysis of randomized controlled trials and observational studies. J Obstet Gynaecol Res. 2017;43(2):247-56. doi:10.1111/jog.13239

233. Morze J, Danielewicz A, Przybylowicz K, Zeng H, Hoffmann G, Schwingshackl L. An updated systematic review and meta-analysis on adherence to mediterranean diet and risk of cancer. Eur J Nutr. 2021;60(3):1561-86. doi:10.1007/s00394-020-02346-6

234. Chowdhury R, Warnakula S, Kunutsor S, et al. Association of dietary, circulating, and supplement fatty acids with coronary risk: a systematic review and meta-analysis. Ann Intern Med. 2014;160(6):398-406. doi:10.7326/M13-1788

235. Yang X, Chen H, Du Y, Wang S, Wang Z. Periconceptional folic acid fortification for the risk of gestational hypertension and pre-eclampsia: a meta-analysis of prospective studies. Matern Child Nutr. 2016;12(4):669-79. doi:10.1111/mcn.12209

236. Vinceti M, Filippini T, Rothman KJ. Selenium exposure and the risk of type 2 diabetes: a systematic review and meta-analysis. Eur J Epidemiol. 2018;33(9):789-810. doi:10.1007/s10654-018-0422-8

237. Schwingshackl L, Missbach B, Konig J, Hoffmann G. Adherence to a Mediterranean diet and risk of diabetes: a systematic review and meta-analysis. Public Health Nutr. 2015;18(7):1292-9. doi:10.1017/S1368980014001542

238. Salas-Salvado J, Bullo M, Estruch R, et al. Prevention of diabetes with Mediterranean diets: a subgroup analysis of a randomized trial. Ann Intern Med. 2014;160(1):1-10. doi:10.7326/M13-1725

239. Trikalinos TA, Moorthy D, Chung M, et al. Concordance of randomized and nonrandomized studies was unrelated to translational patterns of two nutrient-disease associations. J Clin Epidemiol. 2012;65(1):16-29. doi:10.1016/j.jclinepi.2011.07.006

240. Afshin A, Micha R, Khatibzadeh S, Mozaffarian D. Consumption of nuts and legumes and risk of incident ischemic heart disease, stroke, and diabetes: a systematic review and meta-analysis. Am J Clin Nutr. 2014;100(1):278-88. doi:10.3945/ajcn.113.076901

241. Chowdhury R, Stevens S, Gorman D, et al. Association between fish consumption, long chain omega 3 fatty acids, and risk of cerebrovascular disease: systematic review and meta-analysis. BMJ. 2012;345:e6698. doi:10.1136/bmj.e6698

242. Chung M, Lee J, Terasawa T, Lau J, Trikalinos TA. Vitamin D with or without calcium supplementation for prevention of cancer and fractures: an updated meta-analysis for the U.S. Preventive Services Task Force. Ann Intern Med. 2011;155(12):827-38. doi:10.7326/0003-4819-155-12-201112200-00005

243. Ding M, Huang T, Bergholdt HK, Nordestgaard BG, Ellervik C, Qi L. Dairy consumption, systolic blood pressure, and risk of hypertension: Mendelian randomization study. BMJ. 2017;356:j1000. doi:10.1136/bmj.j1000

244. Filippini T, Malavolti M, Borrelli F, et al. Green tea (Camellia sinensis) for the prevention of cancer. Cochrane Database Syst Rev. 2020;3:CD005004. doi:10.1002/14651858.CD005004.pub3

245. Fu Y, Xu F, Jiang L, et al. Circulating vitamin C concentration and risk of cancers: a Mendelian randomization study. BMC Med. 2021;19(1):171. doi:10.1186/s12916-021-02041-1

246. Gayer BA, Avendano EE, Edelson E, Nirmala N, Johnson EJ, Raman G. Effects of Intake of Apples, Pears, or Their Products on Cardiometabolic Risk Factors and Clinical Outcomes: A Systematic Review and Meta-Analysis. Curr Dev Nutr. 2019;3(10):nzz109. doi:10.1093/cdn/nzz109

247. Johnston BC, Zeraatkar D, Han MA, et al. Unprocessed Red Meat and Processed Meat Consumption: Dietary Guideline Recommendations From the Nutritional Recommendations (NutriRECS) Consortium. Ann Intern Med. 2019;171(10):756-64. doi:10.7326/M19-1621

248. Jonker H, Capelle N, Lanes A, Wen SW, Walker M, Corsi DJ. Maternal folic acid supplementation and infant birthweight in low- and middle-income countries: A systematic review. Matern Child Nutr. 2020;16(1):e12895. doi:10.1111/mcn.12895

249. Kong P, Cai Q, Geng Q, et al. Vitamin intake reduce the risk of gastric cancer: meta-analysis and systematic review of randomized and observational studies. PLoS One. 2014;9(12):e116060. doi:10.1371/journal.pone.0116060

250. Lin J-H, Chen S-J, Liu H, Yan Y, Zheng J-H. Vitamin E consumption and the risk of bladder cancer. Int J Vitam Nutr Res. 2019;89(3-4):168-75. doi:10.1024/0300-9831/a000553

251. Lin BB, Lin ME, Huang RH, Hong YK, Lin BL, He XJ. Dietary and lifestyle factors for primary prevention of nephrolithiasis: a systematic review and meta-analysis. BMC Nephrol. 2020;21(1):267. doi:10.1186/s12882-020-01925-3

252. Martinez-Gonzalez MA, Dominguez LJ, Delgado-Rodriguez M. Olive oil consumption and risk of CHD and/or stroke: a meta-analysis of case-control, cohort and intervention studies. Br J Nutr. 2014;112(2):248-59. doi:10.1017/S0007114514000713

253. Miller PE, Perez V. Low-calorie sweeteners and body weight and composition: a meta-analysis of randomized controlled trials and prospective cohort studies. Am J Clin Nutr. 2014;100(3):765-77. doi:10.3945/ajcn.113.082826

254. Moazzen S, Dolatkhah R, Tabrizi JS, et al. Folic acid intake and folate status and colorectal cancer risk: A systematic review and meta-analysis. Clin Nutr. 2018;37(6 Pt A):1926-34. doi:10.1016/j.clnu.2017.10.010

255. Mocellin S, Briarava M, Pilati P. Vitamin B6 and Cancer Risk: A Field Synopsis and Meta-Analysis. J Natl Cancer Inst. 2017;109(3):1-9. doi:10.1093/jnci/djw230

256. Picasso MC, Lo-Tayraco JA, Ramos-Villanueva JM, Pasupuleti V, Hernandez AV. Effect of vegetarian diets on the presentation of metabolic syndrome or its components: A systematic review and meta-analysis. Clin Nutr. 2019;38(3):1117-32. doi:10.1016/j.clnu.2018.05.021

257. Sayehmiri K, Azami M, Mohammadi Y, Soleymani A, Tardeh Z. The association between Selenium and Prostate Cancer: a Systematic Review and Meta-Analysis. Asian Pac J Cancer Prev. 2018;19(6):1431-7. doi:10.22034/APJCP.2018.19.6.1431

258. Schwingshackl L, Lampousi AM, Portillo MP, Romaguera D, Hoffmann G, Boeing H. Olive oil in the prevention and management of type 2 diabetes mellitus: a systematic review and meta-analysis of cohort studies and intervention trials. Nutr Diabetes. 2017;7(4):e262. doi:10.1038/nutd.2017.12

259. Setien-Suero E, Suarez-Pinilla M, Suarez-Pinilla P, Crespo-Facorro B, Ayesa-Arriola R. Homocysteine and cognition: A systematic review of 111 studies. Neurosci Biobehav Rev. 2016;69:280-98. doi:10.1016/j.neubiorev.2016.08.014

260. Te Morenga L, Mallard S, Mann J. Dietary sugars and body weight: systematic review and meta-analyses of randomised controlled trials and cohort studies. BMJ. 2013;346:e7492. doi:10.1136/bmj.e7492

261. Zhao L-Q, Li L-M, Zhu H, The Epidemiological Evidence-Based Eye Disease Study Research Group EY. The effect of multivitamin/mineral supplements on age-related cataracts: a systematic review and meta-analysis. Nutrients. 2014;6(3):931-49. doi:10.3390/nu6030931

262. Howard BV, Van Horn L, Hsia J, et al. Low-fat dietary pattern and risk of cardiovascular disease: the Women's Health Initiative Randomized Controlled Dietary Modification Trial. JAMA. 2006;295(6):655-66. doi:10.1001/jama.295.6.655

263. Thomson CA, Van Horn L, Caan BJ, et al. Cancer incidence and mortality during the intervention and postintervention periods of the Women's Health Initiative dietary modification trial. Cancer Epidemiol Biomarkers Prev. 2014;23(12):2924-35. doi:10.1158/1055-9965.EPI-14-0922

264. Tinker LF, Bonds DE, Margolis KL, et al. Low-fat dietary pattern and risk of treated diabetes mellitus in postmenopausal women: the Women's Health Initiative randomized controlled dietary modification trial. Arch Intern Med. 2008;168(14):1500-11. doi:10.1001/archinte.168.14.1500

265. Gaziano JM, Glynn RJ, Christen WG, et al. Vitamins E and C in the prevention of prostate and total cancer in men: the Physicians' Health Study II randomized controlled trial. JAMA. 2009;301(1):52-62. doi:10.1001/jama.2008.862

266. Achkar M, Dodds L, Giguere Y, et al. Vitamin D status in early pregnancy and risk of preeclampsia. Am J Obstet Gynecol. 2015;212(4):511 e1-7. doi:10.1016/j.ajog.2014.11.009

267. Arisoy R, Bostanci E, Erdogdu E, Polat M, Kaya E, Tugrul S. Association between maternal serum 25-hydroxyvitamin D level and pre-eclampsia. J Matern Fetal Neonatal Med. 2016;29(12):1941-4. doi:10.3109/14767058.2015.1069269

268. Bener A, Al-Hamaq AO, Saleh NM. Association between vitamin D insufficiency and adverse pregnancy outcome: global comparisons. Int J Womens Health. 2013;5:523-31. doi:10.2147/IJWH.S51403

269. Burris HH, Rifas-Shiman SL, Huh SY, et al. Vitamin D status and hypertensive disorders in pregnancy. Ann Epidemiol. 2014;24(5):399-403 e1. doi:10.1016/j.annepidem.2014.02.001

270. Gidlöf S, Silva AT, Gustafsson S, Lindqvist PG. Vitamin D and the risk of preeclampsia – a nested case–control study. Acta Obstetricia et Gynecologica Scandinavica. 2015;94(8):904-8. doi:https://doi.org/10.1111/aogs.12658

271. Schneuer FJ, Roberts CL, Guilbert C, et al. Effects of maternal serum 25-hydroxyvitamin D concentrations in the first trimester on subsequent pregnancy outcomes in an Australian population. Am J Clin Nutr. 2014;99(2):287-95. doi:10.3945/ajcn.113.065672

272. van Weert B, van den Berg D, Hrudey EJ, Oostvogels A, de Miranda E, Vrijkotte TGM. Is first trimester vitamin D status in nulliparous women associated with pregnancy related hypertensive disorders? Midwifery. 2016;34:117-22. doi:10.1016/j.midw.2015.12.007

273. Trivedi DP, Doll R, Khaw KT. Effect of four monthly oral vitamin D3 (cholecalciferol) supplementation on fractures and mortality in men and women living in the community: randomised double blind controlled trial. BMJ. 2003;326(7387):469. doi:10.1136/bmj.326.7387.469

274. Czeizel AE. Periconceptional folic acid containing multivitamin supplementation. Eur J Obstet Gynecol Reprod Biol. 1998;78(2):151-61. doi:10.1016/s0301-2115(98)00061-x

275. Christian P, Khatry SK, Katz J, et al. Effects of alternative maternal micronutrient supplements on low birth weight in rural Nepal: double blind randomised community trial. BMJ. 2003;326(7389):571. doi:10.1136/bmj.326.7389.571

276. Balarajan Y, Subramanian SV, Fawzi WW. Maternal Iron and Folic Acid Supplementation Is Associated with Lower Risk of Low Birth Weight in India. J Nutr. 2013;143(8):1309-15. doi:10.3945/jn.112.172015

277. Chaudhary AK, Chaudhary A, Tiwari SC, Dwivedi R. Can community-based, low-cost antenatal care in the third trimester of pregnancy reduce the incidence of low birth weight newborns? J Obstet Gynaecol India. 2012;62(3):286-90. doi:10.1007/s13224-012-0217-x

278. Joseph N, Subba SH, Naik VA, et al. Incidence, Correlates and Outcomes of Low Birth Weight – A One Year Longitudinal Study. Indian Journal of Public Health Research and Development. 2011;2:132-7.

279. Nisar YB, Dibley MJ. Antenatal iron–folic acid supplementation reduces risk of low birthweight in Pakistan: secondary analysis of Demographic and Health Survey 2006–2007. Matern Child Nutr. 2016;12(1):85-98. doi:10.1111/mcn.12156

280. Wang S, Ge X, Zhu B, et al. Maternal Continuing Folic Acid Supplementation after the First Trimester of Pregnancy Increased the Risk of Large-for-Gestational-Age Birth: A Population-Based Birth Cohort Study. Nutrients. 2016;8(8). doi:10.3390/nu8080493

281. Zheng J-S, Guan Y, Zhao Y, et al. Pre-conceptional intake of folic acid supplements is inversely associated with risk of preterm birth and small-for-gestational-age birth: a prospective cohort study. Br J Nutr. 2016;115(3):509-16. doi:10.1017/S0007114515004663

282. Meyer F, Galan P, Douville P, et al. Antioxidant vitamin and mineral supplementation and prostate cancer prevention in the SU.VI.MAX trial. Int J Cancer. 2005;116(2):182-6. doi:10.1002/ijc.21058

283. Heinonen OP, Albanes D, Virtamo J, et al. Prostate cancer and supplementation with alpha-tocopherol and beta-carotene: incidence and mortality in a controlled trial. J Natl Cancer Inst. 1998;90(6):440-6. doi:10.1093/jnci/90.6.440

284. Álvarez-Silvares E, Vilouta-Romero M, Borrajo-Hernández E, Morales-Serrano ML, Alves-Pérez MT. [Maternal serum 25-hydroxy vitamin D levels in the first trimester and adverse gestational outcomes]. Ginecol Obstet Mex. 2016;84(3):150-63.

285. Burris HH R-SS, Camargo Jr CA, Litonjua AA, Huh SY, Rich-Edwards JW, Gillman, MW. Vitamin D deficiency in pregnancy is associated with gestational diabetes. Obesity (Silver Spring, Md.). 2011;19:S208.

286. Burris HH, Rifas-Shiman SL, Kleinman K, et al. Vitamin D deficiency in pregnancy and gestational diabetes mellitus. Am J Obstet Gynecol. 2012;207(3):182.e1-8. doi:10.1016/j.ajog.2012.05.022

287. Davies-Tuck M, Yim C, Hodges R, Wallace E. Vitamin d testing in pregnancy: Results from a routine screening program. J Paediatr Child Health. 2013;49:56.

288. Davies-Tuck M, Yim C, Knight M, Hodges R, Doery JC, Wallace E. Vitamin D testing in pregnancy: Does one size fit all? Aust N Z J Obstet Gynaecol. 2015;55(2):149-55. doi:10.1111/ajo.12278

289. Fernandez-Alonso A, Ferrando-Marco P, Dionis-Sanchez E, Gonzalez-Salmeron M, Fernandez-Soriano M, Fiol-Ruiz G. Serum 25-hydroxy-vitamin d levels during the first trimester of pregnancy and perinatal outcomes. J Matern Fetal Neonatal Med. 2010;23:560.

290. Hu X. The correlation study on Vitamin D and gestational diabetes mellitus. Chin J Med Drug. 2015;9(4):45-6.

291. Soytac Inancli I, Yayci E, Atacag T, Uncu M. Is maternal Vitamin D associated with gestational diabetes mellitus in pregnant women in Cyprus? Clin Exp Obstet Gynecol. 2016;43(6):840-3.

292. Kramer CK, Swaminathan B, Hanley AJ, et al. Vitamin D and parathyroid hormone status in pregnancy: effect on insulin sensitivity, β-cell function, and gestational diabetes mellitus. J Clin Endocrinol Metab. 2014;99(12):4506-13. doi:10.1210/jc.2014-2341

293. Loy SL, Lek N, Yap F, et al. Association of Maternal Vitamin D Status with Glucose Tolerance and Caesarean Section in a Multi-Ethnic Asian Cohort: The Growing Up in Singapore Towards Healthy Outcomes Study. PLoS One. 2015;10(11):e0142239. doi:10.1371/journal.pone.0142239

294. Park S, Yoon HK, Ryu HM, et al. Maternal vitamin D deficiency in early pregnancy is not associated with gestational diabetes mellitus development or pregnancy outcomes in Korean pregnant women in a prospective study. J Nutr Sci Vitaminol (Tokyo). 2014;60(4):269-75. doi:10.3177/jnsv.60.269

295. Song H, Lu F, Lei H. Research of the correlation between vitamin D levels and gestational diabetes mellitus. China Medical Herald. 2015;12(33):121-4.

296. Tomedi LE, Simhan HN, Bodnar LM. Early-pregnancy maternal vitamin D status and maternal hyperglycaemia. Diabet Med. 2013;30(9):1033-9. doi:10.1111/dme.12229

297. Zhou J, Su L, Liu M, et al. Associations between 25-hydroxyvitamin D levels and pregnancy outcomes: a prospective observational study in southern China. Eur J Clin Nutr. 2014;68(8):925-30. doi:10.1038/ejcn.2014.99

298. Zhu Z. The diagnostic value of 25OHD in gestational diabetes mellitus, evaluated according to receiver operating characteristic curve. J Southeast Univ (Med Sci Edi). 2015;34(6):1009-11.

299. Arnold DL, Enquobahrie DA, Qiu C, et al. Early pregnancy maternal vitamin D concentrations and risk of gestational diabetes mellitus. Paediatr Perinat Epidemiol. 2015;29(3):200-10. doi:10.1111/ppe.12182

300. Baker AM, Haeri S, Camargo CA, Jr., Stuebe AM, Boggess KA. First-trimester maternal vitamin D status and risk for gestational diabetes (GDM) a nested case-control study. Diabetes Metab Res Rev. 2012;28(2):164-8. doi:10.1002/dmrr.1282

301. Dodds L, Giguere Y, Woolcott C, Armson BA, Forest J-C, Weiler H. Vitamin D Status in Early Pregnancy and Development of Gestational Diabetes. Canadian Journal of Diabetes. 2013;37:S80-S1. doi:10.1016/j.jcjd.2013.08.247

302. Dodds L, Woolcott CG, Weiler H, et al. Vitamin D Status and Gestational Diabetes: Effect of Smoking Status during Pregnancy. Paediatr Perinat Epidemiol. 2016;30(3):229-37. doi:10.1111/ppe.12278

303. Giguere Y, Dodds L, Woolcott C, Armson A, Forest J, Weiler H. Is vitamin D level in early pregnancy associated with development of gestational diabetes? Clinical Chemistry and Laboratory Medicine. 2014;52:S1552.

304. Li L, Jiang H, Liu J, Fu L, Ma Y, Sun Z. A clinical study on relationship between Vitamin D and gestational diabetes mellitus. Journal of Chinese Physician. 2016;18(3):434-5.

305. Makgoba M, Nelson SM, Savvidou M, Messow CM, Nicolaides K, Sattar N. First-trimester circulating 25-hydroxyvitamin D levels and development of gestational diabetes mellitus. Diabetes Care. 2011;34(5):1091-3. doi:10.2337/dc10-2264

306. Zhang C, Qiu C, Hu FB, et al. Maternal plasma 25-hydroxyvitamin D concentrations and the risk for gestational diabetes mellitus. PLoS One. 2008;3(11):e3753. doi:10.1371/journal.pone.0003753

307. Hossain N, Kanani FH, Ramzan S, et al. Obstetric and neonatal outcomes of maternal vitamin D supplementation: results of an open-label, randomized controlled trial of antenatal vitamin D supplementation in Pakistani women. J Clin Endocrinol Metab. 2014;99(7):2448-55. doi:10.1210/jc.2013-3491

308. Mojibian M, Soheilykhah S, Fallah Zadeh MA, Jannati Moghadam M. The effects of vitamin D supplementation on maternal and neonatal outcome: A randomized clinical trial. Iran J Reprod Med. 2015;13(11):687-96.

309. Sablok A, Batra A, Thariani K, et al. Supplementation of vitamin D in pregnancy and its correlation with feto-maternal outcome. Clin Endocrinol (Oxf). 2015;83(4):536-41. doi:10.1111/cen.12751

310. Yap C, Cheung NW, Gunton JE, et al. Vitamin D supplementation and the effects on glucose metabolism during pregnancy: a randomized controlled trial. Diabetes Care. 2014;37(7):1837-44. doi:10.2337/dc14-0155

311. Asemi Z, Samimi M, Tabassi Z, Shakeri H, Esmaillzadeh A. Vitamin D supplementation affects serum high-sensitivity C-reactive protein, insulin resistance, and biomarkers of oxidative stress in pregnant women. J Nutr. 2013;143(9):1432-8. doi:10.3945/jn.113.177550

312. World Health Organization. Effect of reduced sodium intake on cardiovascular disease, coronary heart disease and stroke. Geneva: World Health Organization; 2012.

313. Cohen HW, Hailpern SM, Alderman MH. Sodium intake and mortality follow-up in the Third National Health and Nutrition Examination Survey (NHANES III). J Gen Intern Med. 2008;23(9):1297-302. doi:10.1007/s11606-008-0645-6

314. He J, Ogden LG, Vupputuri S, Bazzano LA, Loria C, Whelton PK. Dietary sodium intake and subsequent risk of cardiovascular disease in overweight adults. JAMA. 1999;282(21):2027-34. doi:10.1001/jama.282.21.2027

315. Umesawa M, Iso H, Date C, et al. Relations between dietary sodium and potassium intakes and mortality from cardiovascular disease: the Japan Collaborative Cohort Study for Evaluation of Cancer Risks. Am J Clin Nutr. 2008;88(1):195-202. doi:10.1093/ajcn/88.1.195

316. Curhan GC, Willett WC, Speizer FE, Spiegelman D, Stampfer MJ. Comparison of dietary calcium with supplemental calcium and other nutrients as factors affecting the risk for kidney stones in women. Ann Intern Med. 1997;126(7):497-504. doi:10.7326/0003-4819-126-7-199704010-00001

317. Ferraro PM, Curhan GC, Sorensen MD, Gambaro G, Taylor EN. Physical activity, energy intake and the risk of incident kidney stones. J Urol. 2015;193(3):864-8. doi:10.1016/j.juro.2014.09.010

318. Hartman TJ, Albanes D, Pietinen P, et al. The association between baseline vitamin E, selenium, and prostate cancer in the alpha-tocopherol, beta-carotene cancer prevention study. Cancer Epidemiol Biomarkers Prev. 1998;7(4):335-40.

319. Peters U, Littman AJ, Kristal AR, Patterson RE, Potter JD, White E. Vitamin E and selenium supplementation and risk of prostate cancer in the Vitamins and lifestyle (VITAL) study cohort. Cancer Causes Control. 2008;19(1):75-87. doi:10.1007/s10552-007-9072-y

320. Maruti SS, Ulrich CM, White E. Folate and one-carbon metabolism nutrients from supplements and diet in relation to breast cancer risk. Am J Clin Nutr. 2009;89(2):624-33. doi:10.3945/ajcn.2008.26568

321. Oaks BM, Dodd KW, Meinhold CL, Jiao L, Church TR, Stolzenberg-Solomon RZ. Folate intake, post-folic acid grain fortification, and pancreatic cancer risk in the Prostate, Lung, Colorectal, and Ovarian Cancer Screening Trial. Am J Clin Nutr. 2010;91(2):449-55. doi:10.3945/ajcn.2009.28433

322. Skinner HG, Michaud DS, Giovannucci EL, et al. A prospective study of folate intake and the risk of pancreatic cancer in men and women. Am J Epidemiol. 2004;160(3):248-58. doi:10.1093/aje/kwh214

323. Slatore CG, Littman AJ, Au DH, Satia JA, White E. Long-term use of supplemental multivitamins, vitamin C, vitamin E, and folate does not reduce the risk of lung cancer. Am J Respir Crit Care Med. 2008;177(5):524-30. doi:10.1164/rccm.200709-1398OC

324. Stolzenberg-Solomon RZ, Chang SC, Leitzmann MF, et al. Folate intake, alcohol use, and postmenopausal breast cancer risk in the Prostate, Lung, Colorectal, and Ovarian Cancer Screening Trial. Am J Clin Nutr. 2006;83(4):895-904. doi:10.1093/ajcn/83.4.895

325. Brough L, Rees GA, Crawford MA, Morton RH, Dorman EK. Effect of multiple-micronutrient supplementation on maternal nutrient status, infant birth weight and gestational age at birth in a low-income, multi-ethnic population. Br J Nutr. 2010;104(3):437-45. doi:10.1017/S0007114510000747

326. Kirke PN, Daly LE, Elwood JH. A randomised trial of low dose folic acid to prevent neural tube defects. The Irish Vitamin Study Group. Arch Dis Child. 1992;67(12):1442-6. doi:10.1136/adc.67.12.1442

327. Li Z, Ye R, Zhang L, Li H, Liu J, Ren A. Folic acid supplementation during early pregnancy and the risk of gestational hypertension and preeclampsia. Hypertension. 2013;61(4):873-9. doi:10.1161/hypertensionaha.111.00230

328. Oken E, Ning Y, Rifas-Shiman SL, Rich-Edwards JW, Olsen SF, Gillman MW. Diet during pregnancy and risk of preeclampsia or gestational hypertension. Ann Epidemiol. 2007;17(9):663-8. doi:10.1016/j.annepidem.2007.03.003

329. Timmermans S, Jaddoe VW, Silva LM, et al. Folic acid is positively associated with uteroplacental vascular resistance: the Generation R study. Nutr Metab Cardiovasc Dis. 2011;21(1):54-61. doi:10.1016/j.numecd.2009.07.002

330. Bodnar LM, Tang G, Ness RB, Harger G, Roberts JM. Periconceptional multivitamin use reduces the risk of preeclampsia. Am J Epidemiol. 2006;164(5):470-7. doi:10.1093/aje/kwj218

331. Bukowski R, Malone FD, Porter FT, et al. Preconceptional folate supplementation and the risk of spontaneous preterm birth: a cohort study. PLoS Med. 2009;6(5):e1000061. doi:10.1371/journal.pmed.1000061

332. Catov JM, Nohr EA, Bodnar LM, Knudson VK, Olsen SF, Olsen J. Association of periconceptional multivitamin use with reduced risk of preeclampsia among normal-weight women in the Danish National Birth Cohort. Am J Epidemiol. 2009;169(11):1304-11. doi:10.1093/aje/kwp052

333. Catov JM, Bodnar LM, Olsen J, Olsen S, Nohr EA. Periconceptional multivitamin use and risk of preterm or small-for-gestational-age births in the Danish National Birth Cohort. Am J Clin Nutr. 2011;94(3):906-12. doi:10.3945/ajcn.111.012393

334. Li Q, Xu S, Chen X, et al. Folic Acid Supplement Use and Increased Risk of Gestational Hypertension. Hypertension. 2020;76(1):150-6. doi:10.1161/HYPERTENSIONAHA.119.14621

335. Liu X, Lv L, Zhang H, et al. Folic acid supplementation, dietary folate intake and risk of preterm birth in China. Eur J Nutr. 2016;55(4):1411-22. doi:10.1007/s00394-015-0959-1

336. Martinussen MP, Bracken MB, Triche EW, Jacobsen GW, Risnes KR. Folic acid supplementation in early pregnancy and the risk of preeclampsia, small for gestational age offspring and preterm delivery. Eur J Obstet Gynecol Reprod Biol. 2015;195:94-9. doi:10.1016/j.ejogrb.2015.09.022

337. Vanderlelie J, Scott R, Shibl R, Lewkowicz J, Perkins A, Scuffham PA. First trimester multivitamin/mineral use is associated with reduced risk of pre-eclampsia among overweight and obese women. Matern Child Nutr. 2016;12(2):339-48. doi:10.1111/mcn.12133

338. Wang Y, Zhao N, Qiu J, et al. Folic acid supplementation and dietary folate intake, and risk of preeclampsia. Eur J Clin Nutr. 2015;69(10):1145-50. doi:10.1038/ejcn.2014.295

339. Wen SW, Chen XK, Rodger M, et al. Folic acid supplementation in early second trimester and the risk of preeclampsia. Am J Obstet Gynecol. 2008;198(1):45 e1-7. doi:10.1016/j.ajog.2007.06.067

340. Wen SW, Guo Y, Rodger M, et al. Folic Acid Supplementation in Pregnancy and the Risk of Pre-Eclampsia-A Cohort Study. PLoS One. 2016;11(2):e0149818. doi:10.1371/journal.pone.0149818

341. Maraini G, Williams SL, Sperduto RD, et al. A randomized, double-masked, placebo-controlled clinical trial of multivitamin supplementation for age-related lens opacities. Clinical trial of nutritional supplements and age-related cataract report no. 3. Ophthalmology. 2008;115(4):599-607.e1. doi:10.1016/j.ophtha.2008.01.005

342. Sperduto RD, Hu TS, Milton RC, et al. The Linxian cataract studies. Two nutrition intervention trials. Arch Ophthalmol. 1993;111(9):1246-53. doi:10.1001/archopht.1993.01090090098027

343. Papadaki A, Scott JA. Follow-up of a web-based tailored intervention promoting the Mediterranean diet in Scotland. Patient Educ Couns. 2008;73(2):256-63. doi:10.1016/j.pec.2008.05.030

344. Gómez P, Fernández de la Puebla RA, Castro P, et al. Efecto de la dieta mediterránea en los valores plasmáticos de factor VII activado en personas sanas. Revista Española de Cardiología. 2005. doi:10.1157/13072476

345. Fowler SP, Williams K, Resendez RG, Hunt KJ, Hazuda HP, Stern MP. Fueling the obesity epidemic? Artificially sweetened beverage use and long-term weight gain. Obesity (Silver Spring). 2008;16(8):1894-900. doi:10.1038/oby.2008.284

346. Ebbeling CB, Feldman HA, Osganian SK, Chomitz VR, Ellenbogen SJ, Ludwig DS. Effects of decreasing sugar-sweetened beverage consumption on body weight in adolescents: a randomized, controlled pilot study. Pediatrics. 2006;117(3):673-80. doi:10.1542/peds.2005-0983

347. James J, Thomas P, Cavan D, Kerr D. Preventing childhood obesity by reducing consumption of carbonated drinks: cluster randomised controlled trial. BMJ. 2004;328(7450):1237. doi:10.1136/bmj.38077.458438.EE

348. Paineau DL, Beaufils F, Boulier A, et al. Family dietary coaching to improve nutritional intakes and body weight control: a randomized controlled trial. Arch Pediatr Adolesc Med. 2008;162(1):34-43. doi:10.1001/archpediatrics.2007.2

349. Brestrich M, Claus J, Blumchen G. Lactovegetarian dict: Influence on weight, lipids, fibrinogen and lipoprotein (a) of heart-patients in the course of their clinical rehabilitation. Z Kardiol. 1996;85(6):418-27.

350. Vernooij RWM, Zeraatkar D, Han MA, et al. Patterns of Red and Processed Meat Consumption and Risk for Cardiometabolic and Cancer Outcomes: A Systematic Review and Meta-analysis of Cohort Studies. Ann Intern Med. 2019;171(10):732-41. doi:10.7326/M19-1583

351. Zeraatkar D, Johnston BC, Bartoszko J, et al. Effect of Lower Versus Higher Red Meat Intake on Cardiometabolic and Cancer Outcomes: A Systematic Review of Randomized Trials. Ann Intern Med. 2019;171(10):721-31. doi:10.7326/M19-0622

352. Shea BJ, Reeves BC, Wells G, et al. AMSTAR 2: a critical appraisal tool for systematic reviews that include randomised or non-randomised studies of healthcare interventions, or both. BMJ. 2017;358:j4008. doi:10.1136/bmj.j4008

353. Adler AJ, Taylor F, Martin N, Gottlieb S, Taylor RS, Ebrahim S. Reduced dietary salt for the prevention of cardiovascular disease. Cochrane Database Syst Rev. 2014(12):Cd009217. doi:10.1002/14651858.CD009217.pub3

354. Palacios C, Trak-Fellermeier MA, Martinez RX, et al. Regimens of vitamin D supplementation for women during pregnancy. Cochrane Database Syst Rev. 2019;10:Cd013446. doi:10.1002/14651858.Cd013446

355. Yuan Y, Tai W, Xu P, et al. Association of maternal serum 25-hydroxyvitamin D concentrations with risk of preeclampsia: a nested case-control study and meta-analysis. J Matern Fetal Neonatal Med. 2019:1-10. doi:10.1080/14767058.2019.1640675

356. Abdelhamid AS, Brown TJ, Brainard JS, et al. Omega-3 fatty acids for the primary and secondary prevention of cardiovascular disease. Cochrane Database Syst Rev. 2018;11(11):Cd003177. doi:10.1002/14651858.CD003177.pub4

357. Wei J, Hou R, Xi Y, et al. The association and dose-response relationship between dietary intake of alpha-linolenic acid and risk of CHD: a systematic review and meta-analysis of cohort studies. Br J Nutr. 2018;119(1):83-9. doi:10.1017/s0007114517003294

358. Abdelhamid AS, Martin N, Bridges C, et al. Polyunsaturated fatty acids for the primary and secondary prevention of cardiovascular disease. Cochrane Database Syst Rev. 2018;11(11):Cd012345. doi:10.1002/14651858.CD012345.pub3

359. Hooper L, Al-Khudairy L, Abdelhamid AS, et al. Omega-6 fats for the primary and secondary prevention of cardiovascular disease. Cochrane Database Syst Rev. 2018;11:Cd011094. doi:10.1002/14651858.CD011094.pub4

360. Bjelakovic G, Gluud LL, Nikolova D, et al. Vitamin D supplementation for prevention of mortality in adults. Cochrane Database Syst Rev. 2014(1):Cd007470. doi:10.1002/14651858.CD007470.pub3

361. Bjelakovic G, Gluud LL, Nikolova D, et al. Vitamin D supplementation for prevention of cancer in adults. Cochrane Database Syst Rev. 2014(6):Cd007469. doi:10.1002/14651858.CD007469.pub2

362. Hossain S, Beydoun MA, Beydoun HA, Chen X, Zonderman AB, Wood RJ. Vitamin D and breast cancer: A systematic review and meta-analysis of observational studies. Clin Nutr ESPEN. 2019;30:170-84. doi:10.1016/j.clnesp.2018.12.085

363. De-Regil LM, Peña-Rosas JP, Fernández-Gaxiola AC, Rayco-Solon P. Effects and safety of periconceptional oral folate supplementation for preventing birth defects. Cochrane Database of Systematic Reviews. 2015(12). doi:10.1002/14651858.CD007950.pub3

364. Rees K, Takeda A, Martin N, et al. Mediterranean-style diet for the primary and secondary prevention of cardiovascular disease. Cochrane Database Syst Rev. 2019;3:Cd009825. doi:10.1002/14651858.CD009825.pub3

365. Rosato V, Temple NJ, La Vecchia C, Castellan G, Tavani A, Guercio V. Mediterranean diet and cardiovascular disease: a systematic review and meta-analysis of observational studies. Eur J Nutr. 2019;58(1):173-91. doi:10.1007/s00394-017-1582-0

366. Mathew MC, Ervin AM, Tao J, Davis RM. Antioxidant vitamin supplementation for preventing and slowing the progression of age-related cataract. Cochrane Database Syst Rev. 2012(6):Cd004567. doi:10.1002/14651858.CD004567.pub2

367. Keats EC, Haider BA, Tam E, Bhutta ZA. Multiple-micronutrient supplementation for women during pregnancy. Cochrane Database Syst Rev. 2019;3:Cd004905. doi:10.1002/14651858.CD004905.pub6

368. Avenell A, Mak JC, O'Connell D. Vitamin D and vitamin D analogues for preventing fractures in post-menopausal women and older men. Cochrane Database Syst Rev. 2014(4):Cd000227. doi:10.1002/14651858.CD000227.pub4

369. Feng Y, Cheng G, Wang H, Chen B. The associations between serum 25-hydroxyvitamin D level and the risk of total fracture and hip fracture. Osteoporos Int. 2017;28(5):1641-52. doi:10.1007/s00198-017-3955-x

370. Rees K, Hartley L, Day C, Flowers N, Clarke A, Stranges S. Selenium supplementation for the primary prevention of cardiovascular disease. Cochrane Database Syst Rev. 2013;2013(1):Cd009671. doi:10.1002/14651858.CD009671.pub2

371. Xiang S, Dai Z, Man C, Fan Y. Circulating Selenium and Cardiovascular or All-Cause Mortality in the General Population: a Meta-Analysis. Biol Trace Elem Res. 2019. doi:10.1007/s12011-019-01847-8

372. Hu L, Zhang Y, Wang X, et al. Maternal Vitamin D Status and Risk of Gestational Diabetes: a Meta-Analysis. Cell Physiol Biochem. 2018;45(1):291-300. doi:10.1159/000486810

373. Tous M, Villalobos M, Iglesias L, Fernandez-Barres S, Arija V. Vitamin D status during pregnancy and offspring outcomes: a systematic review and meta-analysis of observational studies. Eur J Clin Nutr. 2020;74(1):36-53. doi:10.1038/s41430-018-0373-x

374. Usinger L, Reimer C, Ibsen H. Fermented milk for hypertension. Cochrane Database Syst Rev. 2012(4):Cd008118. doi:10.1002/14651858.CD008118.pub2

375. Soedamah-Muthu SS, Verberne LD, Ding EL, Engberink MF, Geleijnse JM. Dairy consumption and incidence of hypertension: a dose-response meta-analysis of prospective cohort studies. Hypertension. 2012;60(5):1131-7. doi:10.1161/hypertensionaha.112.195206

376. Rees K, Dyakova M, Wilson N, Ward K, Thorogood M, Brunner E. Dietary advice for reducing cardiovascular risk. Cochrane Database Syst Rev. 2013;2013(12):Cd002128. doi:10.1002/14651858.CD002128.pub5
